# Syntheses of 1-Aryl-5-nitro-1*H*-indazoles and a General One-Pot Route to 1-Aryl-1*H*-indazoles

**DOI:** 10.3390/molecules23030674

**Published:** 2018-03-16

**Authors:** Joel K. Annor-Gyamfi, Krishna Kumar Gnanasekaran, Richard A. Bunce

**Affiliations:** Department of Chemistry, Oklahoma State University, Stillwater, OK 74078-3071, USA; jannorg@okstate.edu (J.K.A.-G.); krishna.gnanasekaran@okstate.edu (K.K.G.)

**Keywords:** 1-aryl-5-nitro-1*H*-indazole, 1-aryl-1*H*-indazole, arylhydrazone, S_N_Ar reaction, Ullmann reaction

## Abstract

An efficient route to substituted 1-aryl-1*H*-indazoles has been developed and optimized. The method involved the preparation of arylhydrazones from acetophenone or benzaldehyde substituted by fluorine at C2 and nitro at C5, followed by deprotonation and nucleophilic aromatic substitution (S_N_Ar) ring closure in 45–90%. Modification of this procedure to a one-pot domino process was successful in the acetophenone series (73–96%), while the benzaldehyde series (63–73%) required a step-wise addition of reagents. A general one-pot protocol for 1-aryl-1*H*-indazole formation without the limiting substitution patterns required for the S_N_Ar cyclization has also been achieved in 62–78% yields. A selection of 1-aryl-1*H*-indazoles was prepared in high yield by a procedure that requires only a single laboratory operation.

## 1. Introduction

**Figure 1:**
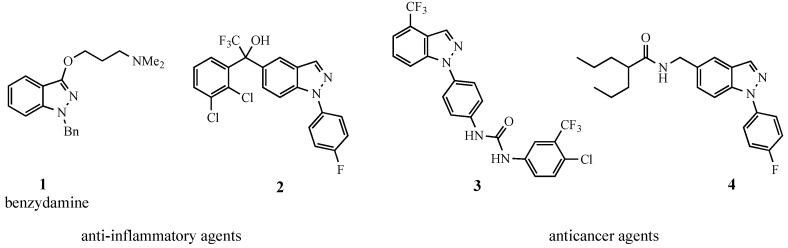
Drugs incorporating 1-aryl-1*H*-indazoles.

Our synthetic methods program has recently been focused on the use of nucleophilic aromatic substitution (S_N_Ar)-terminated domino reactions as a means to prepare heterocyclic compounds [1,2,3,4]. The current project has developed a synthesis of 1-aryl-1*H*-indazoles, which are core ring structures in a broad range of biologically active compounds (Figure 1). Among the compounds pictured, benzydamine (**1**) is a well-known NSAID drug [5], while alcohol **2** has likewise shown promise as an anti-inflammatory agent [6]. The urea-based structure **3** has recently been judged to possess significant tumor antiproliferative properties [7]. Similarly, amide **4** has also demonstrated anticancer activity [8,9].

Several strategies have previously been disclosed as routes to 1-aryl-1*H*-indazoles. All but one required multiple steps. The closest work to that presented here utilized a two-step sequence involving acid catalyzed formation of the arylhydrazone, followed by a copper(I)-catalyzed Ullmann-type coupling to close the ring [10]. A related investigation described a similar cyclization of arylhydrazones from 2-(2-haloaryl)-2-oxoacetic esters [11]. A complementary approach utilized a copper(I)-promoted coupling of *N*-aroyl-*N*′-arylhydrazines with 2-bromoaryl ketones or aldehydes [12], followed by deacylation and condensative ring closure. Two additional accounts examined (1) an S_N_Ar displacement of nitro by anions derived from arylhydrazones of 2-nitrobenzaldehydes [13] and (2) a one-step conversion from salicylaldehydes and arylhydrazine hydrochlorides [14]. Other reports have described a one-pot condensation-S_N_Ar protocol using 2-fluorobenzaldehyde to prepare 1*H*-indazoles, but these utilized more reactive hydrazines and did not yield 1-aryl-substituted derivatives [15,16]. Along a different line, another study delineated a novel [3 + 2]-type annulation between arynes and arylhydrazones in the presence of fluoride under aerobic conditions [17]. Finally, syntheses promoted by palladium- [18] and rhodium-based [19] catalysts have also furnished these targets. A full listing of methods to prepare 1*H*-indazoles, with and without the N1 aryl group, have been cataloged in three published reviews [20,21,22].

The number of steps and the waste generated are two important considerations in planning synthetic procedures. Our aim, in the current study, was to develop a one-pot domino or consecutive process [23,24,25] to accomplish the synthesis of 1-aryl-1*H*-indazoles. Utilizing these techniques, a single operation—one set-up and one work-up—would generate members of this emerging class of heterocyclic building blocks rapidly and with a minimum of chemical waste. The details of our efforts towards this goal are given below. 

## 2. Results and Discussion

A logical route to 1*H*-indazoles would involve the ring closure of an arylhydrazone anion derived from an appropriately substituted aromatic ketone or aldehyde. Although the arylhydrazones would form as two isomers, with the *E* isomer (less favorable for cyclization) predominating, these should readily interconvert with heat [26] and allow access to the *Z* isomer required for ring closure. Furthermore, the pKa of the phenylhydrazone proton (Ar = Ph) is 21.5 [27], and with slight variations due to substituents on the aromatic ring, should permit the use of K_2_CO_3_ in a polar aprotic solvent as the deprotonating agent [28,29]. Optimally, these annulations would proceed in one pot, but we initially sought to determine the feasibility of the sequence by evaluating each transformation separately. Once each step was optimized, attempts would then be made to merge the two reactions into a single laboratory operation. Finally, since this would generate structures with limited substitution patterns, we also aspired to generalize the process to include structures without S_N_Ar activating groups.

**Scheme 1:**
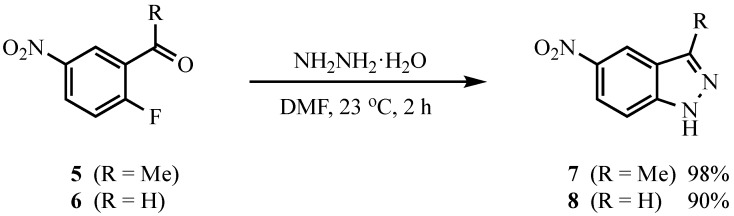
Reaction of **5** and **6** with hydrazine hydrate.

Although there are numerous examples of S_N_Ar-type reactions to generate five-membered rings fused to a benzene, some strain would be expected in ring formations involving closure of a fragment incorporating three sp^2^ atoms onto an aromatic nucleus [30,31]. Thus, 2′-fluoro-5′-nitroacetophenone (**5**) and 2-fluoro-5-nitrobenzaldehyde (**6**) were initially reacted with hydrazine hydrate (NH_2_NH_2_·H_2_O) to assess the feasibility of forming indazoles in this manner. Due to the alpha-effect [32], hydrazine is an exceptionally potent nucleophile and might be expected to react without added base. Indeed, treatment of **5** and **6** with NH_2_NH_2_·H_2_O (3.0 equivalents for **5** and 2.0 equivalents for **6**) in *N*,*N*-dimethylformamide (DMF) at 23 °C for two hours resulted in high yields of targets **7** and **8**, respectively (see Scheme 1). Encouraged by this result, we forged ahead to prepare 1-aryl-5-nitro-1*H*-indazoles.

Formation of the intermediate arylhydrazones **9** and **10** was initially addressed. This involved warming a DMF solution of the carbonyl compound with an arylhydrazine hydrochloride (ArNHNH_2_·HCl) at 50 °C for two to three hours. DMF was used as a solvent for this process since we anticipated that a one-pot procedure where the hydrazone was prepared, deprotonated, and cyclized would likely require the use of a polar aprotic solvent. Optimization studies indicated that DMF was a suitable medium and the use of 3.0 equivalents of ArNHNH_2_·HCl with acetophenone **5** and 2.0 equivalents with benzaldehyde **6** afforded the highest yields of **9** and **10**. In all cases, the intermediate hydrazones were isolated in ≥70% yield from **5** and ≥50% yield from **6**. Interestingly, compounds arising from direct S_N_Ar displacement of fluoride by the arylhydrazine were not observed to any significant extent under these conditions. While the product of addition–elimination by hydrazine is known to cyclize to a 1*H*-indazole [33,34], ring closure of the analogous product from arylhydrazine would give a 2-aryl-2*H*-indazole. Neither the arylhydrazine S_N_Ar product nor the 2*H*-indazole were detected.

Cyclization of hydrazones **9** and **10** required base, higher temperature and occasionally extended reaction times. This reaction was performed in DMF at 90 °C and found to work best using 3.0 equivalents of K_2_CO_3_. Yields were slightly lower from the benzaldehyde since this substrate led to a product that was unhindered at C3 and possibly susceptible to nucleophilic attack by various species in the reaction. A summary of optimization experiments is given in the Electronic Supplemental Inofrmation, and the results of our two-step synthesis of 1-aryl-5-nitro-1*H*-indazoles are summarized in Table 1.

Modification of the protocol to allow for a one-pot procedure had to overcome several problems that could lead to unwanted impurities. Since water is produced during arylhydrazone formation, some conversion of the K_2_CO_3_ to KOH would be expected. Furthermore, KOH could hydrolyze the DMF solvent to form Me_2_NH. Both of these nucleophiles (OH^–^ and Me_2_NH) could potentially undergo competitive S_N_Ar addition to the carbonyl containing substrate. While no significant product resulting from OH^–^ addition was observed, some of the Me_2_NH addition product was noted when prolonged heating was required. These side reactions were suppressed by adding 30 wt % (relative to **5** or **6**) of powdered 4 Å molecular sieves to scavenge water produced during the initial condensation and using anhydrous 1,3-dimethyl-3,4,5,6-tetrahydro-2(1*H*)-pyrimidinone (DMPU) as the solvent to minimize hydrolysis.

The one-step domino process was successful for the conversion of ketone **5** to indazoles **11**, and in most cases, the yield was superior to the two-step sequence (see Table 2). Reactions of aldehyde **6** to give **12**, however, were more limited and several of the substrates bearing acidic functions on the aromatic ring failed to react. For aldehydes that did generate a product, sequential treatment with ArNHNH_2_·HCl and base afforded yields that also surpassed the two-step process. In both one-pot procedures, it was often noted that the cyclization required a longer reaction time when the aryl group of the hydrazone was substituted by an electron-withdrawing group. This would be expected since the arylhydrazone anion would be less nucleophilic with a stabilizing group at C2 or C4 of the hydrazone aromatic ring [35]. Conversely, this same anion with an electron-donating substituent at C2 or C4 should be more reactive with potentially shorter reaction times.

Finally, we sought to develop a general approach to 1*H*-indazoles unrestricted by the substitution patterns needed for the S_N_Ar ring closure (see Table 3). Protocol development efforts indicated that this variant of the reaction required sequential addition of the reagents. The hydrazone was initially generated using 1.5 equivalents of the ArNHNH_2_·HCl and then cyclized by addition of 20 mol % of copper(I) iodide (CuI) and 2.5 equivalents of K_2_CO_3_, all in the same flask at 90 °C. Again, the key to success in this transformation was the inclusion of 30 wt % of powdered 4 Å molecular sieves and the use of DMPU as the solvent. Without activating groups on the ring, Ullmann conditions (CuI and base) for the final ring closure were essential. An earlier paper noted that this copper(I)-catalyzed ring closure was facilitated by the addition of 20 mol % of l-proline or 4-hydroxy-l-proline [8,9]. In the current procedure, however, addition of these ligands had no impact on the yield of the reaction. The cyclization using 2′-bromoacetophenone (**13**), 2-bromobenzaldehyde (**14**) and 2-chloronicotinaldehyde (**15**) was performed using phenylhydrazine as well as electron-rich and electron-poor arylhydrazines in relatively uniform yields. Though it was expected that the nicotinaldehyde derivatives would not need a copper catalyst, since it could directly cyclize by a S_N_Ar reaction, this ring closure failed to proceed to completion without this additive. Overall, our procedure permits the rapid construction of 1-aryl-1*H*-indazoles in a single reaction vessel without isolation or purification of intermediates.

## 3. Experimental Section

### 3.1. General Methods

Unless otherwise indicated, all reactions were carried out under dry N_2_ in oven-dried glassware. All reagents and solvents were used as received. Reactions were monitored by thin layer chromatography on silica gel GF plates (Analtech No. 21521, Newark, DE, USA). Preparative separations were performed by flash chromatography on silica gel (Davisil^®^, grade 62, 60–200 mesh, Sorbent Technologies, Norcross, GA, USA) containing UV-active phosphor (Sorbent Technologies No. UV-05) slurry packed into quartz columns. Band elution for all chromatographic separations was monitored using a hand-held UV lamp (Fisher Scientific, Pittsburgh, PA, USA). Melting points were obtained using a MEL-TEMP apparatus (Laboratory Devices, Cambridge, MA, USA) and are uncorrected. FT-IR spectra were run using a Varian Scimitar FTS 800 spectrophotometer (Varian Instruments, Randolph, MA, USA) as thin films or nujol mulls on NaCl disks. ^1^H- and ^13^C-NMR spectra were measured using a Bruker Avance 400 system (Bruker Corporation, Billerica, MA, USA) in the indicated solvents at 400 MHz and 101 MHz, respectively, with (CH_3_)_4_Si as the internal standard; coupling constants (*J*) are given in Hz. Unit resolution mass spectra were obtained using a Shimadzu LCMS-2010EV system (Shimadzu Corporation, Columbia, MD, USA). Elemental analyses (±0.4%) were determined by Atlantic Microlabs (Norcross, GA, USA).

### 3.2. Indazoles from 2′-Fluoro-5′-nitroacetophenone *(**5**)* or 2-Fluoro-5-nitrobenzaldehyde *(**6**)* with NH_2_NH_2_·H_2_O

To a stirred solution of **5** or **6** (1.0 mmol) in DMF (5 mL) at 23 °C was added NH_2_NH_2_·H_2_O (3.0 mmol for **5**, 2.0 mmol for **6**). The solution was stirred at 23 °C for 2 h at which time thin layer chromatography (TLC, 20% EtOAc in hexanes) indicated complete consumption of the starting carbonyl compound. The crude mixture was added to water and extracted with EtOAc (2 × 15 mL). The combined organic layers were washed with water and saturated aq NaCl, dried (MgSO_4_), filtered, and concentrated under vacuum to give the pure indazole products.

*3-Methyl-5-nitro-1H-indazole* (**7**). Yield: 173 mg (0.98 mmol, 98%) as a yellow solid, m.p. 213–214 °C; IR: 1517, 1332 cm^−1^; ^1^H-NMR (DMSO-*d*_6_): δ 13.1 (br s, 1H), 8.79 (s, 1H), 8.18 (d, *J* = 9.1 Hz, 1H), 7.64 (d, *J* = 9.1 Hz, 1H), 2.59 (s, 3H); ^13^C-NMR (DMSO-*d*_6_): δ 145.4, 143.1, 141.3, 121.9, 121.3, 118.8, 111.2, 12.1; MS: *m*/*z* 177 (M^+^), calculated *m*/*z* 177.05. Anal. Calcd. for C_8_H_7_N_3_O_2_: C, 54.24; H, 3.98; N, 23.72. Found: C, 54.29; H, 4.01; N, 23.59.

*5-Nitro-1H-indazole* (**8**). Yield: 147 mg (0.90 mmol, 90%) as a tan solid, m.p. 207–208 °C; IR: 1534, 1341 cm^−1^; ^1^H-NMR (DMSO-*d*_6_): δ 13.7 (br s, 1H), 8.84 (s, 1H), 8.41 (s, 1H), 8.19 (d, *J* = 9.0 Hz, 1H), 7.74 (d, *J* = 9.0 Hz, 1H); ^13^C-NMR (DMSO-*d*_6_): δ 142.3, 142.0, 137.2, 122.5, 121.3, 119.3, 111.5; MS: *m*/*z* 163 (M^+^), calculated *m*/*z* 163.04. Anal. Calcd. for C_7_H_5_N_3_O_2_: C, 51.54; H, 3.09; N, 25.76. Found: C, 51.85; H, 3.15; N, 25.54.

### 3.3. Indazoles from ***5*** or ***6*** with ArNHNH_2_·HCl. Method A. Representative Two-Step Procedure

To a stirred solution of the carbonyl compound (1.0 mmol) in DMF (5 mL) at 50 °C (oil bath) was added ArNHNH_2_·HCl (3.0 mmol for **5**, 2.0 mmol for **6**) and the solution was stirred until TLC (20% EtOAc in hexanes) indicated complete conversion (roughly 2 h). The crude reaction mixture was added to water and extracted with EtOAc (2 × 15 mL). The combined organic layers were washed with water and saturated aq NaCl, dried (MgSO_4_), filtered, and concentrated under vacuum to give the crude hydrazones. The crude products were stirred with 20% ether/pentane for 1 h, filtered and dried to give arylhydrazones **9** and **10**. Characterization data for these materials are given in the ESI.

To a stirred solution of the arylhydrazone (1.00 mmol) in DMF (5 mL) at 90 °C (oil bath) was added anhydrous K_2_CO_3_ (3.0 mmol) and the mixture was stirred for the time indicated in Table 1. The reaction mixture was added to water, extracted with EtOAc, dried (MgSO_4_), filtered, and concentrated under vacuum to afford the crude product. Indazoles from **5** were stirred with 20% ether in pentane, filtered and dried, while those from **6** were purified by silica gel chromatography on a 20 cm × 2 cm column eluted with increasing concentrations of EtOAc in hexanes. Yields as well as physical and spectral data are given below.

### 3.4. Indazoles from ***5*** or ***6*** with ArNHNH_2_·HCl. Method B. Representative One-Pot Procedure

To a stirred solution of the carbonyl com.p.ound (1.0 mmol) in DMPU (5 mL) were added powdered 4 Å molecular sieves (30 wt % relative to the carbonyl substrate), ArNHNH_2_·HCl (3.0 mmol for **5**, 2.0 mmol for **6**), and K_2_CO_3_ (3.0 mmol for **5**, 2.0 mmol for **6**). For **5**, all reagents were placed in the flask and heated to 90 °C; for **6**, the hydrazone was allowed to form at 90 °C (1.5 h) before K_2_CO_3_ was added. The reaction was stirred at 90 °C for the time indicated in Table 2. Workup was performed as described in Method A.

*3-Methyl-1-phenyl-5-nitro-1H-indazole* (**11a**). Yield: 240 mg, (0.95 mmol, 95%) as a brown solid, m.p. 115–117 °C; IR: 1516, 1336 cm^−1^; ^1^H-NMR (DMSO-*d*_6_): δ 8.89 (d, *J* = 2.2 Hz, 1H), 8.28 (dd, *J* = 9.3, 2.2 Hz, 1H), 7.93 (d, *J* = 9.3 Hz, 1H), 7.77 (d, *J* = 7.8 Hz, 2H), 7.62 (t, *J* = 7.7 Hz, 2H), 7.46 (t, *J* = 7.4 Hz, 1H), 2.69 (s, 3H); ^13^C-NMR (DMSO-*d*_6_): δ 147.3, 142.2, 141.1, 139.2, 130.3, 127.8, 124.4, 122.9, 122.8, 119.3, 111.7, 12.1; MS: *m*/*z* 253 (M^+^), calculated *m*/*z* 253.09. Anal. Calcd. for C_14_H_11_N_3_O_2_: C, 66.40; H, 4.38; N, 16.59. Found: C, 66.33; H, 4.34; N, 16.42.

*1-(2-Methoxyphenyl)-3-methyl-5-nitro-1H-indazole* (**11b**). Yield: 232 mg (0.82 mmol, 82%) as a light brown solid, m.p. 128–130 °C; IR: 2838, 1519, 1338 cm^−1^; ^1^H-NMR (DMSO-*d*_6_): δ 8.86 (s, 1H), 8.21 (d, *J* = 9.3 Hz, 1H), 7.55 (t, *J* = 8.0 Hz, 1H), 7.49 (d, *J* = 7.8 Hz, 1H), 7.38–7.28 (overlapping signals, 2H), 7.16 (t, *J* = 7.7 Hz, 1H), 3.78 (s, 3H), 2.66 (s, 3H); ^13^C-NMR (DMSO-*d*_6_): δ 154.0, 146.6, 143.0, 141.9, 130.8, 128.6, 127.1, 123.2, 122.0, 121.4, 119.0, 113.4, 112.2, 56.2, 12.1; MS: *m*/*z* 283 (M^+^), calculated *m*/*z* 283.10. Anal. Calcd. for C_15_H_13_N_3_O_3_: C, 63.60; H, 4.63; N, 14.83. Found: C, 63.49; H, 4.61; N, 14.88.

*1-(3-Methoxyphenyl)-3-methyl-5-nitro-1H-indazole* (**11c**). Yield: 229 mg (0.81 mmol, 81%) as a light brown solid, m.p. 105–106 °C; IR: 2836, 1518, 1338 cm^−1^; ^1^H-NMR (DMSO-*d*_6_): δ 8.90 (s, 1H), 8.29 (d, *J* = 9.3 Hz, 1H), 7.96 (d, *J* = 9.3 Hz, 1H), 7.52 (t, *J* = 8.1 Hz, 1H), 7.34 (d, *J* = 8.1 Hz, 1H), 7.28 (s, 1H), 7.04 (d, *J* = 8.3 Hz, 1H); ^13^C-NMR (DMSO-*d*_6_): δ 160.6, 147.3, 142.3, 141.2, 140.3, 131.2, 124.4, 122.9, 119.3, 114.9, 113.7, 111.9, 108.6, 56.0, 12.1; MS: *m*/*z* 283 (M^+^), calculated *m*/*z* 283.10. Anal. Calcd. for C_15_H_13_N_3_O_3_: C, 63.60; H, 4.63; N, 14.83. Found: C, 63.52; H, 4.55; N, 14.71.

*1-(4-Methoxyphenyl)-3-methyl-5-nitro-1H-indazole* (**11d**). Yield: 266 mg (0.94 mmol, 94%) as a tan solid, m.p. 159–160 °C. IR: 2836, 1514, 1346 c cm^−1^; ^1^H-NMR (DMSO-*d*_6_): δ 8.88 (d, *J* = 2.2 Hz, 1H), 8.26 (dd, *J* = 9.3, 2.2 Hz, 1H), 7.79 (d, *J* = 9.3 Hz, 1H), 7.65 (d, *J* = 8.1 Hz, 2H), 7.15 (d, *J* = 8.1 Hz, 2H), 3.85 (s, 3H), 2.69 (s, 3H); ^13^C-NMR (DMSO-*d*_6_): δ 158.9, 146.7, 142.0, 141.2, 132.2, 124.8, 123.9, 122.5, 119.3, 1153, 111.4, 56.0, 12.1; MS: *m*/*z* 283 (M^+^), calculated *m*/*z* 283.10. Anal. Calcd. for C_15_H_13_N_3_O_3_: C, 63.60; H, 4.63; N, 14.83. Found: C, 63.57; H, 4.60; N, 14.76.

*1-(4-Bromophenyl)-3-methyl-5-nitro-1H-indazole* (**11e**). Yield: 314 mg, (0.95 mmol, 95%) as a tan solid, m.p. 202–203 °C; IR: 1512, 1345 cm^−1^; ^1^H-NMR (DMSO-*d*_6_): δ 8.90 (d, *J* = 2.2 Hz, 1H), 8.31 (dd, *J* = 9.2, 2.2 Hz, 1H), 7.96 (d, *J* = 9.2 Hz, 1H), 7.80 (d, *J* = 8.8 Hz, 2H), 7.75 (d, *J* = 8.8 Hz, 2H), 2.69 (s, 3H); ^13^C-NMR (DMSO-*d*_6_): δ 147.7, 142.4, 141.1, 138.5, 133.1, 124.7, 124.6, 123.0, 120.1, 119.3, 111.8, 12.1; MS: *m*/*z* 331 (M^+^), calculated *m*/*z* 331.00. Anal. Calcd. for C_14_H_10_BrN_3_O_2_: C, 50.62; H, 3.03; N, 12.65. Found: C, 50.44; H, 2.99; N, 12.73.

*1-(3-Chlorophenyl)-3-methyl-5-nitro-1H-indazole* (**11f**). Yield: 250 mg, (0.87 mmol, 87%) as a tan solid, m.p. 135–136 °C; IR: 1517, 1339 cm^−1^; ^1^H-NMR (DMSO-*d*_6_): δ 8.89 (d, *J* = 2.2 Hz, 1H), 8.31 (dd, *J* = 9.3, 2.2 Hz, 1H), 7.98 (d, *J* = 9.3 Hz, 1H), 7.83 (t, *J* = 2.2 Hz, 1H), 7.78 (d, *J* = 8.4 Hz, 1H), 7.64 (t, *J* = 8.1 Hz, 1H), 7.52 (d, *J* = 8.0 Hz, 1H), 2.69 (s, 3H); ^13^C-NMR (DMSO-*d*_6_): δ 146.8, 141.1, 139.3, 133.4, 130.8, 126.4, 123.6, 122.0, 121.4, 120.1, 118.2, 110.9; MS: *m*/*z* 287 (M^+^), calculated *m*/*z* 287.05. Anal. Calcd. for C_14_H_10_ClN_3_O_2_: C, 58.45; H, 3.50; N, 14.61. Found: C, 58.58; H, 3.59; N, 14.53.

*1-(4-Chlorophenyl)-3-methyl-5-nitro-1H-indazole* (**11g**). Yield: 267 mg (0.93 mmol, 93%) as a tan solid, m.p. 217–218 °C; IR: 1511, 1339 cm^−1^; ^1^H-NMR (DMSO-*d*_6_): δ 8.91 (d, *J* = 2.2 Hz, 1H), 8.31 (dd, *J* = 9.3, 2.2 Hz, 1H), 7.95 (d, *J* = 9.3 Hz, 1H), 7.82 (d, *J* = 8.7 Hz, 2H), 7.67 (d, *J* = 8.7 Hz, 2H), 2.69 (s, 3H); ^13^C-NMR (DMSO-*d*_6_): δ 147.7, 1424, 141.1, 138.1, 131.8, 130.2, 124.6, 124.5, 123.0, 119.4, 111.8, 12.1; MS: *m*/*z* 287 (M^+^), calculated *m*/*z* 287.05. Anal. Calcd. for C_14_H_10_ClN_3_O_2_: C, 58.45; H, 3.50; N, 14.61. Found: C, 58.42; H, 3.49; N, 14.48.

*1-(2,4-Dichlorophenyl)-3-methyl-5-nitro-1H-indazole* (**11h**). Yield: 257 mg (0.80 mmol, 80%), m.p. 144–145 °C; IR: 1516, 1338 cm^−1^; ^1^H-NMR (DMSO-*d*_6_): δ 8.92 (d, *J* = 2.1 Hz, 1H), 8.27 (dd, *J* = 9.2, 2.1 Hz, 1H), 8.01 (d, *J* = 2.2 Hz, 1H), 7.74 (d, *J* = 8.5 Hz, 1H), 7.69 (dd, *J* = 8.5, 2.2 Hz, 1H), 7.44 (d, *J* = 9.2 Hz, 1H), 2.68 (s, 3H); ^13^C-NMR (DMSO-*d*_6_): δ 147.5, 143.1, 142.4, 135.3, 135.0, 132.0, 131.5, 130.7, 129.3, 123.5, 122.8, 119.3, 111.6, 12.1; MS: *m*/*z* 321 (M^+^), calculated *m*/*z* 321.01. Anal. Calcd. for C_14_H_9_Cl_2_N_3_O_2_: C, 52.20; H, 2.82; N, 13.04. Found: C, 52.11; H, 2.91; N, 13.09.

*3-Methyl-5-nitro-1-(3-(trifluoromethyl)phenyl)-1H-indazole* (**11i**). Yield: 282 mg (0.88 mmol, 88%) as a white solid, m.p. 112–113 °C; IR: 1520, 1339 cm^−1^; ^1^H-NMR (DMSO-*d*_6_): δ 8.90 (s, 1H), 8.32 (d, *J* = 9.3 Hz, 1H), 8.13 (d, *J* = 7.8 Hz, 1H), 8.07 (s, 1H), 7.99 (d, *J* = 9.3 Hz, 1H), 7.90–7.77 (complex, 2H), 2.70 (s, 3H); ^13^C-NMR (DMSO-*d*_6_): δ 148.0, 142.5, 141.1, 139.8, 131.6, 130.9 (q, *J* = 32.6 Hz), 126.2, 124.8, 124.2 (q, *J* = 272.6 Hz), 124.0 (q, *J* = 6.7 Hz), 123.1, 119.2 (2C), 111.8, 12.0; MS: *m*/*z* 321 (M^+^), calculated *m*/*z* 321.07. Anal. Calcd. for C_15_H_10_F_3_N_3_O_2_: C, 56.08; H, 3.14; N, 13.08. Found: C, 56.02; H, 3.18; N, 13.11.

*3-Methyl-5-nitro-1-(4-(trifluoromethyl)phenyl)-1H-indazole* (**11j**). Yield: 225 mg, (0.70 mmol, 70%) as a white solid, mp 167–168 °C; IR: 1525, 1331 cm^−1^; ^1^H-NMR (DMSO-*d*_6_): δ 8.89 (d, *J* = 2.2 Hz, 1H), 8.31 (dd, *J* = 9.3, 2.2 Hz, 1H), 8.05 (d, *J* = 9.3 Hz, 1H), 8.02 (d, *J* = 8.6 Hz, 2H), 7.95 (d, *J* = 8.6 Hz, 2H), 2.69 (s, 3H); ^13^C-NMR (DMSO-*d*_6_): δ 148.3, 142.6, 142.3, 141.1, 127.4 (q, *J* = 4.0 Hz), 127.1, 125.1, 124.5 (q, *J* = 269.1 Hz), 123.2, 122.6, 119.2, 112.0, 12.1; MS: *m*/*z* 321 (M^+^). calculated *m*/*z* 321.07. Anal. Calcd. for C_15_H_10_F_3_N_3_O_2_: C, 56.08; H, 3.14; N, 13.08. Found: C, 55.98; H, 3.19; N, 13.04.

*1-(4-Cyanophenyl)-3-methyl-5-nitro-1H-indazole* (**11k**). Yield: 222 mg (0.80 mmol, 80%) as a brown solid, m.p. ≥ 194 °C (sub), ≥ 260 °C (dec); IR: 2226, 1516, 1338 cm^−1^; ^1^H-NMR (DMSO-*d*_6_): δ 8.93 (d, *J* = 2.2 Hz, 1H), 8.35 (dd, *J* = 9.1, 2.2 Hz, 1H), 8.11 (d, *J* = 9.1 Hz, 1H), 8.08 (d, *J* = 8.9 Hz, 2H), 8.04 (d, *J* = 8.9 Hz, 2H), 2.71 (s, 3H); ^13^C-NMR (DMSO-*d*_6_): δ 148.7, 142.8, 142.7, 141.1, 134.5, 125.3, 123.3, 122.6, 119.3, 118.9, 112.3, 109.4, 12.1; MS: *m*/*z* 278 (M^+^), calculated *m*/*z* 278.08. Anal. Calcd. for C_15_H_10_N_4_O_2_: C, 64.74; H, 3.62; N, 20.13. Found: C, 64.69; H, 3.67; N, 20.06.

*4-(3-Methyl-5-nitro-1H-indazol-1-yl)benzenesulfonamide* (**11l**). Yield: 249 mg (0.75 mmol, 75%), m.p. 265–266 °C; IR: 3422, 1515, 1331, 1320, 1123 cm^−1^; ^1^H-NMR (DMSO-*d*_6_): δ 8.92 (d, *J* = 2.2 Hz, 1H), 8.34 (dd, *J* = 9.3, 2.2 Hz, 1H), 8.09 (d, *J* = 9.3 Hz, 1H), 8.04 (d, *J* = 8.4 Hz, 2H), 8.01 (d, *J* = 8.4 Hz, 2H), 7.51 (s, 2H), 2.71 (s, 3H); ^13^C-NMR (DMSO-*d*_6_): δ 148.2, 142.54, 145.50, 141.6, 141.0, 127.9, 125.0, 123.1, 122.3, 119.2, 112.0, 12.0; MS: *m*/*z* 332 (M^+^), calculated *m*/*z* 332.06. Anal. Calcd. for C_14_H_12_N_4_O_4_S: C, 50.60; H, 3.64; N, 16.86. Found: C, 50.70; H, 3.63; N, 16.78.

*4-(3-Methyl-5-nitro-1H-indazol-1-yl)benzoic acid* (**11m**). Yield: 208 mg (0.70 mmol, 70%) as a tan solid, m.p. ≥ 240 °C (sub), ≥ 320 °C (dec). IR: 3441–2352, 1697, 1514, 1346 cm^−1^; ^1^H-NMR (DMSO-*d*_6_): δ 12.8 (br s, 1H), 8.93 (d, *J* = 2.2 Hz, 1H), 8.34 (dd, *J* = 9.3, 2.2 Hz, 1H), 8.16 (d, *J* = 8.6 Hz, 2H), 8.09 (d, *J* = 9.3, Hz, 1H), 7.95 (d, *J* = 8.6 Hz, 2H), 2.71 (s, 3H); ^13^C-NMR (DMSO-*d*_6_): δ 167.1, 148.2, 142.6, 142.5, 141.1, 131.5, 125.0, 123.1, 122.0, 119.4, 119.3, 112.2, 12.1; MS: *m*/*z* 297 (M^+^), calculated *m*/*z* 297.07. Anal. Calcd. for C_15_H_11_N_3_O_4_: C, 60.61; H, 3.73; N, 14.14. Found: C, 60.53; H, 3.68; N, 14.22.

*1-Phenyl-5-nitro-1H-indazole* (**12a**). Yield: 172 mg (0.72 mmol, 72%) as a tan solid, m.p. 178–180 °C (lit m.p. 178–180 °C [18]); IR: 1534, 1341 cm^−1^; ^1^H-NMR (DMSO-*d*_6_): δ 8.95 (s, 1H), 8.70 (s, 1H), 8.30 (d, *J* = 9.3 Hz, 1H), 7.98 (d, *J* = 9.3 Hz, 1H), 7.81 (d, *J* = 7.9 Hz, 2H), 7.65 (d, *J* = 7.7 Hz, 2H), 7.51 (t, *J* = 7.4 Hz, 1H); ^13^C-NMR (DMSO-*d*_6_): δ 142.8, 140.5, 139.1, 138.9, 130.3, 128.2, 124.8, 123.3, 122.7, 119.9, 111.9; MS: *m*/*z* 239 (M^+^), calculated *m*/*z* 239.07. Anal. Calcd. for C_13_H_9_N_3_O_2_: C, 65.27; H, 3.79; N, 17.56. Found: C, 65.16; H, 3.77; N, 17.57.

*1-(2-Methoxyphenyl)-5-nitro-1H-indazole* (**12b**). This reaction stopped at the hydrazone stage and did not give an indazole.

*1-(3-Methoxyphenyl)-5-nitro-1H-indazole* (**12c**): Yield: 180 mg (0.67 mmol, 67%) as a yellow solid, m.p. 132–133 °C (lit [36] m.p. 135 °C); IR: 1516, 1344 cm^−1^; ^1^H-NMR (DMSO-*d*_6_): δ 8.94 (s, 1H), 8.69 (s, 1H), 8.30 (d, *J* = 9.2 Hz, 1H), 8.01 (d, *J* = 9.3 Hz, 1H), 7.55 (t, *J* = 8.0 Hz, 1H), 7.37 (d, *J* = 8.0 Hz, 1H), 7.32 (s, 1H), 7.08 (d, *J* = 8.2 Hz, 1H), 3.87 (s, 3H); ^13^C-NMR (DMSO-*d*_6_): δ 160.6, 142.8, 140.5, 140.2, 130.8, 131.2, 124.8, 122.7, 119.8, 115.3, 114.1, 112.0, 109.0, 56.0; MS: *m*/*z* 269 (M^+^), calculated *m*/*z* 269.08. Anal. Calcd. for C_14_H_11_N_3_O_3_: C, 62.45; H, 4.12; N, 15.61. Found: C, 62.37; H, 4.06; N, 15.74.

*1-(4-Methoxyphenyl)-5-nitro-1H-indazole* (**12d**). Yield: 199 mg (0.74 mmol, 74%) as a light yellow solid, m.p. 181–182 °C; IR: 1516, 1342 cm^−1^; ^1^H-NMR (DMSO-*d*_6_): δ 8.92 (d, *J* = 2.2 Hz, 1H), 8.64 (s, 1H), 8.27 (d, *J* = 9.2, 2.2 Hz, 1H), 7.84 (d, *J* = 9.2 Hz, 1H), 7.68 (d, *J* = 8.7 Hz, 2H), 7.18 (d, *J* = 8.7 Hz, 2H), 3.86 (s, 3H); ^13^C-NMR (DMSO-*d*_6_): δ 159.2, 142.6, 141.6, 138.3, 132.1, 125.2, 124.3, 122.4, 119.8, 115.6, 111.6, 56.2; MS: *m*/*z* 269 (M^+^), calculated *m*/*z* 269.08. Anal. Calcd. for C_14_H_11_N_3_O_3_: C, 62.45; H, 4.12; N, 15.61. Found: C, 62.41; H, 4.07; N, 15.56.

*1-(4-Bromophenyl)-5-nitro-1H-indazole* (**12e**). Yield: 222 mg (0.70 mmol, 70%) as a tan solid, m.p. 169–170 °C; IR: 1511, 1348 cm^−1^; ^1^H-NMR (DMSO-*d*_6_): δ 8.95 (s, 1H), 8.71 (s, 1H), 8.32 (d, *J* = 9.3 Hz, 1H), 8.01 (d, *J* = 9.2 Hz, 1H), 7.84 (d, *J* = 8.4 Hz, 2H), 7.79 (d, *J* = 8.4 Hz, 2H); ^13^C-NMR (DMSO-*d*_6_): δ 142.9, 140.4, 139.2, 138.4, 133.2, 125.2, 125.0, 122.8, 120.7, 119.9, 111.9; MS: *m*/*z* 317 (M^+^), calculated *m*/*z* 316.98. Anal. Calcd. for C_13_H_8_BrN_3_O_2_: C, 49.08; H, 2.53; N, 13.21. Found: C, 48.97; H, 2.59; N, 13.25.

*1-(3-Chlorophenyl)-5-nitro-1H-indazole* (**12f**). Yield: 164 mg (0.60 mmol, 60%) as an off-white solid, m.p. 143–144 °C; IR: 1514, 1346 cm^−1^; ^1^H-NMR (DMSO-*d*_6_): δ 8.95 (s, 1H), 8.72 (s, 1H), 8.32 (d, *J* = 9.2 Hz, 1H), 8.03 (d, *J* = 9.2 Hz, 1H), 7.88 (s, 1H), 7.82 (d, *J* = 8.0 Hz, 1H), 7.67 (t, *J* = 8.1 Hz, 1H), 7.57 (d, *J* = 8.2 Hz, 1H); ^13^C-NMR (DMSO-*d*_6_): δ 142.9, 140.5, 140.3, 139.4. 134.6, 132.0, 128.0, 125.0, 123.0, 122.9, 121.7, 119.8, 112.0; MS: *m*/*z* 273 (M^+^), calculated *m*/*z* 273.03. Anal. Calcd. for C_13_H_8_ClN_3_O_2_: C, 57.05; H, 2.95; N, 15.35. Found: C, 56.96; H, 3.00; N, 15.29.

*1-(4-Chlorophenyl)-5-nitro-1H-indazole* (**12g**). Yield: 191 mg (0.70 mmol, 70%) as a light yellow solid, m.p. 179–180 °C; IR: 1509, 1343 cm^−1^; ^1^H-NMR (DMSO-*d*_6_): δ 8.95 (d, *J* = 2.2 Hz, 1H), 8.71 (s, 1H), 8.31 (dd, *J* = 9.3 , 2.2 Hz, 1H), 7.99 (d, *J* = 9.3 Hz, 1H), 7.85 (d, *J* = 8.6 Hz, 2H), 7.70 (d, *J* = 8.6 Hz, 2H); ^13^C-NMR (DMSO-*d*_6_): δ 142.9, 140.5, 139.2, 138.0, 132.4, 130.3, 124.9, 122.8, 119.9, 111.9 (one C not observed); MS: *m*/*z* 273 (M^+^), calculated *m*/*z* 273.03. Anal. Calcd. for C_13_H_8_ClN_3_O_2_: C, 57.05; H, 2.95; N, 15.35. Found: C, 57.12; H, 2.97; N, 15.33.

*1-(2,4-Dichlorophenyl)-5-nitro-1H-indazole* (**12h**). Yield: 215 mg (0.70 mmol, 70%) as a light pink solid, m.p. 101–102 °C. IR: 1517, 1344 cm^−1^; ^1^H-NMR (DMSO-*d*_6_): δ 8.96 (s, 1H), 8.72 (s, 1H), 8.29 (d, *J* = 9.2 Hz, 1H), 8.03 (s, 1H), 7.78 (d, *J* = 8.3 Hz, 1H), 7.73 (d, *J* = 8.3 Hz, 1H), 7.52 (d, *J* = 9.2 Hz, 1H); ^13^C-NMR (DMSO-*d*_6_): δ 143.0, 142.3, 139.2, 135.7, 135.0, 132.2, 131.6, 130.8, 129.3, 123.9, 122.7, 119.8, 111.7; MS: *m*/*z* 307 (M^+^), calculated *m*/*z* 306.99. Anal. Calcd. for C_13_H_7_Cl_2_N_3_O_2_: C, 50.68; H, 2.29; N, 13.64. Found: C, 50.67; H, 2.34; N, 13.53.

*1-(3-(Trifluoromethyl)phenyl)-5-nitro-1H-indazole* (**12i**). Yield: 190 mg (0.62 mmol, 62%) as a light yellow solid, m.p. 118–119 °C. IR: 1513, 1347 cm^−1^; ^1^H-NMR (DMSO-*d*_6_): δ 8.97 (s, 1H), 8.75 (s, 1H), 8.35 (d, *J* = 9.2 Hz, 1H), 8.18 (d, *J* = 7.2 Hz, 1H), 8.13 (s, 1H), 8.05 (d, *J* = 9.3 Hz, 1H), 7.94–7.85 (complex, 2H); ^13^C-NMR (DMSO-*d*_6_): δ 143.0, 140.6, 139.8, 139.6, 131.7, 131.0 (q, *J* = 32.7 Hz), 126.9, 125.2, 124.6 (q, *J* = 4.3 Hz), 124.1 (q, *J* = 272.6 Hz), 123.0, 119.8 (q, *J* = 4.9 Hz), 112.0 (one C not observed); MS: *m*/*z* 307 (M^+^), calculated *m*/*z* 307.06. Anal. Calcd. for C_14_H_8_F_3_N_3_O_2_: C, 54.73; H, 2.62; N, 13.68. Found: C, 54.66; H, 2.64; N, 13.57.

*1-(4-(Trifluoromethyl)phenyl)-5-nitro-1H-indazole* (**12j**). Yield: 209 mg (0.68 mmol, 68%) as a light yellow solid, m.p. 151–152 °C. IR: 1519, 1327 cm^−1^; ^1^H-NMR (DMSO-*d*_6_): δ 8.95 (s, 1H), 8.76 (s, 1H), 8.34 (d, *J* = 9.5 Hz, 1H), 8.11 (obscured d, *J* = 9.5 Hz, 1H), 8.08 (d, *J* = 8.5 Hz, 2H), 8.00 (d, *J* = 8.5 Hz, 2H); ^13^C-NMR (DMSO-*d*_6_): δ 143.1, 142.3, 140.5, 139.8, 127.9, (q, *J* = 32.3 Hz), 127.5 (q, *J* = 3.5 Hz), 125.4, 124.4 (q, *J* = 273.7 Hz), 123.3, 123.1, 119.9, 112.1; MS: *m*/*z* 307 (M^+^), calculated *m*/*z* 307.06. Anal. Calcd. for C_14_H_8_F_3_N_3_O_2_: C, 54.73; H, 2.62; N, 13.68. Found: C, 54.68; H, 2.61; N, 13.64.

*1-(4-Cyanophenyl)-5-nitro-1H-indazole* (**12k**). Yield: 158 mg (0.60 mmol, 60%) as a light yellow solid, m.p. 250–251 °C. IR: 2227, 1512, 1344 cm^−1^; ^1^H-NMR (DMSO-*d*_6_): δ 8.97 (d, *J* = 2.2 Hz, 1H), 8.78 (s, 1H), 8.36 (dd, *J* = 9.3, 2.2 Hz, 1H), 8.15 (d, *J* = 9.3 Hz, 1H), 8.12 (d, *J* = 8.8 Hz, 2H), 8.07 (d, *J* = 8.8 Hz, 2H); ^13^C-NMR (DMSO-*d*_6_): δ 142.1, 141.6, 139.4, 139.1, 133.6, 124.5, 122.1, 120.0, 118.9, 117.7, 111.3, 109.0; MS: *m*/*z* 264 (M^+^), calculated *m*/*z* 264.06. Anal. Calcd. for C_14_H_8_N_4_O_2_: C, 63.64; H, 3.05; N, 21.20. Found: C, 63.55; H, 3.09; N, 21.08.

*4-(5-Nitro-1H-indazol-1-yl)benzenesulfonamide* (**12l**). Yield: 159 mg (0.50 mmol, 50%), m.p. 237–238 °C; IR: 3329, 3241, 1512, 1339 cm^−1^; ^1^H-NMR (DMSO-*d*_6_): δ 8.97 (d, *J* = 2.2 Hz, 1H), 8.76 (s, 1H), 8.35 (dd, *J* = 9.3, 2.2 Hz, 1H), 8.13 (d, *J* = 9.3 Hz, 1H), 8.06 (s, 4H), 7.53 (br s, 2H); ^13^C-NMR (DMSO-*d*_6_): δ 143.1, 141.60, 141.55, 140.5, 139.8, 128.0, 125.3,123.1, 122.2, 119.9, 112.2; MS: *m*/*z* 318 (M^+^), calculated *m*/*z* 318.04. Anal. Calcd. for C_13_H_10_N_4_O_4_S: C, 49.05; H, 3.17; N, 17.60. Found: C, 49.11; H, 3.16; N, 17.66.

*4-(5-Nitro-1H-indol-1yl)benzoic acid* (**12m**). This product was formed only using the two-step procedure. Yield: 142 mg (0.50 mmol, 50%) as a brown product, m.p. 158–159 °C; IR: 3395–2372, 1694, 1518, 1345 cm^−1^; ^1^H-NMR (DMSO-*d*_6_): δ 13.3 (br s, 1H), 8.97 (d, *J* = 2.2 Hz, 1H), 8.76 (s, 1H), 8.35 (dd, *J* = 9.3, 2.2 Hz , 1H), 8.18 (d, *J* = 8.4 Hz, 2H), 8.13 (d, *J* = 9.3 Hz, 1H), 7.98 (d, *J* = 8.4 Hz, 2H); ^13^C-NMR (DMSO-*d*_6_): δ 167.0, 143.0, 142.5, 140.4, 139.7, 131.5, 129.8, 125.3, 123.0, 122.5, 119.9, 112.2; MS: *m*/*z* 283 (M^+^), calculated *m*/*z* 283.06. Anal. Calcd. for C_14_H_9_N_3_O_4_: C, 59.37; H, 3.20; N, 14.84. Found: C, 59.32; H, 3.23; N, 14.75.

### 3.5. Representative Procedure for the General Indazole Synthesis

To a stirred solution of the carbonyl compound (**13**, **14** or **15**, 1.0 mmol) in DMPU (5 mL) were added powdered 4 Å molecular sieves (30 wt% relative to the carbonyl substrate) and ArNHNH_2_·HCl (1.5 mmol). The mixture was heated at 90 °C (oil bath) for 1.5 h at which time CuI (0.2 mmol) and K_2_CO_3_ (2.5 mmol) were added and heating was continued at this temperature for 16 h. The crude reaction mixture was cooled to 23 °C and filtered through a Celite^®^ pad (Fisher Scientific, Pittsburgh, PA, USA). The pad was rinsed with ether (2 × 20 mL) and the combined filtrate was washed with water (25 mL), saturated NaCl (25 mL), dried (MgSO_4_), filtered, and concentrated under vacuum. The products were purified by silica gel chromatography using increasing concentrations of EtOAc in hexanes. Yields as well as physical and spectral data are given below.

*3-Methyl-1-phenyl-1H-indazole* (**16a**). Yield: 169 mg (0.81 mmol, 81%) as tan solid, m.p. 73–74 °C (lit [18] m.p. 73–74 °C); IR: 1597, 1505 cm^−1^; ^1^H-NMR (CDCl_3_): δ 7.75–7.96 (complex, 4H), 7.52 (t, *J* = 7.3 Hz, 2H), 7.42 (t, *J* = 7.5 Hz, 1H), 7.32 (t, *J* = 7.3 Hz, 1H), 7.21 (t, *J* = 7.5 Hz, 1H), 2.65 (s, 3H); ^13^C-NMR (CDCl_3_): δ 144.0, 140.3, 139.5, 129.4, 127.1, 126.1, 124.9, 122.4, 120.8, 120.6, 110.3, 12.0; MS: *m*/*z* 208 (M^+^), calculated *m*/*z* 208.10. Anal. Calcd. for C_14_H_12_N_2_: C, 80.74; H, 5.81; N, 13.45. Found: C, 81.01; H, 6.08; N, 13.23.

*1-(4-Methoxyphenyl)-3-methyl-1H-indazole* (**16d**). Yield: 207 mg (0.87 mmol, 87%) as a yellow oil; IR: 2845, 1517 cm^−1^; ^1^H-NMR (CDCl_3_): δ 7.72 (d, *J* = 8.1 Hz, 1H), 7.60 (d, *J* = 7.7 Hz, 1H), 7.59 (d, *J* = 8.9 Hz, 2H), 7.39 (t, *J* = 7.4 Hz, 1H), 7.18 (t, *J* = 7.5 Hz, 1H), 7.04 (d, *J* = 8.9 Hz, 2H), 3.87 (s, 3H), 2.65 (s, 3H); ^13^C-NMR (CDCl_3_): δ 158.1, 143.8, 139.7, 133.5, 126.9, 124.4, 124.3, 120.50, 120.47, 114.6, 110.1, 56.6, 11.9; MS: *m*/*z* 238 (M^+^), calculated *m*/*z* 238.11. Anal. Calcd. for C_15_H_14_N_2_O: C, 75.61; H, 5.92; N, 11.76. Found: C, 75.35; H, 6.18; N, 11.25.

*4-(3-Methyl-1H-indazol-1-yl)benzonitrile* (**16k**). Yield: 198 mg (0.85 mmol, 85%) as tan solid, m.p. 124–126 °C; IR: 2224, 1604, 1517 cm^−1^; ^1^H-NMR (CDCl_3_): δ 7.91 (d, *J* = 8.6 Hz, 2H), 7.80 (d, *J* = 8.6 Hz, 2H), 7.78 (d, *J* = 8.4 Hz, 1H), 7.75 (d, *J* = 7.9 Hz, 1H), 7.50 (t, *J* = 8.3 Hz, 1H), 7.28 (t, *J* = 7.4 Hz, 1H), 2.65 (s, 3H); ^13^C-NMR (CDCl_3_): δ 146.1, 143.9, 139.1, 133.5, 128.0, 125.9, 121.9, 121.3, 121.1, 118.7, 110.5, 108.5, 12.0; MS: *m*/*z* 233 (M^+^), calculated *m*/*z* 233.10. Anal. Calcd. for C_15_H_11_N_3_: C, 77.23; H, 4.75; N, 18.01. Found: C, 77.35; H, 4.31; N, 18.32.

*1-Phenyl-1H-indazole* (**17a**). Yield: 149 mg (0.77 mmol, 77%) as off-white solid, m.p. 77–78 °C (lit [18] m.p. 76–78 °C); IR: 1595, 1500 cm^−1^; ^1^H-NMR (CDCl_3_): δ 8.21 (s, 1H), 7.81 (d, *J* = 8.1 Hz, 1H), 7.77 (d, *J* = 8.4 Hz, 1H), 7.75 (d, *J* = 8.7 Hz, 2H), 7.54 (t, *J* = 8.4 Hz, 2H), 7.43 (t, *J* = 8.3 Hz, 1H), 7.37 (t, *J* = 8.3 Hz, 1H), 7.23 (t, *J* = 8.1 Hz, 1H); ^13^C-NMR (CDCl_3_): δ 140.2, 138.8, 135.4, 129.5, 127.1, 126.6, 125.3, 122.8, 121.5, 121.3, 110.4; MS: *m*/*z* 194 (M^+^), calculated *m*/*z* 194.08. Anal. Calcd. for C_13_H_10_N_2_: C, 80.39; H, 5.19; N, 14.42. Found: C, 80.15; H, 5.31; N, 14.69.

*1-(4-Methoxyphenyl)-1H-indazole* (**17d**). Yield: 177 mg (0.79 mmol, 79%) as a yellow oil; IR: 2836, 1515 cm^−1^; ^1^H-NMR (CDCl_3_): δ 8.17 (s, 1H), 7.79 (d, *J* = 8.1 Hz, 1H), 7.65 (d, *J* = 8.5 Hz, 1H), 7.62 (d, *J* = 8.9 Hz, 2H), 7.41 (t, *J* = 8.3 Hz, 1H), 7.21 (t, *J* = 8.0 Hz, 1H), 7.06 (d, *J* = 8.9 Hz, 2H), 3.88 (s, 3H); ^13^C-NMR (CDCl_3_): δ 158.4, 139.0, 134.8, 133.4, 126.9, 124.9, 124.5, 121.23, 121.21, 114.6, 110.2, 55.6; MS: *m*/*z* 224 (M^+^), calculated *m*/*z* 224.09. Anal. Calcd. for C_14_H_12_N_2_O: C, 74.98; H, 5.39; N, 12.49. Found: C, 74.63; H, 5.52; N, 12.63.

*4-(1H-Indazol-1-yl)benzonitrile* (**17k**). Yield: 164 mg (0.75 mmol, 75%) as white solid, m.p. 104–106 °C (lit m.p. [18] 103–106 °C); IR: 2225, 1604, 1510 cm^−1^; ^1^H-NMR (CDCl_3_): δ 8.26 (s, 1H), 7.94 (d, *J* = 8.6 Hz, 2H), 7.84 (d, *J* = 8.6 Hz, 2H), 7.86–7.81 (complex, 2H), 7.51 (t, *J* = 8.4 Hz, 1H), 7.31 (t, *J* = 8.0 Hz, 1H); ^13^C-NMR (CDCl_3_): δ 143.8, 138.5, 137.3, 133.6, 128.1, 126.1, 122.5, 121.9, 121.8, 118.5, 110.4, 109.3; MS: *m*/*z* 219 (M^+^), calculated *m*/*z* 219.08. Anal. Calcd. for C_14_H_9_N_3_: C, 76.70; H, 4.14; N, 19.17. Found: C, 76.85; H, 4.31; N, 19.32.

*1-Phenyl-1H-pyrazolo[3,4-b]pyridine* (**18a**). Yield: 133 mg (0.68 mmol, 68%) as a white solid, m.p. 52–54 °C (lit [10] 53–55 °C); IR: 1595, 1499 cm^−1^; ^1^H-NMR (CDCl_3_): δ 8.65 (dd, *J* = 4.5, 1.7 Hz, 1H), 8.28 (dd, *J* = 7.7, 1.2 Hz, 2H), 8.21 (s, 1H), 8.14 (dd, *J* = 8.0, 1.7 Hz, 1H), 7.54 (t, *J* = 7.7 Hz, 1H), 7.33 (t, *J* = 7.5 Hz, 1H), 7.22 (dd, *J* = 8.0, 4.5 Hz, 1H); ^13^C-NMR (CDCl_3_): δ 150.1, 149.2, 139.5, 133.8, 130.2, 129.1, 126.1, 121.4, 117.6, 117.2; MS: *m*/*z* 195 (M^+^), calculated *m*/*z* 195.08. Anal. Calcd. for C_12_H_9_N_3_: C, 73.83; H, 4.65; N, 21.52. Found: C, 73.95; H, 4.87; N, 21.78.

*1-(4-Methoxyphenyl)-1H-pyrazolo[3,4-b]pyridine* (**18d**). Yield: 140 mg (0.62 mmol, 62%) as a purple solid, m.p. 204–205 °C; IR: 2833, 1513 cm^−1^; ^1^H-NMR (CDCl_3_): δ 9.00 (dd, *J* = 8.0, 1.7 Hz, 1H), 8.70 (dd, *J* = 4.5, 1.7 Hz, 1H), 8.28 (d, *J* = 9.0 Hz, 2H), 7.35 (dd, *J* = 8.0, 4.5 Hz, 1H), 7.26 (s, 1H), 7.14 (d, *J* = 9.0 Hz, 2H), 3.91 (s, 3H); ^13^C-NMR (CDCl_3_): δ 158.0, 150.6, 149.6, 138.7, 132.9, 132.6, 123.0, 118.3, 115.3, 114.4, 55.6; MS: *m*/*z* 225 (M^+^), calculated *m*/*z* 225.09. Anal. Calcd. for C_13_H_11_N_3_O: C, 69.32; H, 4.92; N, 18.66. Found: C, 69.68; H, 5.11; N, 19.82.

*4-(1H-Pyrazolo[3,4-b]pyridine-1-yl)benzonitrile* (**18k**). Yield: 172 mg (0.78 mmol, 78%) as a white solid, m.p. 125–127 °C; IR: 2222, 1603, 1450 cm^−1^; ^1^H-NMR (CDCl_3_): δ 8.68 (dd, *J* = 4.5, 1.6 Hz, 1H), 8.66 (d, *J* = 8.8 Hz, 2H), 8.25 (s, 1H), 8.17 (dd. *J* = 8.0, 1.6 Hz, 1H), 7.82 (d, *J* = 8.8 Hz, 2H), 7.30 (dd, *J* = 8.0, 4.5 Hz, 1H); ^13^C-NMR (CDCl_3_): δ 150.7, 149.4, 143.1, 135.4, 133.2, 130.6, 120.3, 11.89, 118.4, 117.9, 108.7; MS: *m*/*z* 220 (M^+^), calculated *m*/*z* 220.07. Anal. Calcd. for C_13_H_8_N_4_: C, 70.90; H, 3.66; N, 25.44. Found: C, 71.23; H, 3.82; N, 25.67.

## 4. Conclusions

We have developed and optimized one and two-pot syntheses of 1-aryl-5-nitro-1*H*-indazoles from 2′-fluoro-5′-nitroacetophenone (**5**) and 2-fluoro-5-nitrobenzaldehyde (**6**). The one-pot conversion of **5** to **11** (a domino reaction) and **6** to **12** (a consecutive reaction) proceeded in yields (70–96%) that surpassed those of the two-pot sequence. We have also developed a general one-pot approach to 1-aryl-1*H*-indazoles that is not limited by the substitution patterns normally required for the S_N_Ar ring closure. Treatment of 2′-bromoacetophenone (**13**), 2-bromobenzaldehyde (**14**) and 2-chloronicotinaldehyde (**15**) with ArNHNH_2_·HCl and 30 wt % of powdered 4 Å molecular sieves in DMPU at 90 °C, followed by addition of CuI and K_2_CO_3_ afforded 1*H*-indazoles bearing phenyl as well as electron-rich and electron-poor aromatic groups at N1 in 62–87% yields. The efficiency of the one-pot process provides access to a selection of 1-aryl-1*H*-indazoles in a single laboratory operation—one set-up and one work-up—without isolation or purification of intermediates. The methodology described herein has streamlined the synthesis of 1-aryl-1*H*-indazoles, improved the yields, and minimized the waste generated in preparing these systems. 

## Figures and Tables

**Table 1:**
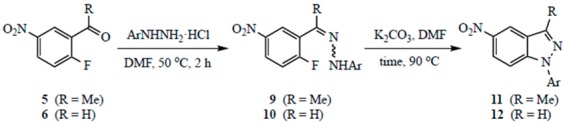
Yields for the two-step synthesis of 1-aryl-5-nitro-1*H*-indazoles.

Substrate	Ar	Time (h) ^a^	Pdt	% Yield ^b^
**5**	**a** Ph-	2	**11a**	95
**5**	**b** 2-MeOPh-	2	**11b**	82
**5**	**c** 3-MeOPh-	2	**11c**	81
**5**	**d** 4-MeOPh-	2	**11d**	94
**5**	**e** 4-BrPh-	2	**11e**	95
**5**	**f** 3-ClPh-	2	**11f**	87
**5**	**g** 4-ClPh-	2	**11g**	93
**5**	**h** 2,4-Cl_2_Ph	36	**11h**	80
**5**	**I** 3-CF_3_Ph-	2	**11i**	88
**5**	**j** 4-CF_3_Ph-	2	**11j**	70
**5**	**k** 4-CNPh-	2	**11k**	80
**5**	**l** 4-H_2_NSO_2_Ph-	10	**11l**	70
**5**	**m** 4-HO_2_CPh-	24	**11m**	75
**6**	**a** Ph-	2	**12a**	72
**6**	**b** 2-MeOPh-	2	**12b**	0 ^c^
**6**	**c** 3-MeOPh-	2	**12c**	67
**6**	**d** 4-MeOPh-	2	**12d**	70
**6**	**e** 4-BrPh-	2	**12e**	70
**6**	**f** 3-ClPh-	2	**12f**	60
**6**	**g** 4-ClPh-	2	**12g**	70
**6**	**h** 2,4-Cl2Ph-	2	**12h**	60
**6**	**I** 3-CF_3_Ph-	2	**12i**	62
**6**	**j** 4-CF_3_Ph-	2	**12j**	68
**6**	**k** 4-CNPh-	3	**12k**	60
**6**	**l** 4-H_2_NSO_2_Ph-	2	**12l**	50
**6**	**m** 4-HO_2_CPh-	2	**12m**	50

^a^ All hydrazones were generated in two hours. Times given are for the final cyclization. ^b^ Isolated yield of 1*H*-indazole for the two-step sequence. ^c^ Only the hydrazone was isolated.

**Table 2:**
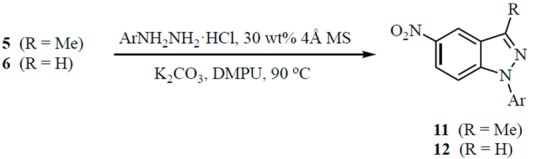
Yields for the modified one-pot synthesis of 1-aryl-5-nitro-1*H*-indazoles.

Substrate	Ar	Time (h) ^a^	Pdt	% Yield ^b^
**5**	**a** Ph-	1.5	**11a**	96
**5**	**d** 4-MeOPh-	1.5	**11d**	95
**5**	**g** 4-ClPh-	1.5	**11g**	94
**5**	**h** 2,4-Cl2Ph-	72	**11h**	85
**5**	**l** H_2_NSO_2_Ph-	16	**11m**	85
**5**	**m** HO_2_CPh-	45	**11n**	73
**6**	**a** Ph-	3.5	**12a**	73
**6**	**d** 4-MeOPh-	3.5	**12d**	74
**6**	**g** 4-ClPh-	3.5	**12g**	71
**6**	**k** 4-NCPh-	6.5	**12k**	63
**6**	**l** H_2_NSO_2_Ph-	8.5	**12l**	0
**6**	**m** HO_2_CPh-	8.5	**12m**	0

^a^ For **5**, time is the total reaction time. For **6**, the hydrazone was allowed to form for 1.5 h at 90 °C before base was added; time is for the final cyclization. ^b^ Isolated yield. DMPU: 1,3-dimethyl-3,4,5,6-tetrahydro-2(1*H*)-pyrimidinone.

**Table 3:**
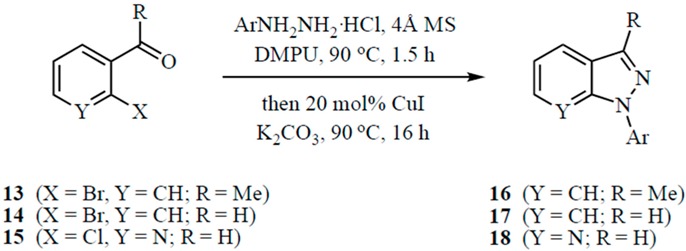
Yields for the general one-pot synthesis of 1-aryl-1*H*-indazoles.

Substrate	Ar	Product	% Yield ^a^
**13**	**a** Ph-	**16a**	81
**13**	**d** 4-MeOPh-	**16d**	83
**13**	**k** 4-CNPh-	**16l**	87
**14**	**a** Ph-	**17a**	77
**14**	**d** 4-MeOPh-	**17d**	72
**14**	**k** 4-CNPh-	**17l**	79
**15**	**a** Ph-	**18a**	68
**15**	**d** 4-MeOPh-	**18d**	62
**15**	**k** 4-CNPh-	**18l**	78

^a^ Isolated yield.

## Supplementary Materials

Electronic Supplementary Information (ESI) are available online: Tables summarizing optimization experiments, a listing of characterization data for the intermediate arylhydrazones, and ^1^H- and ^13^C-NMR spectra for all compounds, are available online.
