# Supplementary material for: Syntheses of 1-Aryl-5-nitro-1H-indazoles and a General One-Pot Route to 1-Aryl-1H-indazoles
Source: Molecules. 2018 Mar 16;23(3):674. doi: 10.3390/molecules23030674 (PMC6017161; doi:10.3390/molecules23030674)

Syntheses of 1-Aryl-5-nitro-1*H*-indazoles and a General Sequential Route to 1-Aryl-1*H*-indazoles

Joel K. Annor-Gyamfi, Krishna Kumar Gnanasekaran and Richard A. Bunce*

Department of Chemistry, Oklahoma State University, Stillwater, OK 74078-3071, USA

Supplementary Information

page

Table S-1. Optimization of conditions for the two-step procedure to produce 1-aryl-5-nitro-1*H*-indazoles. 4

Table S-2. Optimization of conditions for the one-pot procedure to produce 1-aryl-5-nitro-1*H*-indazoles. 5

Table S-3: Optimization results for the general 1*H*-indazole synthesis using 2-bromobenzaldehyde. 6

Characterization data for hydrazones derived from 2'-fluoro-5'-nitroacetophenone 7–9

Phenylhydrazone **9a**
(2-Methoxyphenyl)hydrazone **9b**
(3-Methoxyphenyl)hydrazone **9c**
(4-Methoxyphenyl)hydrazone **9d**
(4-Bromophenyl)hydrazone **9e**
(3-Chlorophenyl)hydrazone **9f**
(4-Chlorophenyl)hydrazone **9g**
(2,4-Dichlorophenyl)hydrazone **9h**
(3-(Trifluoromethyl)phenyl)hydrazone **9i**
(4-(Trifluoromethyl)phenyl)hydrazone **9j**
(4-Cyanophenyl)hydrazone **9k**
(4-Sulfonamidophenyl)hydrazone **9l**
(4-Carboxyphenyl)hydrazone **9m**

Characterization data for hydrazones derived from 2-fluoro-5-nitrobenzaldehyde 9–12

Phenylhydrazone **10a**
(2-Methoxyphenyl)hydrazone **10b**
(3-Methoxyphenyl)hydrazone **10c**
(4-Methoxyphenyl)hydrazone **10d**
(4-Bromophenyl)hydrazone **10e**
(3-Chlorophenyl)hydrazone **10f**
(4-Chlorophenyl)hydrazone **10g**
(2,4-Dichlorophenyl)hydrazone **10h**
(3-(Trifluoromethyl)phenyl)hydrazone **10i**
(4-(Trifluoromethyl)phenyl)hydrazone **10j**
(4-Cyanophenyl)hydrazone **10k**
(4-Sulfonamidophenyl)hydrazone **10l**
(4-Carboxyphenyl)hydrazone **10m**

^{1}H and ^{13}C NMR spectra for hydrazones derived from:	page
Phenylhydrazone **9a**	13
(2-Methoxyphenyl)hydrazone **9b**	14
(3-Methoxyphenyl)hydrazone **9c**	15
(4-Methoxyphenyl)hydrazone **9d**	16
(4-Bromophenyl)hydrazone **9e**	17
(3-Chlorophenyl)hydrazone **9f**	18
(4-Chlorophenyl)hydrazone **9g**	19
(2,4-Dichlorophenyl)hydrazone **9h**	20
(3-(Trifluoromethyl)phenyl)hydrazone **9i**	21
(4-(Trifluoromethyl)phenyl)hydrazone **9j**	22
(4-Cyanophenyl)hydrazone **9k**	23
(4-Sulfonamidophenyl)hydrazone **9l**	24
(4-Carboxyphenyl)hydrazone **9m**	25
Phenylhydrazone **10a**	26
(2-Methoxyphenyl)hydrazone **10b**	27
(3-Methoxyphenyl)hydrazone **10c**	28
(4-Methoxyphenyl)hydrazone **10d**	29
(4-Bromophenyl)hydrazone **10e**	30
(3-Chlorophenyl)hydrazone **10f**	31
(4-Chlorophenyl)hydrazone **10g**	32
(2,4-Dichlorophenyl)hydrazone **10h**	33
(3-(Trifluoromethyl)phenyl)hydrazone **10i**	34
(4-(Trifluoromethyl)phenyl)hydrazone **10j**	35
(4-Cyanophenyl)hydrazone **10k**	36
(4-Sulfonamidophenyl)hydrazone **10l**	37
(4-Carboxyphenyl)hydrazone **10m**	38
3-Methyl-5-nitro-1*H*-indazole (**7**)	39
5-Nitro-1*H*-indazole (**8**)	40
3-Methyl-1-phenyl-5-nitro-1*H*-indazole (**11a**)	41
1-(2-Methoxyphenyl)-3-methyl-5-nitro-1*H*-indazole (**11b**)	42
1-(3-Methoxyphenyl)-3-methyl-5-nitro-1*H*-indazole (**11c**)	43
1-(4-Methoxyphenyl)-3-methyl-5-nitro-1*H*-indazole (**11d**)	44
1-(4-Bromophenyl)-3-methyl-5-nitro-1*H*-indazole (**11e**)	45
1-(3-Chlorophenyl)-3-methyl-5-nitro-1*H*-indazole (**11f**)	46
1-(4-Chlorophenyl)-3-methyl-5-nitro-1*H*-indazole (**11g**)	47
1-(2,4-Dichlorophenyl)-3-methyl-5-nitro-1*H*-indazole (**11h**)	48
3-Methyl-5-nitro-1-(3-(trifluoromethyl)phenyl)-1*H*-indazole (**11i**)	49
3-Methyl-5-nitro-1-(4-(trifluoromethyl)phenyl)-1*H*-indazole (**11j**)	50
1-(4-Cyanophenyl)-3-methyl-5-nitro-1*H*-indazole (**11k**)	51
4-(3-Methyl-5-nitro-1*H*-indazol-1-yl)benzenesulfonamide (**11l**)	52
4-(3-Methyl-5-nitro-1*H*-indazol-1-yl)benzoic acid (**11m**)	53

^1H and ^{13}C NMR spectra for: page

1-Phenyl-5-nitro-1*H*-indazole (**12a**) 54
1-(3-Methoxyphenyl)-5-nitro-1*H*-indazole (**12c**) 55
1-(4-Methoxyphenyl)- 5-nitro-1*H*-indazole (**12d**) 56
1-(4-Bromophenyl)-5-nitro-1*H*-indazole (**12e**) 57
1-(3-Chlorophenyl)-5-nitro-1*H*-indazole (**12f**) 58
1-(4-Chlorophenyl)-5-nitro-1*H*-indazole (**12g**) 59
1-(2,4-Dichlorophenyl)-5-nitro-1*H*-indazole (**12h**) 60
5-Nitro-1-(3-(trifluoromethyl)phenyl)-1*H*-indazole (**12i**) 61
5-Nitro-1-(4-(trifluoromethyl)phenyl)-1*H*-indazole (**12j**) 62
1-(4-Cyanophenyl)-5-nitro-1*H*-indazole (**12k**) 63
4-(5-Nitro-1*H*-indazol-1-yl)benzenesulfonamide (**12l**) 64
4-(5-Nitro-1*H*-indazol-1-yl)benzoic acid (**12m**) 65

3-Methyl-1-phenyl-1*H*-indazole (**16a**) 66
1-(4-Methoxyphenyl)-3-methyl-1*H*-indazole (**16d**) 67
4-(3-Methyl-1*H*-indazol-1-yl)benzonitrile (**16k**) 68

1-Phenyl-1*H*-indazole (**17a**) 69
1-(4-Methoxyphenyl)-1*H*-indazole (**17d**) 70
4-(1*H*-Indazol-1-yl)benzonitrile (**17k**) 71

1-Phenyl-1*H*-pyrazolo[3,4-*b*]pyridine (**18a**) 72
1-(4-Methoxyphenyl)-1*H*-pyrazolo[3,4-*b*]pyridine (**18d**) 73
4-(1*H*-Pyrazolo[3,4-*b*]pyridin-1-yl)benzonitrile (**18k**) 74

Table S-1. Optimization of conditions for the two-step procedure to produce 1-aryl-5-nitro-1*H*-indazoles.

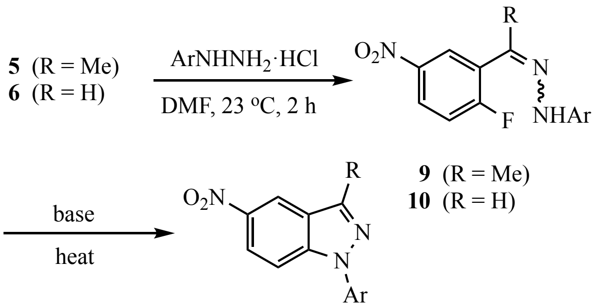

11 (R = Me)
12 (R = H)

Equiv $ArNHNH_2 \cdot HCl$	R	Yield Hydrazone
1.5	Me	60%
	H	85%
2.0	Me	80%
	H	95%*
2.5	Me	85%
	H	95%
3.0	Me	96%*
	H	92%

Base	Equiv Base	T (°C)	Yield Indazole
K_2CO_3	2.0	90	50% for Me<br>33% for H
K_2CO_3	2.5	90	85% for Me<br>65% for H
Et_3N	3.0	90	30% for Me<br><5% for H
DBU	3.0	90	65% for Me<br>25% for H
Na_2CO_3	3.0	90	52% for Me<br>10% for H
K_2CO_3	3.0	50	50% for Me<br>20% for H
K_2CO_3	3.0	90	95% for Me*<br>72% for H*

* indicates optimum conditions

The optimum conditions for hydrazone formation involved the use of 3.0 mmol of $ArNHNH_2 \cdot HCl$ with the ketone substrate, 2.0 mmol with the aldehyde substrate. Ring closure was best effected through the use of 3.0 mmol of K_2CO_3 at 90 °C.

Table S-2. Optimization of conditions for the one-pot procedure to produce 1-aryl-5-nitro-1*H*-indazoles.

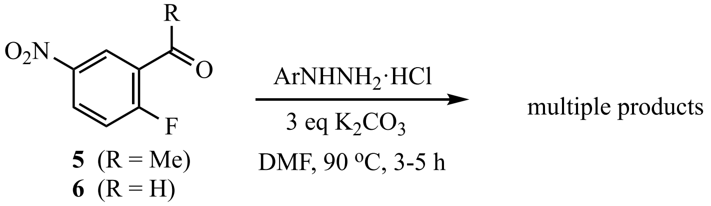

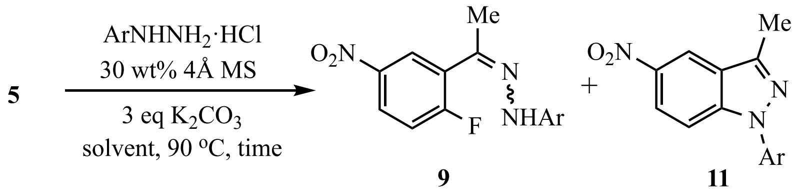

Solvent	Time (h)	Products Formed
DMF	3	50% hydrazone<br>50% indazole
Dioxane	3	No hydrazone or indazole formed
MeCN	3	No hydrazone or indazole formed
DMPU	1.5	Indazole

The one-step procedure to make 5-nitro-1-aryl-1*H*-indazoles was successful for the acetophenone substrate **1**. Benzaldehyde **2** gave higher yields when the reagents were added sequentially to first produce the hydrazone, followed by ring closure with base. The optimum conditions used 30 wt% of 4Å molecular sieves (to scavenge water) and DMPU as the solvent at 90 °C.

Table S-3: Optimization results for the general indazole synthesis using 2-bromobenzaldehyde.

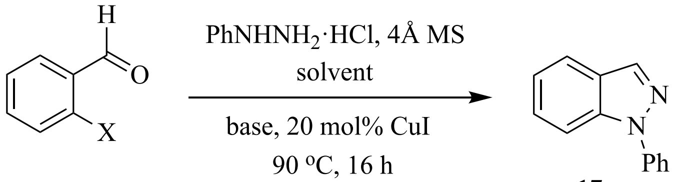

14 **17a**

Expt No	Equiv $PhNHNH_2 \cdot HCl$	Base	Equiv Base	Solvent	% Yield
1	3.0	K_2CO_3	3.5	DMF	< 5
2	1.5	K_2CO_3	2.5	DMF	27
3	1.5	K_2CO_3	2.5	DMF	48[a]
4	1.5	K_2CO_3	2.5	DMPU	77[b]
5	1.5	K_2CO_3	2.5	1,4-dioxane	35
6	1.5	Na_2CO_3	2.5	DMPU	22
7	1.5	DBU	2.5	DMPU	36
8	1.5	TMG[c]	2.5	DMPU	51

[a]All reagents were added and then gradually heated to 90-100 ^oC; [b] Substrate **14**, $PhNHNH_2 \cdot HCl$ and 30 wt% of powdered 4Å molecular sieves were added and immersed into a pre-heated oil bath at 90 ^oC, stirred for 1.5 h, and then 20 mol% CuI and base were added. [c]Tetramethylguanidine.

Optimum conditions for the general synthesis of 1-aryl-1*H*-indazoles involved mixing 1.5 equiv of the $ArNHNH_2 \cdot HCl$, 2.5 equiv of K_2CO_3 and 30 wt% of powdered 4Å molecular sieves in 5 mL of DMPU and heating at 90 ^oC (pre-heated oil bath). After 1.5 h, 20 mol% of CuI and 2.5 equiv of K_2CO_3 were added and heating was resumed at 90 ^oC for *ca* 16 h. Filtration, aqueous work-up and silica gel chromatography afforded the final product in pure form.

Characterization of arylhydrazones from 2'-fluoro-5'-nitroacetophenone.

Phenylhydrazone (**9a**): Yield: 259 mg (0.95 mmol, 95%)as an orange solid, mp 136-138 °C. IR (nujol): 3297, 1531, 1347 cm^{-1}: ^{1}H NMR (400 MHz, DMSO-d_6): δ 9.61 (s, 1H), 8.46 (dd, *J* = 6.7, 3.0 Hz, 1H), 8.22 (dt, *J* = 9.0, 3.6 Hz, 1H), 7.54 (t, *J* = 9.9 Hz, 1H), 7.29-7.20 (complex, 4H), 6.81 (m, 1H), 2.31 (s, 3H); ^{13}C NMR (100 MHz, DMSO-d_6): δ 162.4 (d, *J* = 256.4 Hz), 144.7, 143.4, 135.1, 128.6, 128.5 (d, *J* = 13.3 Hz), 128.4, 124.0 (d, *J* = 7.9 Hz), 119.1, 117.5 (d, *J* = 25.7 Hz), 112.4, 15.1.

(2-Methoxyphenyl)hydrazone (**9b**): Yield: 248 mg (0.82 mmol, 82%) as a brown solid, mp 120-121 °C. IR (nujol): 3299, 1531, 1338 cm^{-1}: ^{1}H NMR (400 MHz, DMSO-d_6): δ 8.46 (dd, *J* = 6.6, 3.0 Hz, 1H), 8.33 (s, 1H), 8.25 (dt, *J* = 9.0, 3.7 Hz, 1H), 7.37 (d, *J* = 7.7 Hz), 7.01 (d, *J* = 7.9 Hz, 1H), 6.93 (s, *J* = 7.5 Hz, 1H), 6.85 (t, *J* = 7.6 Hz, 1H), 3.88 (s, 3H), 2.33 (s, 3H); ^{13}C NMR (100 MHz, DMSO-d_6): δ 163.4 (d, *J* = 257.2 Hz), 146.2, 144.5, 138.5, 134.2, 129.3 (d, *J* = 13.8 Hz), 125.4 (d, *J* = 10.8 Hz), 125.1 (d, *J* = 5.8 Hz), 121.6, 120.6, 118.5 (d, *J* = 25.6 Hz), 112.9, 111.3, 56.1, 15.4.

(3-Methoxyphenyl)hydrazone (**9c**): Yield: 251 mg (0.83 mmol, 83%) as an orange solid, mp 120-121 °C. IR (nujol): 3295, 1528, 1341 cm^{-1}: ^{1}H NMR (400 MHz, DMSO-d_6): δ 9.60 (s, 1H), 8.46 (dd, *J* = 6.2, 3.0 Hz, 1H), 8.22 (dt, *J* = 9.3, 3.7 Hz, 1H), 7.53 (t, *J* = 9.8 Hz, 1H), 7.13 (d, *J* = 8.3 Hz, 1H), 6.82 (m, 2H), 6.40 (d, *J* = 8.0 Hz, 1H), 3.73 (s, 3H), 2.30 (s, 3H); ^{13}C NMR (100 MHz, DMSO-d_6): δ 163.4 (d, *J* = 257.7 Hz), 160.6, 147.1, 144.5, 136.2, 130.3, 129.5 (d, *J* = 13.3 Hz), 125.1, 125.0 (d, *J* = 4.4 Hz), 118.5 (d, *J* = 25.9 Hz), 106.2, 105.6, 99.3, 55.3, 16.1.

(4-Methoxyphenyl)hydrazone (**9d**): Yield: 273 mg (0.90 mmol, 90%) as a dark red solid, mp 112-114 °C. IR (nujol): 3302, 1531, 1350 cm^{-1}: ^{1}H NMR (400 MHz, DMSO-d_6): δ 9.42 (s, 1H), 8.44 (dd, *J* = 6.7, 3.0 Hz, 1H), 8.19 (dt, *J* = 9.0, 3.6 Hz, 1H), 7.52 (dd, *J* = 10.8, 9.0 Hz, 1H), 7.17 (d, *J* = 9.0 Hz, 2H), 6.87 (d, *J* = 9.0 Hz, 2H), 3.85 (s, 3H), 2.28 (s, 3H); ^{13}C NMR (100 MHz, DMSO-d_6): δ 163.6 (d, *J* = 257.0 Hz), 153.7, 144.5, 142.0, 141.3, 139.8, 129.7 (d, *J* = 13.3 Hz), 124.9 (d, *J* = 6.6 Hz), 118.4 (d, *J* = 25.8 Hz), 115.3, 114.9, 55.7, 16.0.

(4-Bromophenyl)hydrazone (**9e**): Yield: 333 mg (0.95 mmol, 95%) as an orange solid, mp 183-185 °C. IR (nujol): 3287, 1529, 1343 cm^{-1}: ^{1}H NMR (400 MHz, DMSO-d_6): δ 9.76 (s, 1H), 8.44 (dd, *J* = 6.8, 3.1 Hz, 1H), 8.24 (dt, *J* = 9.0, 3.7 Hz, 1H), 7.57 (t, *J* = 9.8 Hz, 1H), 7.41 (d, *J* = 8.6 Hz, 2H), 7.18 (d, *J* = 8.6 Hz, 2H), 2.31 (s, 3H); ^{13}C NMR (100 MHz, DMSO-d_6): δ 161.3 (d, *J* =

256.8 Hz), 144.1, 143.4, 136.2, 131.1, 128.4 (d, J = 13.5 Hz), 124.3 (d, J = 10.9 Hz), 124.1 (d, J = 5.9 Hz), 117.4 (d, J = 25.7 Hz), 114.3, 110.1, 15.3.

(3-Chlorophenyl)hydrazone (**9f**): Yield: 261 mg (0.85 mmol, 85%) as an orange solid, mp 143-144 °C. IR (nujol): 3286, 1535, 1337 cm^{-1}: ^{1}H NMR (400 MHz, DMSO-d_6): δ 9.78 (s, 1H), 8.44 (dd, J = 6.3, 3.0 Hz, 1H), 8.23 (dt, J = 9.0, 3.7 Hz, 1H), 7.55 (t, J = 9.9 Hz, 1H), 7.26 (t, J = 8.0 Hz, 1H), 7.24 (s, 1H), 7.16 (d, J = 8.0 Hz, 1H), 6.83 (d, J = 7.6 Hz, 1H), 2.31 (s, 3H); ^{13}C NMR (100 MHz, DMSO-d_6): δ 163.5 (d, J = 257.8 Hz), 147.3, 144.5, 137.9, 134.1, 131.1, 129.3 (d, J = 13.5 Hz), 125.4 (d, J = 10.9 Hz), 125.2 (d, J = 6.2 Hz), 119.5, 118.5 (d, J = 25.7 Hz), 112.8, 112.1, 16.4.

(4-Chlorophenyl)hydrazone (**9g**): Yield: 301 mg (0.98 mmol, 98%) as an orange solid, mp 159-161 °C. IR (nujol): 3285, 1528, 1343 cm^{-1}: ^{1}H NMR (400 MHz, DMSO-d_6): δ 9.75 (s, 1H), 8.44 (dd, J = 6.6, 3.0 Hz, 1H), 8.24 (dt, J = 9.0, 3.7 Hz, 1H), 7.55 (t, J = 9.9 Hz, 1H), 7.29 (d, J = 8.8 Hz, 2H), 7.22 (d, J = 8.8 Hz, 2H), 2.31 (s, 3H); ^{13}C NMR (100 MHz, DMSO-d_6): δ 163.5 (d, J = 256.8 Hz), 144.8, 144.5, 137.2, 129.4 (d, J = 13.6 Hz), 129.3, 125.3 (d, J = 10.9 Hz), 125.2 (d, J = 5.9 Hz), 123.5, 118.5 (d, J = 25.8 Hz), 114.9, 16.4.

(2,4-Dichlorophenyl)hydrazone (**9h**): Yield: 289 mg (0.92 mmol, 92%) as a pale orange solid, mp 138-139 °C. IR (nujol): 3315, 1535, 1347 cm^{-1}: ^{1}H NMR (400 MHz, DMSO-d_6): δ 8.60 (s, 1H), 8.45 (dd, J = 6.6, 3.0 Hz, 1H), 8.29 (dt, J = 9.0, 3.8 Hz, 1H), 7.58 (t, J = 9.9 Hz, 1H), 7.58 (d, J = 2.3 Hz, 1H), 7.52 (d, J = 8.8 Hz, 1H), 7.38 (dd, J = 8.9, 2.4 Hz, 1H), 2.39 (s, 3H); ^{13}C NMR (100 MHz, DMSO-d_6): δ 160.5 (d, J = 256.4 Hz), 144.5, 142.1, 140.7, 129.2, 128.9 (d, J = 13.8 Hz), 128.7, 126.0 (d, J = 10.9 Hz), 125.4 (d, J = 5.7 Hz), 124.4, 119.1, 118.6 (d, J = 25.2 Hz), 116.6, 16.1.

(3-(Trifluoromethyl)phenyl)hydrazone (**9i**): Yield: 270 mg (0.84 mmol, 84%) as a yellow solid, mp 122-123 °C. IR (nujol): 3294, 1533, 1342 cm^{-1}: ^{1}H NMR (400 MHz, DMSO-d_6): δ 9.93 (s, 1H), 8.46 (dd, J = 6.3, 3.0 Hz, 1H), 8.25 (dt, J = 8.9, 3.6 Hz, 1H), 7.56 (t, J = 9.8 Hz, 1H), 7.52-7.45 (complex, 3H), 7.13 (d, J = 6.1 Hz, 1H), 2.33 (s, 3H); ^{13}C NMR (100 MHz, DMSO-d_6) δ 163.5 (d, J = 257.1 Hz), 146.2, 144.5, 138.3, 130.6, 130.3 (q, J = 31.3 Hz), 129.2 (d, J = 13.2 Hz), 125.9 (d, J = 10.7 Hz), 125.2 (d, J = 4.8 Hz), 124.8 (q, J = 271.9 Hz), 118.5 (d, J = 25.6 Hz), 116.9, 1160 (q, J = 3.7 Hz), 109.5 (q, J = 3.9 Hz), 16.4.

(4-(Trifluoromethyl)phenyl)hydrazone (**9j**): Yield: 303 mg (0.94 mmol, 94%) as a yellow solid, mp 185-186 °C. IR (nujol): 3292, 1544, 1346 cm^{-1}: ^{1}H NMR (400 MHz, DMSO-d_6): δ 10.0 (s,

1H), 8.45 (dd, J = 6.7, 3.0 Hz, 1H), 8.26 (dt, J = 9.0, 3.7 Hz, 1H), 7.59 (d, J = 8.4 Hz, 2H), 7.56 (t, J = 9.8 Hz, 1H), 7.36 (d, J = 8.4 Hz, 2H), 2.34 (s, 3H); ^{13}C NMR (100 MHz, DMSO-d_6): δ 163.5 (d, J = 256.8 Hz), 148.5, 144.5, 139.0, 129.3 (d, J = 13.4 Hz), 126.8 (q, J = 9.1 Hz), 125.6 (d, J = 11.0 Hz), 125.4 (q, J = 270.1 Hz), 125.3 (d, J = 5.6 Hz), 119.9 (q, J = 24.9 Hz), 118.5 (d, J = 25.5 Hz), 113.2, 16.5.

(4-Cyanophenyl)hydrazone (**9k**): Yield: 292 mg (0.98 mmol, 98%) as a pale orange solid, mp 206-208 ^{o}C. IR (nujol): 2215, 1532, 1350 cm^{-1}: ^{1}H NMR (400 MHz, DMSO-d_6): δ 10.2 (s, 1H), 8.45 (d, J = 6.5, 3.0 Hz), 1H), 8.27 (m, 1H), 7.68 (d, J = 8.4 Hz, 2H), 7.57 (t, J = 9.8 Hz, 1H), 7.32 (d, J = 8.4 Hz, 2H), 2.35 (s, 3H); ^{13}C NMR (100 MHz, DMSO-d_6): δ 163.5 (d, J = 256.8 Hz), 149.3, 144.5, 140.2, 134.1, 129.1 (d, J = 13.7 Hz), 125.8 (d, J = 11.0 Hz), 125.4, 120.4, 118.6 (d, J = 25.5 Hz), 113.6, 100.9, 16.6.

(4-Sulfonamidophenyl)hydrazone (**9l**): Yield: 320 mg (0.91 mmol, 91%) as a yellow solid, mp 217-219 ^{o}C. IR (nujol): 3344, 3289, 3247, 1534, 1346, 1327, 1146 cm^{-1}: ^{1}H NMR (400 MHz, DMSO-d_6): δ 10.0 (s, 1H), 8.46 (dd, J = 6.7, 3.0 Hz, 1H), 8.26 (dt, J = 9.0, 3.7 Hz, 1H), 7.70 (d, J = 8.5 Hz, 2H), 7.57 (dd, J = 9.8, 9.0 Hz, 1H), 7.31 (d, J = 8.6 Hz, 2H), 7.11 (s, 2H), 2.35 (s, 3H); ^{13}C NMR (100 MHz, DMSO-d_6): δ 163.5 (d, J = 259.0 Hz), 148.3, 144.5, 139.0, 135.0, 129.3 (d, J = 13.7 Hz), 127.7, 125.4 (d, J = 10.8 Hz), 125.3 (d, J = 5.6 Hz), 118.5 (d, J = 25.0 Hz), 112.7, 16.6.

4-(Carboxyphenyl)hydrazone (**9m**): Yield: 285 mg (0.90 mmol, 90%) as a pale orange solid, mp 328-330 ^{o}C (dec). IR (nujol): 3451-2478, 3270, 1530, 1344 cm^{-1}: ^{1}H NMR (400 MHz, DMSO-d_6): δ 12.3 (br s, 1H), 10.0 (s, 1H), 8.46 (dd, J = 6.7, 3.0 Hz, 1H), 8.26 (dt, J = 9.0, 3.6 Hz, 1H), 7.85 (d, J = 8.5 Hz, 2H), 7.56 (t, J = 9.8 Hz, 1H), 7.27 (d, J = 8.5 Hz, 2H), 2.35 (s, 3H); ^{13}C NMR (100 MHz, DMSO-d_6): δ 167.7, 163.5 (d, J = 257.5 Hz), 149.4, 144.5, 138.9, 131.5, 129.3 (d, J = 13.7 Hz), 125.5 (d, J = 10.7 Hz), 125.3 (d, J = 5.7 Hz), 121.8, 118.5 (d, J = 25.6 Hz), 112.7, 16.5.

Characterization of arylhydrazones from 2-fluoro-5-nitrobenzaldehyde.

Phenylhydrazone (**10a**): Yield: 246 mg (0.95 mmol, 95%) as an orange solid, mp 167-169 ^{o}C. IR (nujol): 3338, 1531, 1336 cm^{-1}: ^{1}H NMR (400 MHz, DMSO-d_6): δ 10.9 (s, 1H), 8.64 (dd, J = 6.6, 3.0 Hz, 1H), 8.15 (dt, J = 9.2, 3.7 Hz, 1H), 8.01 (s, 1H), 7.52 (t, J = 9.6 Hz, 1H), 7.29 (t, J = 7.7 Hz, 2H), 7.12 (d, J = 7.9 Hz, 2H), 6.84 (t, J = 7.3 Hz, 1H; ^{13}C NMR (100 MHz, DMSO-d_6): δ 162.9 (d, J = 256.9 Hz), 144.9, 144.8, 129.7, 126.8 (d, J = 3.7 Hz), 125.4 (d, J = 12.8 Hz), 124.4 (d, J = 10.2 Hz), 120.8, 120.4, 117.9 (d, J = 23.9 Hz), 112.8.

(2-Methoxyphenyl)hydrazone (**10b**): Yield: 231 mg (0.80 mmol, 80%) as an orange solid, mp 194-196 °C. IR (nujol): 3300, 1527, 1344 cm^{-1}: ^{1}H NMR (400 MHz, DMSO-d_6): δ 10.4 (s, 1H), 8.66 (dd, *J* = 6.3, 2.0 Hz), 8.37 (s, 1H), 8.16 (dt, *J* = 8.7, 3.5 Hz, 1H), 7.52 (t, *J* = 9.5 Hz, 1H), 7.43 (d, *J* = 7.9 Hz, 1H), 6.98 (d, *J* = 8.4 Hz, 1H), 6.95 (t, *J* = 7.8 Hz, 1H), 6.63 (t, *J* = 7.7 Hz, 1H), 3.87 (s, 3H); ^{13}C NMR (100 MHz, DMSO-d_6): δ 162.8 (d, *J* = 257.7 Hz), 145.9, 145.0, 134.0, 128.4 (d, *J* = 4.0 Hz), 125.7 (d, *J* = 12.9 Hz), 124.5 (d, *J* = 10.4 Hz), 121.8, 120.8 (d, *J* = 6.0 Hz), 120.3, 117.9 (d, *J* = 23.9 Hz), 112.5, 111.6, 56.1.

(3-Methoxyphenyl)hydrazone (**10c**): Yield: 237 mg (0.82 mmol, 82%) as an orange solid, mp 196-198 °C. IR (nujol): 3294, 1519, 1345 cm^{-1}: ^{1}H NMR (400 MHz, DMSO-d_6): δ 10.9 (s, 1H), 8.65 (dd, *J* = 6.3, 3.0 Hz, 1H), 8.16 (ddd, *J* = 8.9, 4.3, 1.8 Hz, 1H), 8.00 (s, 1H), 7.53 (t, *J* = 9.5 Hz, 1H), 7.18 (t, *J* = 8.0 Hz, 1H), 6.71-6.65 (complex, 3H), 6.44 (dd, *J* = 8.1, 2.5 Hz, 1H), 3.76 (s, 3H); ^{13}C NMR (100 MHz, DMSO-d_6): δ 162.7 (d, *J* = 257.9 Hz), 160.8, 146.1, 145.0, 130.7, 127.0 (d, *J* = 3.7 Hz), 125.4 (d, *J* = 12.7 Hz), 124.6 (d, *J* = 10.5 Hz), 121.0 (d, *J* = 6.0 Hz), 118.0 (d, *J* = 23.8 Hz), 105.8, 105.7, 98.7, 55.4.

(4-Methoxyphenyl)hydrazone (**10d**): Yield: 260 mg (0.90 mmol, 90%) as an orange soid, mp 154-155 °C. IR (nujol): 3301, 1524, 1344 cm^{-1}: ^{1}H NMR (400 MHz, DMSO-d_6): δ 10.8 (s, 1H), 8.63 (dd, *J* = 6.2, 3.0 Hz), 8.12 (dt, *J* = 9.3, 3.7 Hz, 1H), 7.93 (s, 1H), 7.51 (t, *J* = 9.6 Hz, 1H), 7.06 (d, *J* = 8.5 Hz, 2H), 6.90 (d, *J* = 8.5 Hz, 2H), 3.71 (s, 3H); ^{13}C NMR (100 MHz, DMSO-d_6): δ 161.3 (d, *J* = 256.7 Hz), 153.9, 145.0, 138.7, 125.7 (d, *J* = 12.7 Hz), 125.3, 123.9 (d, *J* = 10.3 Hz), 120.6 (d, *J* = 6.0 Hz), 117.8 (d, *J* = 24.0 Hz), 115.2, 113.9, 55.7.

(4-Bromophenyl)hydrazone (**10e**): Yield: 317 mg (0.94 mmol, 94%) as an orange solid, mp 184-186 °C: IR (nujol): 3327, 1532, 1339 cm^{-1}: ^{1}H NMR (400 MHz, DMSO-d_6): δ 11.0 (s, 1H), 8.64 (dd, *J* = 6.3, 2.9 Hz, 1H), 8.18 (dt, *J* = 9.1, 3.8 Hz, 1H), 8.01 (s, 1H), 7.53 (t, *J* = 9.5 Hz, 1H), 7.43 (d, *J* = 8.4 Hz, 2H), 7.07 (d, *J* = 8.4 Hz, 2H); ^{13}C NMR (100 MHz, DMSO-d_6): δ 162.8 (d, *J* = 257.4 Hz), 144.9, 144.1, 132.4, 127.9, 125.1 (d, *J* = 12.4 Hz), 124.8 (d, *J* = 10.3 Hz), 121.0 (d, *J* = 5.7 Hz), 118.0 (d, *J* = 23.9 Hz), 114.8, 111.3.

(3-Chlorophenyl)hydrazone (**10f**): Yield: 264 mg (0.90 mmol, 90%) as an orange solid, mp 197-199 °C. IR (nujol): 3288, 1523, 1343 cm^{-1}: ^{1}H NMR (400 MHz, DMSO-d_6): δ 11.0 (s, 1H), 8.65 (dd, *J* = 6.2, 3.0 Hz, 1H), 8.18 (dt, *J* = 9.2, 3.5 Hz, 1H), 8.02 (s, 1H), 7.53 (t, *J* = 9.5 Hz, 1H), 7.28 (t, *J* = 8.0 Hz, 1H), 7.12 (s, 1H), 7.02 (d, *J* = 8.1 Hz, 1H), 6.66 (d, *J* = 7.3 Hz, 1H); ^{13}C NMR (100 MHz, DMSO-d_6): δ 161.8 (d, *J* = 258.5 Hz), 145.2, 143.9, 133.3, 130.3, 127.5 (d, *J* = 3.4

Hz), 124.0 (d, J = 10.5 Hz), 123.9 (d, J = 12.7 Hz), 120.2 (d, J = 5.7 Hz), 118.7, 116.9 (d, J = 24.0 Hz), 111.1, 110.5.

(4-Chlorophenyl)hydrazone (**10g**): Yield: 270 mg (0.92 mmol, 92%) as an orange solid, mp 189-191 °C. IR (nujol): 3325, 1533, 1335 cm^{-1}: ^{1}H NMR (400 MHz, DMSO-d_6): δ 11.0 (s, 1H), 8.64 (dd, J = 6.3, 3.1 Hz, 1H), 8.17 (dt, J = 9.0, 3.8 Hz, 1H), 8.00 (s, 1H), 7.53 (t, J = 9.5 Hz, 1H), 7.31 (d, J = 8.4 Hz, 2H), 7.11 (d, J = 8.4 Hz, 2H); ^{13}C NMR (100 MHz, DMSO-d_6): δ 162.8 (d, J = 257.0 Hz), 145.0, 143.7, 129.6, 127.8 (d, J = 3.6 Hz), 125.1 (d, J = 12.7 Hz), 124.8 (d, J = 11.2 Hz), 123.7, 121.0 (d, J = 5.7 Hz), 118.0 (d, J = 23.9 Hz).

(2,4-Dichlorophenyl)hydrazone (**10h**): Yield: 307 mg (0.94 mmol, 94%) as a yellow soid, mp 198-200 °C. IR (nujol): 3297, 1530, 1343 cm^{-1}: ^{1}H NMR (400 MHz, DMSO-d_6): δ 10.6 (s, 1H), 8.66 (dd, J = 6.3, 3.0 Hz, 1H), 8.53 (s, 1H), 8.22 (dt, J = 9.1, 3.7 Hz, 1H), 7.58 (d, J = 8.9 Hz, 1H), 7.56 (t, J = 9.5 Hz, 1H), 7.50 (d, J = 2.4 Hz, 1H), 7.38 (dd, J = 8.9, 2.4 Hz, 1H); ^{13}C NMR (100 MHz, DMSO-d_6): δ 163.2 (d, J = 258.2 Hz), 145.0, 140.3, 131.8 (d, J = 4.1 Hz), 129.2, 128.8, 125.5 (d, J = 10.5 Hz), 14.8 (d, J = 12.6 Hz), 123.8, 121.2 (d, J = 5.5 Hz), 118 (d, J = 23.9 Hz), 117.6, 115.9.

(3-(Trifluoromethyl)phenyl)hydrazone (**10i**): Yield: 286 mg (0.93 mmol, 93%) as a yellow solid, mp 182-183 °C. IR (nujol): 3299, 1530, 1347 cm^{-1}: ^{1}H NMR (400 MHz, DMSO-d_6): δ 11.2 (s, 1H), 8.66 (dd, J = 6.3, 2.9 Hz, 1H), 8.20 (ddd, J = 9.1, 4.4, 2.9 Hz, 1H), 8.06 (s, 1H), 7.55 (t, J = 9.9 Hz, 1H), 7.50 (t, J = 7.8 Hz, 1H), 7.37 (d, J = 7.8 Hz, 1H), 7.34 (s, 1H), 7.15 (d, J = 8.0 Hz, 1H); ^{13}C NMR (100 MHz, DMSO-d_6): δ 162.9 (d, J = 258.7 Hz), 145.5, 145.0, 131.0, 130.5 (q, J = 31.2 Hz), 129.1 (d, J = 3.4 Hz), 125.9 (d, J = 10.2 Hz), 124.9 (d, J = 12.6 Hz), 124.7 (q, J = 272.3 Hz), 121.4 (d, J = 5.6 Hz), 117.9 (d, J = 24.0 Hz), 116.5, 116.2 (q, J = 3.8 Hz), 108.6 (q, J = 3.8 Hz).

(4-(Trifluoromethyl)phenyl)hydrazone (**10j**): Yield: 286 mg (0.93 mmol, 93%) as a yellow solid, mp 203-204 °C. IR (nujol): 3290, 1517, 1342 cm^{-1}: ^{1}H NMR (400 MHz, DMSO-d_6): δ 11.3 (s, 1H), 8.67 (dd, J = 6.2, 3.0 Hz, 1H), 8.21 (dt, J = 9.1, 3.8 Hz, 1H), 8.10 (s, 1H), 7.61 (d, J = 8.4 Hz, 2H), 7.56 (t, J = 9.5 Hz, 1H), 7.24 (d, J = 8.4 Hz, 2H); ^{13}C NMR (100 MHz, DMSO-d_6): δ 163.0 (d, J = 258.4 Hz), 147.9, 145.0, 129.7 (d, J = 3.4 Hz), 127.2 (q, J = 3.9 Hz), 125.3 (d, J = 10.3 Hz), 125.3 (q, J = 270.6 Hz), 124.8 (d, J = 12.7 Hz), 121.3 (d, J = 5.5 Hz), 120.1 (q, J = 32.0 Hz), 118.1 (d, J = 23.9 Hz), 112.7.

(4-Cyanophenyl)hydrazone (**10k**): Yield: 261 mg (0.92 mmol, 92%) as a yellow solid, mp 223-

225 °C. IR (nujol): 3283, 2209, 1519, 1345 cm^{-1}: ^{1}H NMR (400 MHz, DMSO-d_6): δ 11.4 (s, 1H), 8.67 (dd, J = 6.3, 3.0 Hz, 1H), 8.28 (dt, J = 9.2, 3.7 Hz, 1H), 8.12 (s, 1H), 7.69 (d, J = 8.4 Hz, 2H), 7.57 (t, J = 9.5 Hz, 1H), 7.21 (d, J = 8.4 Hz, 2H); ^{13}C NMR (100 MHz, DMSO-d_6): δ 163.1 (d, J = 258.5 Hz), 148.2, 144.9, 134.2, 130.7, 125.6 (d, J = 10.5 Hz), 124.6 (d, J = 12.5 Hz), 121.4 (d, J = 5.5 Hz), 120.3, 118.1 (d, J = 24.0 Hz), 113.0, 101.1.

(4-Sulfonamidophenyl)hydrazone (**10l**): Yield: 306 mg (0.90 mmol, 90%) as a yellow solid, mp 279-280 °C. IR (nujol): 3350, 3344, 3250, 1518, 1346, 1320, 1145 cm^{-1}: ^{1}H NMR (400 MHz, DMSO-d_6): δ 11.3 (s, 1H), 8.69 (dd, J = 6.2, 3.0 Hz, 1H), 8.22 (dt, J = 9.1 , 3.8 Hz, 1H), 8.11 (s, 1H), 7.73 (d, J = 8.4 Hz, 2H), 7.57 (t, J = 9.5 Hz, 1H), 7.21 (d, J = 8.4 Hz, 2H), 7.14 (s, 2H) ; ^{13}C NMR (100 MHz, DMSO-d_6): δ 163.0 (d, J = 258.4 Hz), 147.3, 145.0, 135.2, 129.8, 128.1, 125.4 (d, J = 10.2 Hz), 124.8 (d, J = 12.6 Hz), 121.1 (d, J = 5.7 Hz), 118.1 (d, J = 24.1 Hz), 112.2.

4-(Carboxyphenyl)hydrazone (**10m**): Yield: 297 mg (0.98 mmol, 98%) as a yellow solid, mp 284-285 °C (dec). IR (nujol): 3458-2294, 3367, 1683, 1535, 1347 cm^{-1}: ^{1}H NMR (400 MHz, DMSO-d_6): δ 12.4 (br s, 1H), 11.3 (s, 1H), 8.68 (dd, J = 6.2, 3.0 Hz, 1H), 8.21 (dt, J = 9.1, 3.8 Hz, 1H), 8.11 (s, 1H), 7.88 (d, J = 8.4 Hz, 2H), 7.56 (t, J = 9.5 Hz, 1H), 7.16 (d, J = 8.4 Hz, 2H); ^{13}C NMR (100 MHz, DMSO-d_6): δ 167.6, 163.0 (d, J = 258.6 Hz), 148.4, 145.0, 131.8, 129.7, 125.3 (d, J = 10.6 Hz), 124.9 (d, J = 12.6 Hz), 122.0, 121.3 (d, J = 5.6 Hz), 118.1 (d, J = 24.1 Hz), 112.2.

[1]H Spectrum of phenylhydrazone **9a**

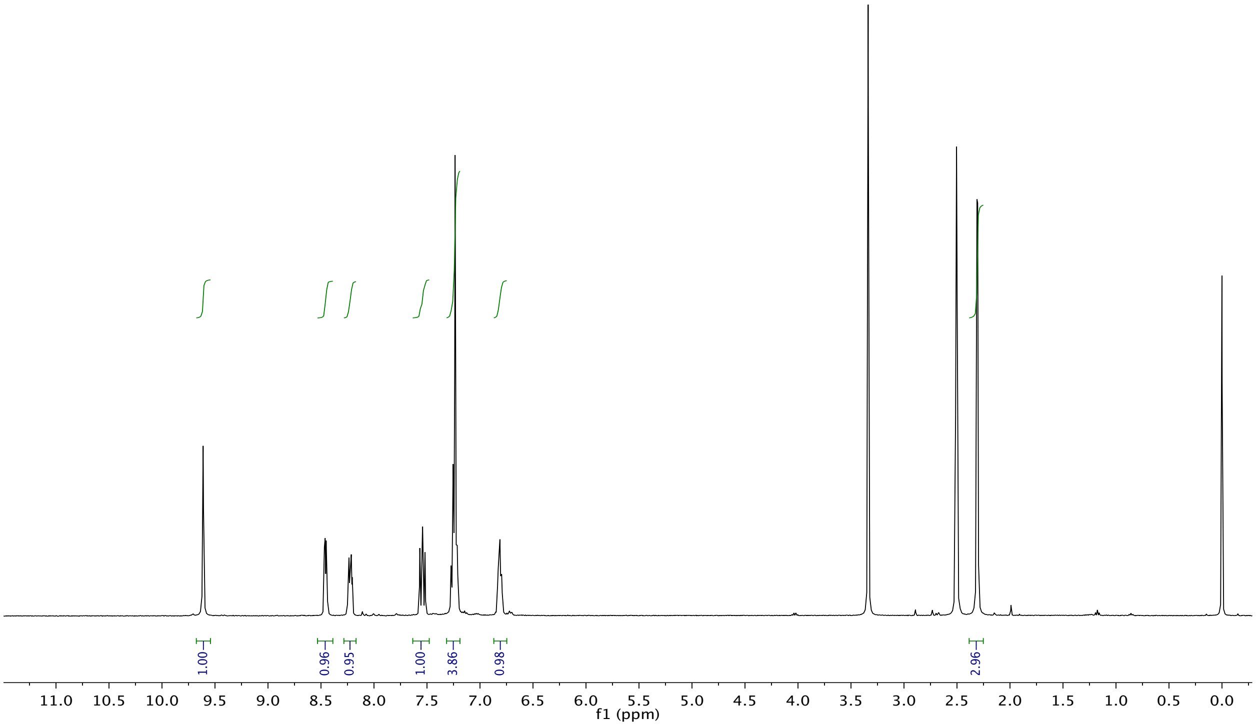

[13]C Spectrum of phenylhydrazone **9a**

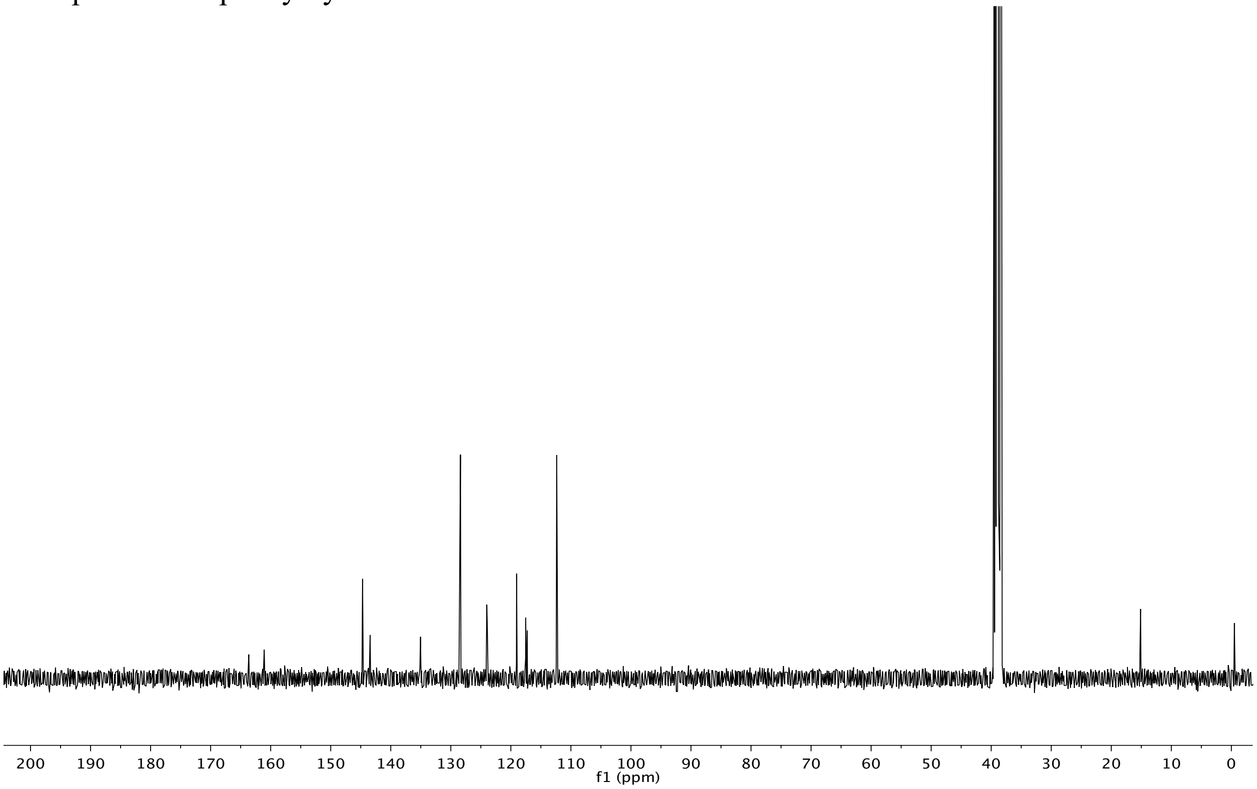

[1]H Spectrum of (2-methoxyphenyl)hydrazone **9b**

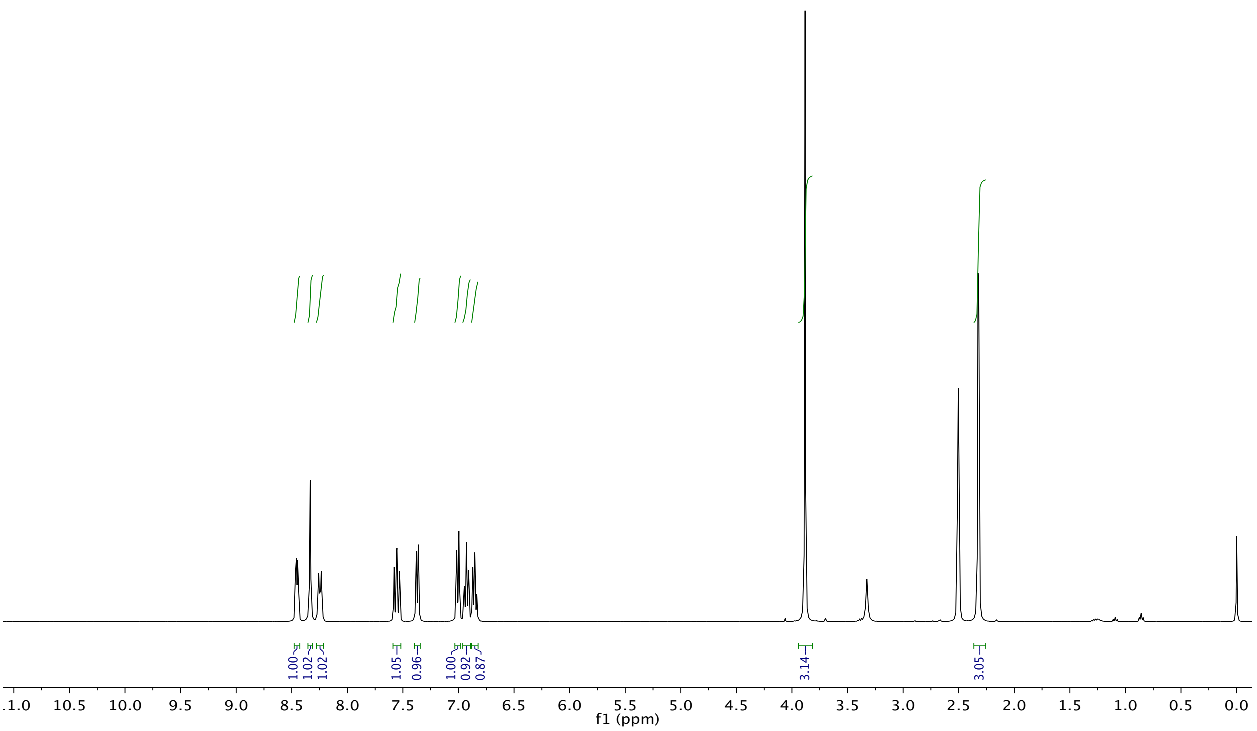

[13]C Spectrum of (2-methoxyphenyl)hydrazone **9b**

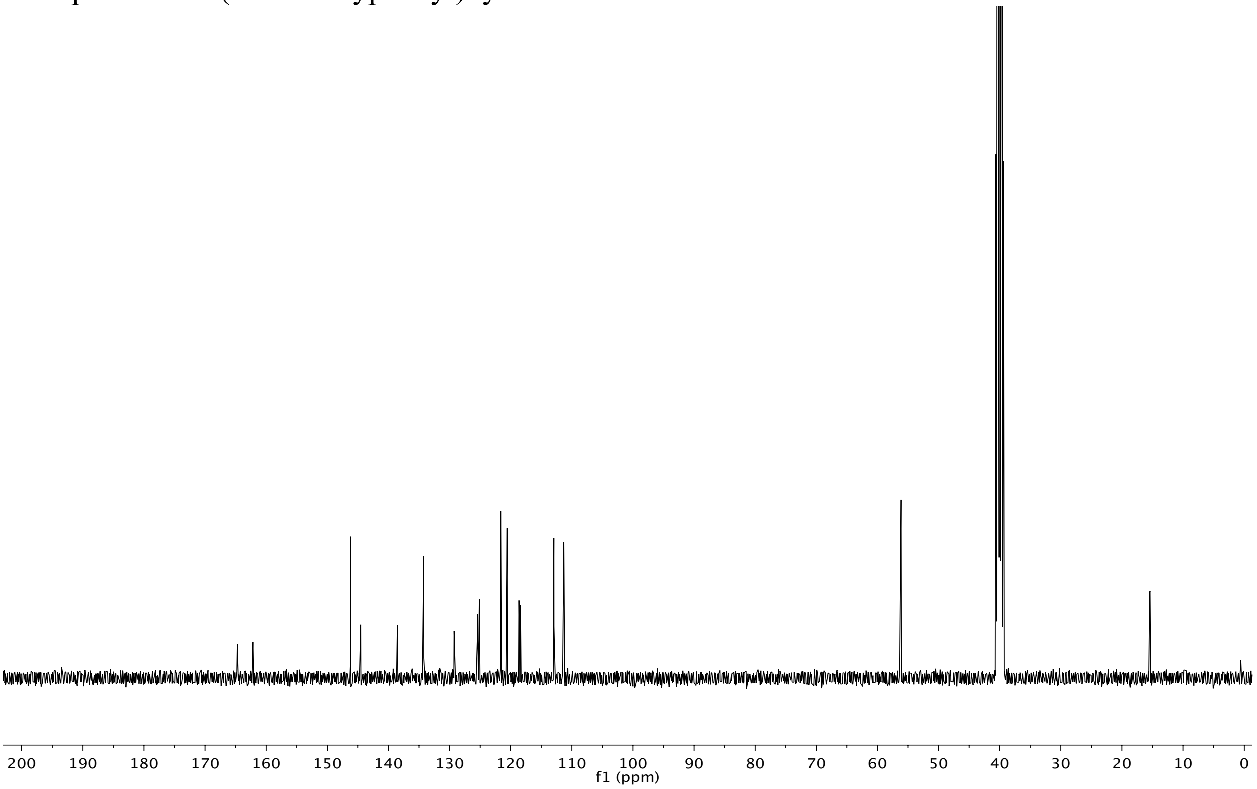

^{13}C Spectrum of (3-methoxyphenyl)hydrazone **9c**

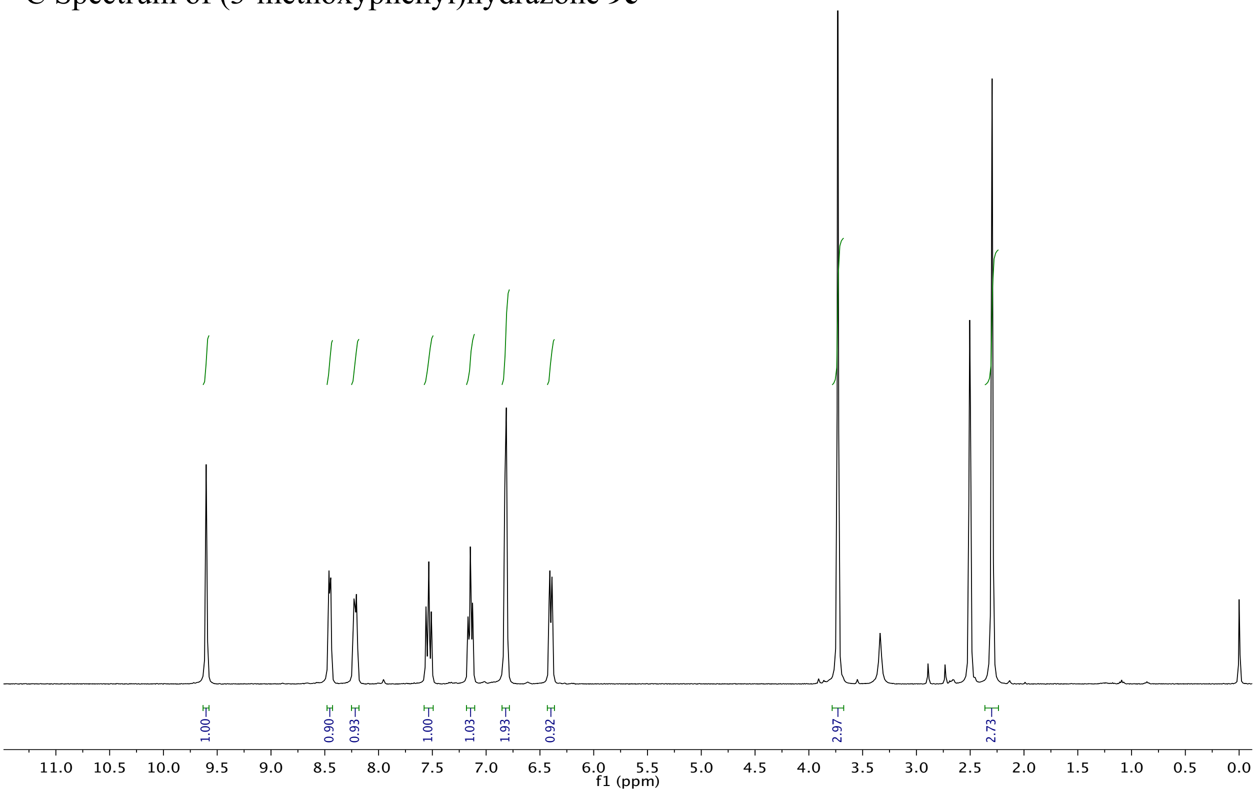

^{13}C Spectrum of (3-methoxyphenyl)hydrazone **9c**

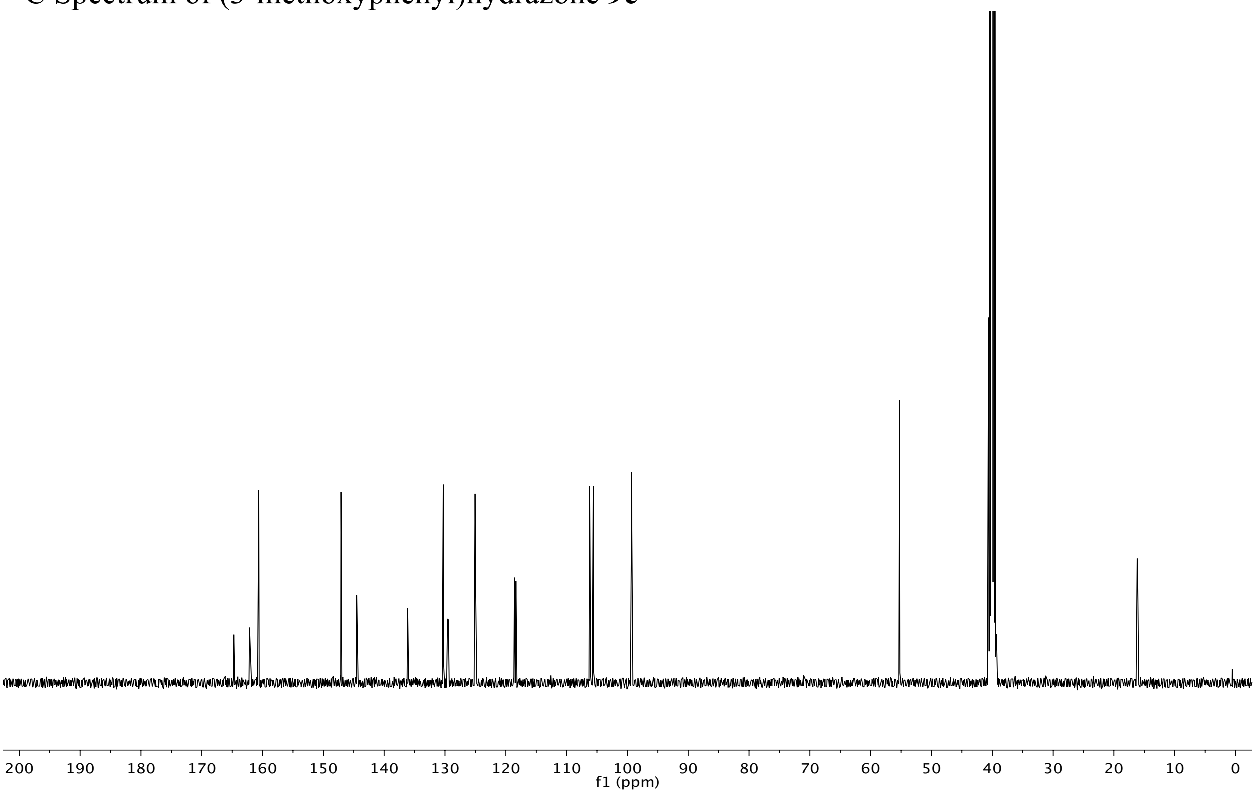

^{13}C Spectrum of (4-methoxyphenyl)hydrazone **9d** (*E* and *Z*)

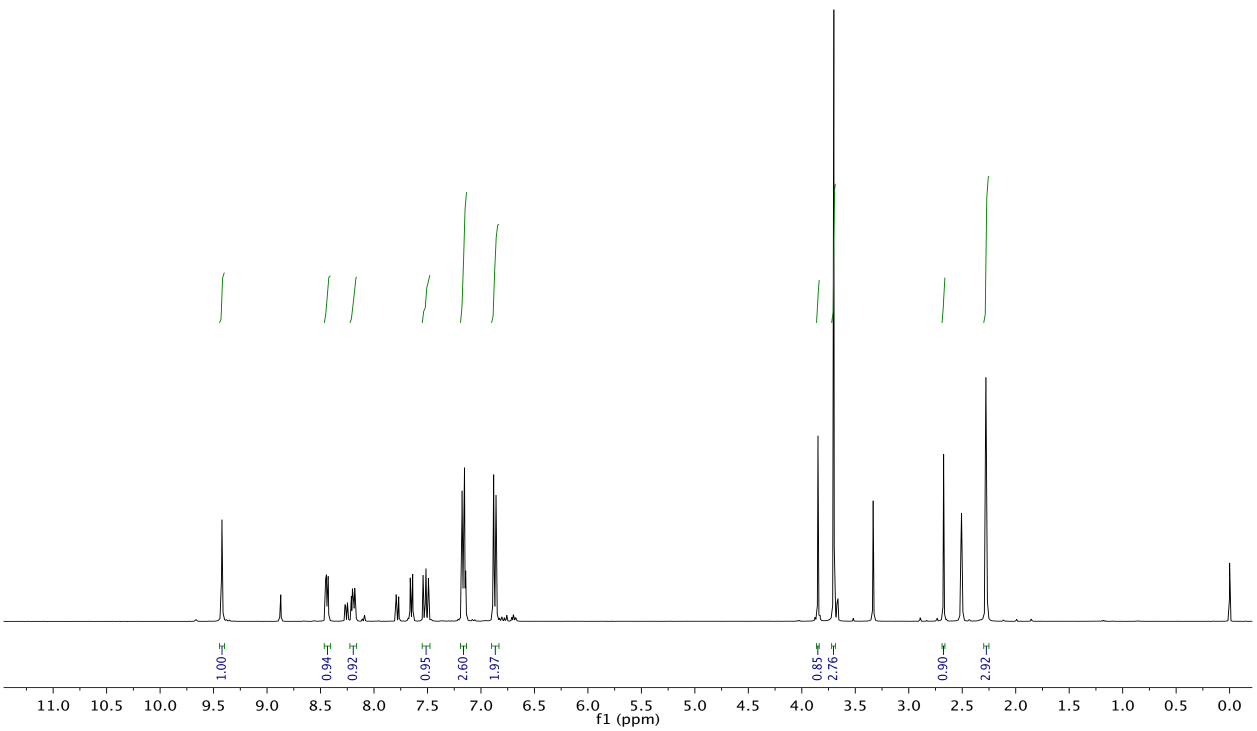

^{13}C Spectrum of (4-methoxyphenyl)hydrazone **9d** (*E* and *Z*)

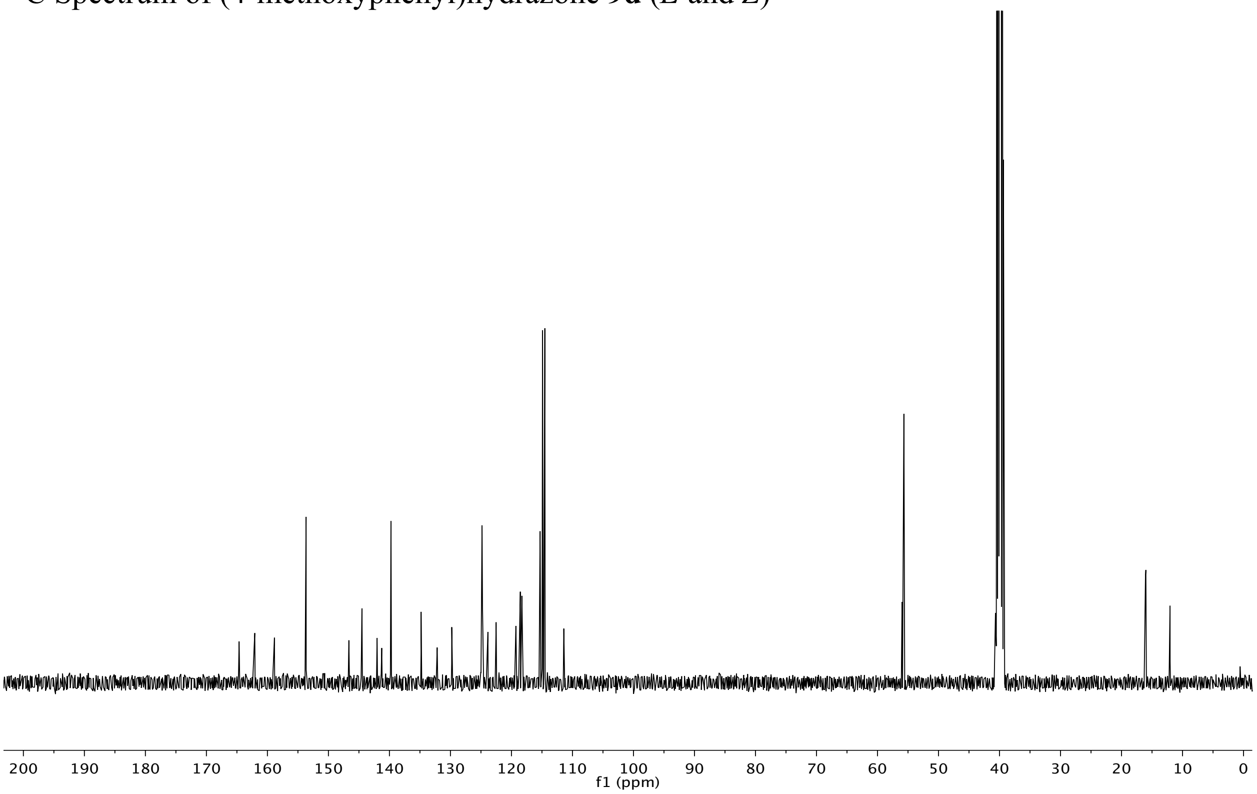

^{1}H Spectrum of (4-bromophenyl)hydrazone **9e**

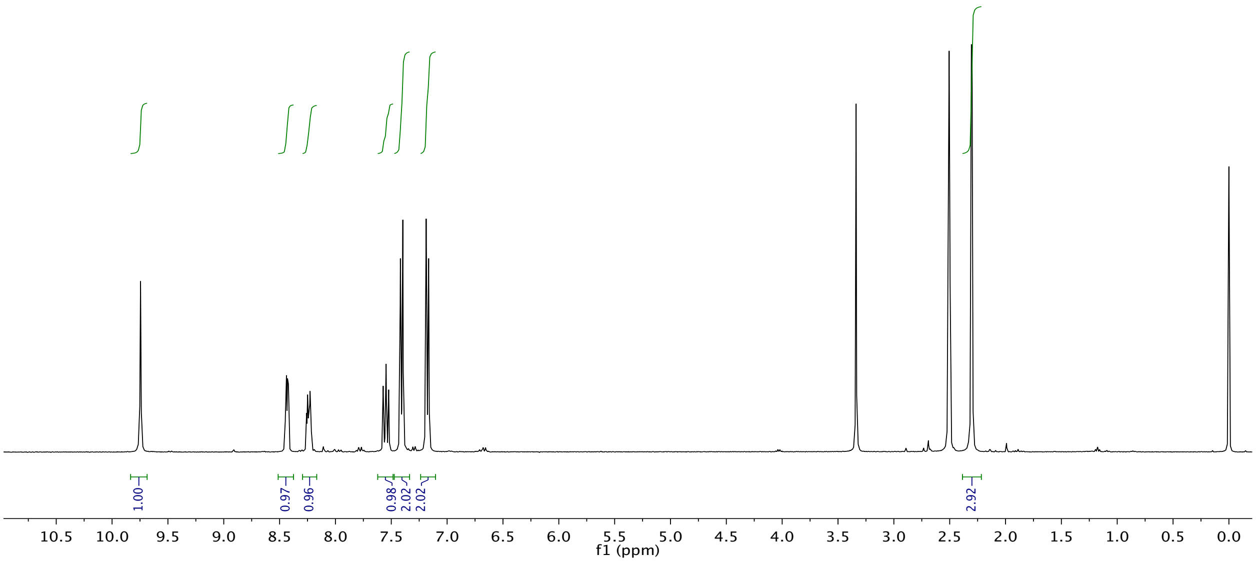

^{13}C Spectrum of (4-bromophenyl)hydrazone **9e**

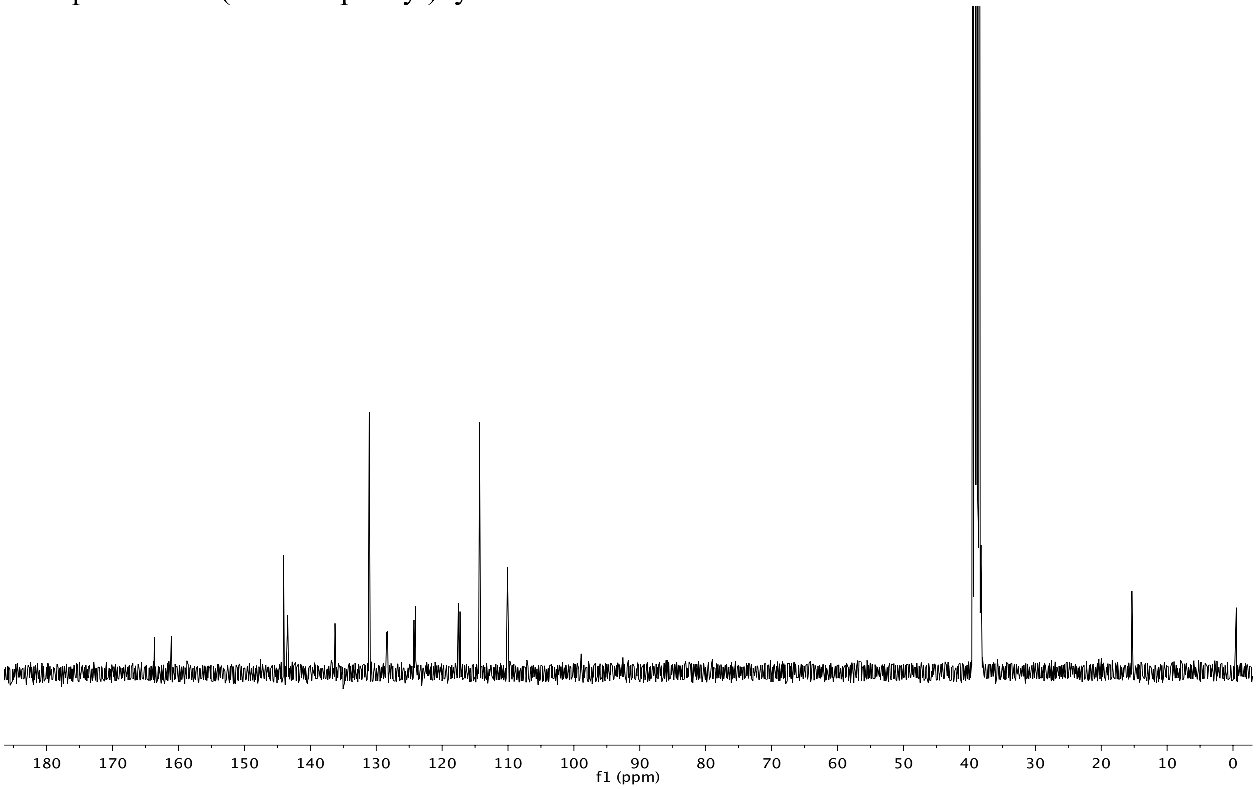

[1]H Spectrum of (3-chlorophenyl)hydrazone **9f**

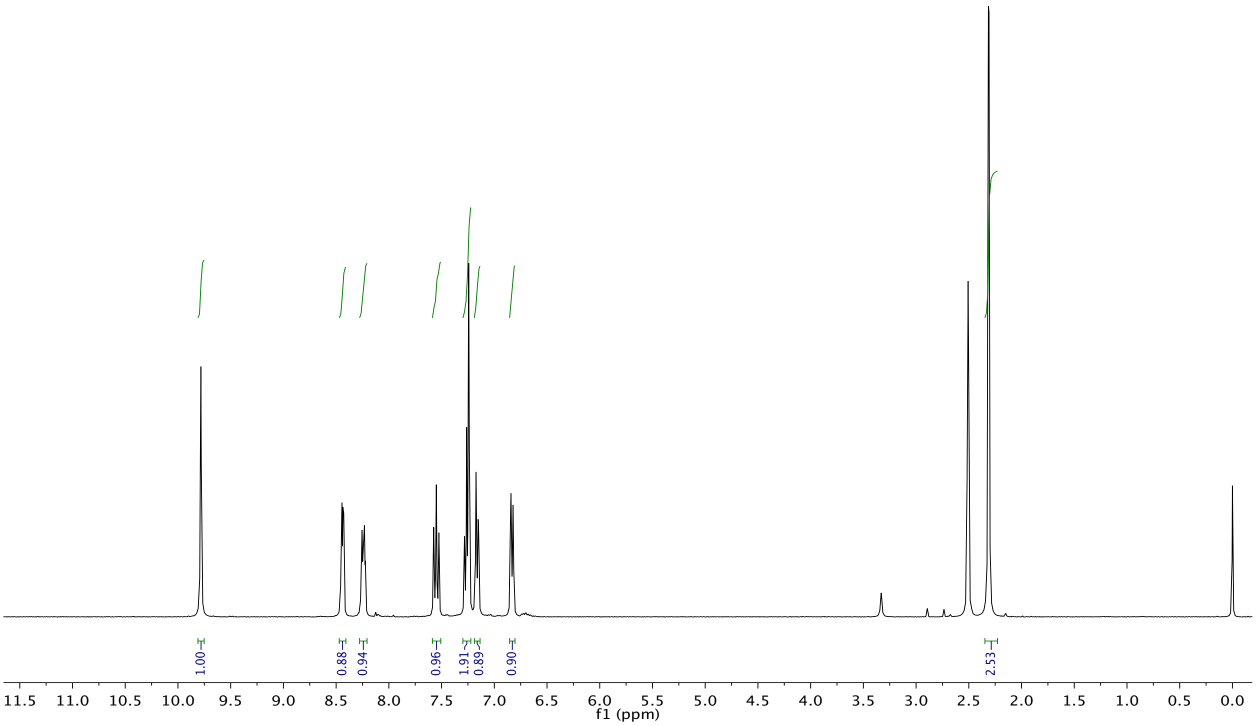

[13]C Spectrum of (3-chlorophenyl)hydrazone **9f**

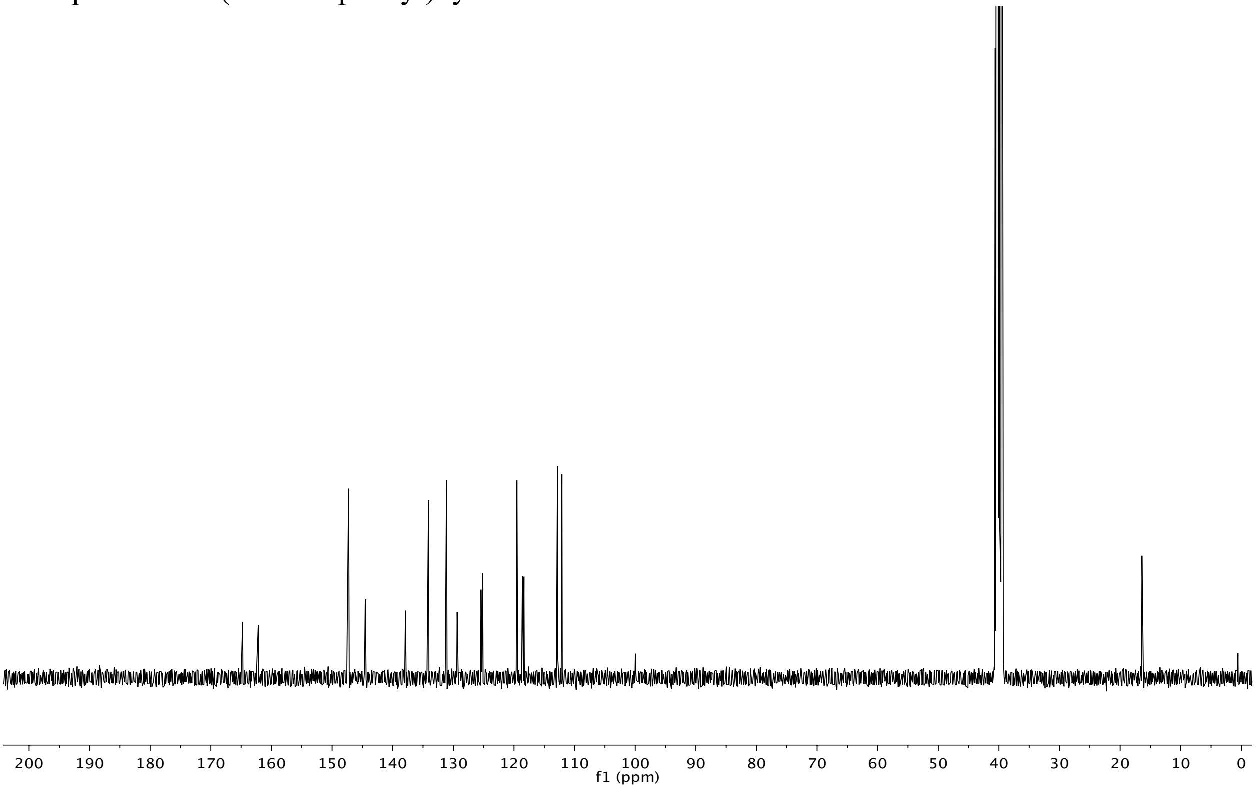

^{1}H Spectrum of (4-chlorophenyl)hydrazone **9g**

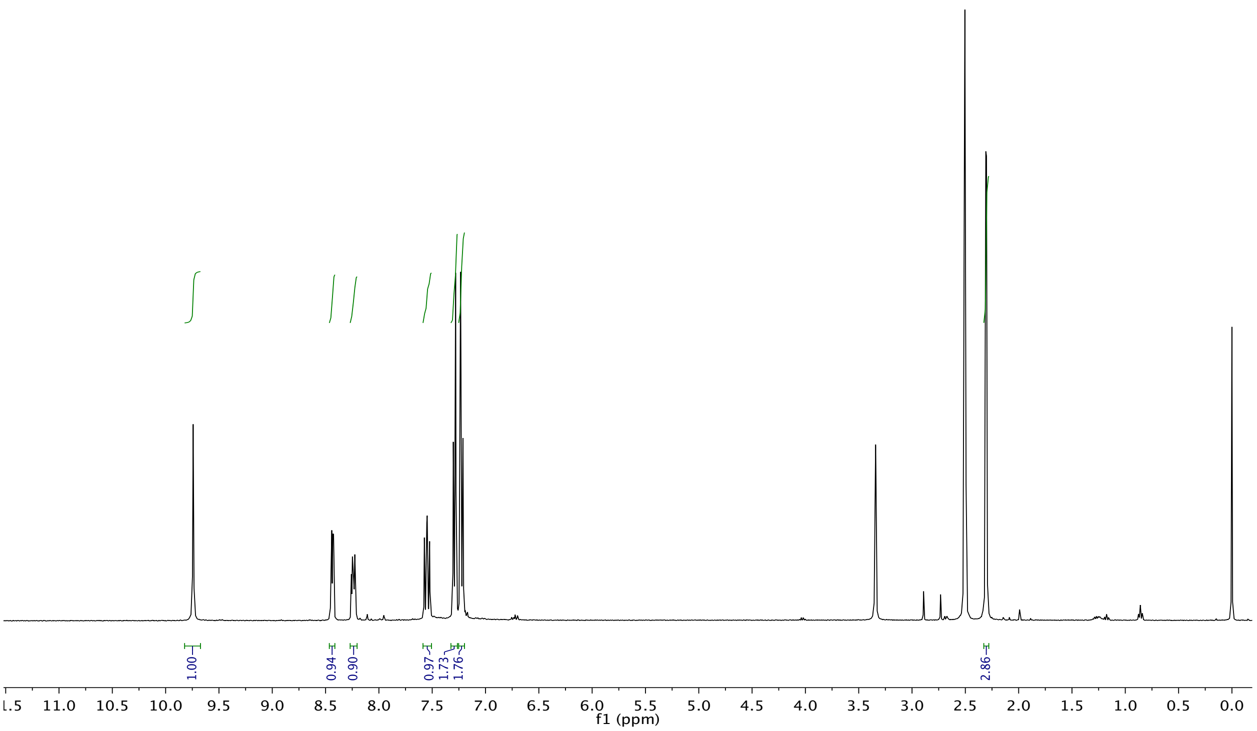

^{13}C Spectrum of (4-chlorophenyl)hydrazone **9g**

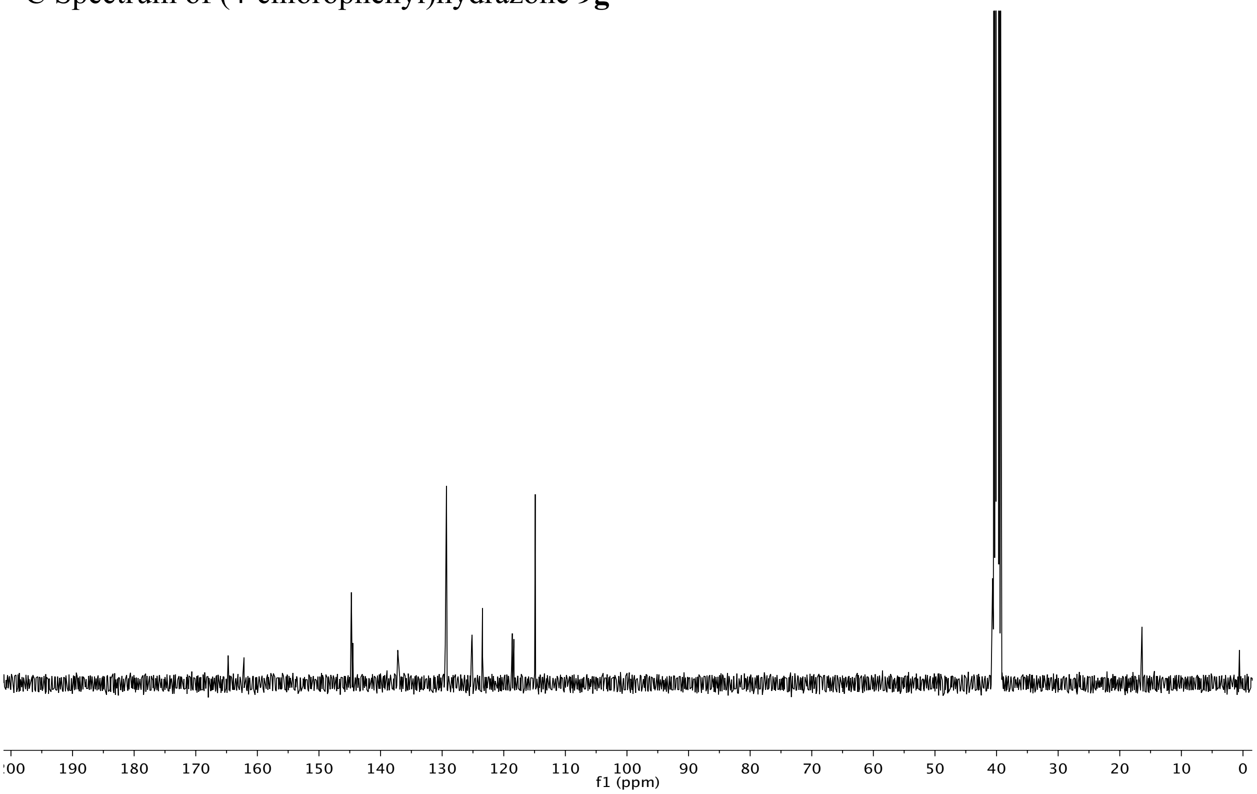

[1]H Spectrum of (2,4-dichlorophenyl)hydrazone **9h**

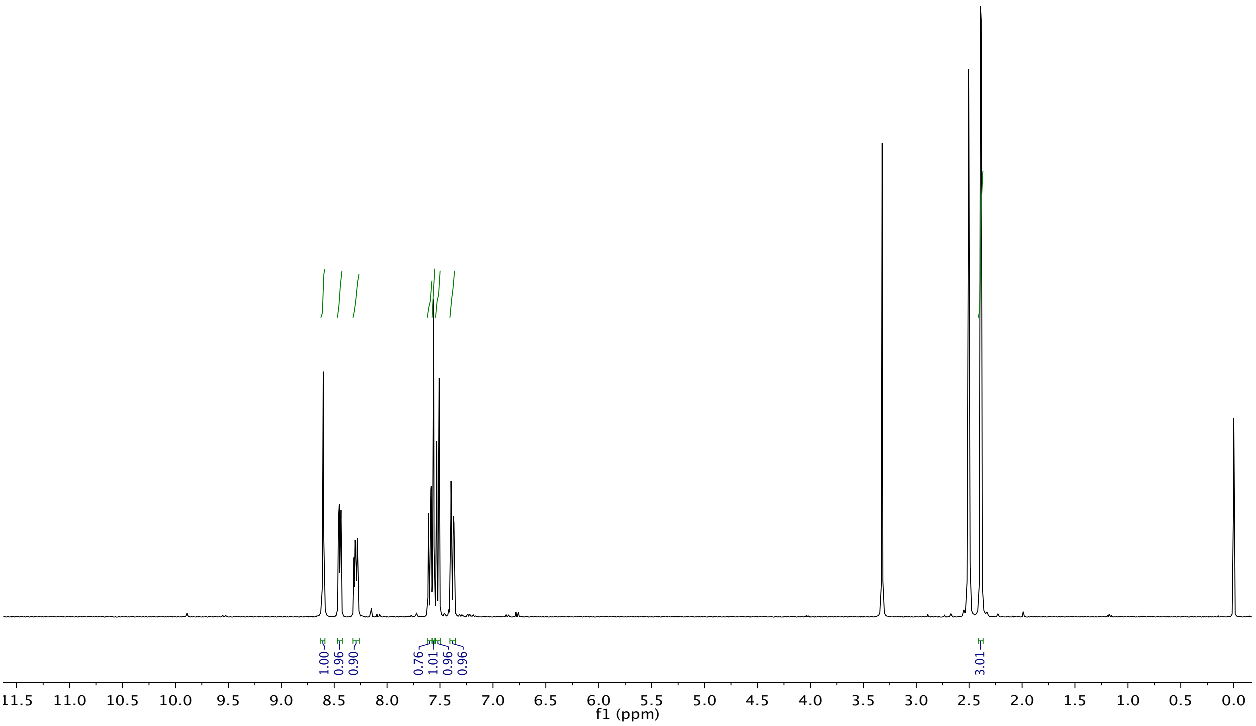

[13]C Spectrum of (2,4-dichlorophenyl)hydrazone **9h**

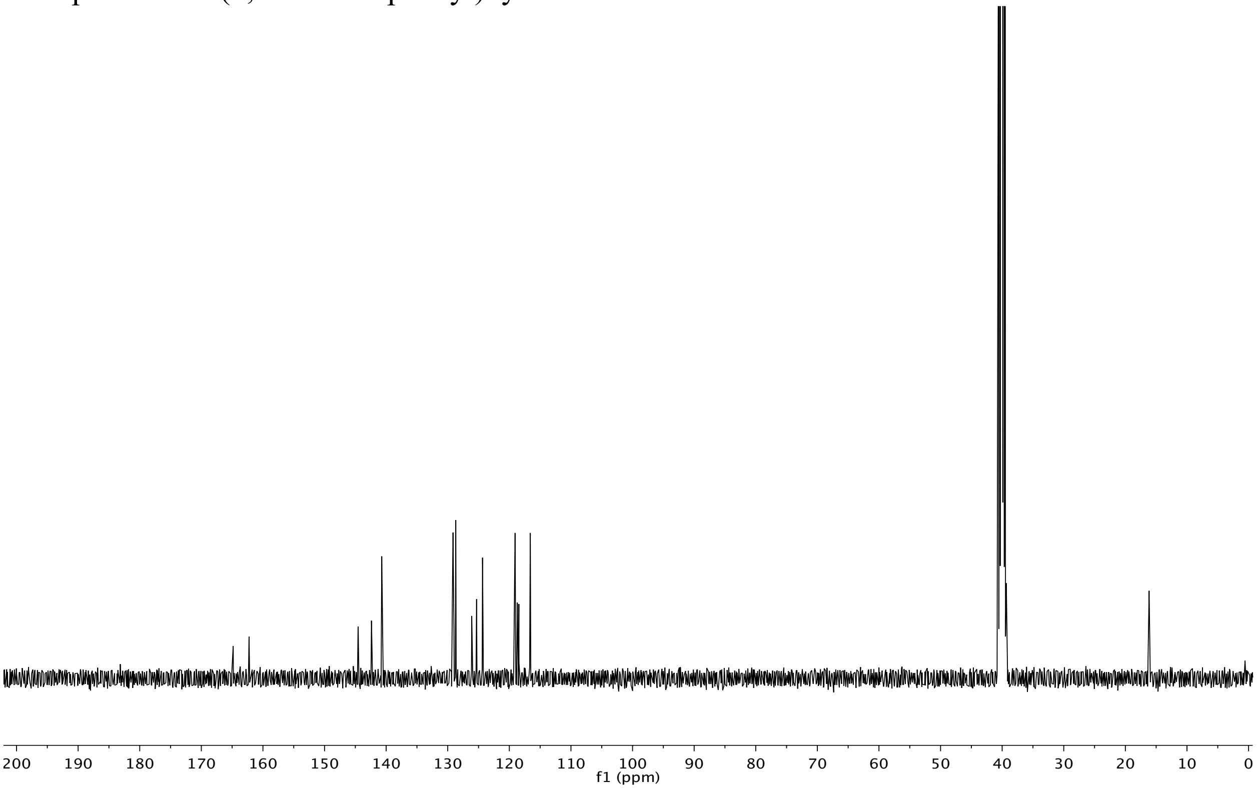

[1]H Spectrum of (3-(trifluoromethyl)phenyl)hydrazone **9i**

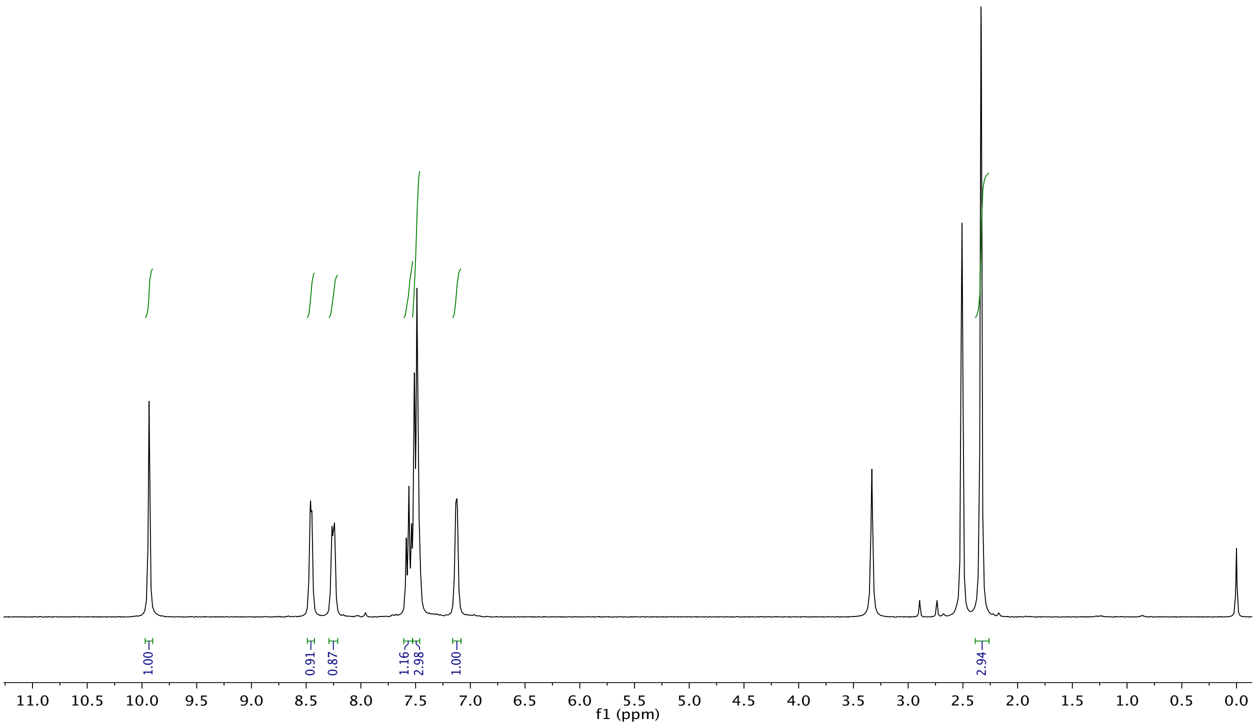

[13]C Spectrum of (3-(trifluoromethyl)phenyl)hydrazone **9i**

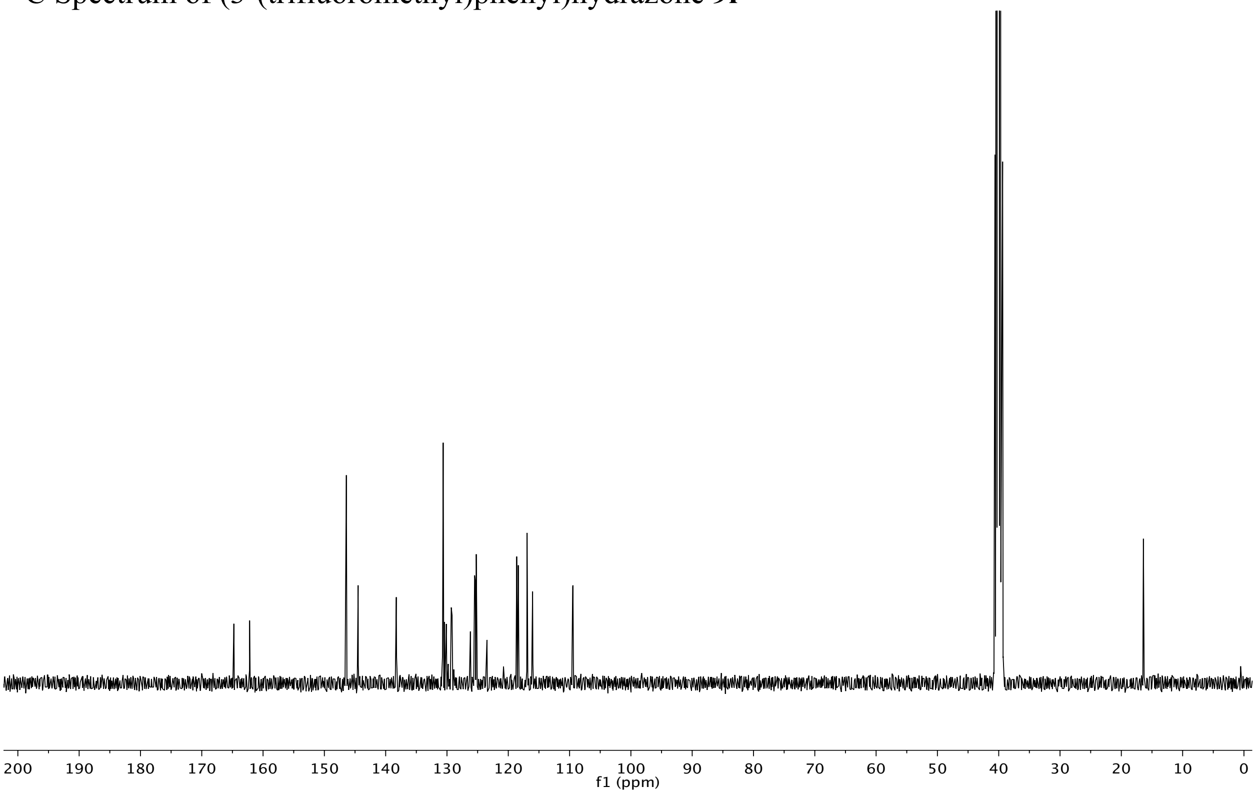

^{1}H Spectrum of (4-(trifluoromethyl)phenyl)hydrazone **9j**

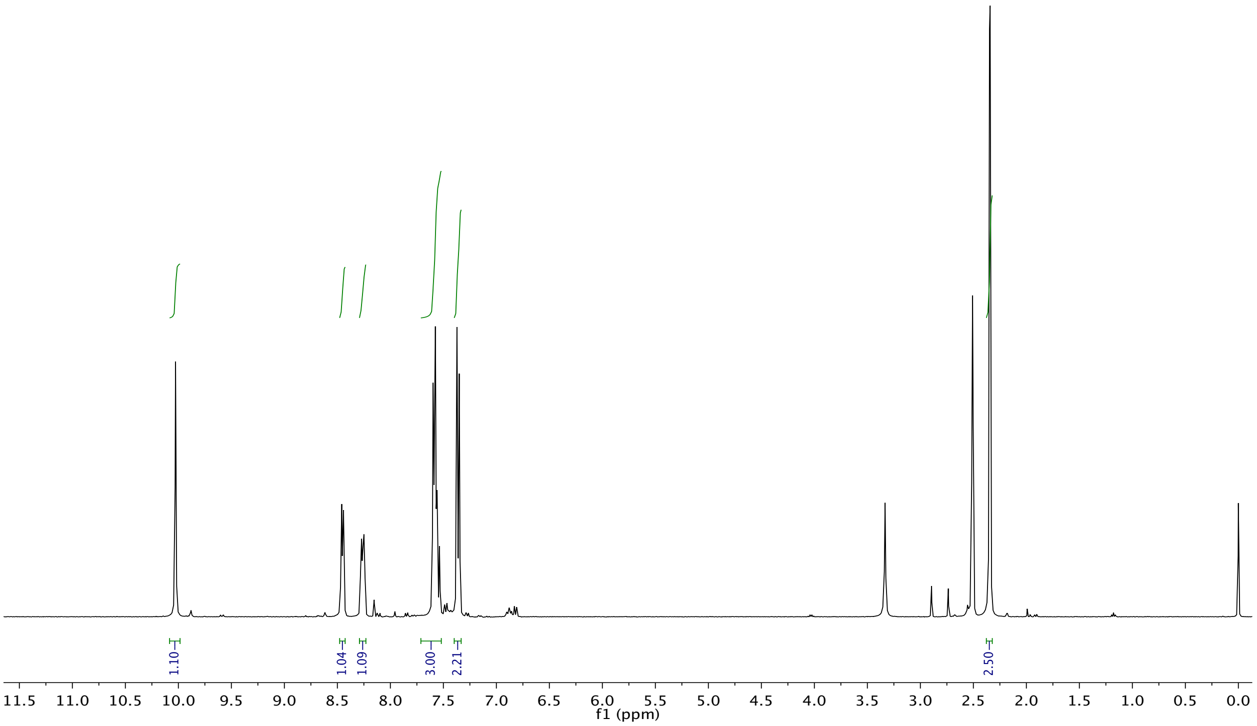

^{13}C Spectrum of (4-(trifluoromethyl)phenyl)hydrazone **9j**

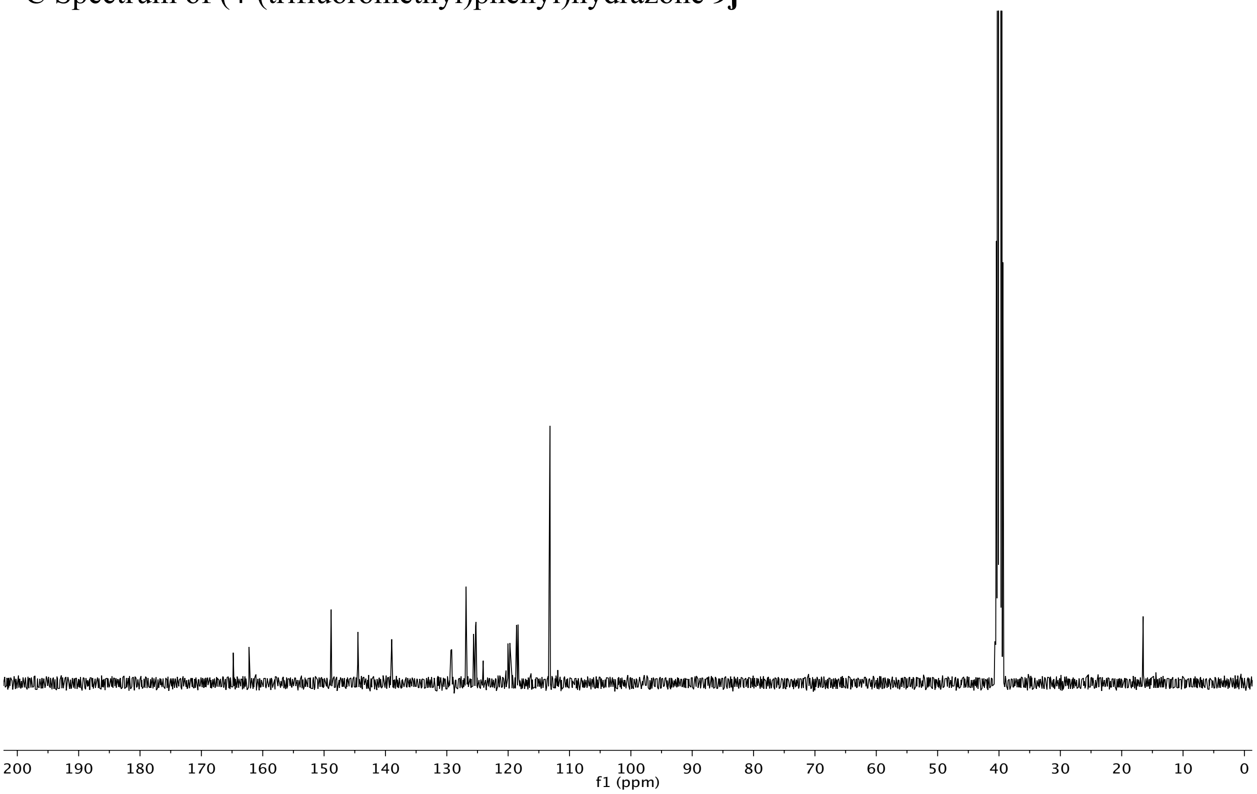

[1]H Spectrum of (4-cyanophenyl)hydrazone **9k**

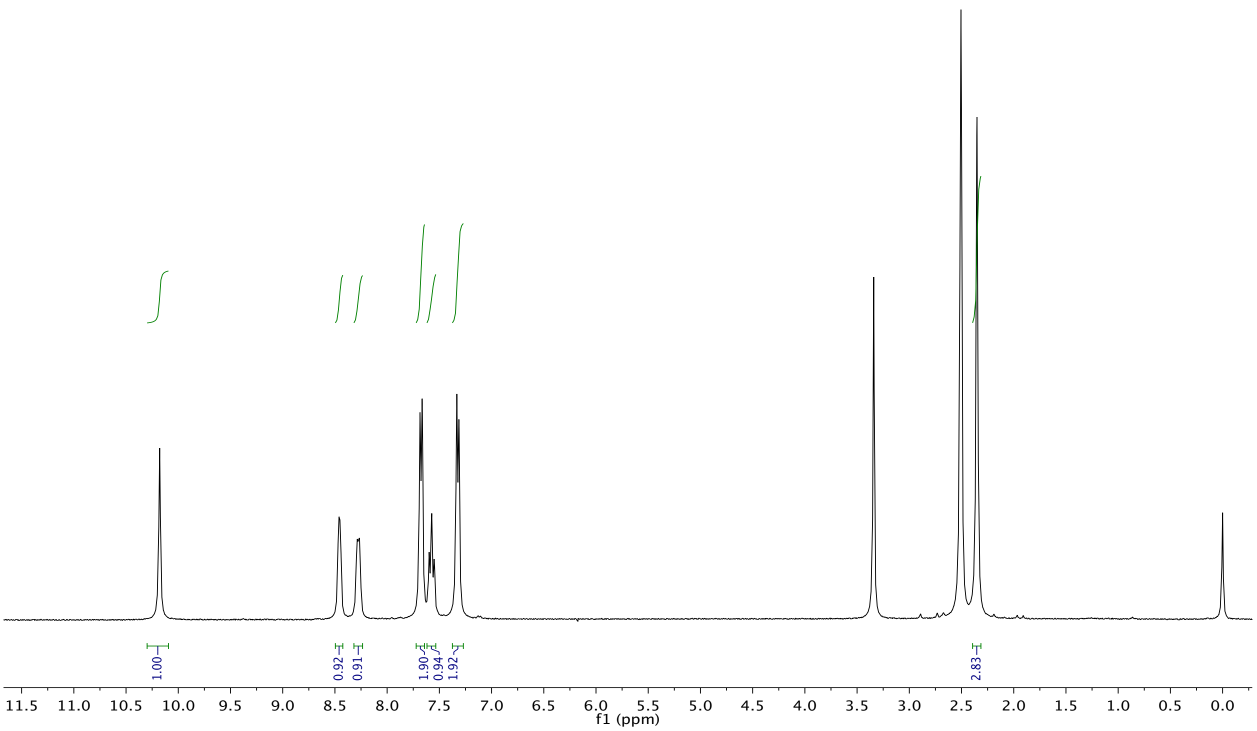

[13]C Spectrum of (4-cyanophenyl)hydrazone **9k**

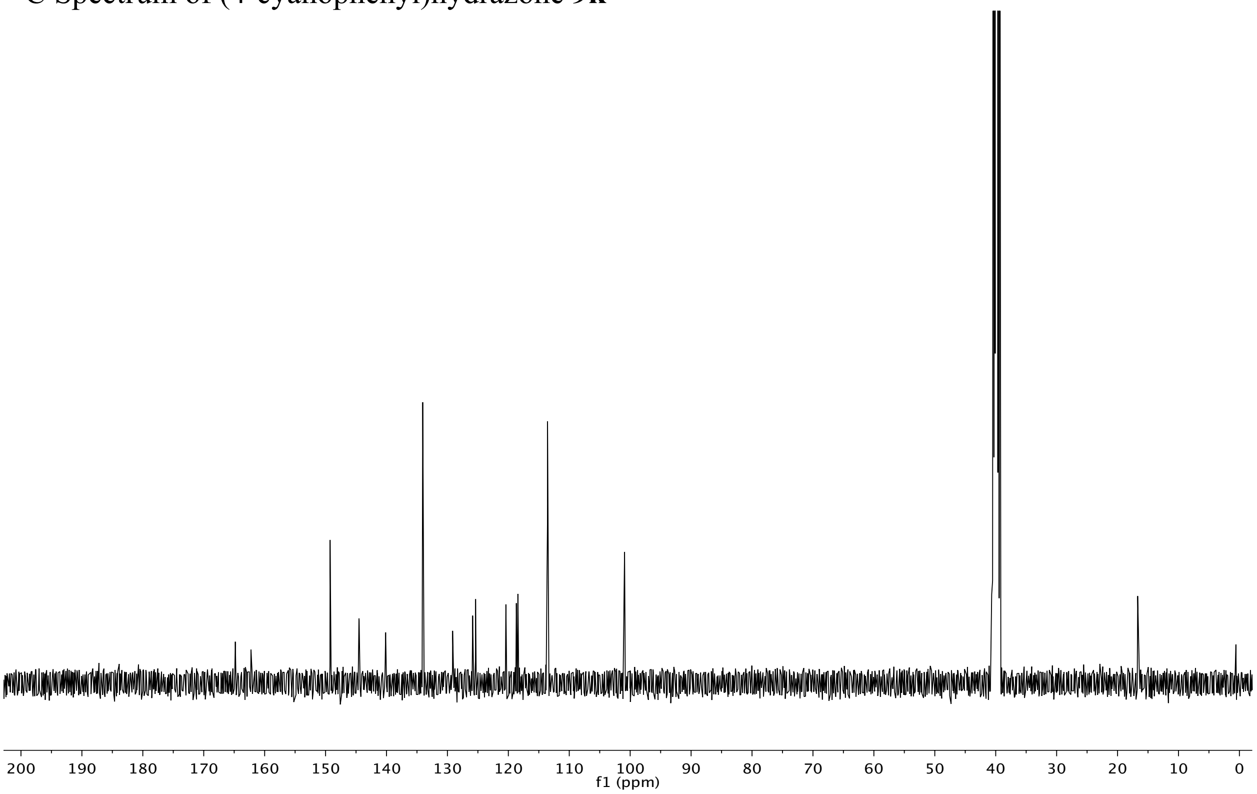

^{13}C Spectrum of (4-sulfonamidophenyl)hydrazone **9l**

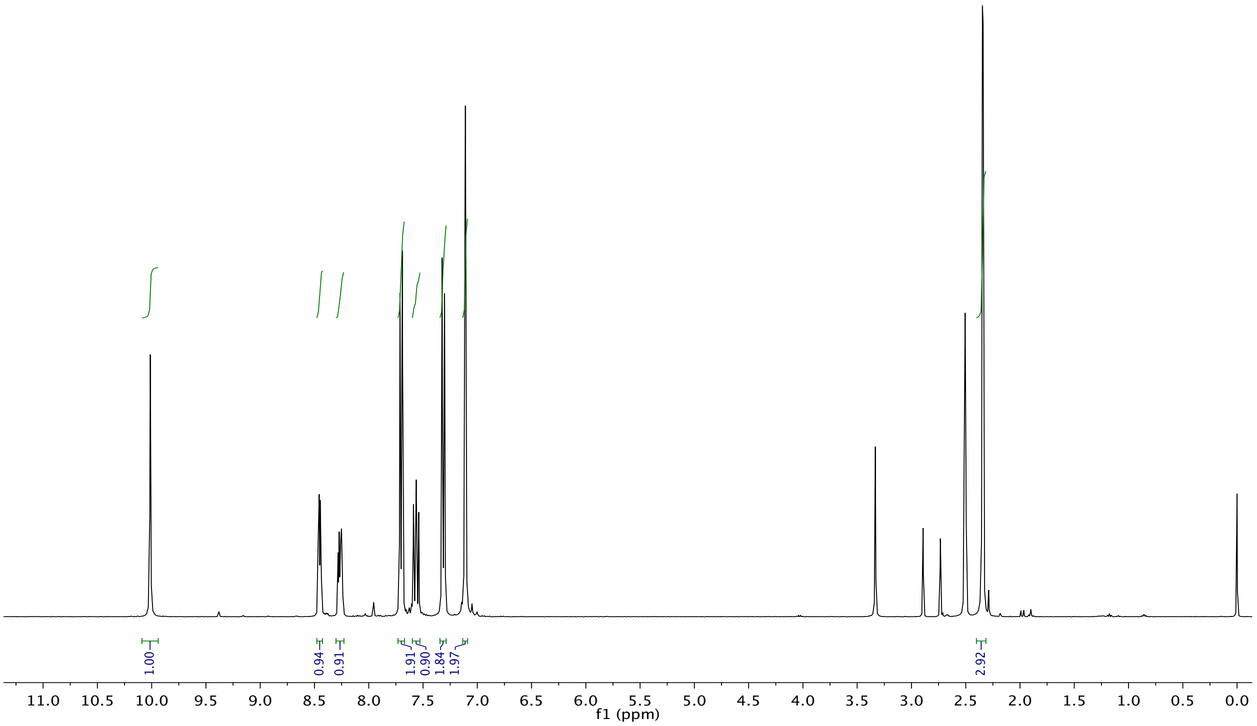

^{13}C Spectrum of (4-sulfonamidophenyl)hydrazone **9l**

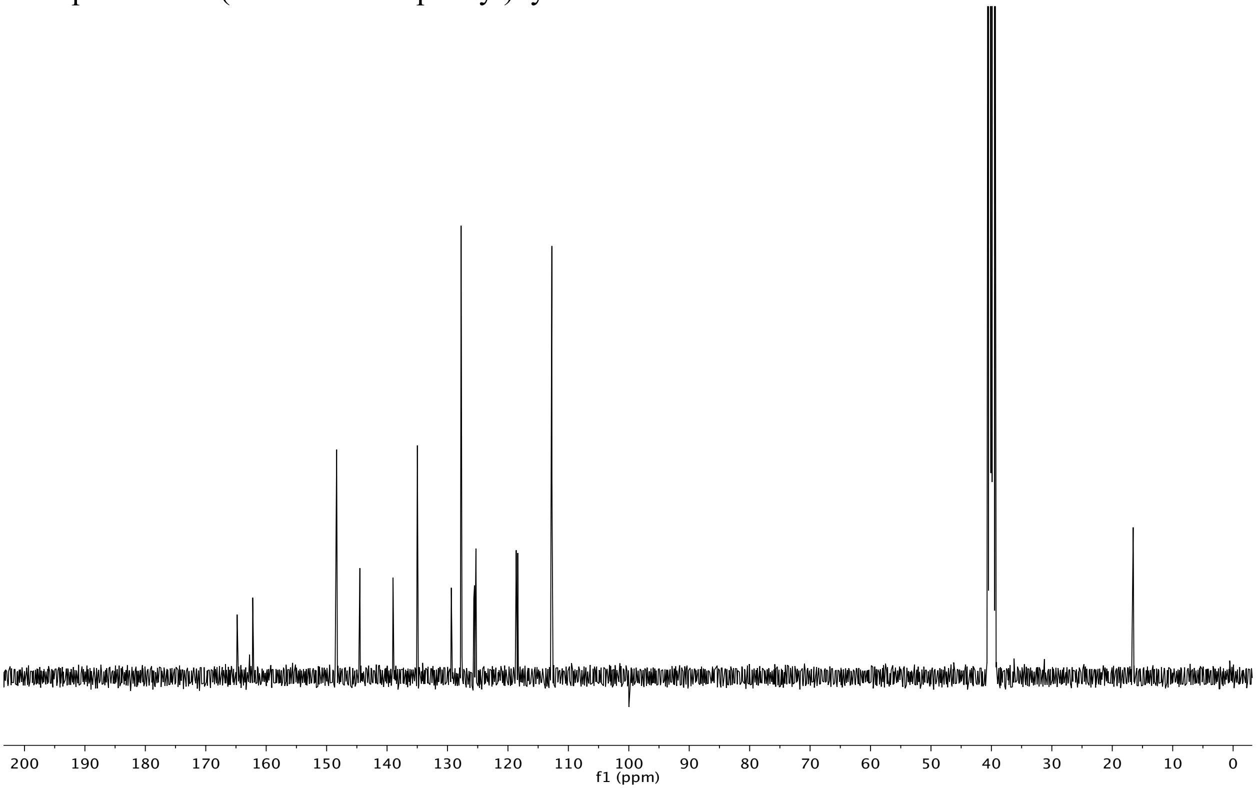

^{13}C Spectrum of (4-carboxyphenyl)hydrazone **9m**

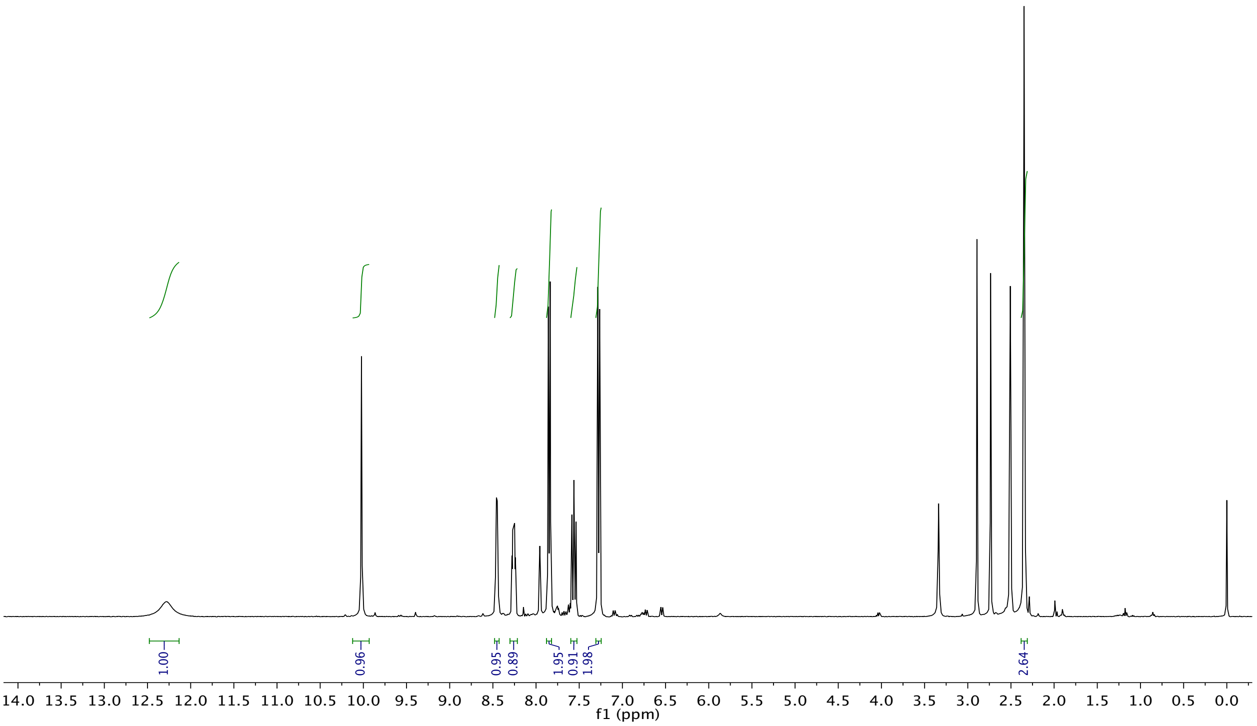

^{13}C Spectrum of (4-carboxyphenyl)hydrazone **9m**

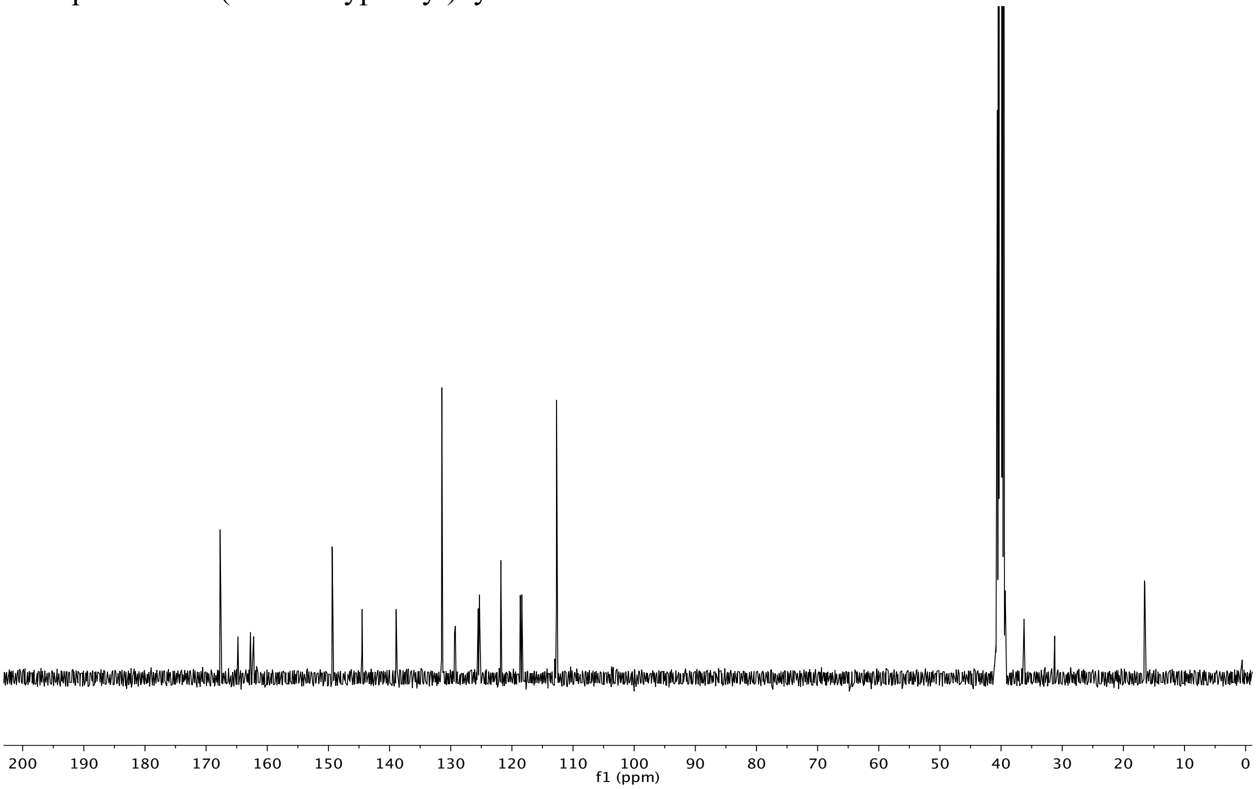

^{1}H Spectra of phenylhydrazone **10a**

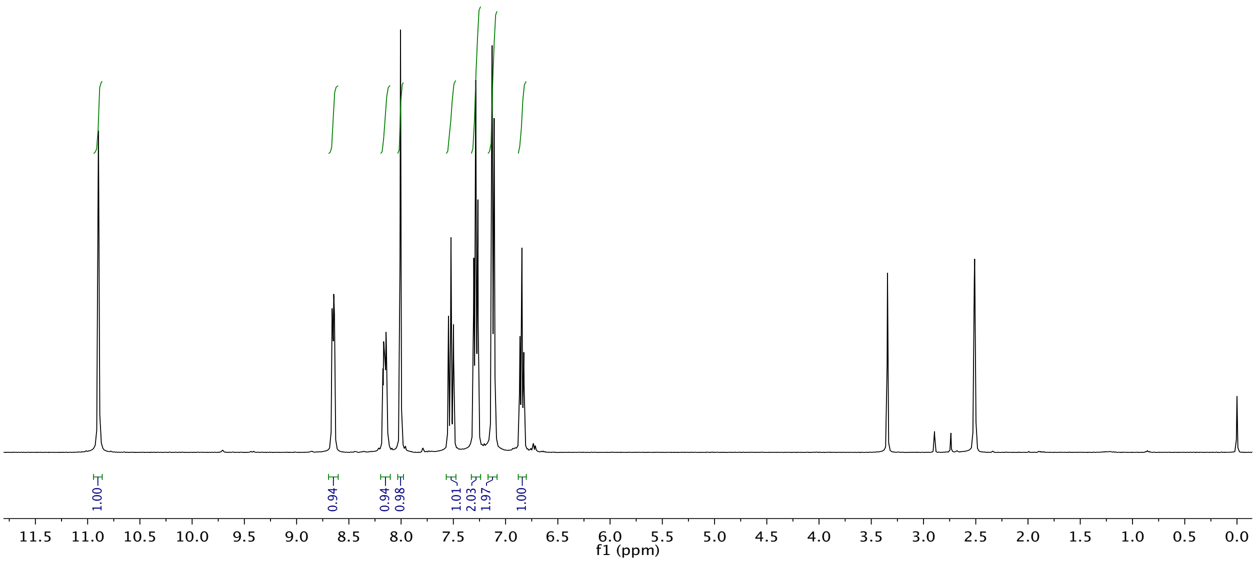

^{13}C Spectra of phenylhydrazone **10a**

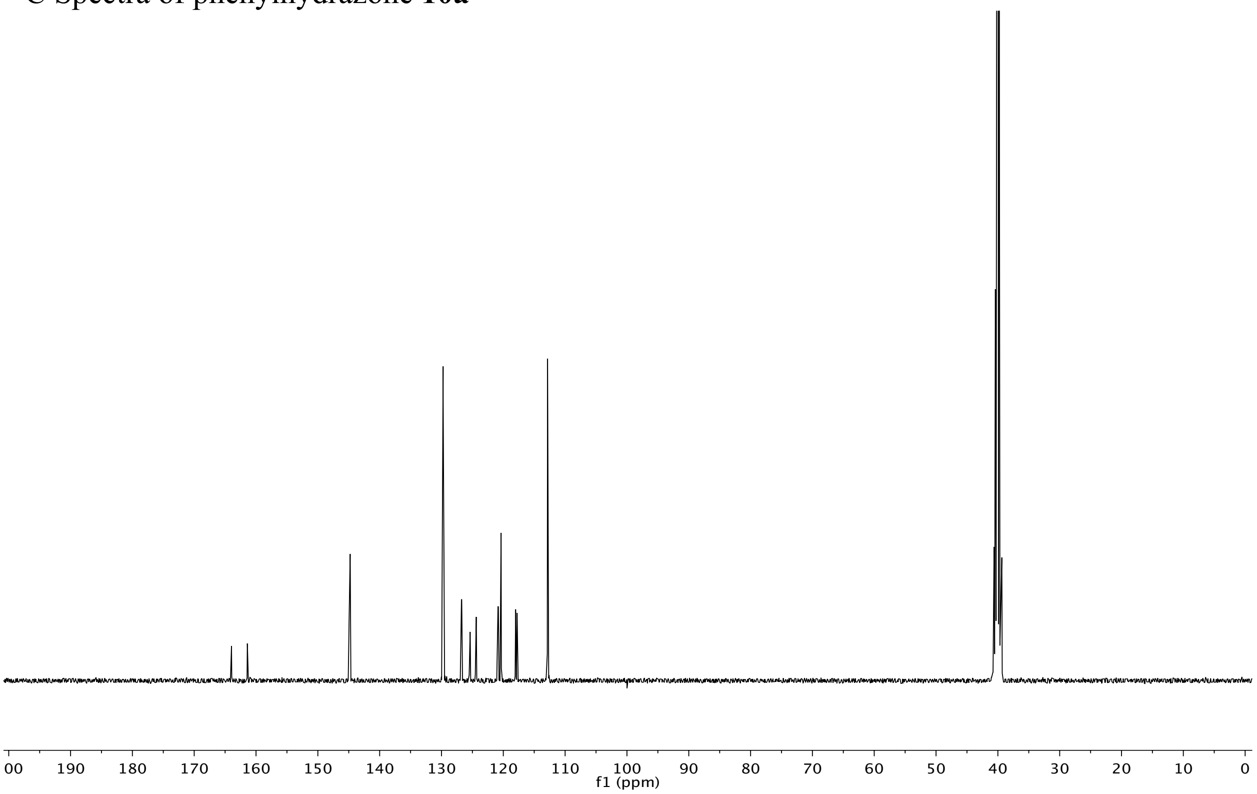

^{13}C Spectrum of (2-methoxyphenyl)hydrazone **10b**

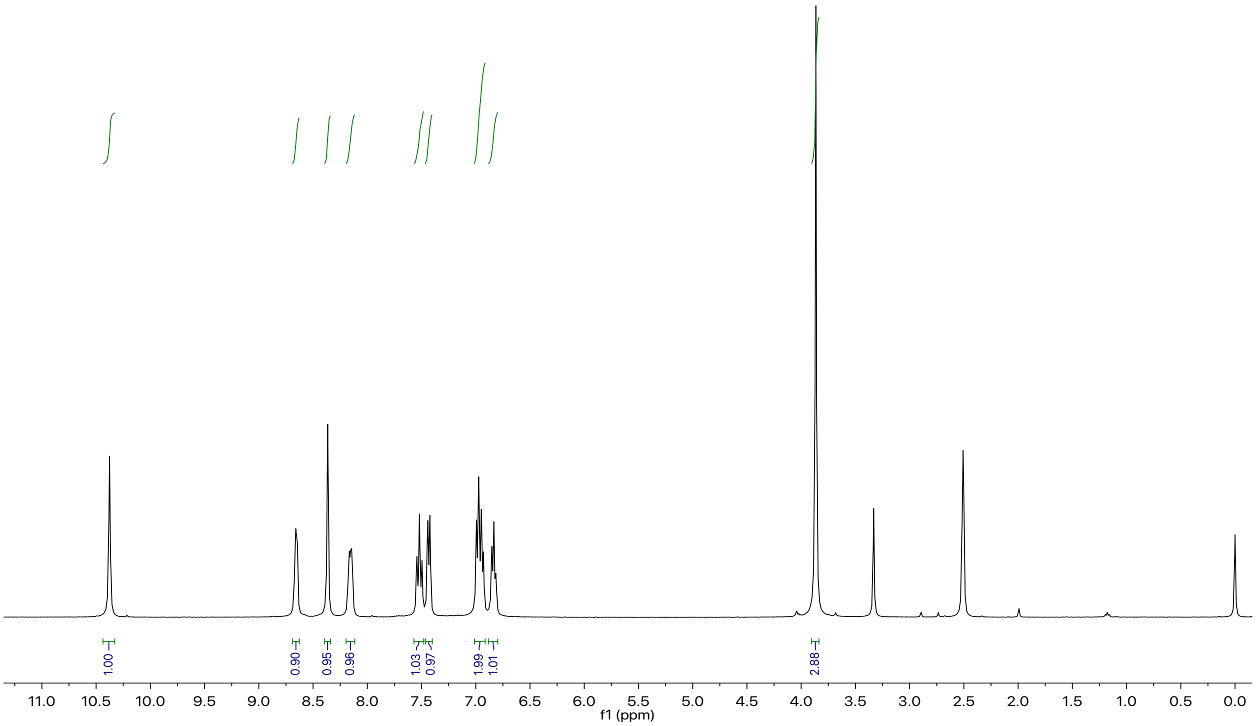

^{13}C Spectrum of (2-methoxyphenyl)hydrazone **10b**

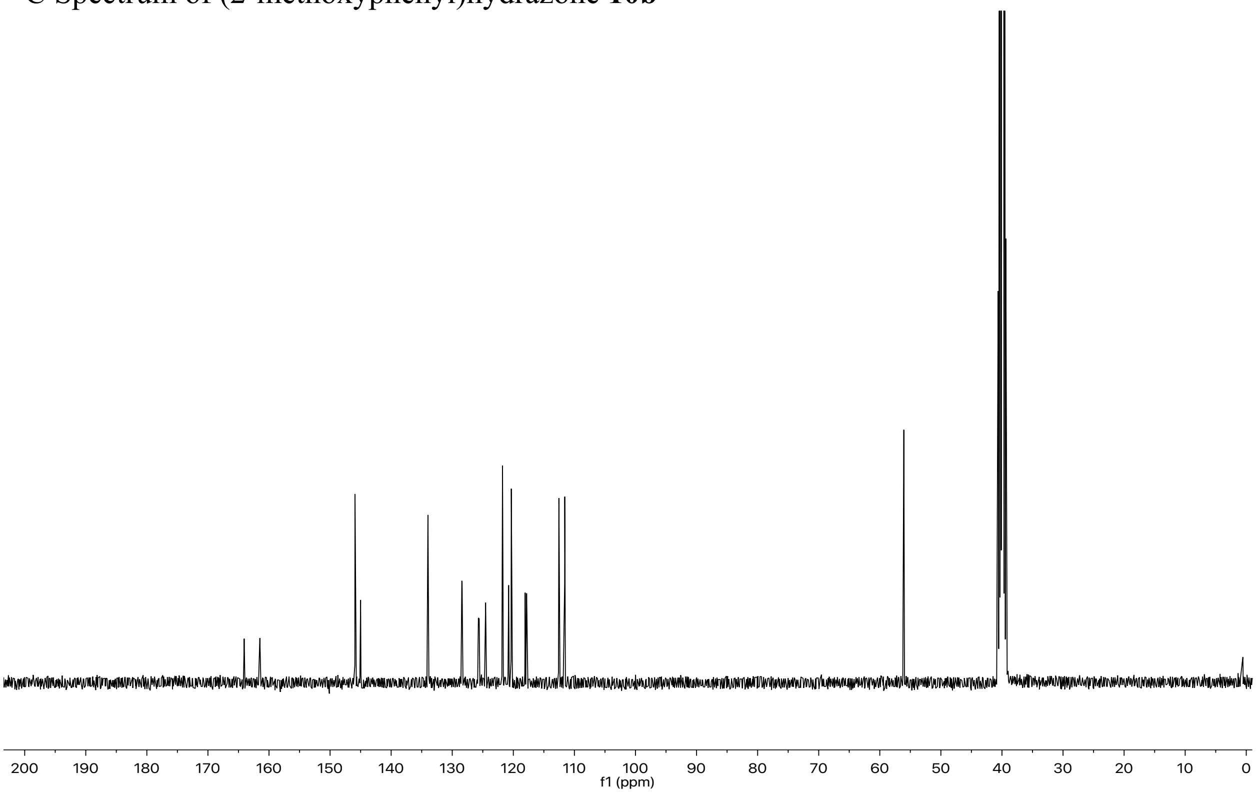

^{1}H Spectrum of (3-methoxyphenyl)hydrazone **10c**

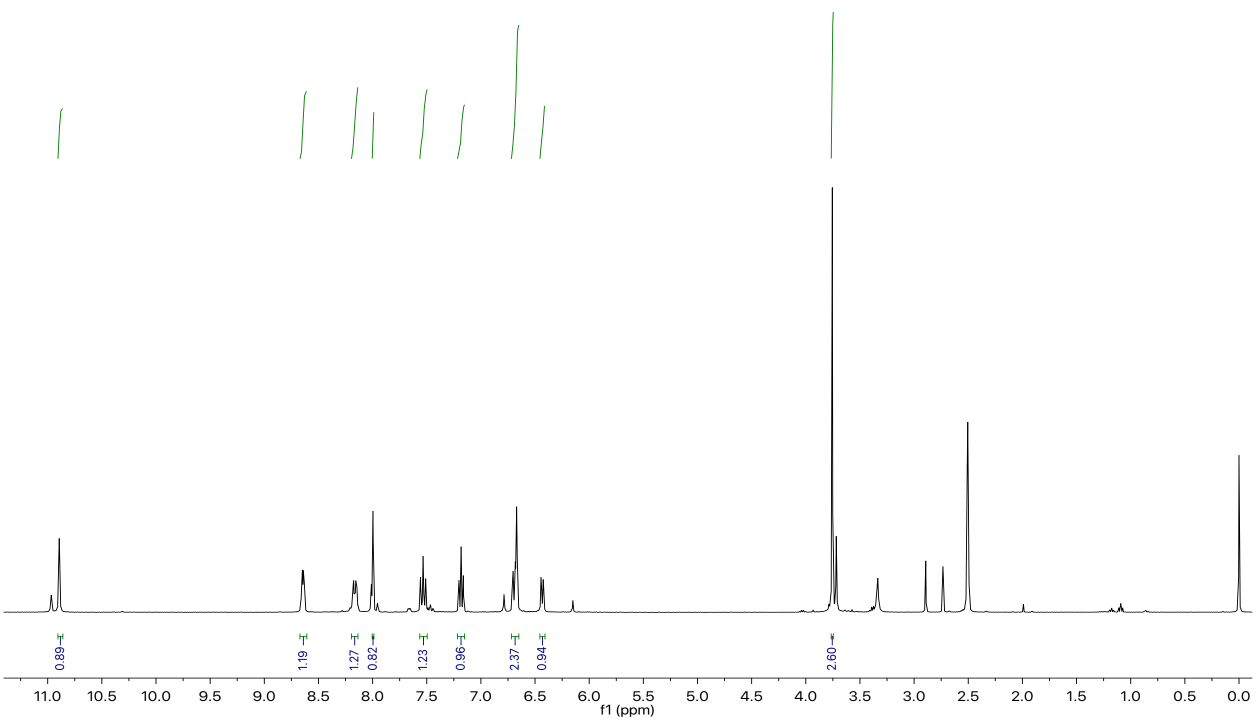

^{13}C Spectrum of (3-methoxyphenyl)hydrazone **10c**

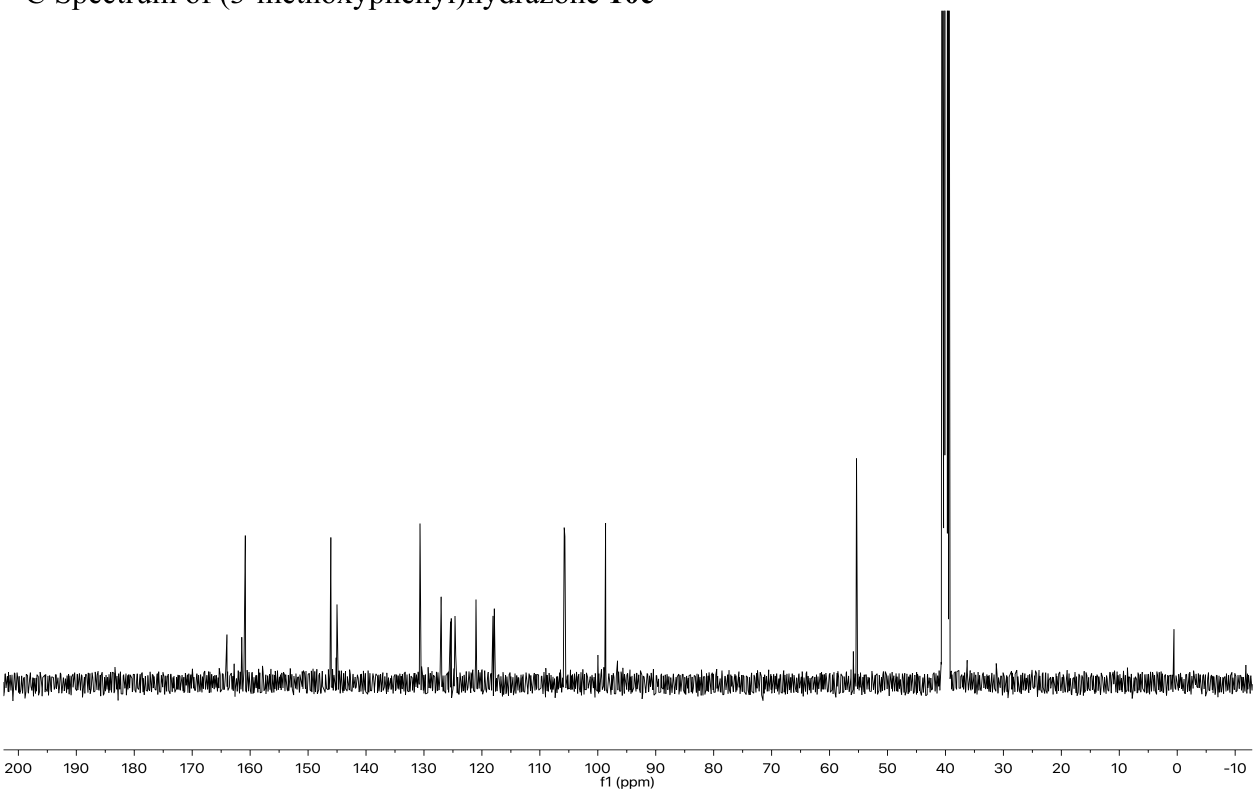

^{1}H Spectrum of (4-methoxyphenyl)hydrazone **10d**

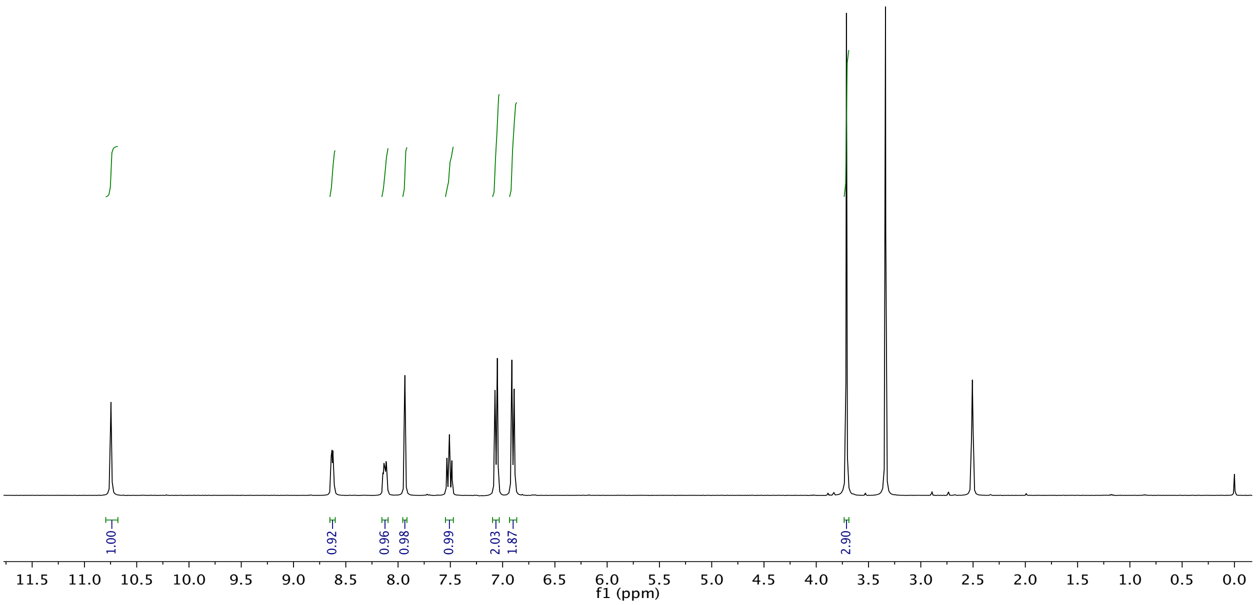

^{13}C Spectrum of (4-methoxyphenyl)hydrazone **10d**

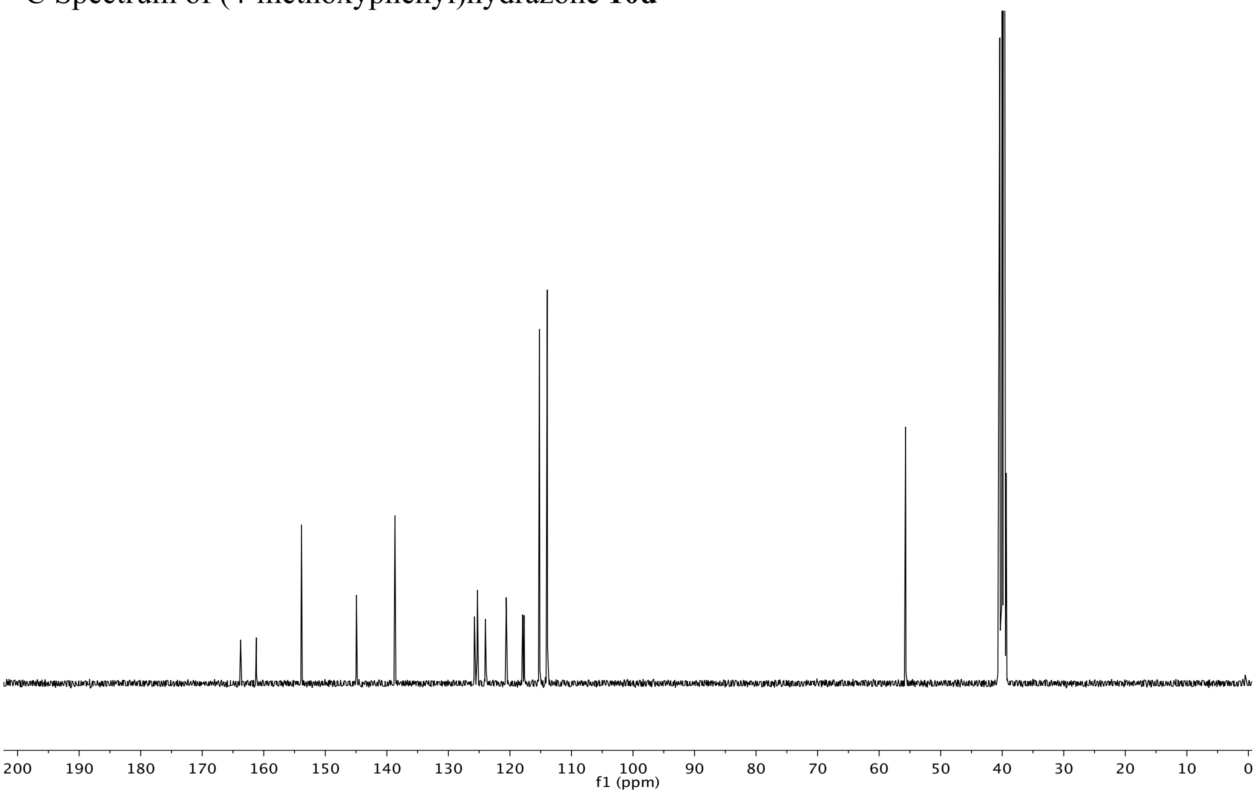

^{1}H Spectrum of (4-bromophenyl)hydrazone **10e**

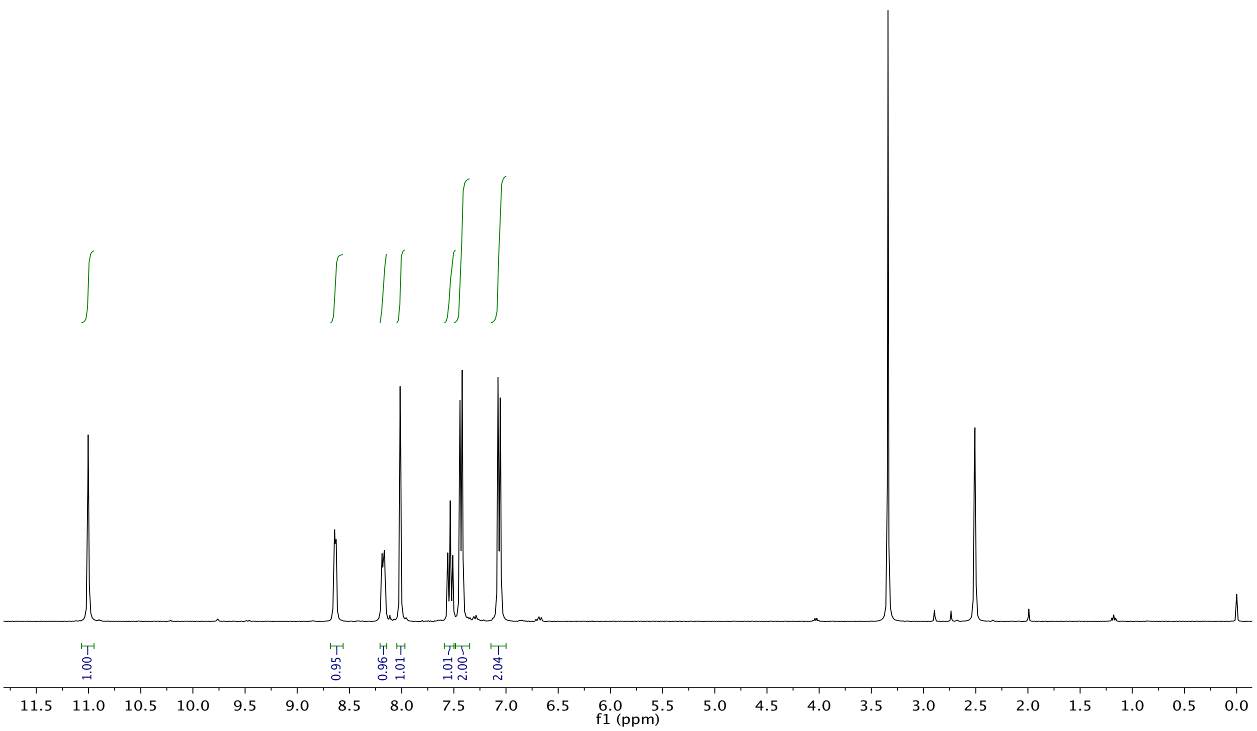

^{13}C Spectrum of (4-bromophenyl)hydrazone **10e**

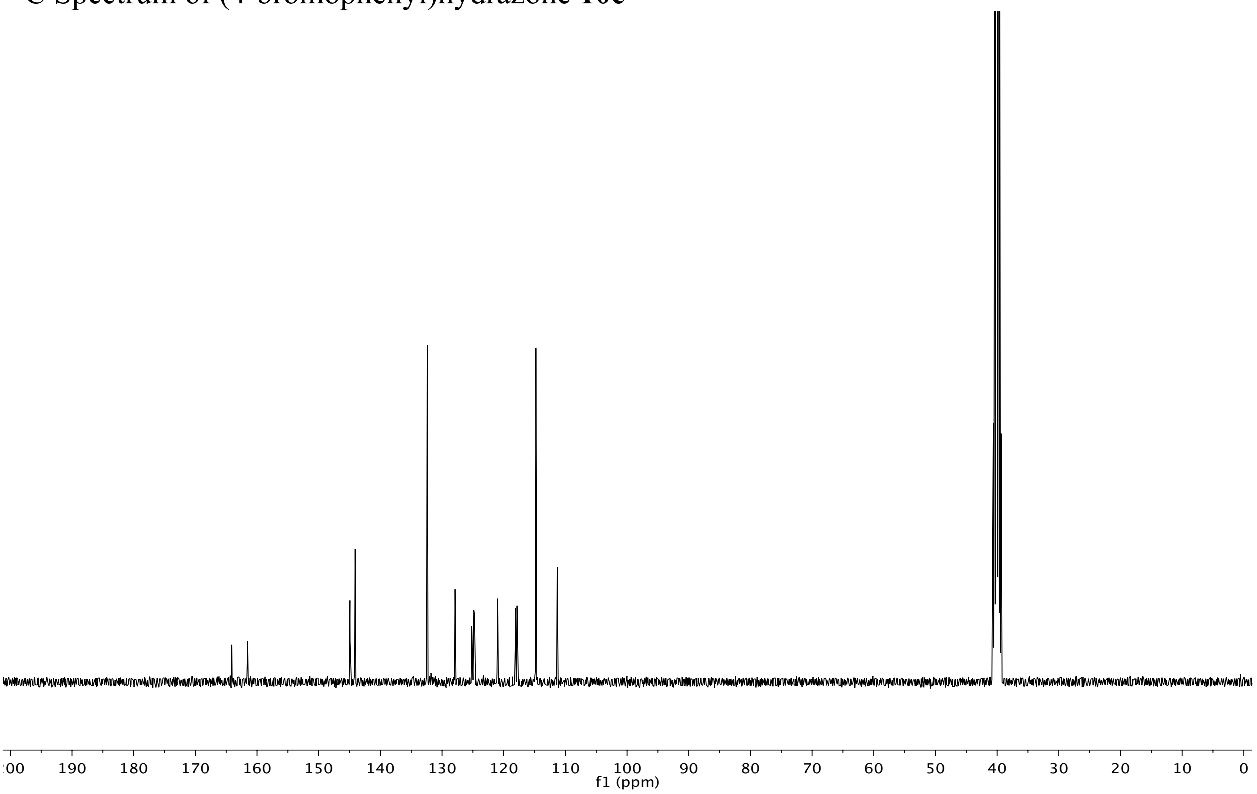

^{1}H Spectrum of (3-chlorophenyl)hydrazone **10f**

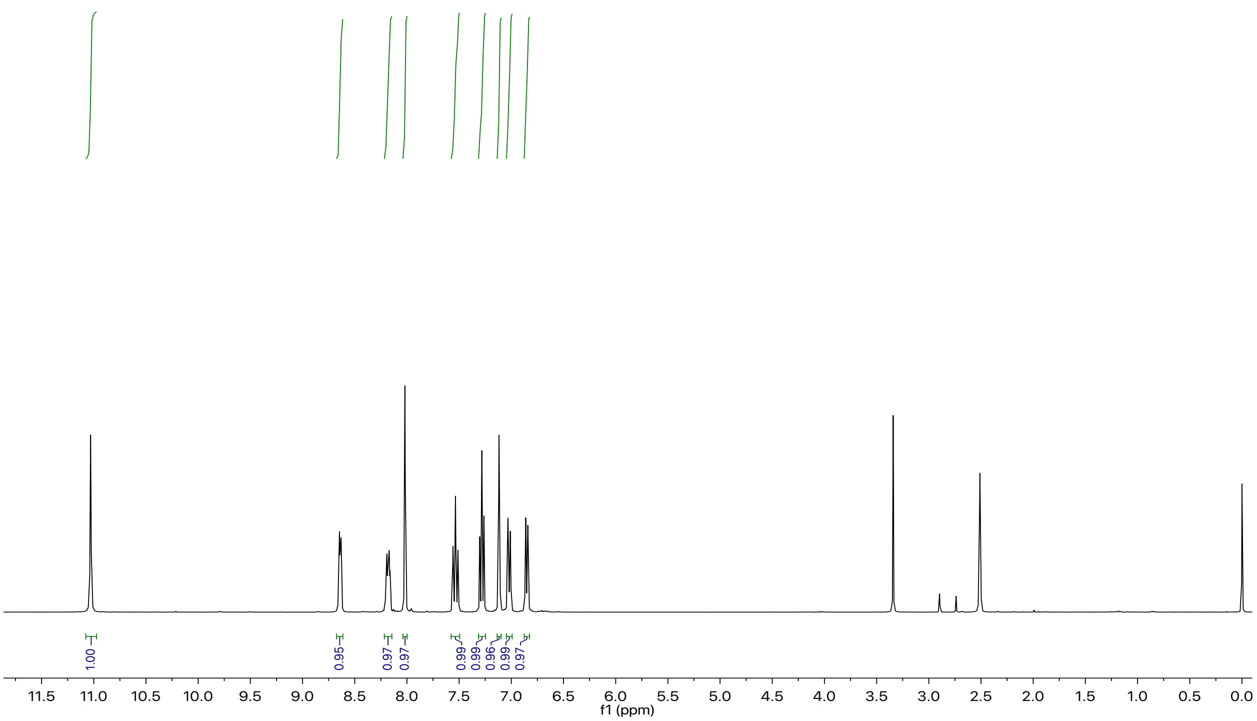

^{13}C Spectrum of (3-chlorophenyl)hydrazone **10f**

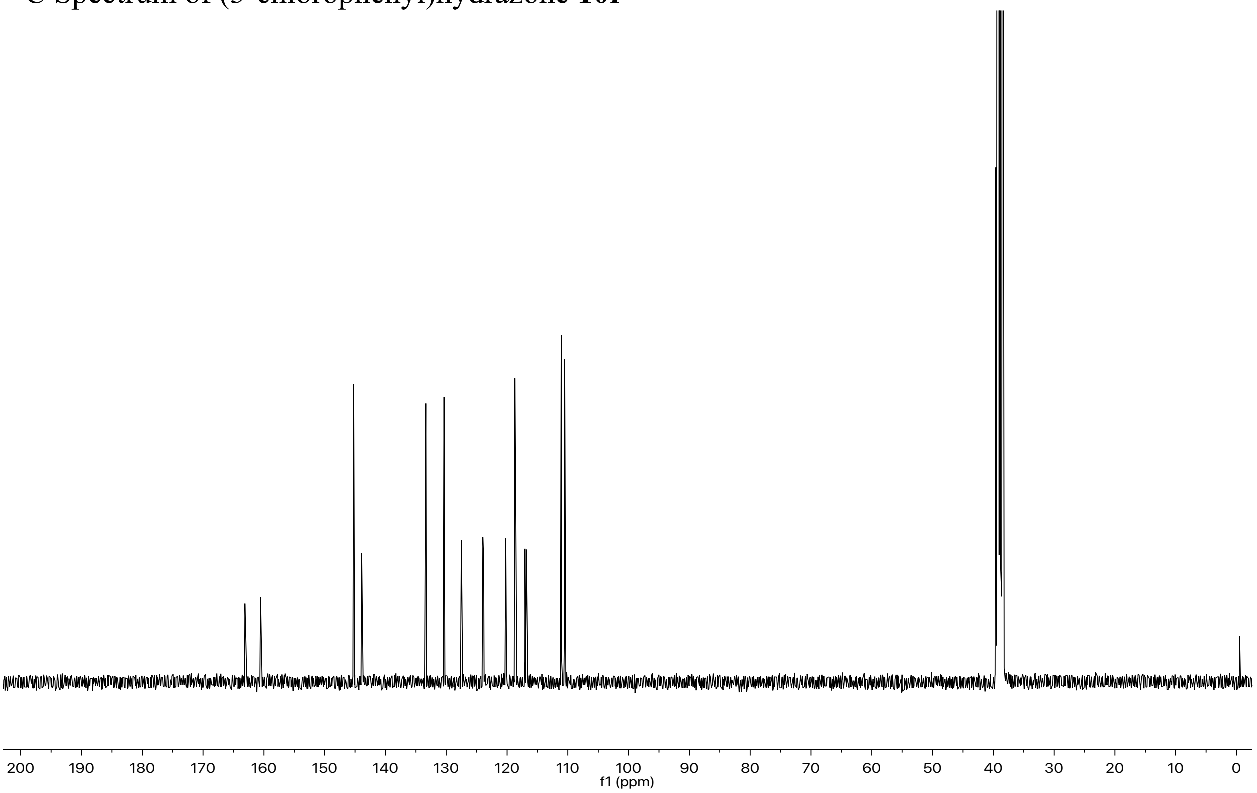

^{1}H Spectrum of (4-chlorophenyl)hydrazone **10g**

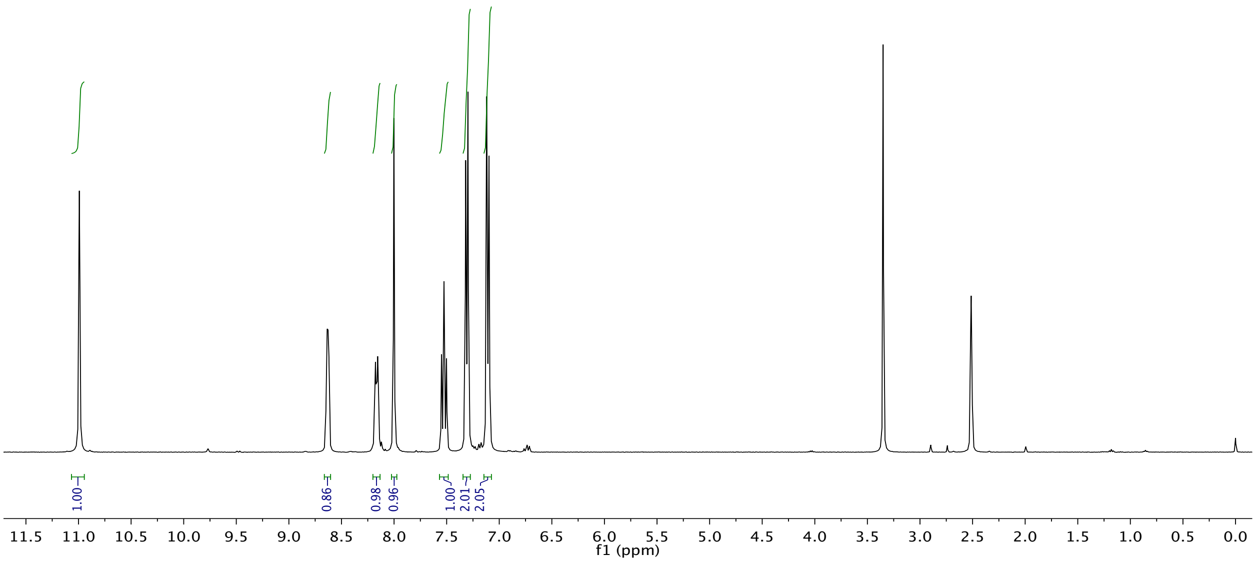

^{13}C Spectrum of (4-chlorophenyl)hydrazone **10g**

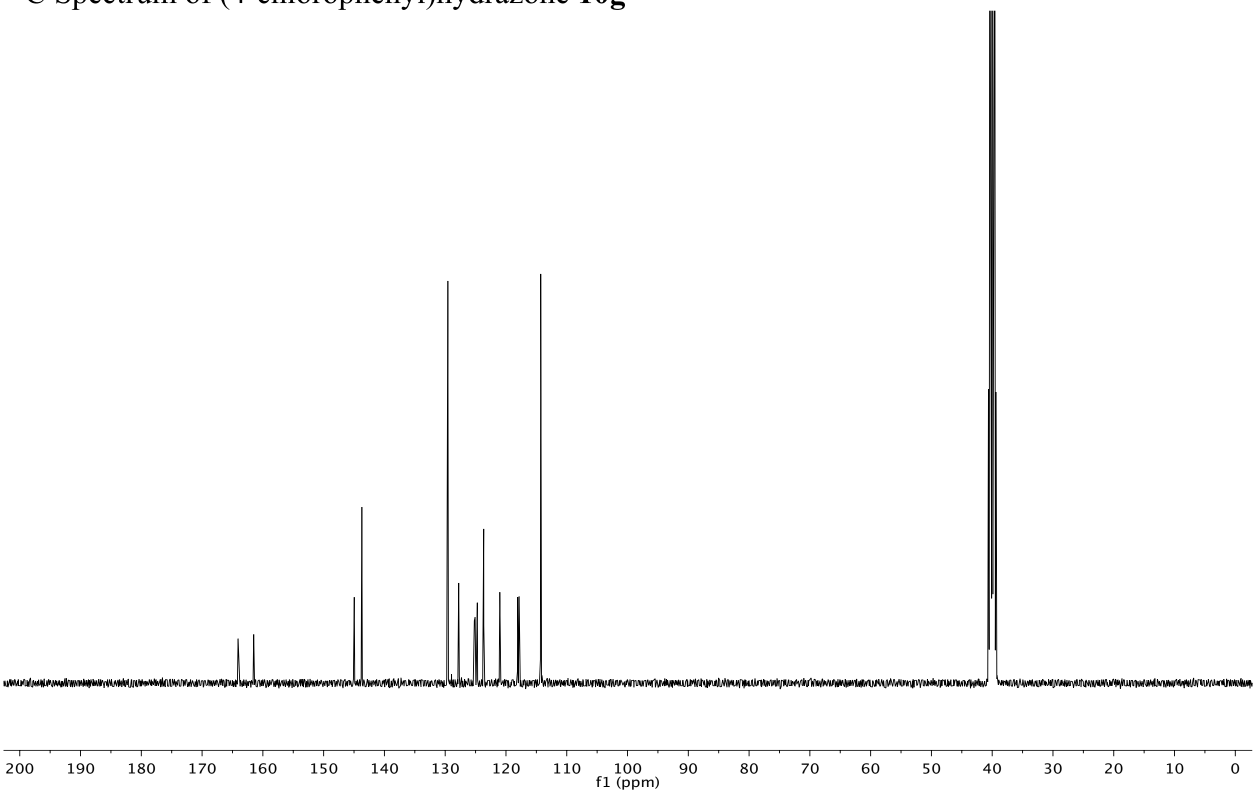

[1]H Spectrum of (2,4-dichlorophenyl)hydrazone **10h**

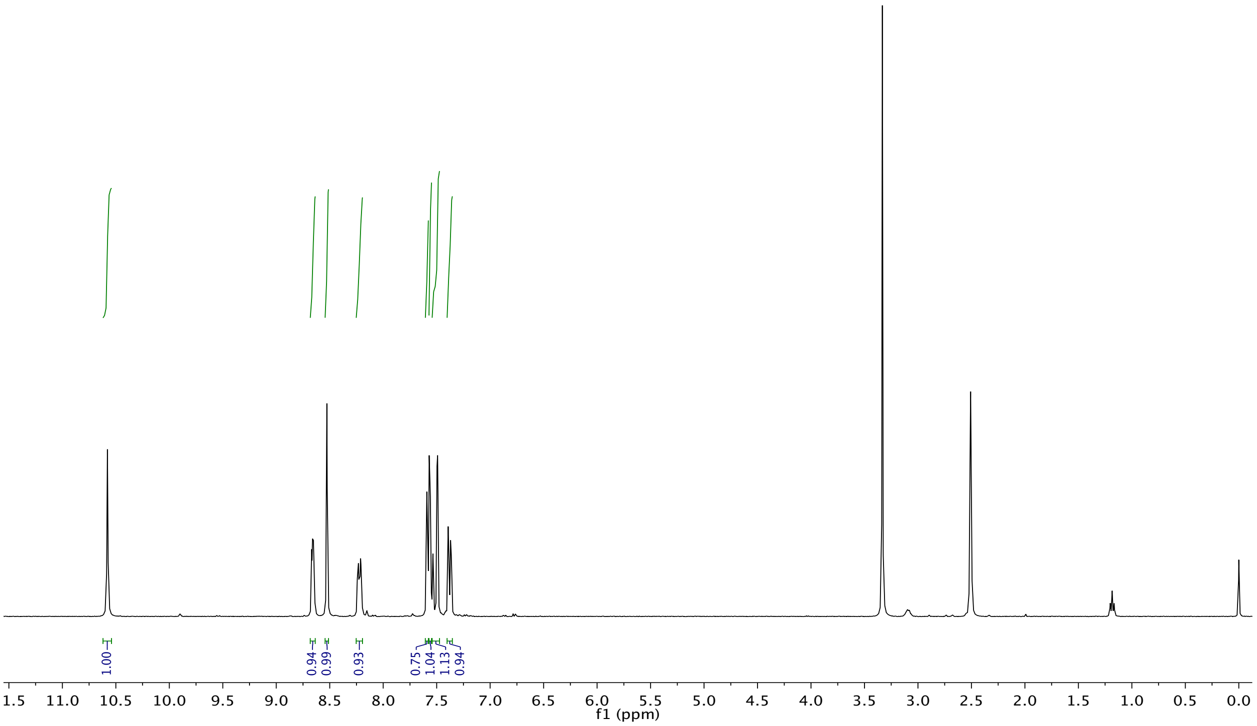

[13]C Spectrum of (2,4-dichlorophenyl)hydrazone **10h**

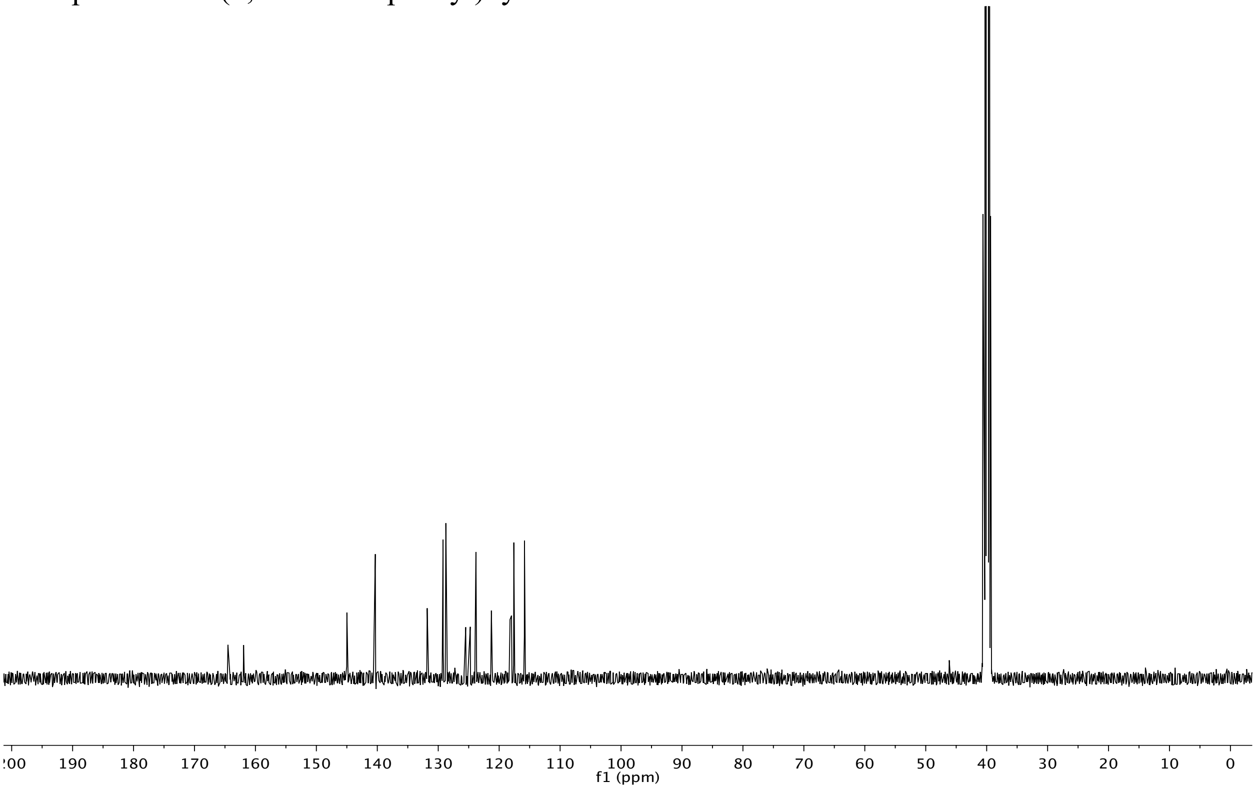

^{1}H Spectrum of (4-(trifluoromethyl)phenyl)hydrazone **10i**

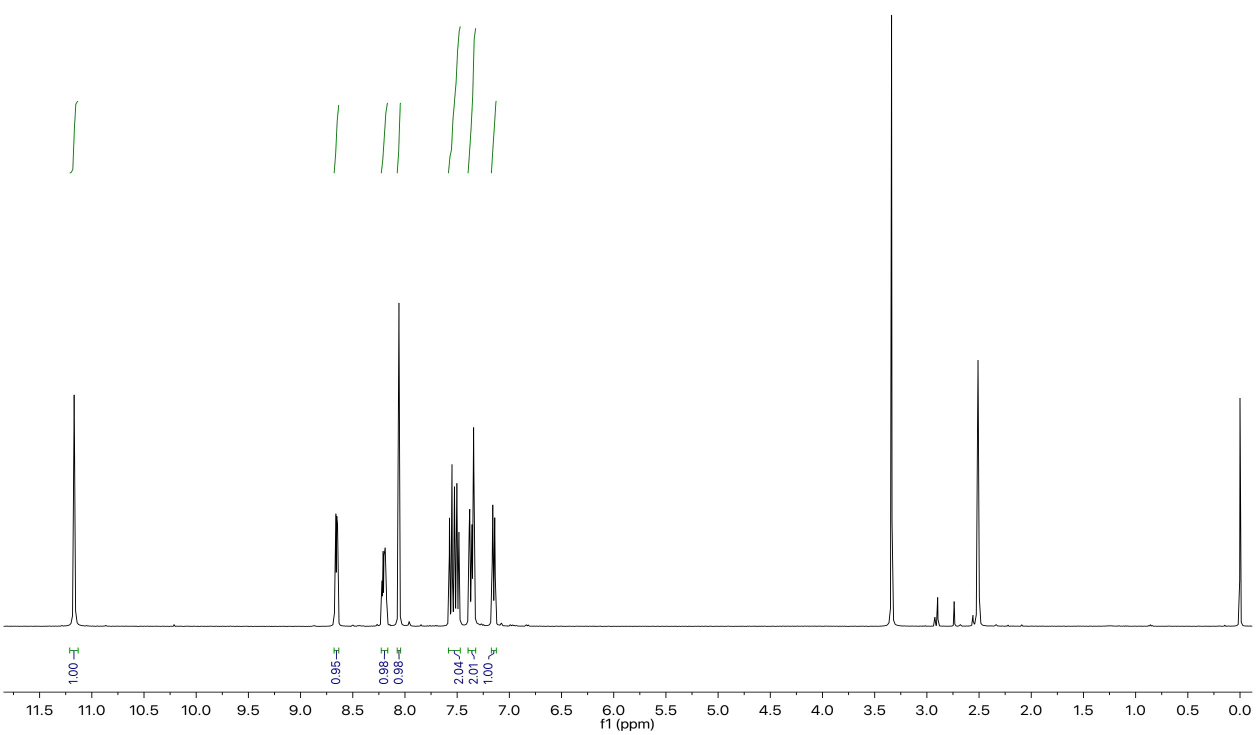

^{13}C Spectrum of (3-(trifluoromethyl)phenyl)hydrazone **10i**

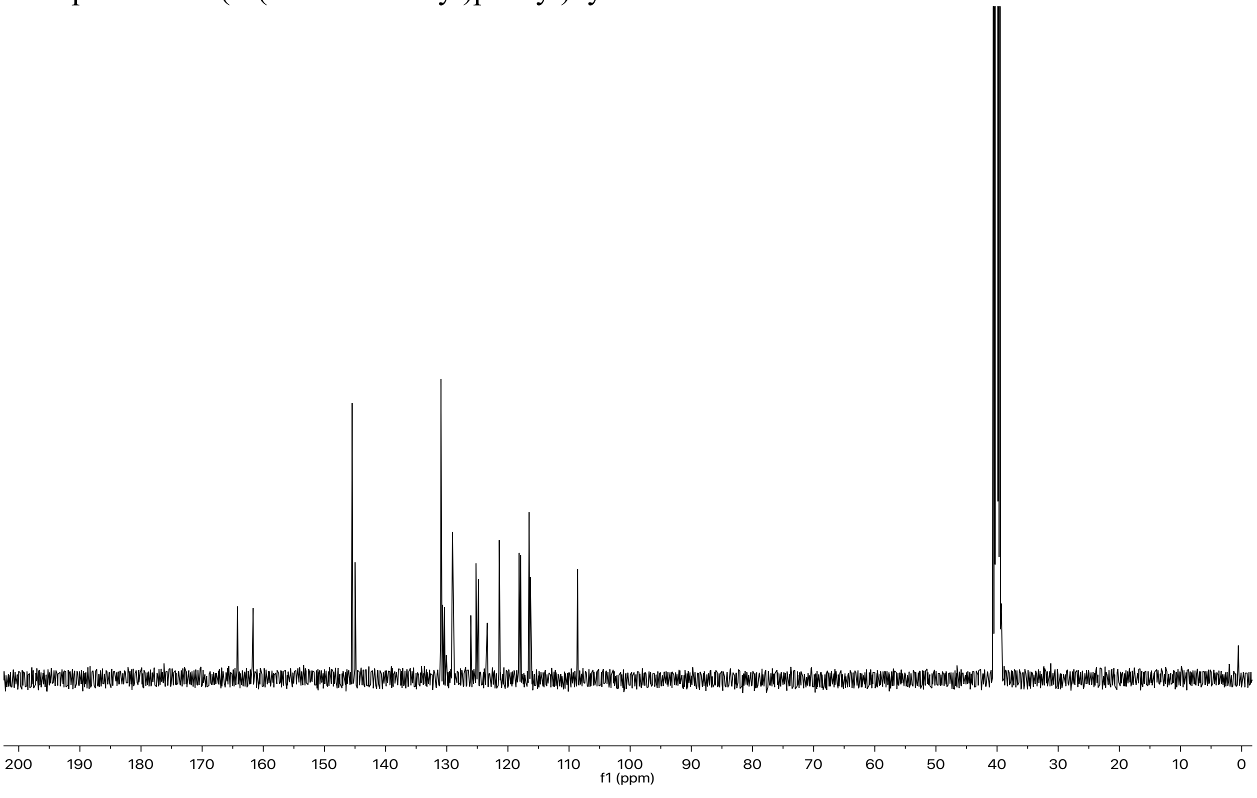

^{1}H Spectrum of (4-(trifluoromethyl)phenyl)hydrazone **10j**

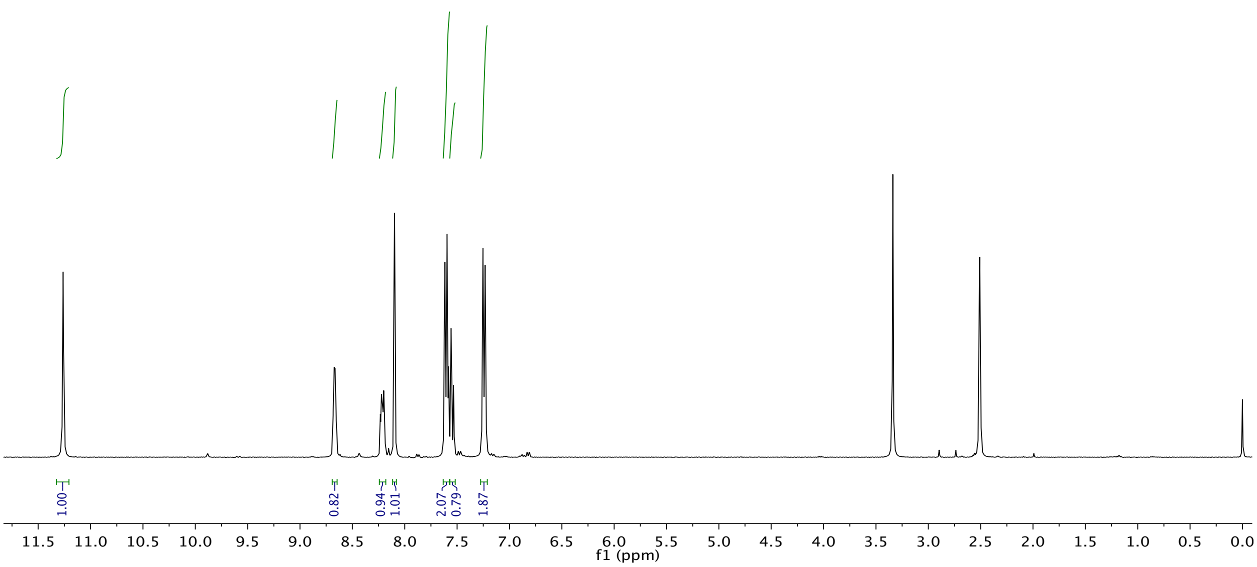

^{13}C Spectrum of (4-(trifluoromethyl)phenyl)hydrazone **10j**

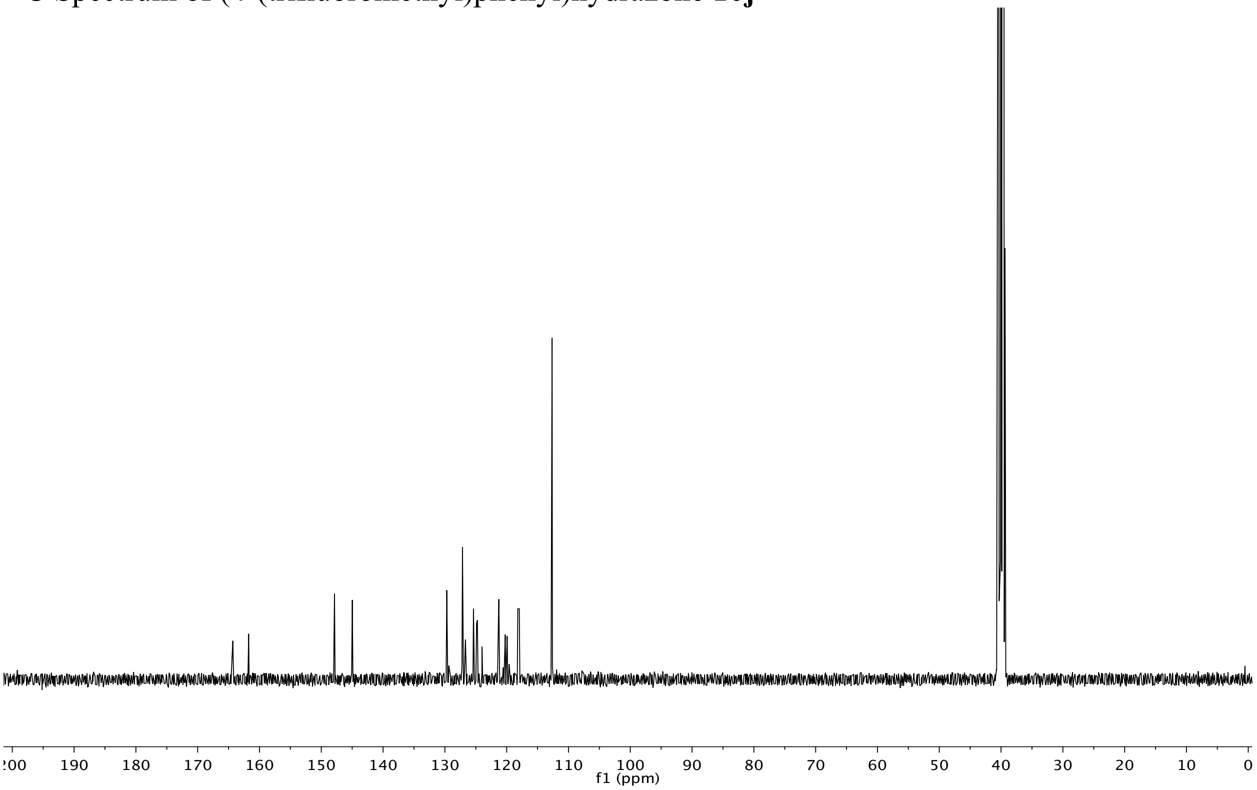

[1]H Spectrum of (4-cyanophenyl)hydrazone **10k**

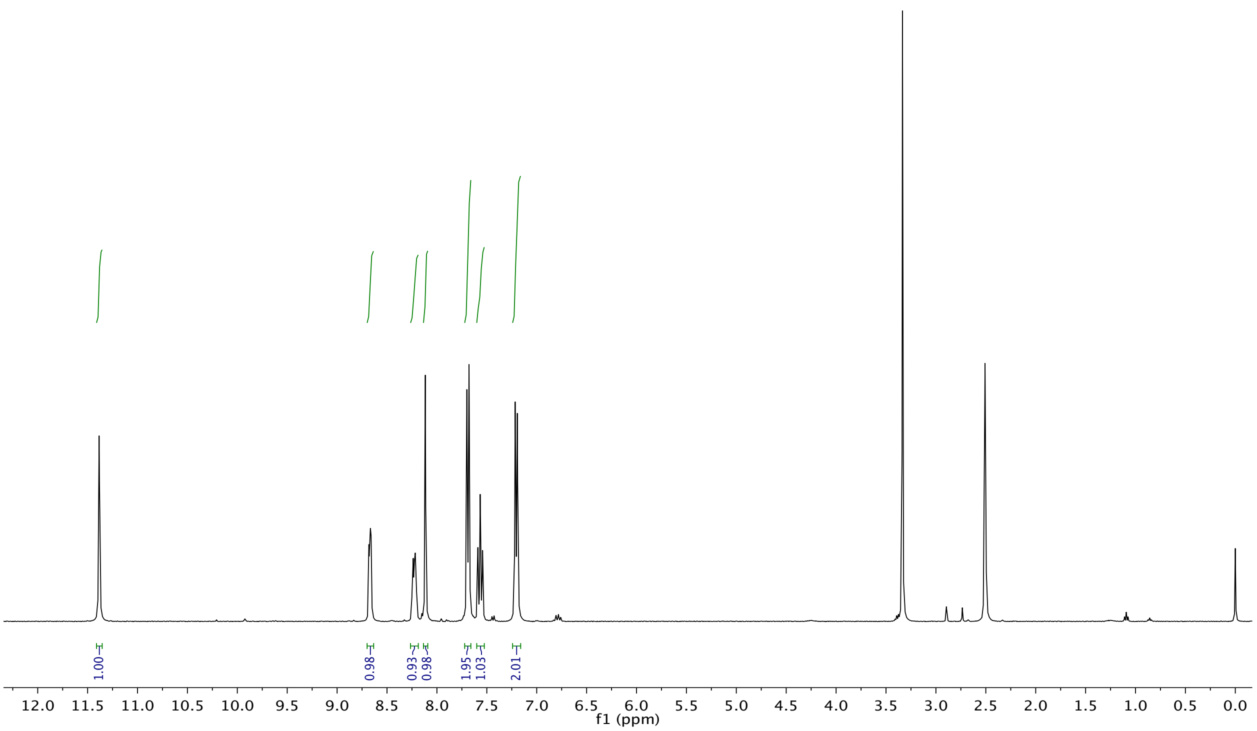

[13]C Spectrum of (4-cyanophenyl)hydrazone **10k**

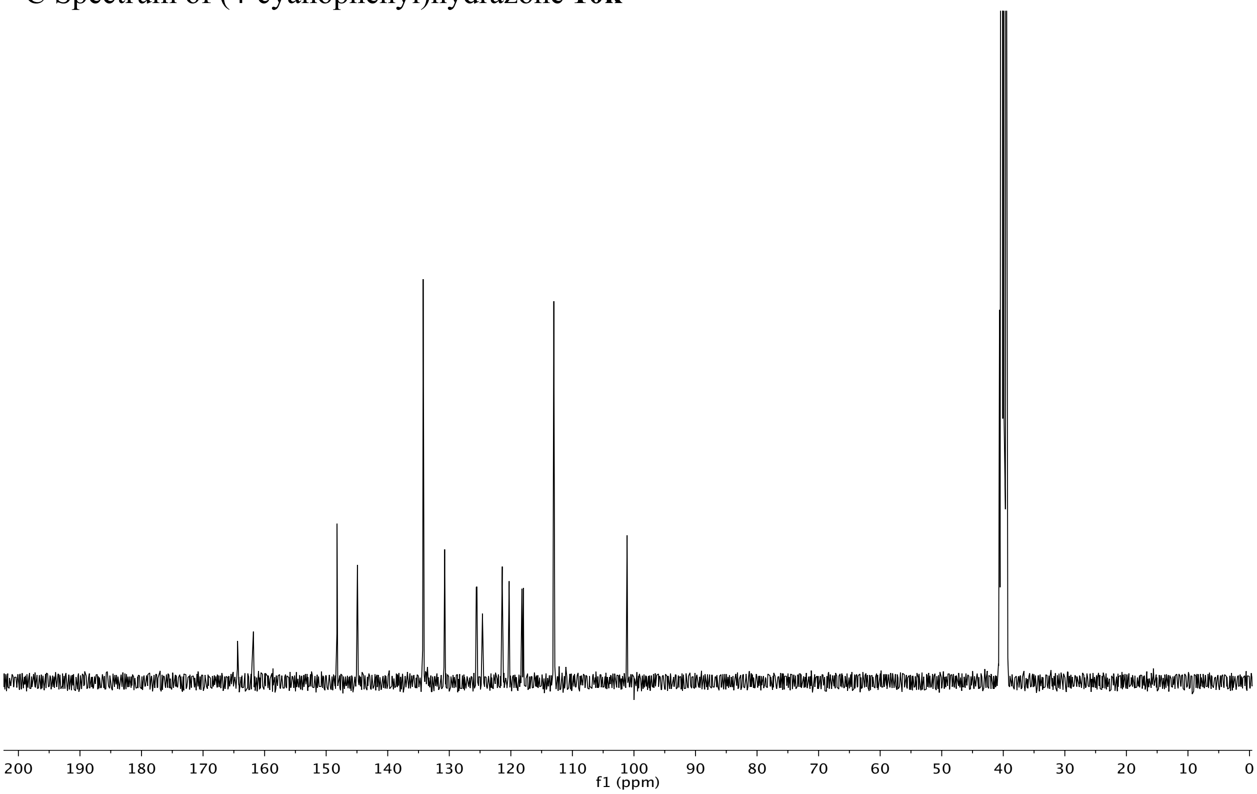

^{1}H Spectrum of (4-sulfonamidophenyl)hydrazone **10l**

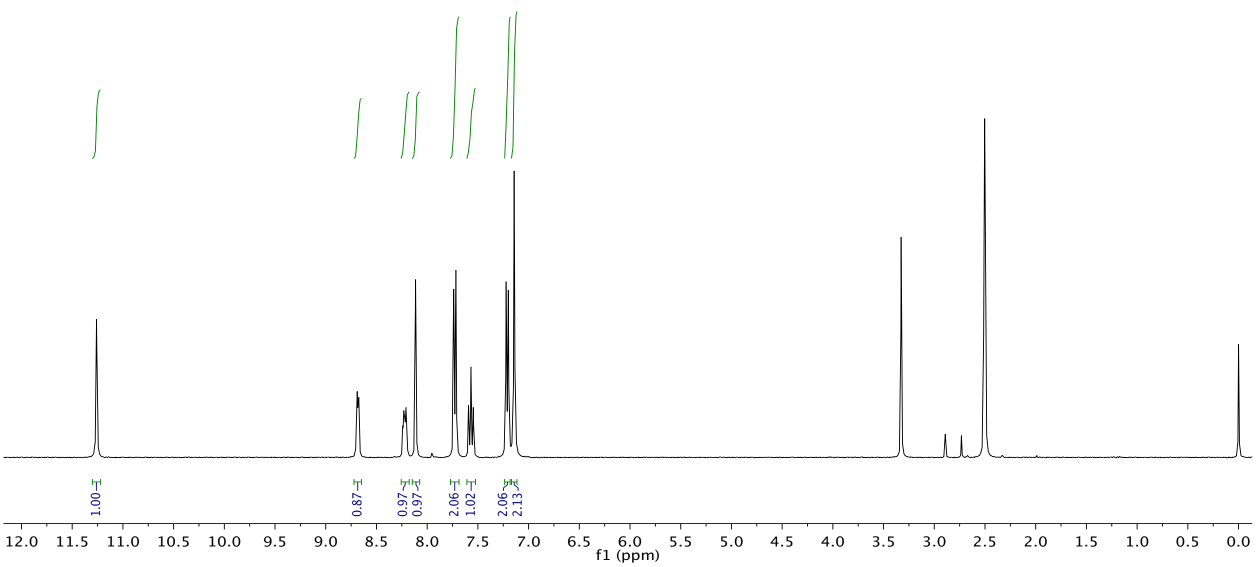

^{13}C Spectrum of (4-sulfonamidophenyl)hydrazone **10l**

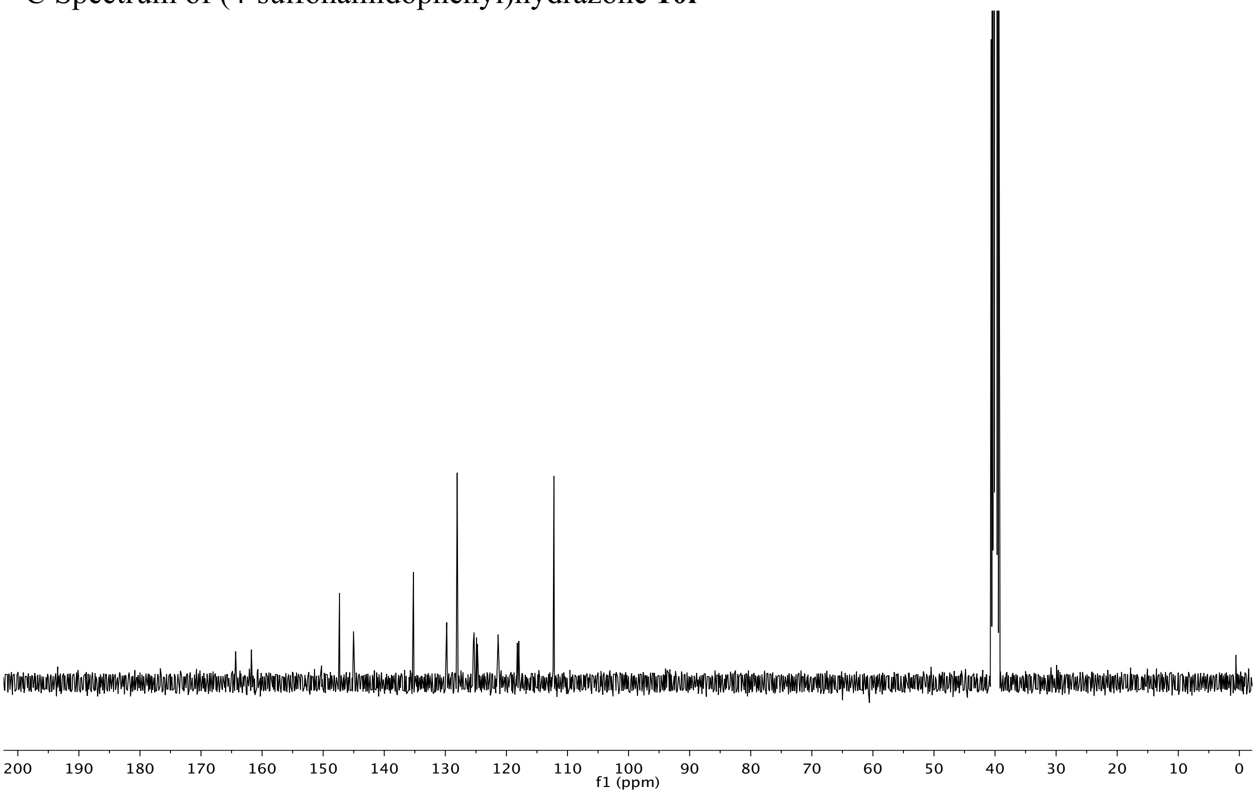

[1]H Spectrum of (4-carboxyphenyl)hydrazone **10m**

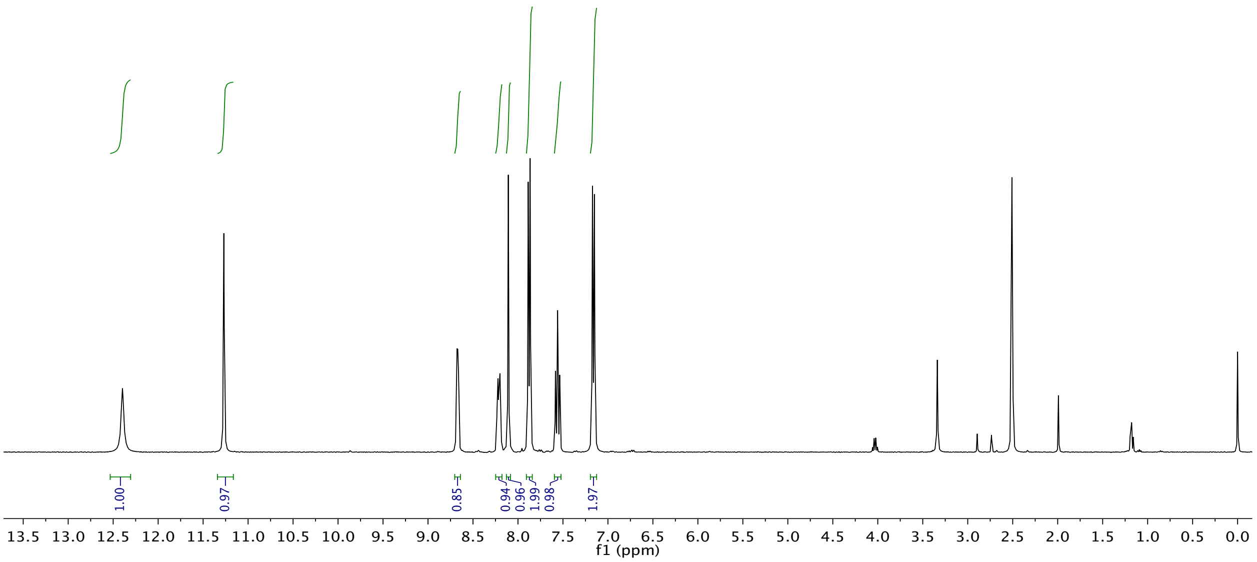

[13]C Spectrum of (4-carboxyphenyl)hydrazone **10m**

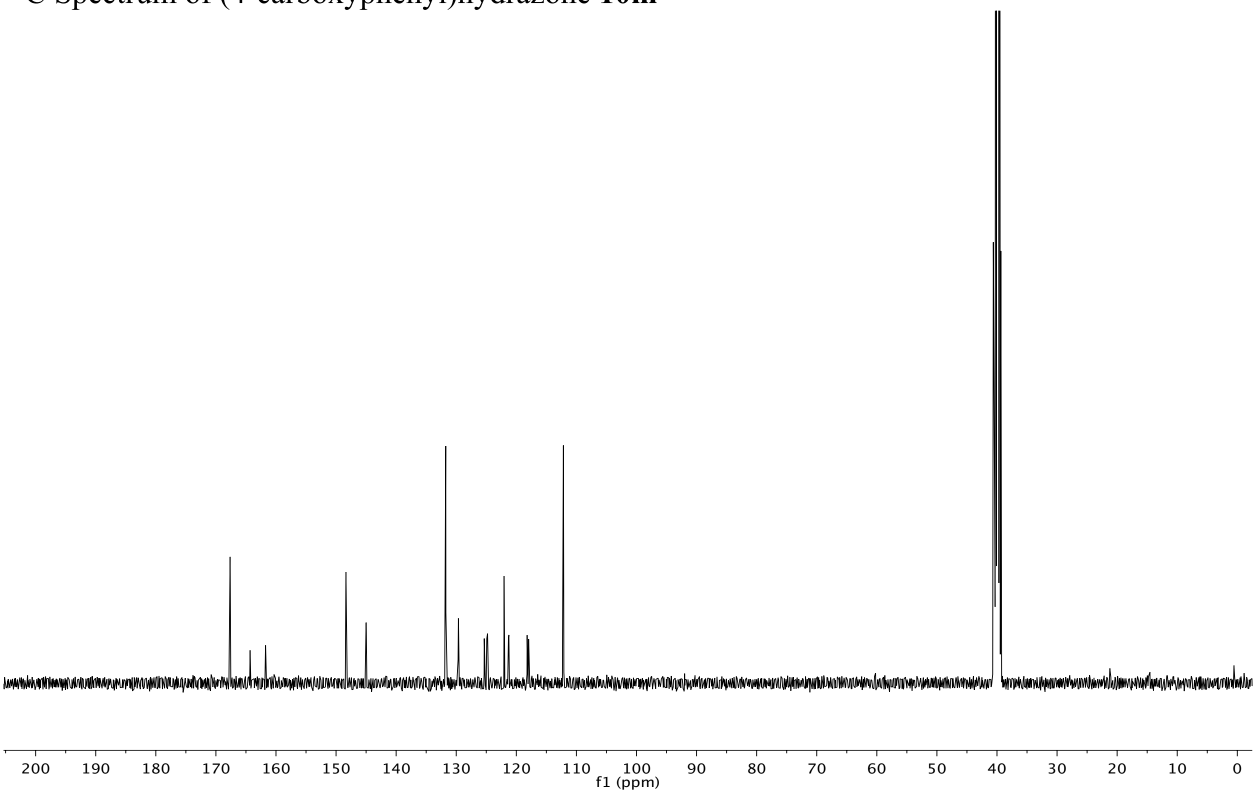

^{1}H Spectrum of 3-methyl-5-nitro-1*H*-indazole (**7**)

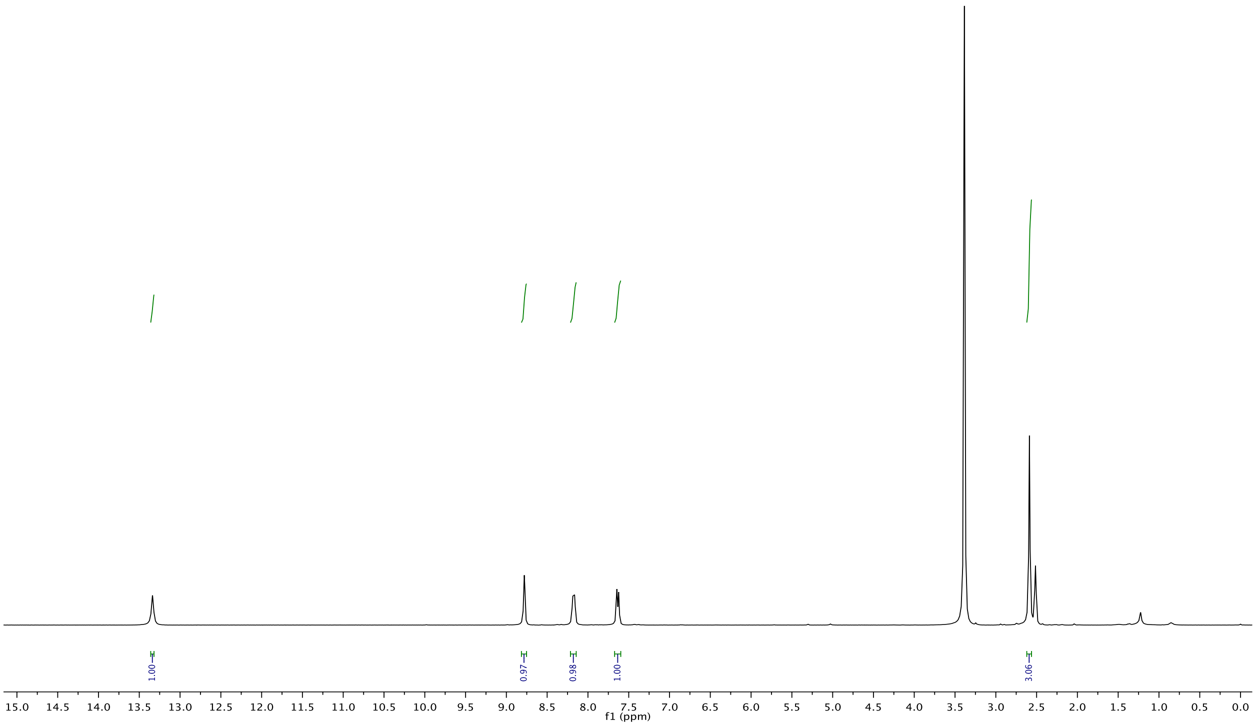

^{13}C Spectrum of 3-methyl-5-nitro-1*H*-indazole (**7**)

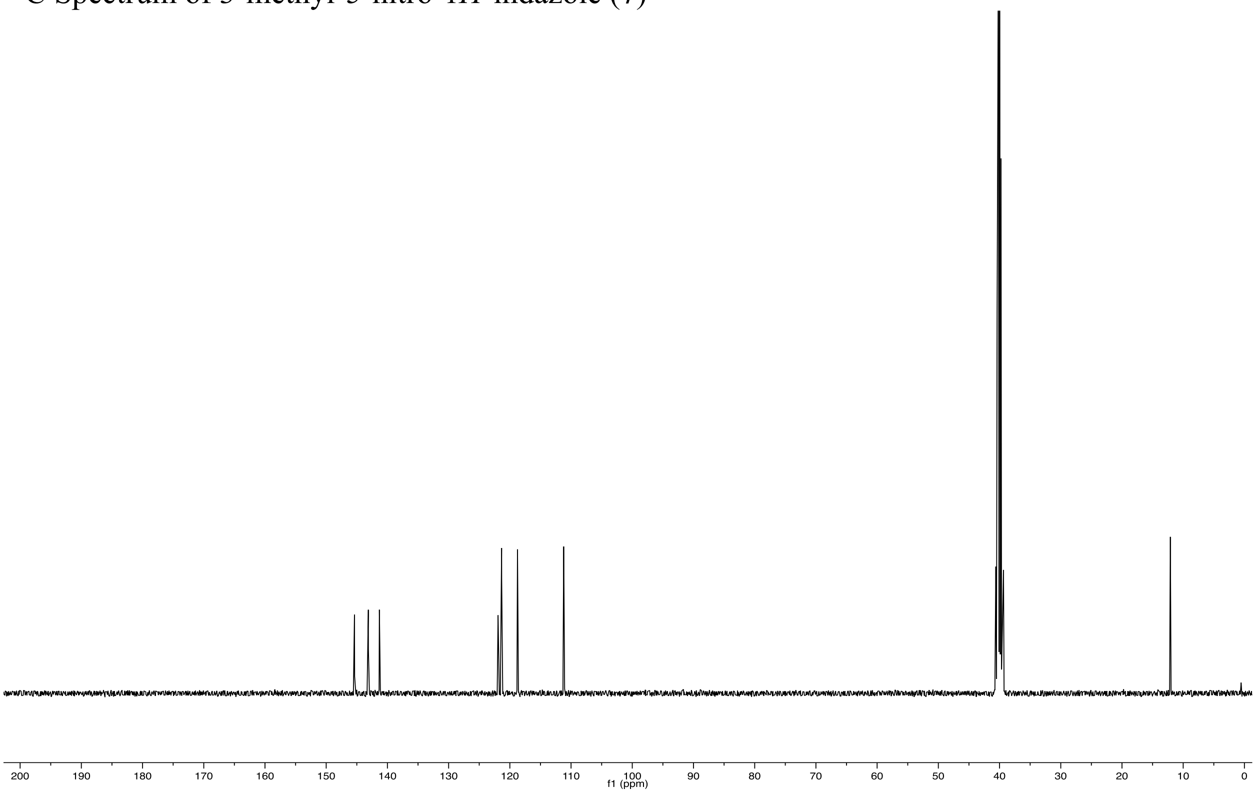

^{1}H Spectrum of 5-nitro-1*H*-indazole (**8**)

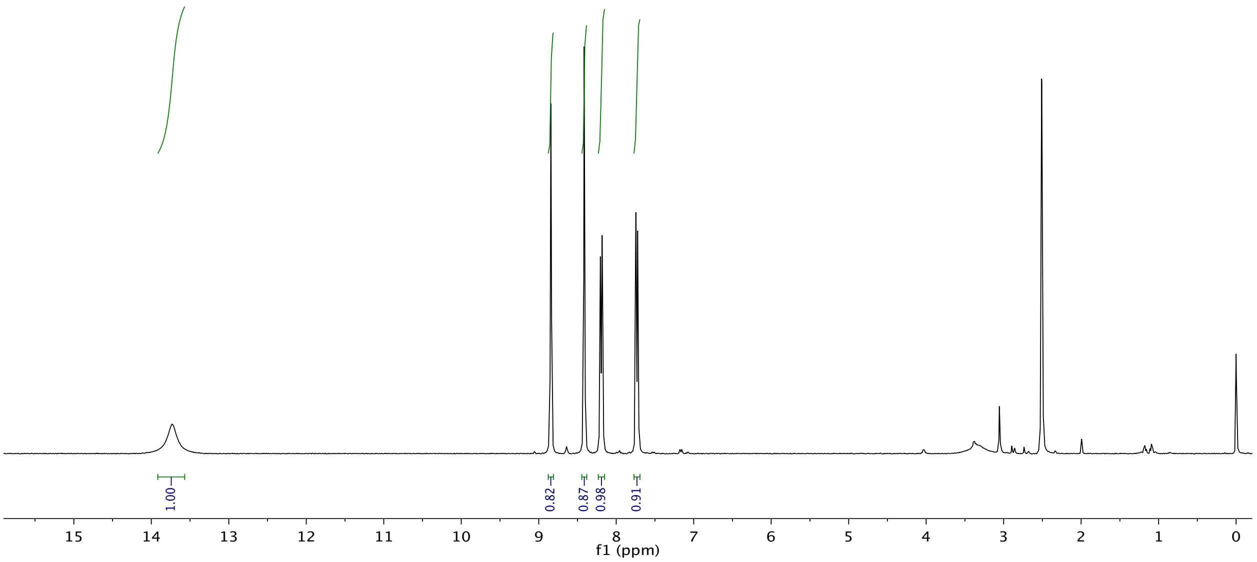

^{13}C Spectrum of 5-nitro-1*H*-indazole (**8**)

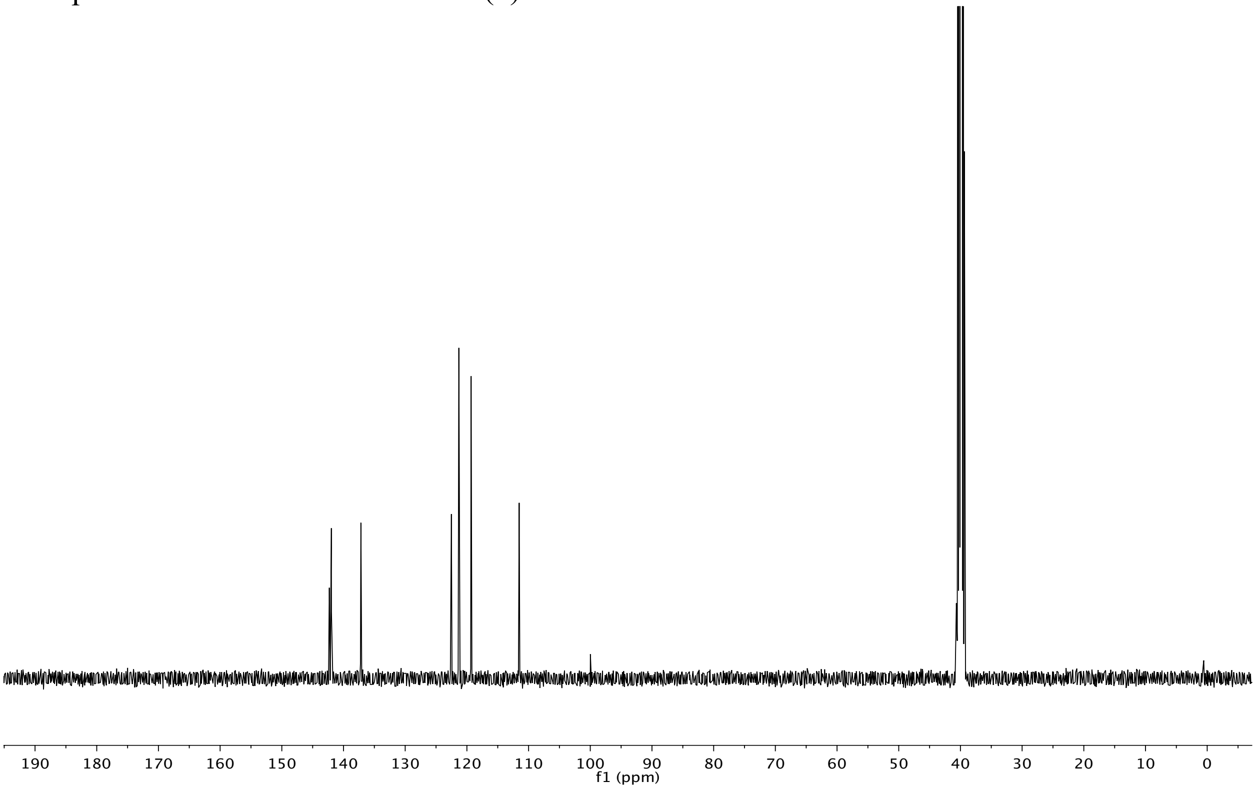

^{1}H Spectrum of 3-methyl-1-phenyl-5-nitro-1*H*-indazole (**11a**)

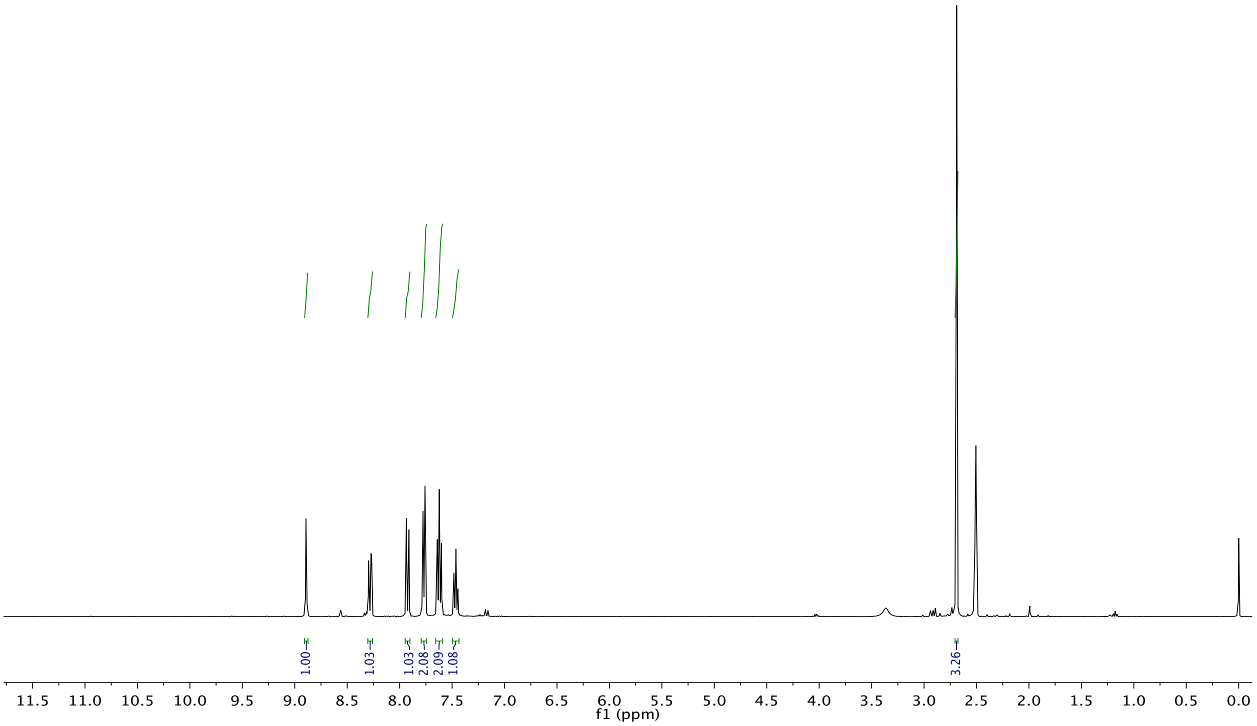

^{13}C Spectrum of 3-methyl-1-phenyl-5-nitro-1*H*-indazole (**11a**)

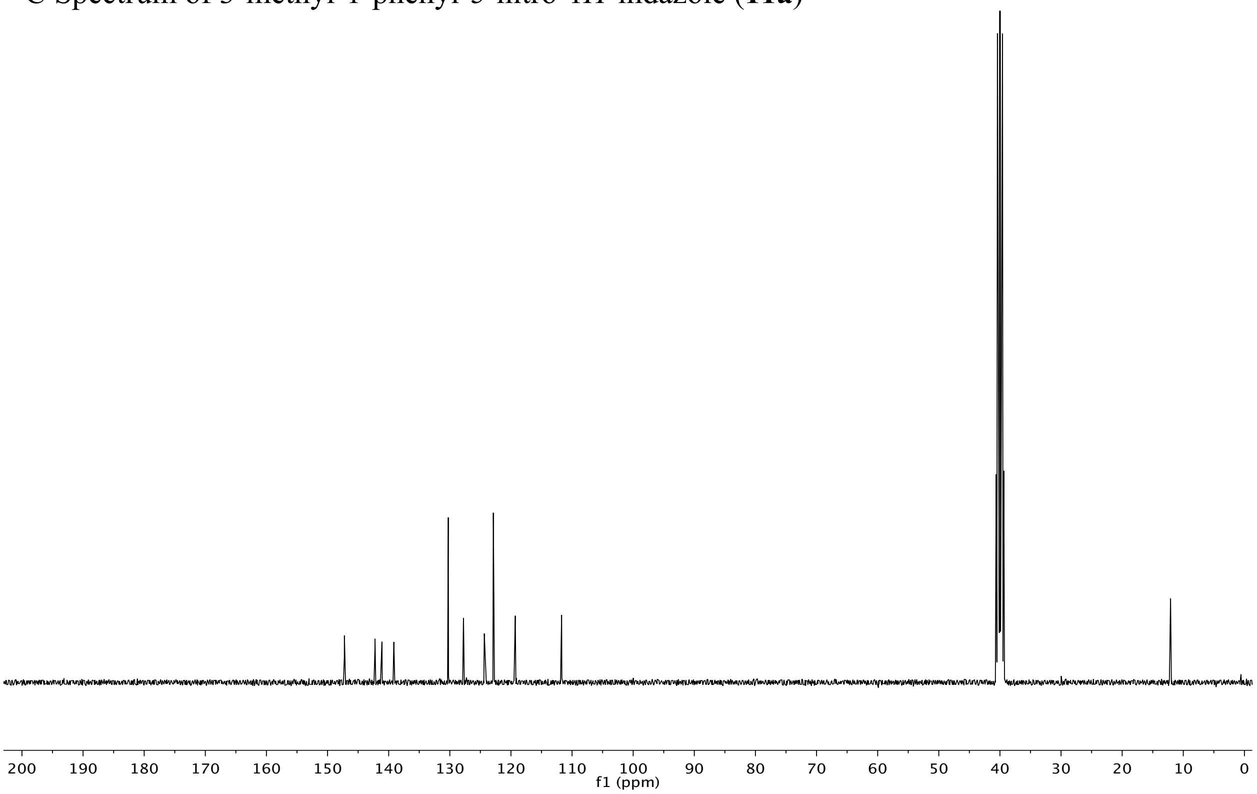

[1]H Spectrum of 1-(2-methoxyphenyl)-3-methyl-5-nitro-1*H*-indazole (**11b**)

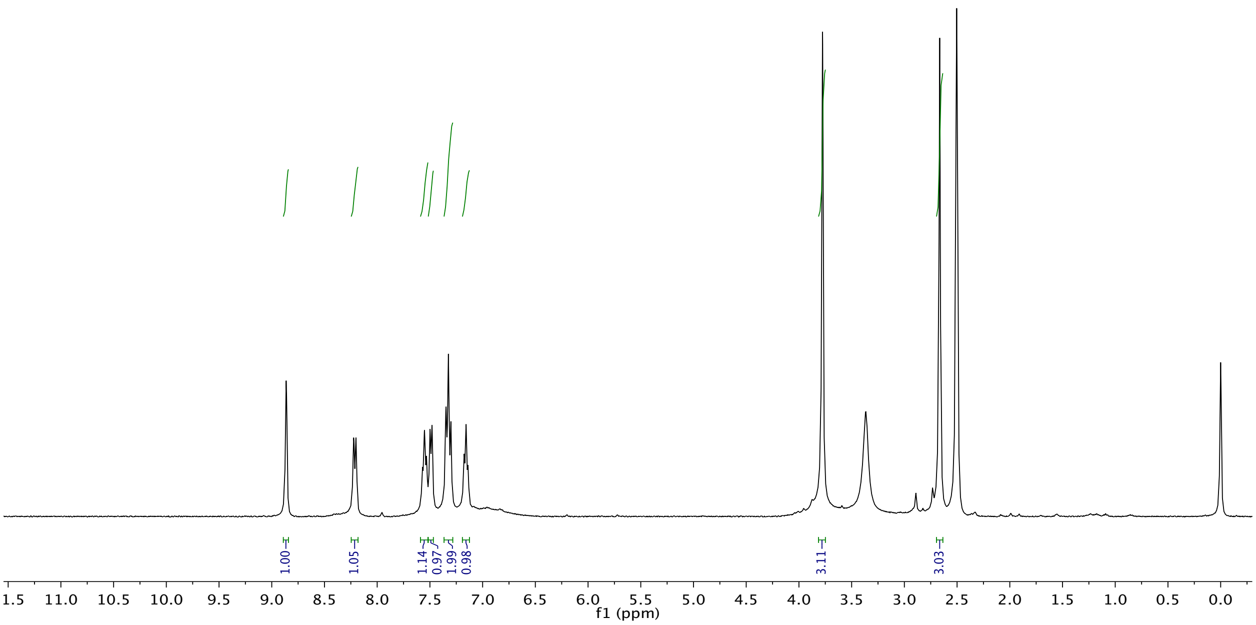

[13]C Spectrum of 1-(2-methoxyphenyl)-3-methyl-5-nitro-1*H*-indazole (**11b**)

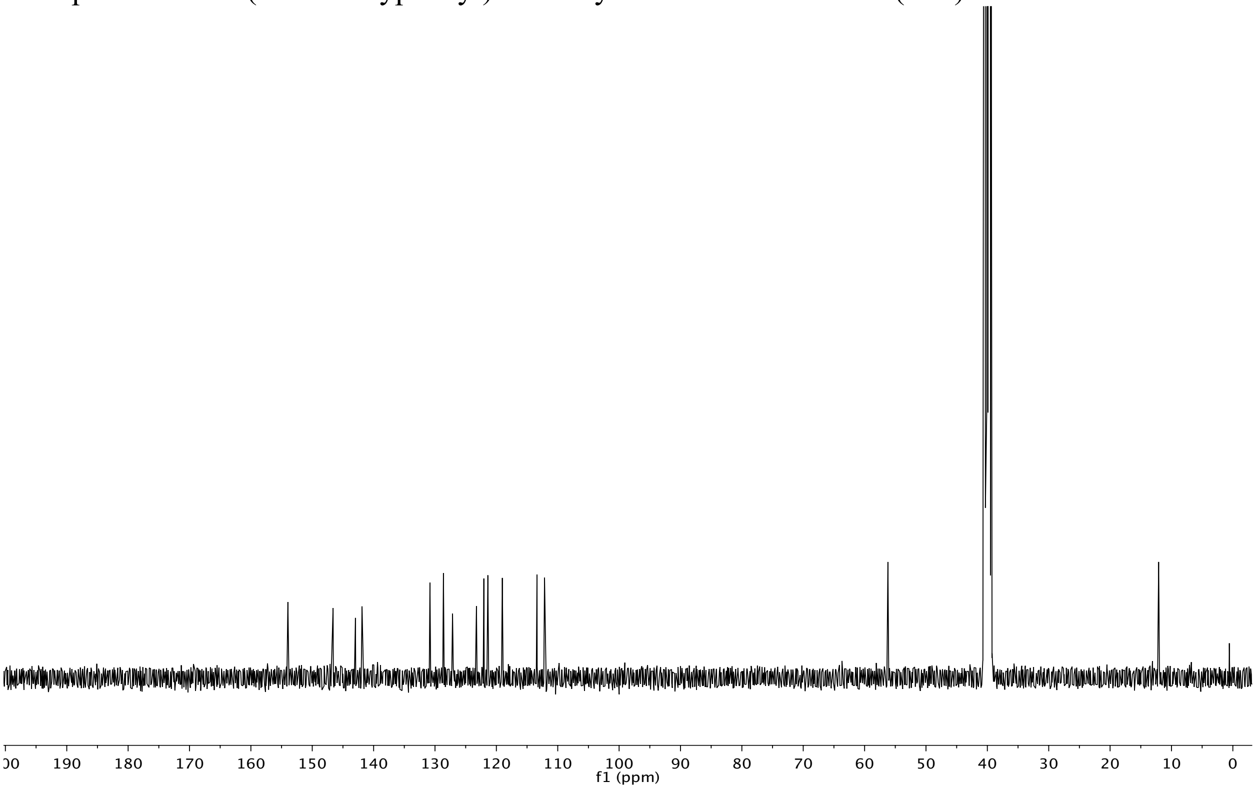

^{1}H Spectrum of 1-(3-methoxyphenyl)-3-methyl-5-nitro-1*H*-indazole (**11c**)

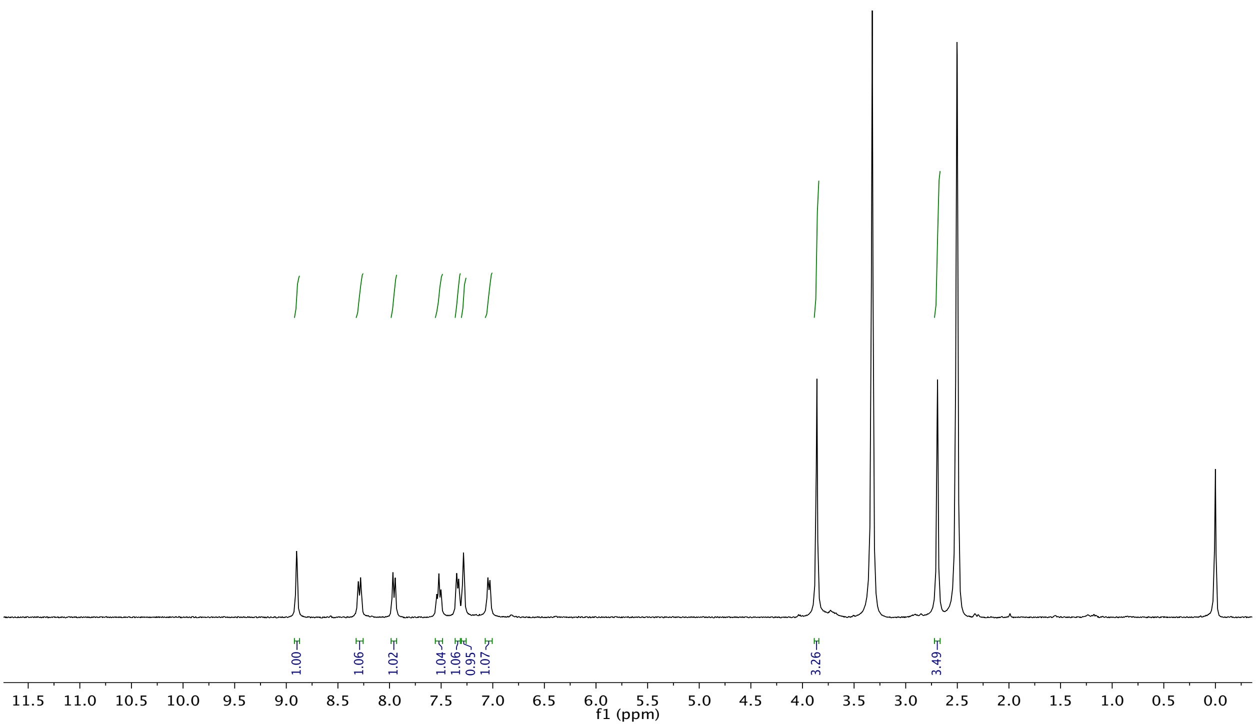

^{13}C Spectrum of 1-(3-methoxyphenyl)-3-methyl-5-nitro-1*H*-indazole (**11c**)

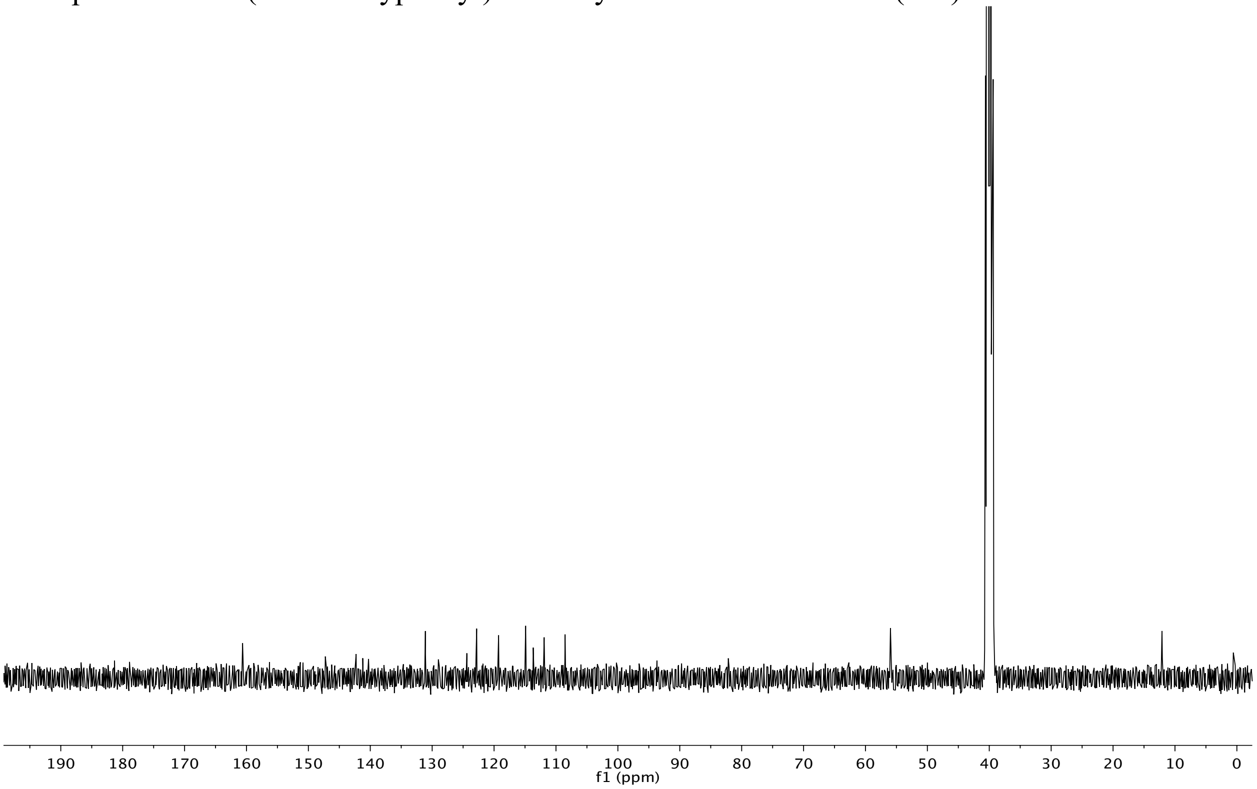

^{1}H Spectra of 1-(4-methoxyphenyl)-3-methyl-5-nitro-1*H*-indazole (**11d**)

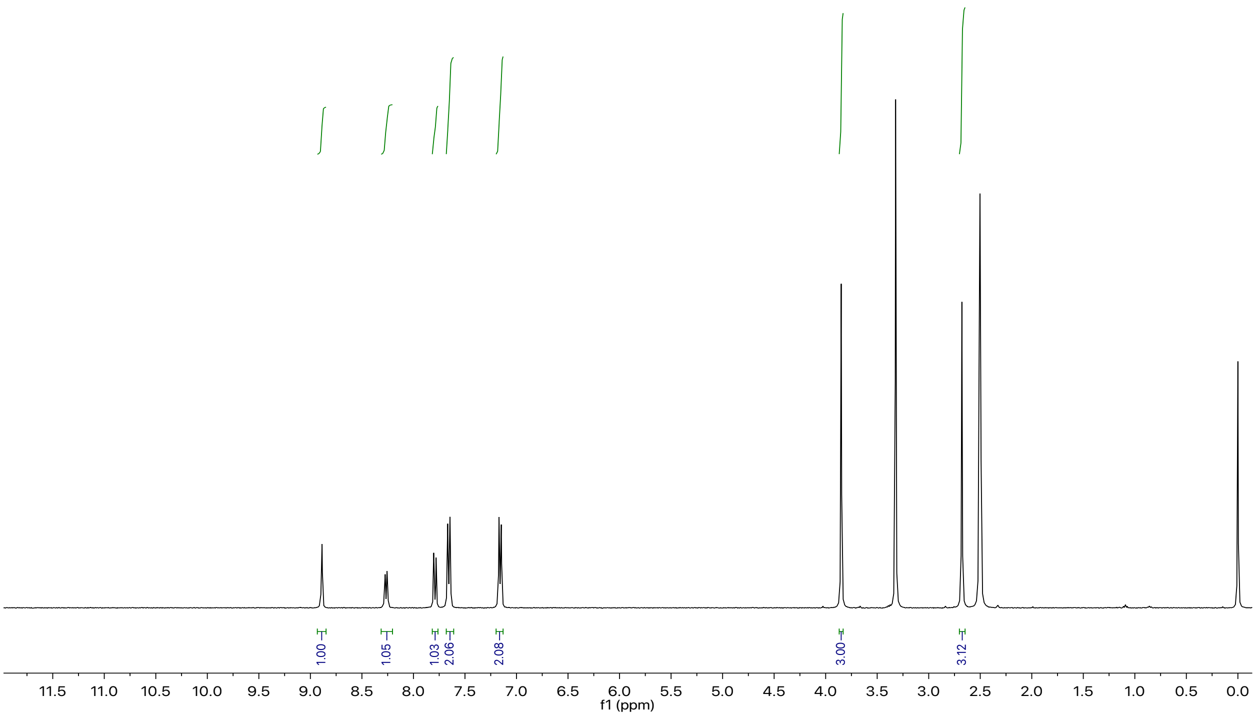

^{13}C Spectrum of 1-(4-methoxyphenyl)-3-methyl-5-nitro-1*H*-indazole (**11d**)

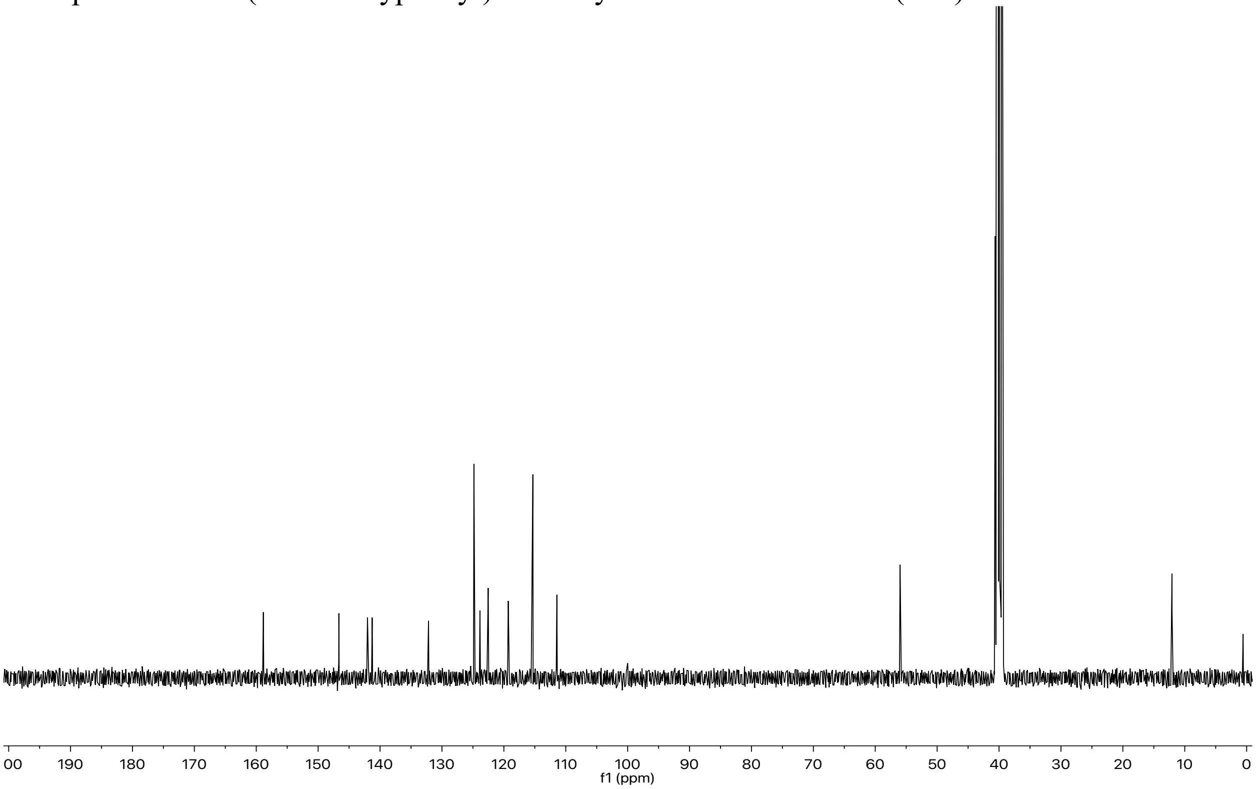

[1]H Spectrum of 1-(4-bromophenyl)-3-methyl-5-nitro-1*H*-indazole (**11e**)

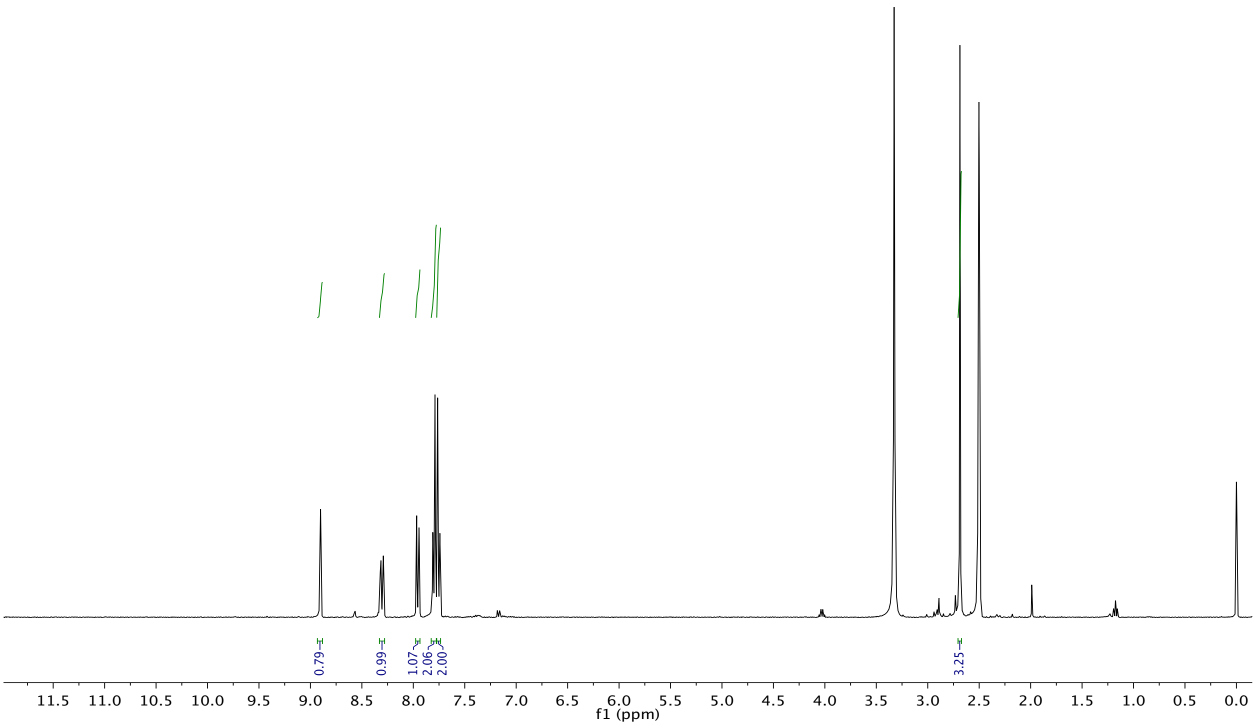

[13]C Spectrum of 1-(4-bromophenyl)-3-methyl-5-nitro-1*H*-indazole (**11e**)

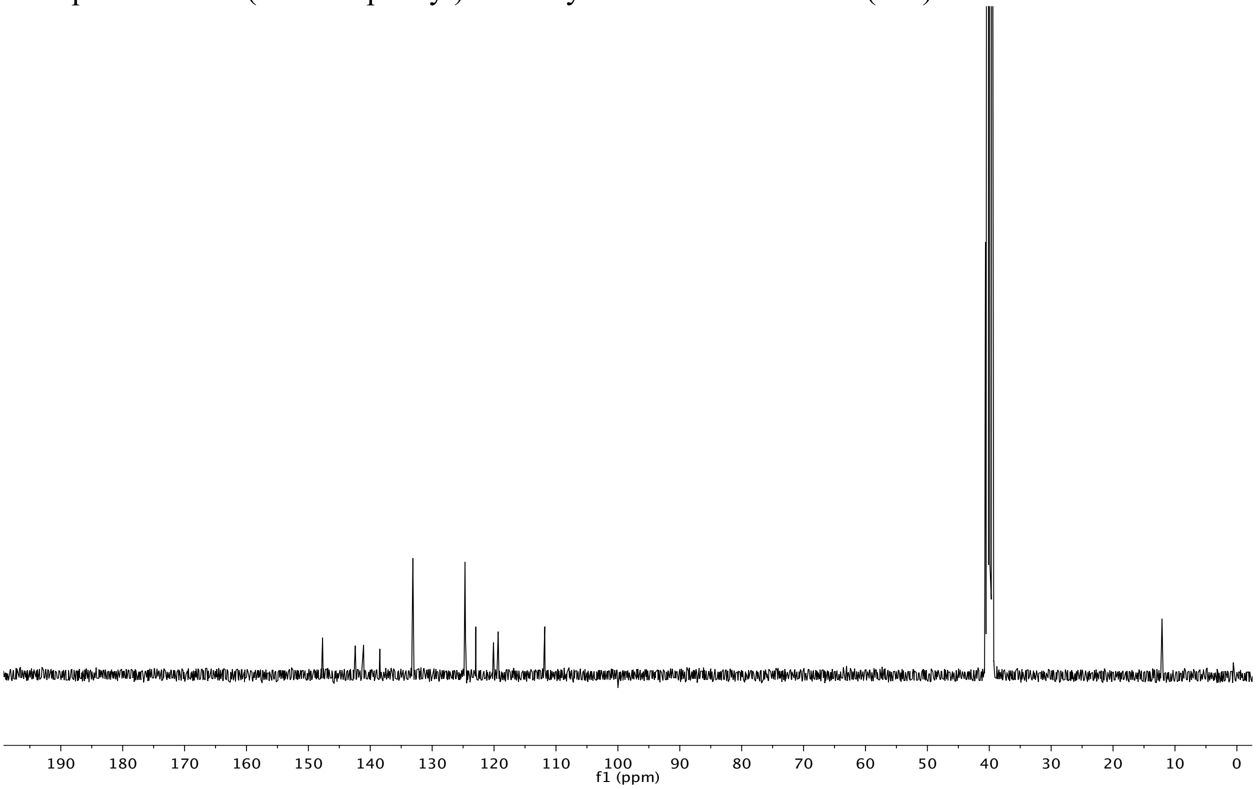

^{1}H Spectrum of 1-(3-chlorophenyl)-3-methyl-5-nitro-1*H*-indazole (**11f**)

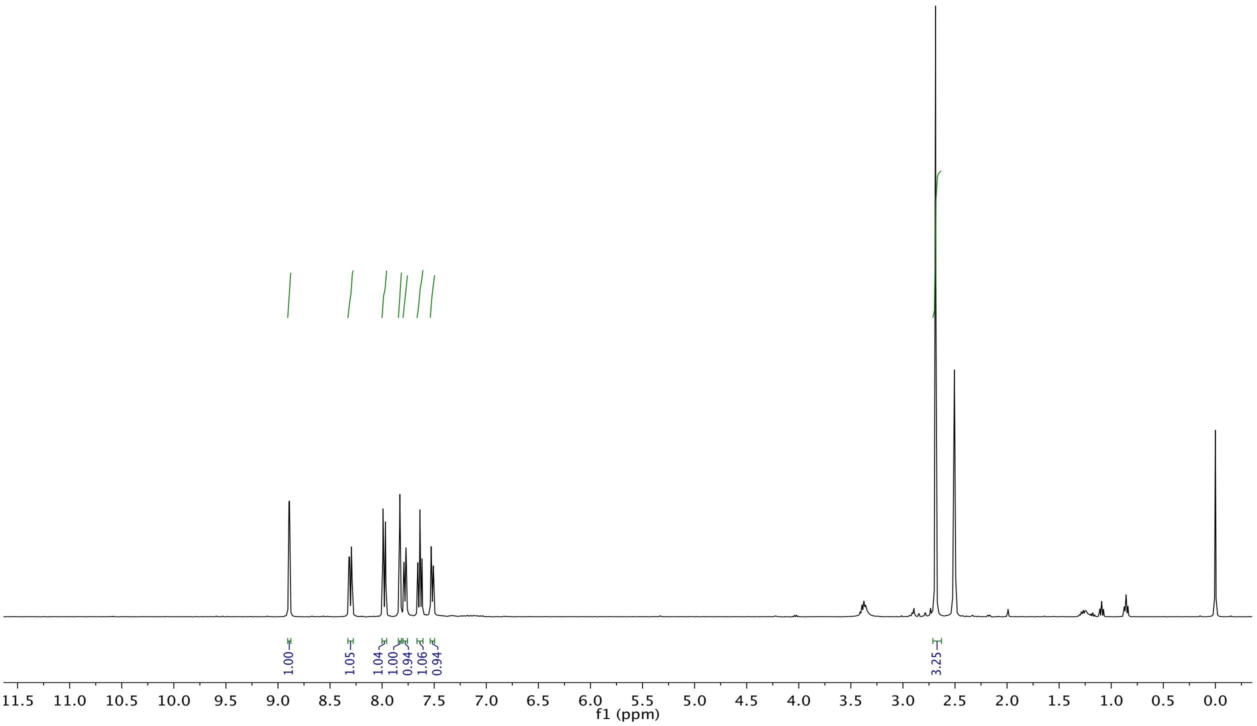

^{13}C Spectrum of 1-(3-chlorophenyl)-3-methyl-5-nitro-1*H*-indazole (**11f**)

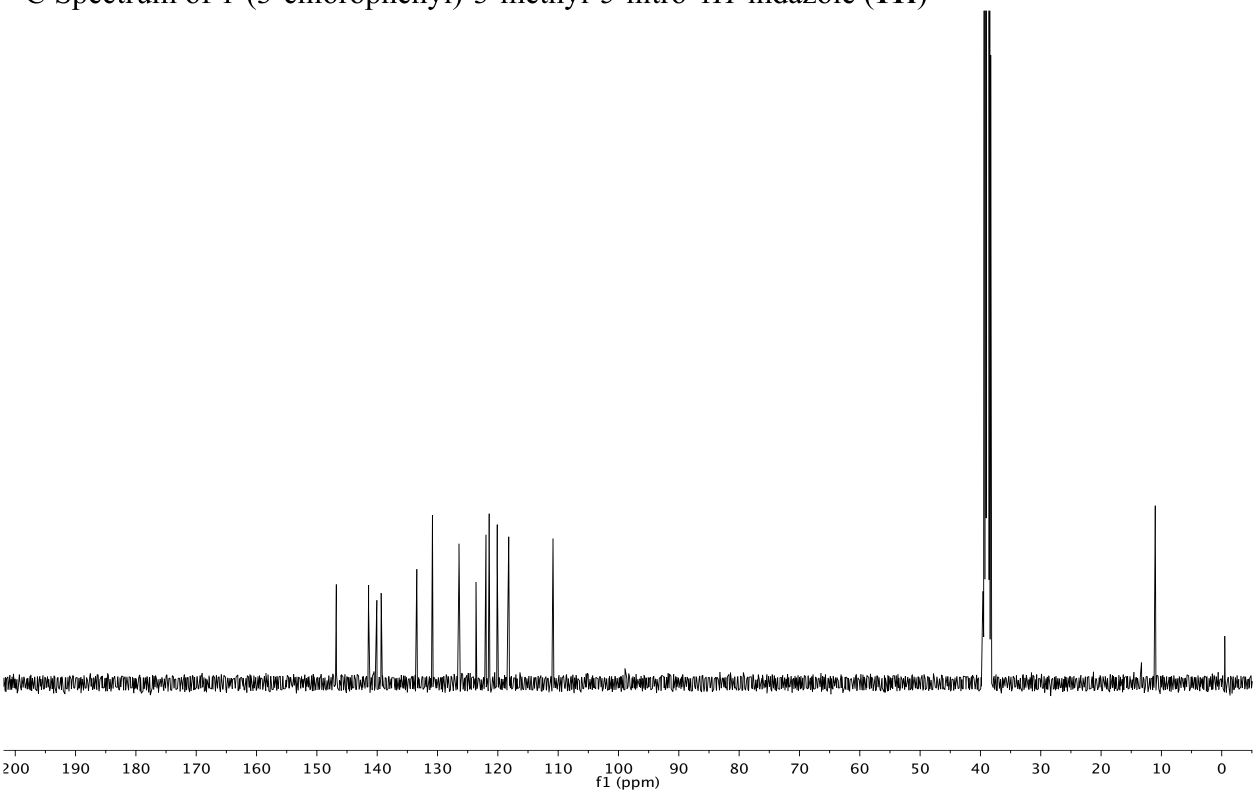

[1]H Spectrum of 1-(4-chlorophenyl)-3-methyl-5-nitro-1*H*-indazole (**11g**)

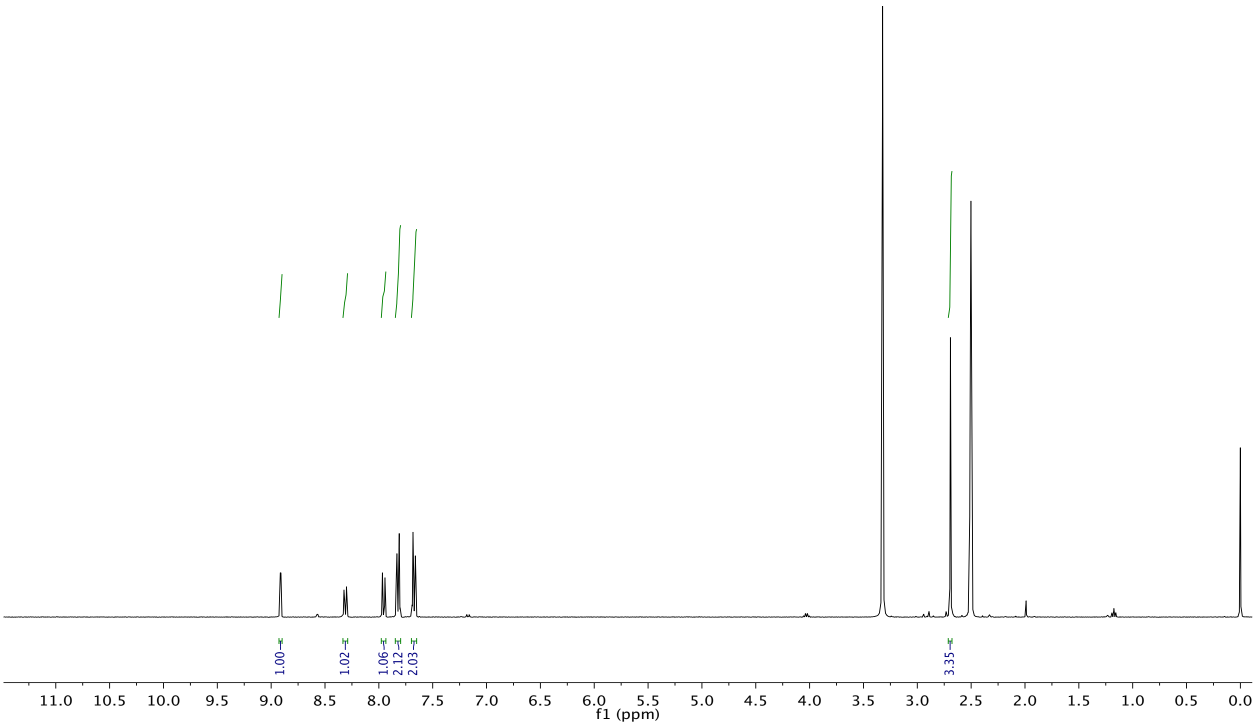

[13]C Spectrum of 1-(4-chlorophenyl)-3-methyl-5-nitro-1*H*-indazole (**11g**)

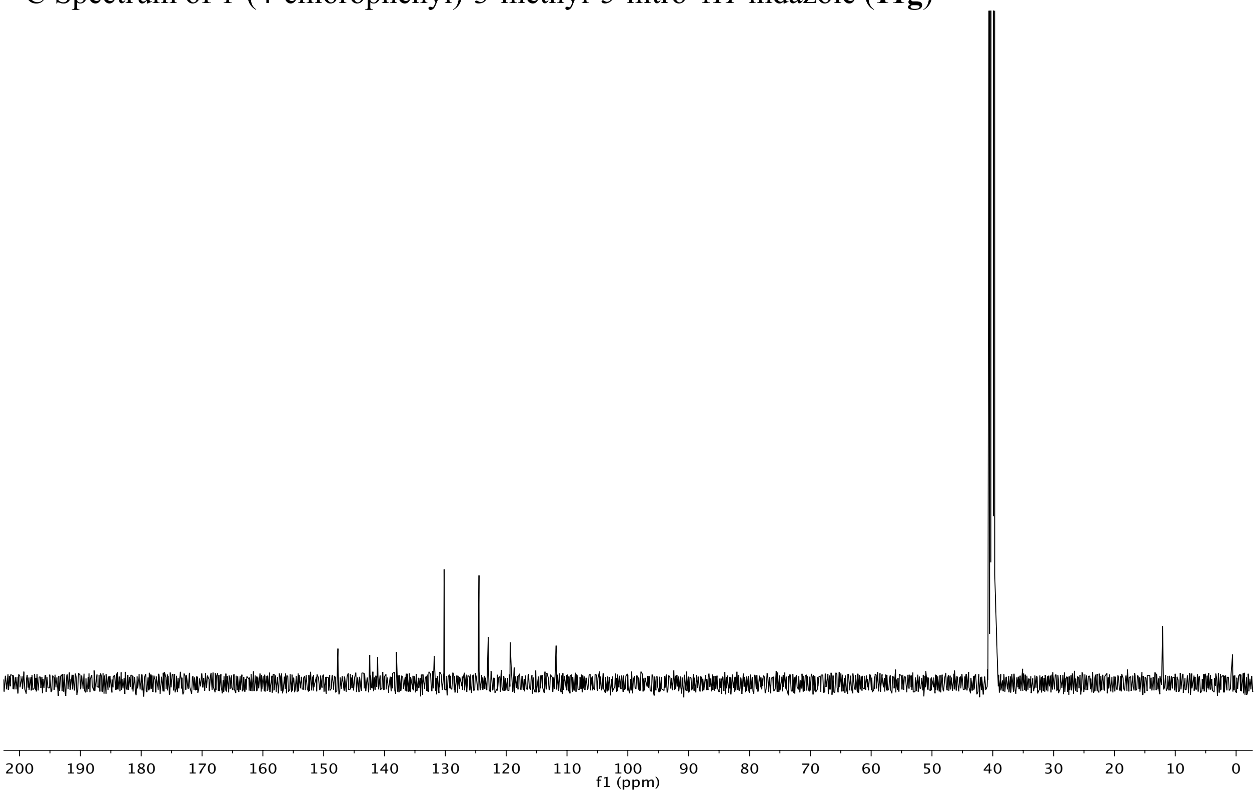

[1]H Spectrum of 1-(2,4-dichlorophenyl)-3-methyl-5-nitro-1*H*-indazole (**11h**)

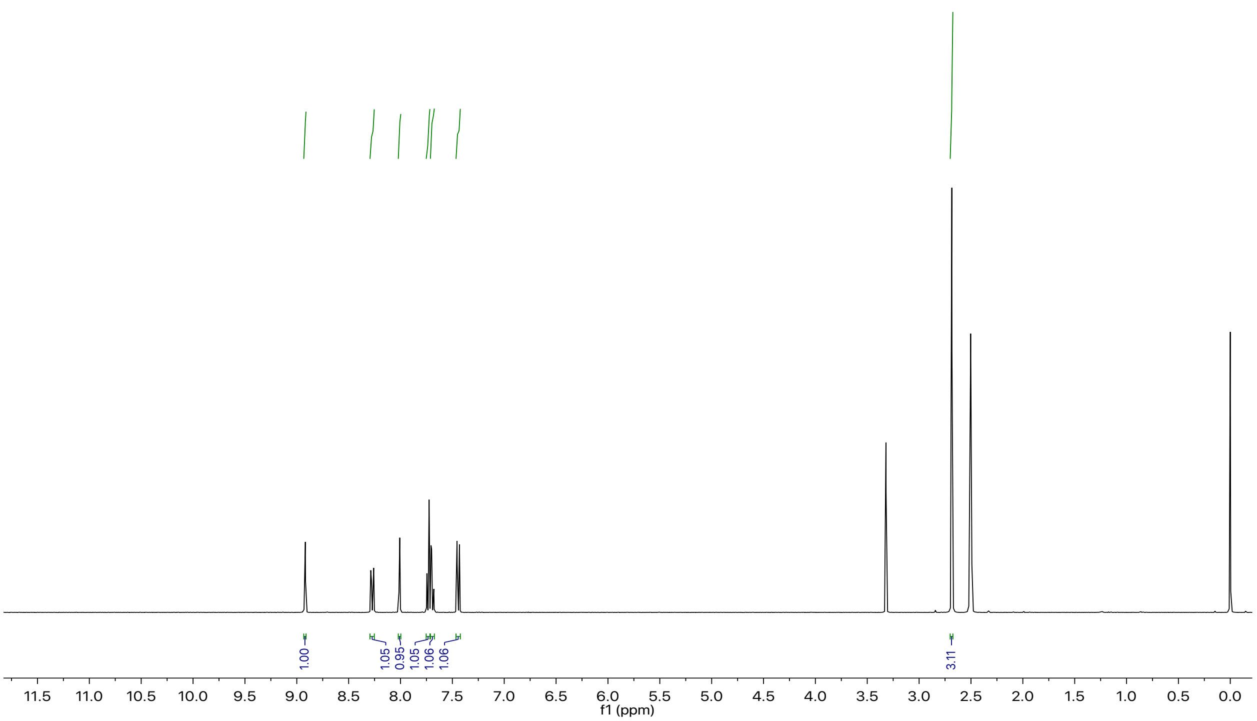

[13]C Spectrum of 1-(2,4-dichlorophenyl)-3-methyl-5-nitro-1*H*-indazole (**11h**)

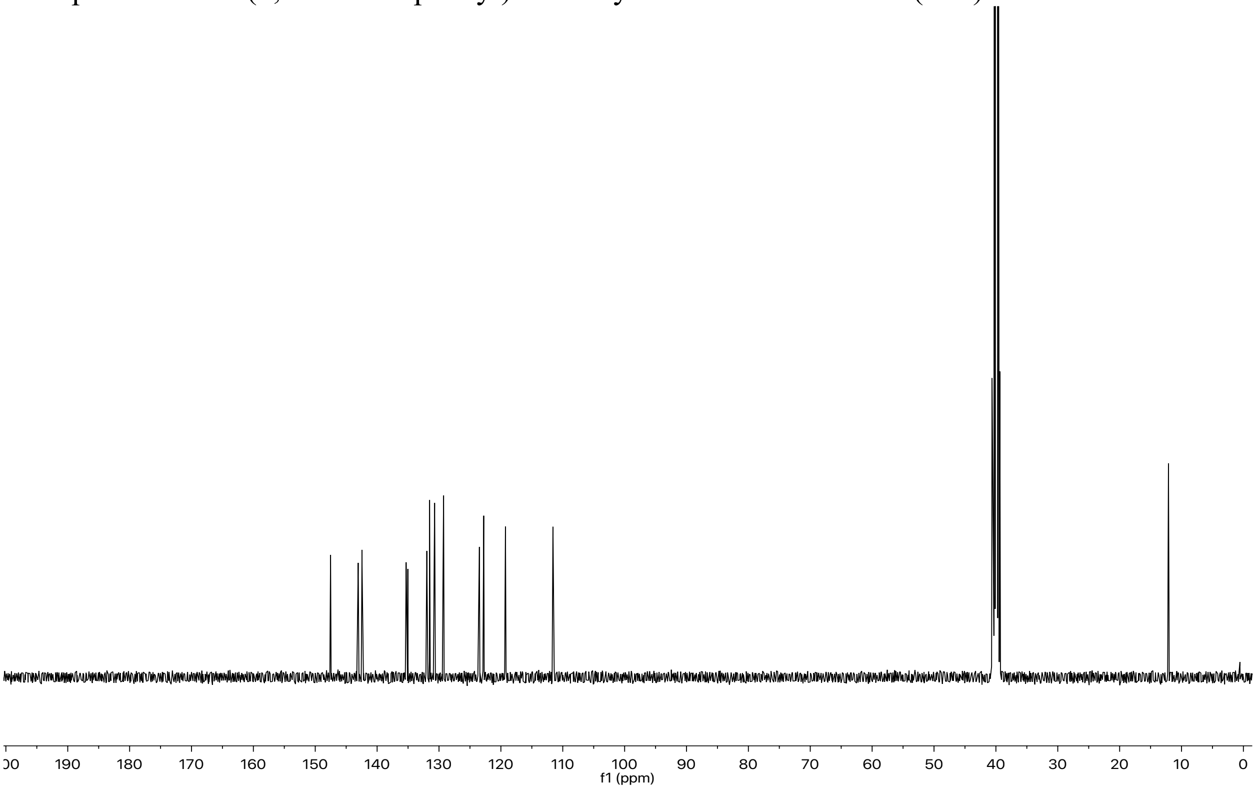

[1]H Spectrum of 3-methyl-5-nitro-1-(3-(trifluoromethyl)phenyl)-1*H*-indazole (**11i**)

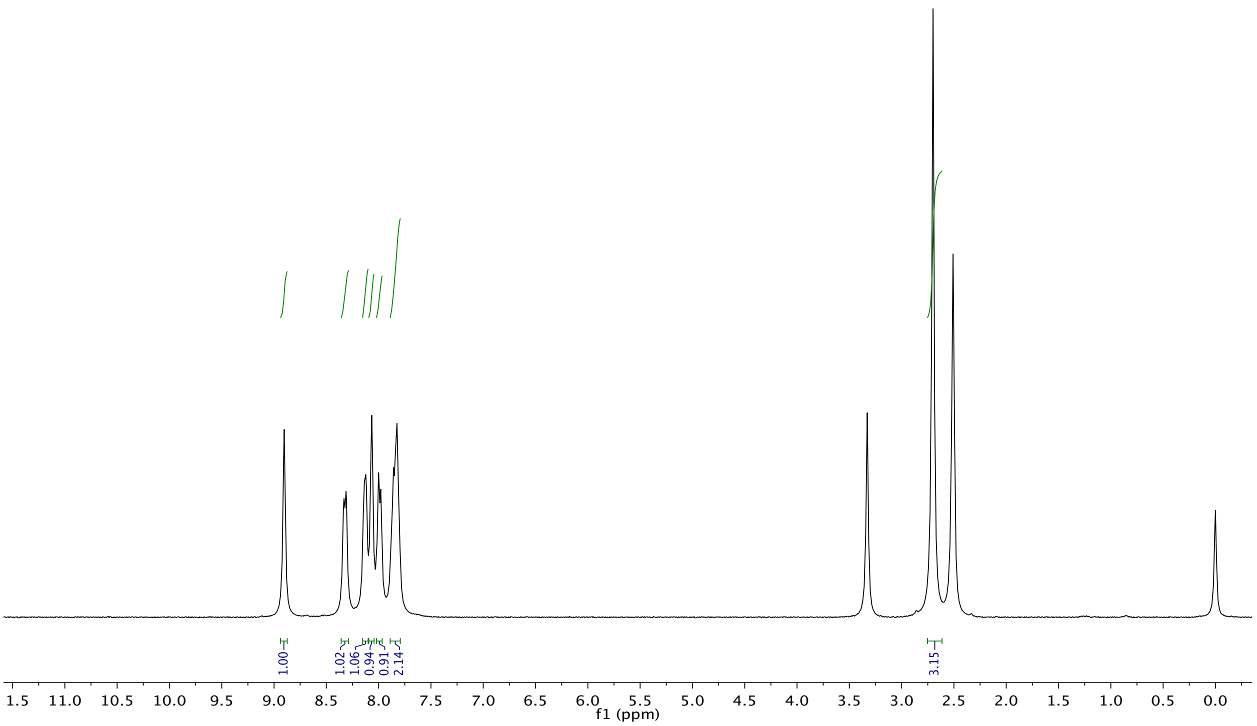

[13]C Spectrum of 3-methyl-5-nitro-1-(3-(trifluoromethyl)phenyl)-1*H*-indazole (**11i**)

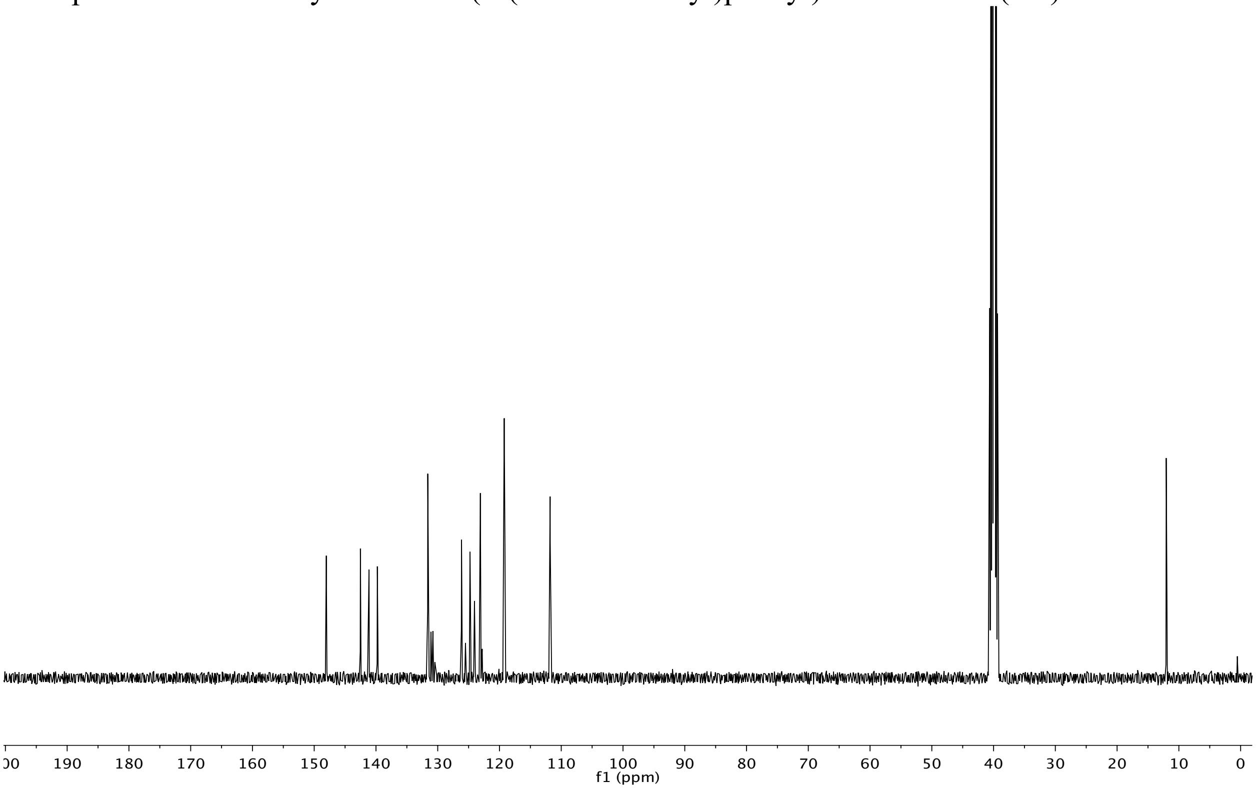

^{1}H Spectra of 3-methyl-5-nitro-1-(4-(trifluoromethyl)phenyl)-1*H*-indazole (**11j**)

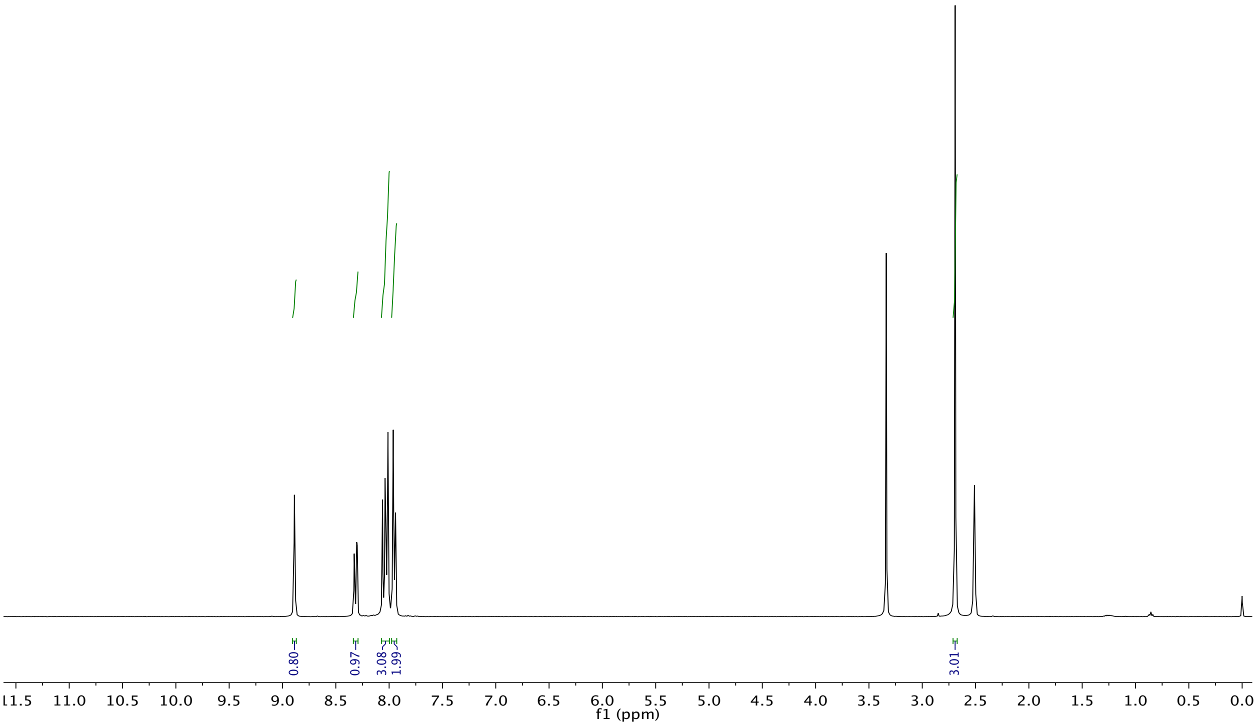

^{13}C Spectra of 3-methyl-5-nitro-1-(4-(trifluoromethyl)phenyl)-1*H*-indazole (**11j**)

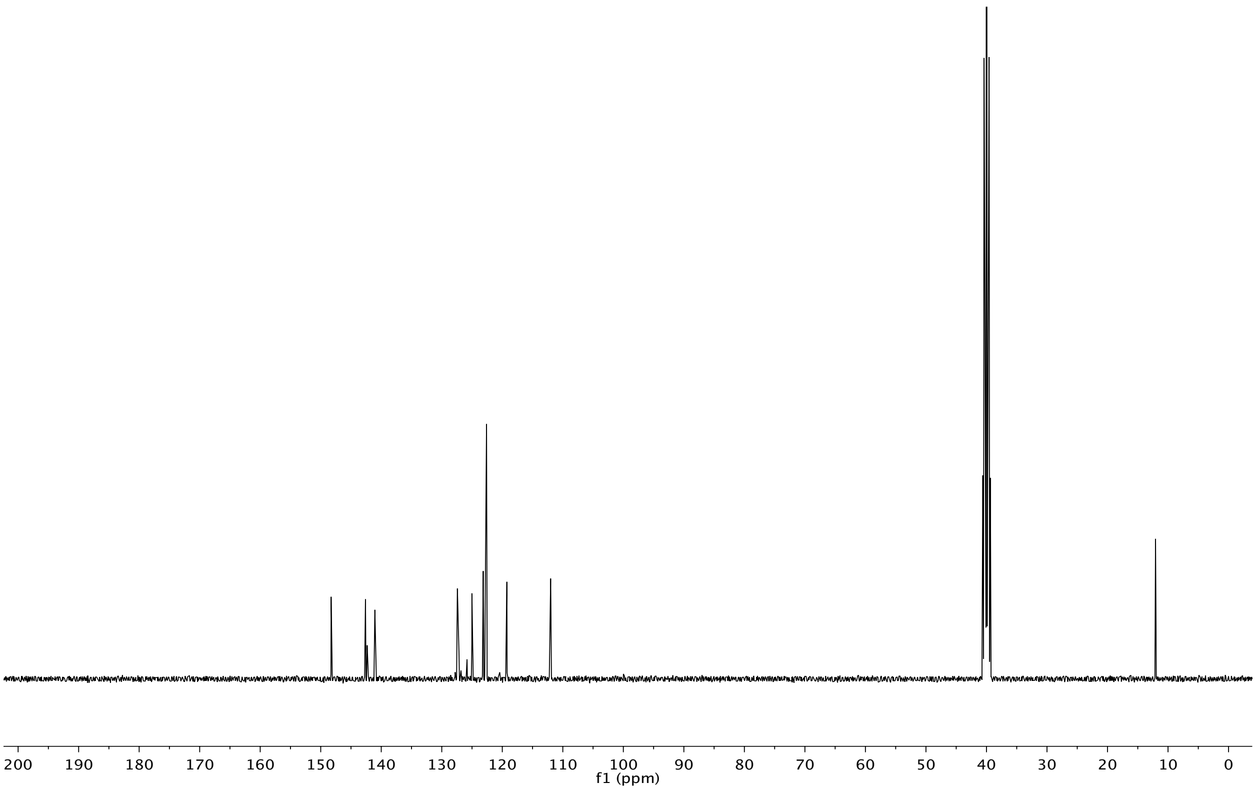

^{1}H Spectrum of 1-(4-cyanophenyl)-3-methyl-5-nitro-1*H*-indazole (**11k**)

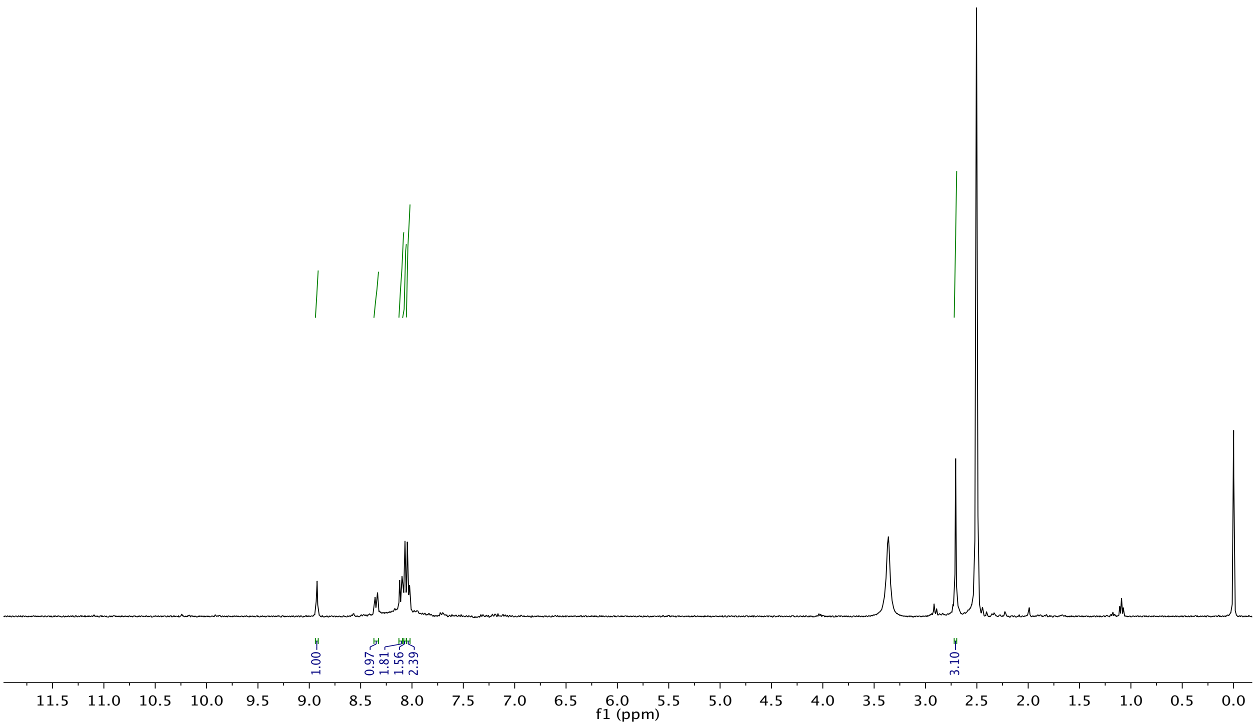

^{13}C Spectrum of 1-(4-cyanophenyl)-3-methyl-5-nitro-1*H*-indazole (**11k**)

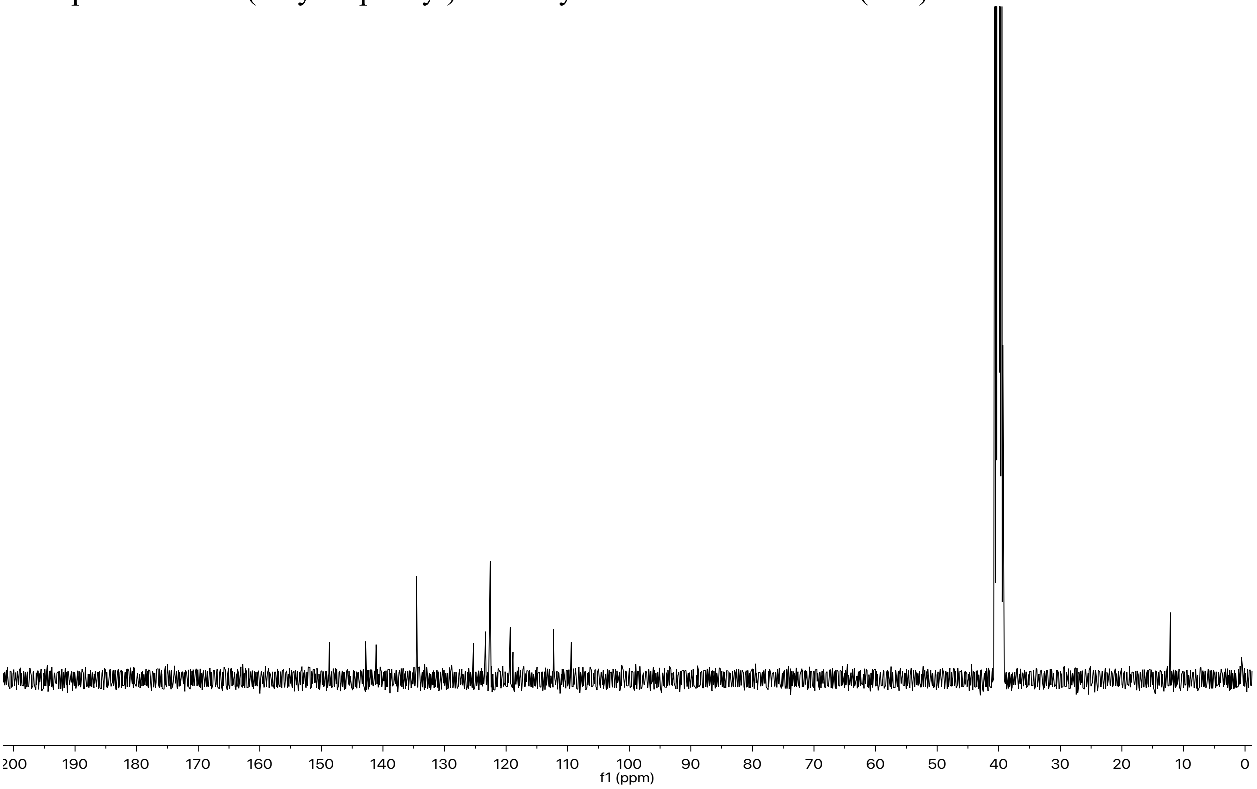

[1]H Spectrum of 4-(3-methyl-5-nitro-1*H*-indazol-1-yl)benzenesulfonamide (**11l**)

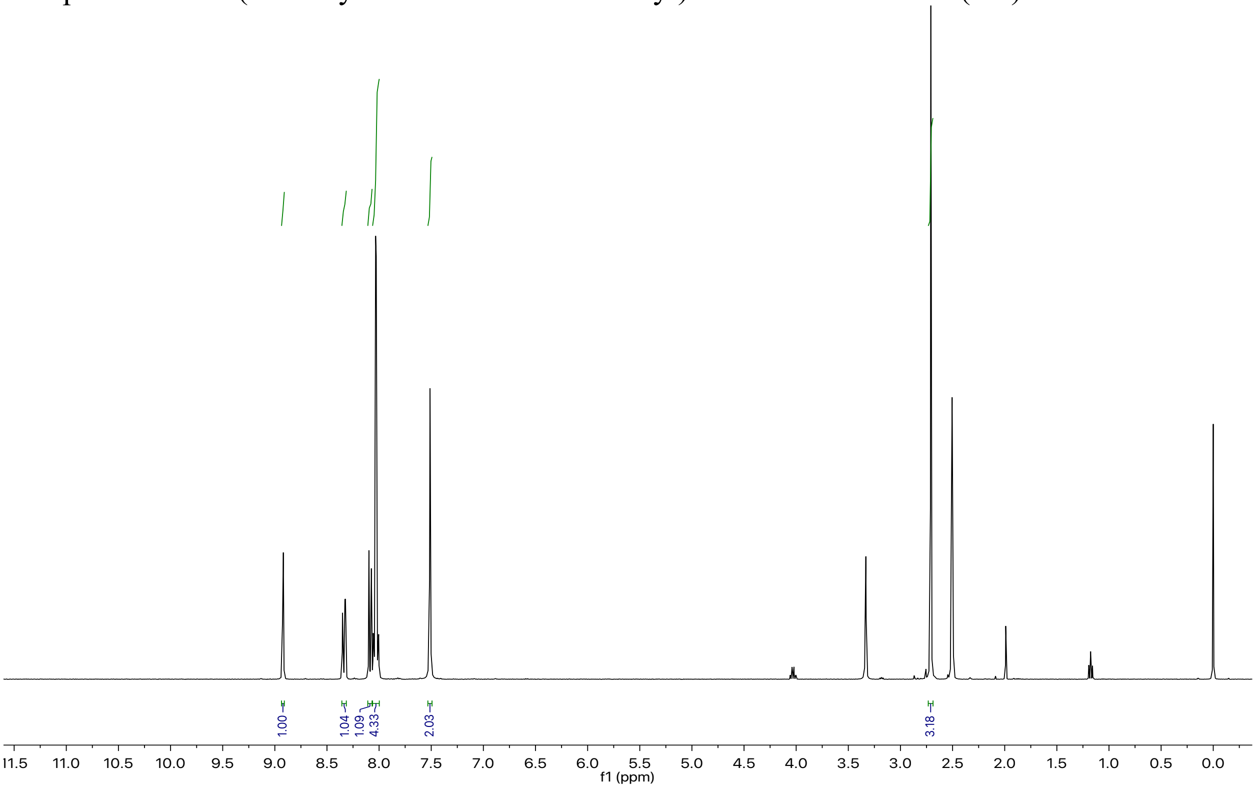

[13]C Spectrum of 4-(3-methyl-5-nitro-1*H*-indazol-1-yl)benzenesulfonamide (**11l**)

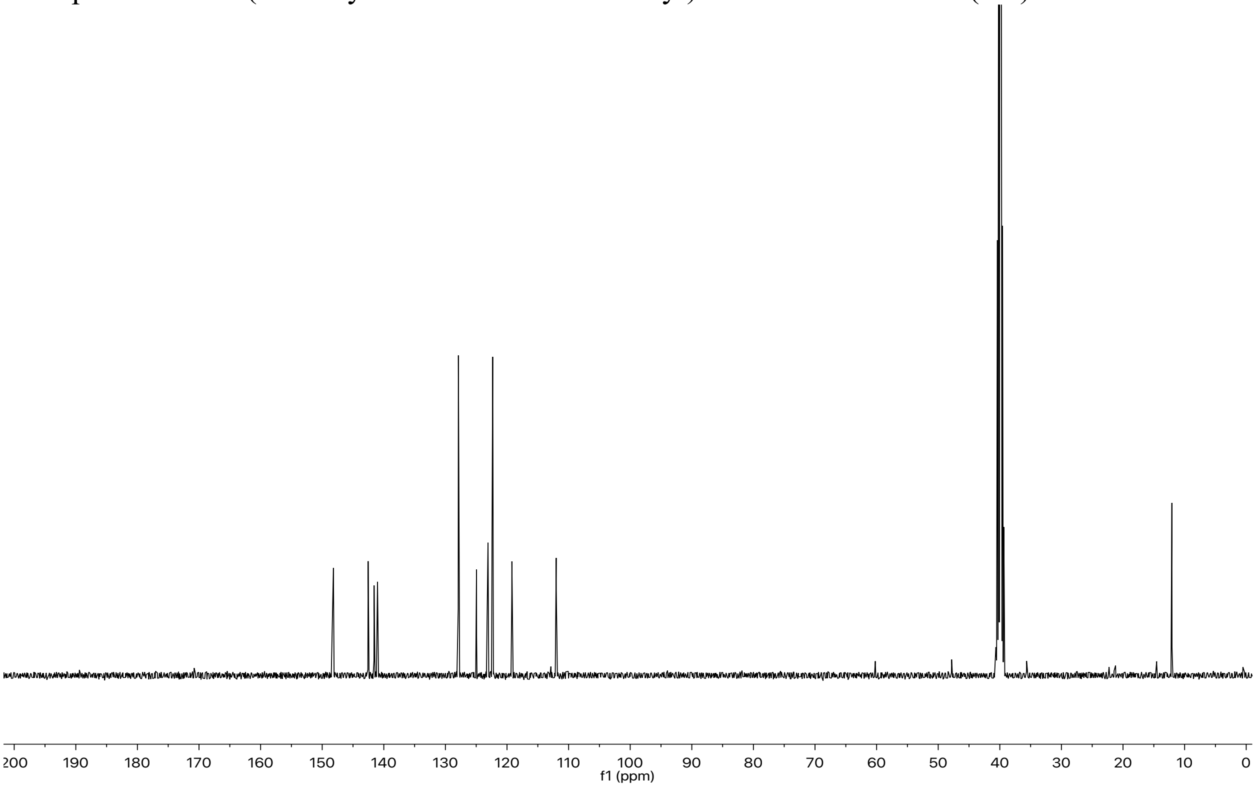

^{1}H Spectrum of 4-(3-methyl-5-nitro-1*H*-indazol-1-yl)benzoic acid (**11m**)

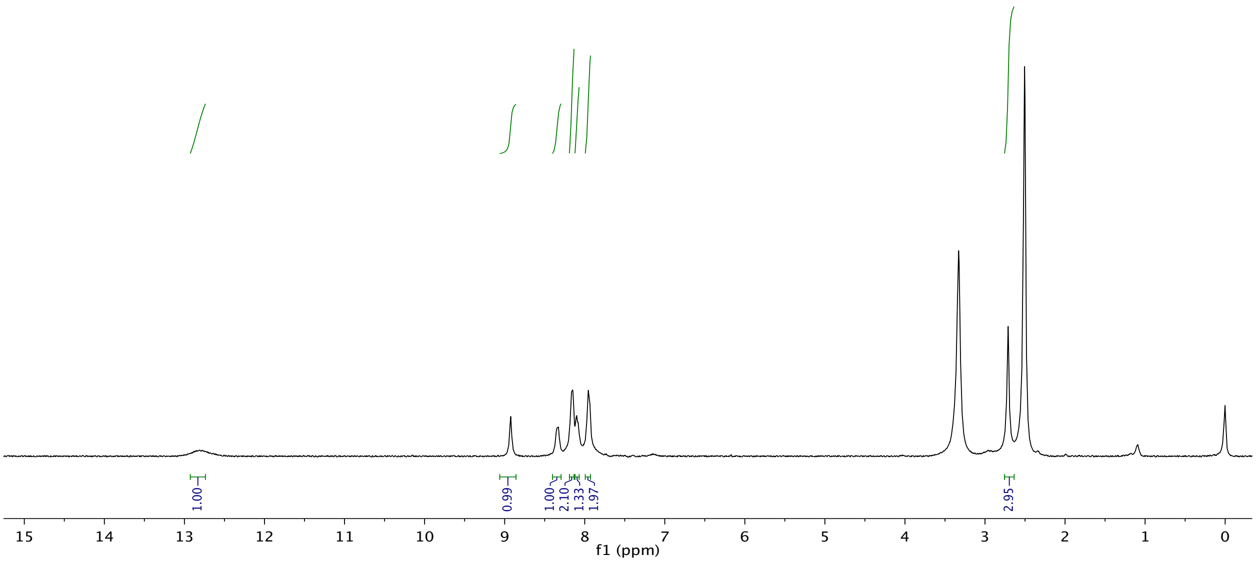

^{13}C Spectrum of 4-(3-methyl-5-nitro-1*H*-indazol-1-yl)benzoic acid (**11m**)

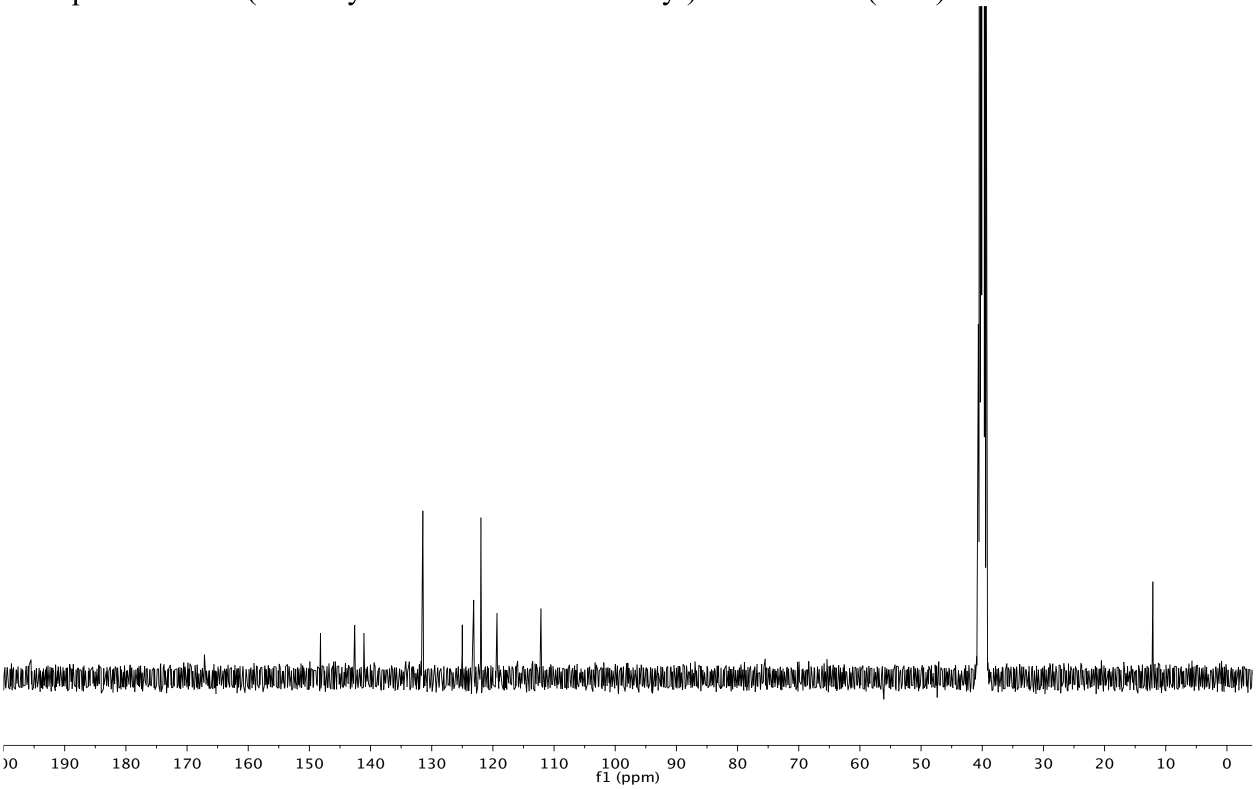

^{1}H Spectrum of 1-phenyl-5-nitro-1*H*-indazole (**12a**)

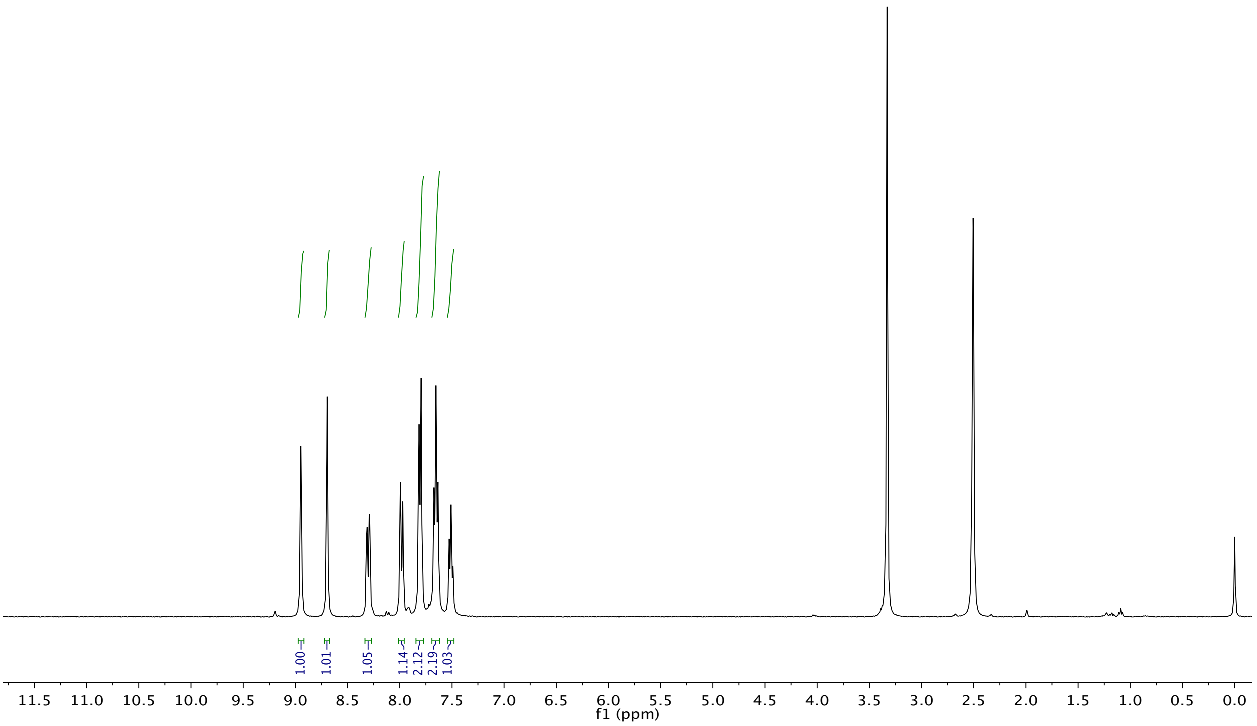

^{13}C Spectrum of 1-phenyl-5-nitro-1*H*-indazole (**12a**)

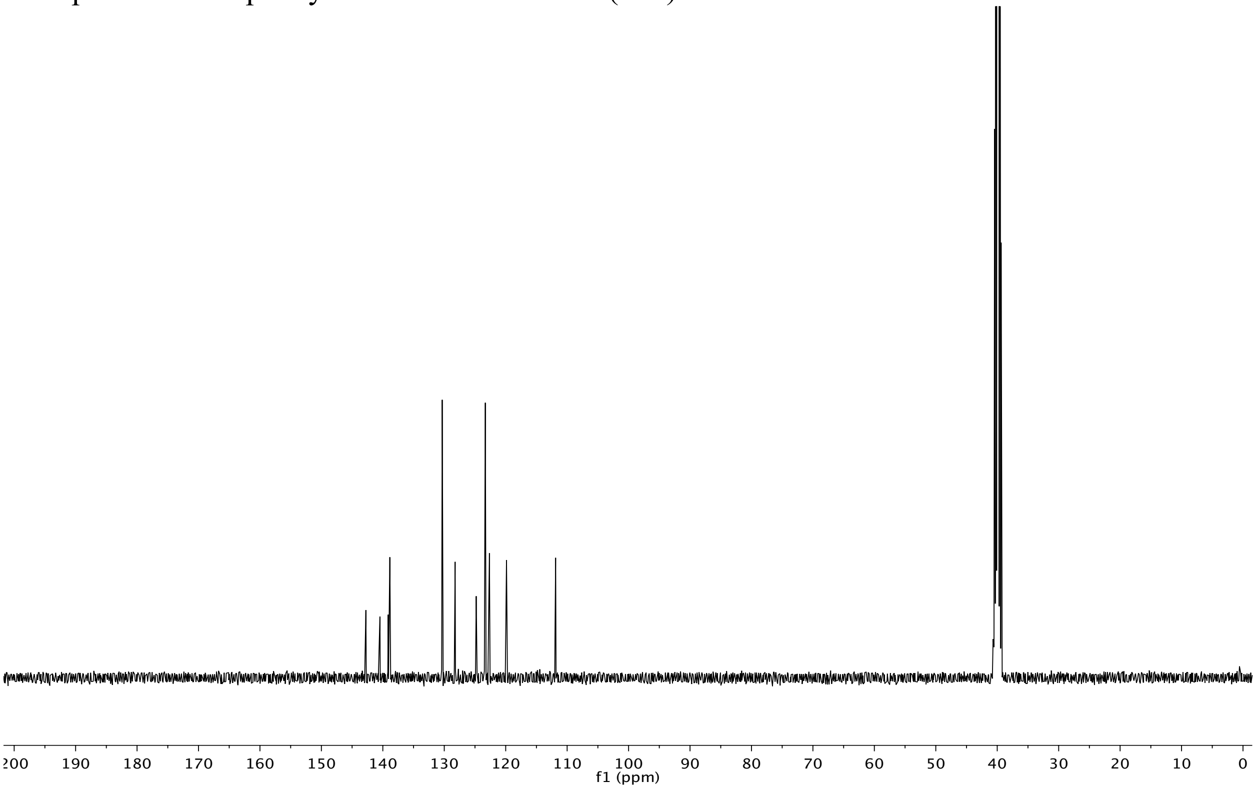

^{1}H Spectra of 1-(3-methoxyphenyl)-5-nitro-1*H*-indazole (**12c**)

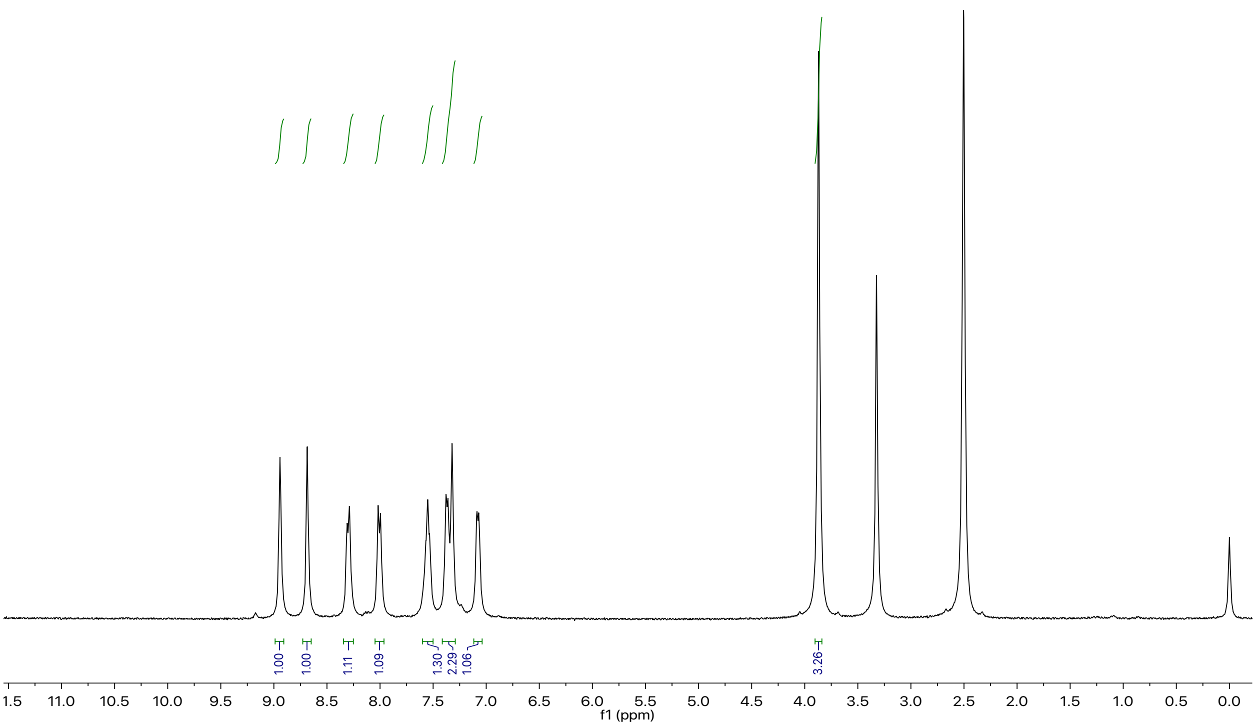

^{13}C Spectra of 1-(3-methoxyphenyl)-5-nitro-1*H*-indazole (**12c**)

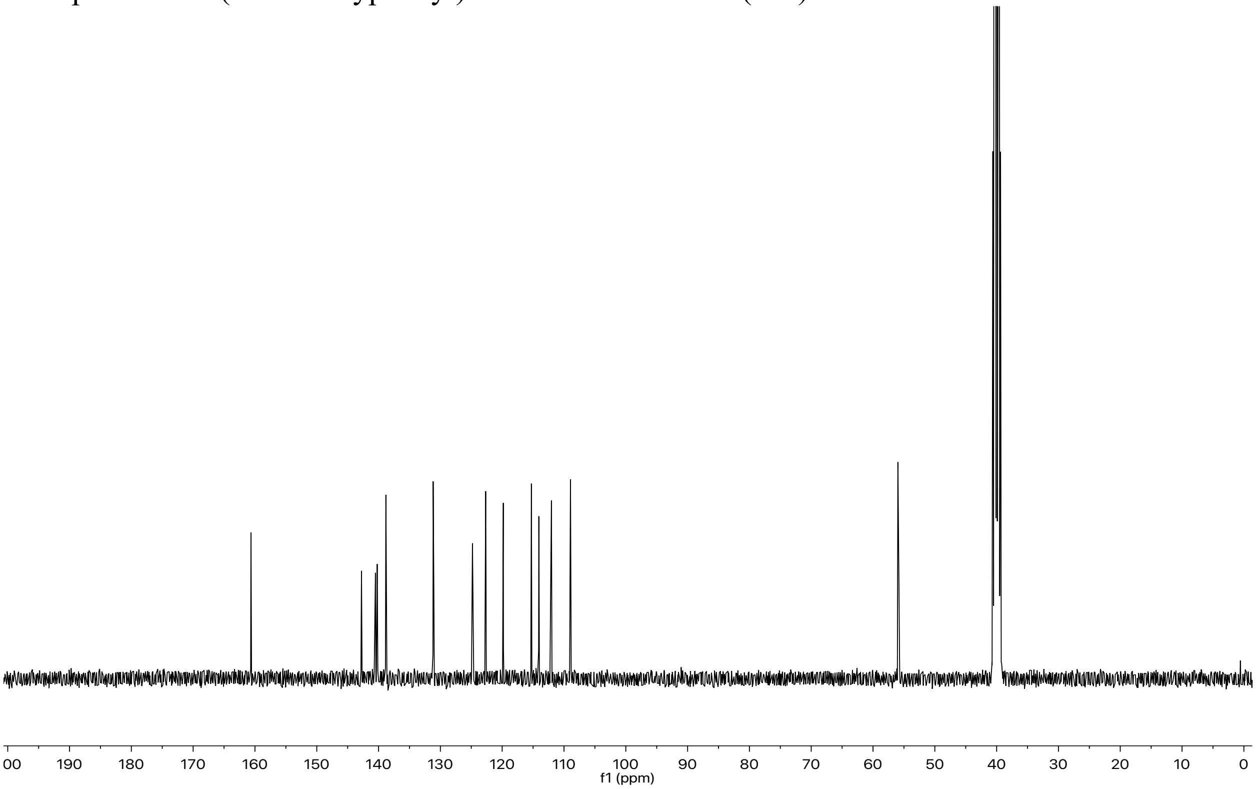

^1H Spectrum of 1-(4-methoxyphenyl)-5-nitro-1*H*-indazole (**12d**)

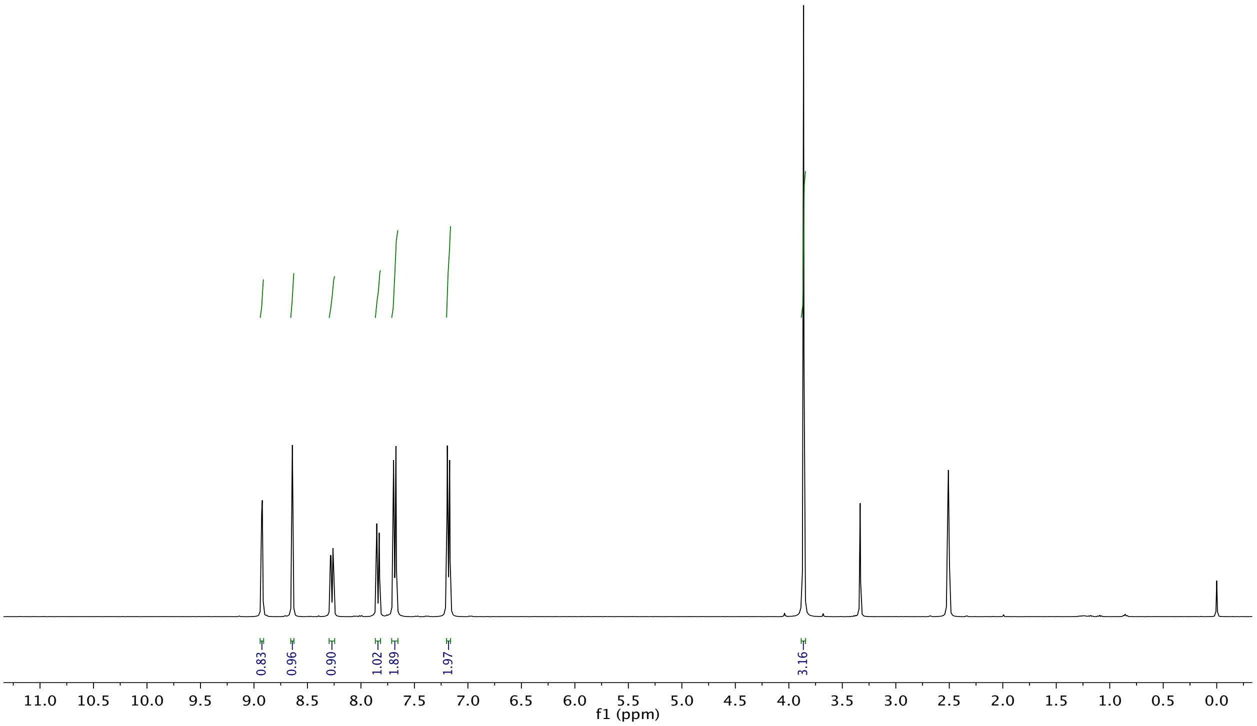

^{13}C Spectrum of 1-(4-methoxyphenyl)-5-nitro-1*H*-indazole (**12d**)

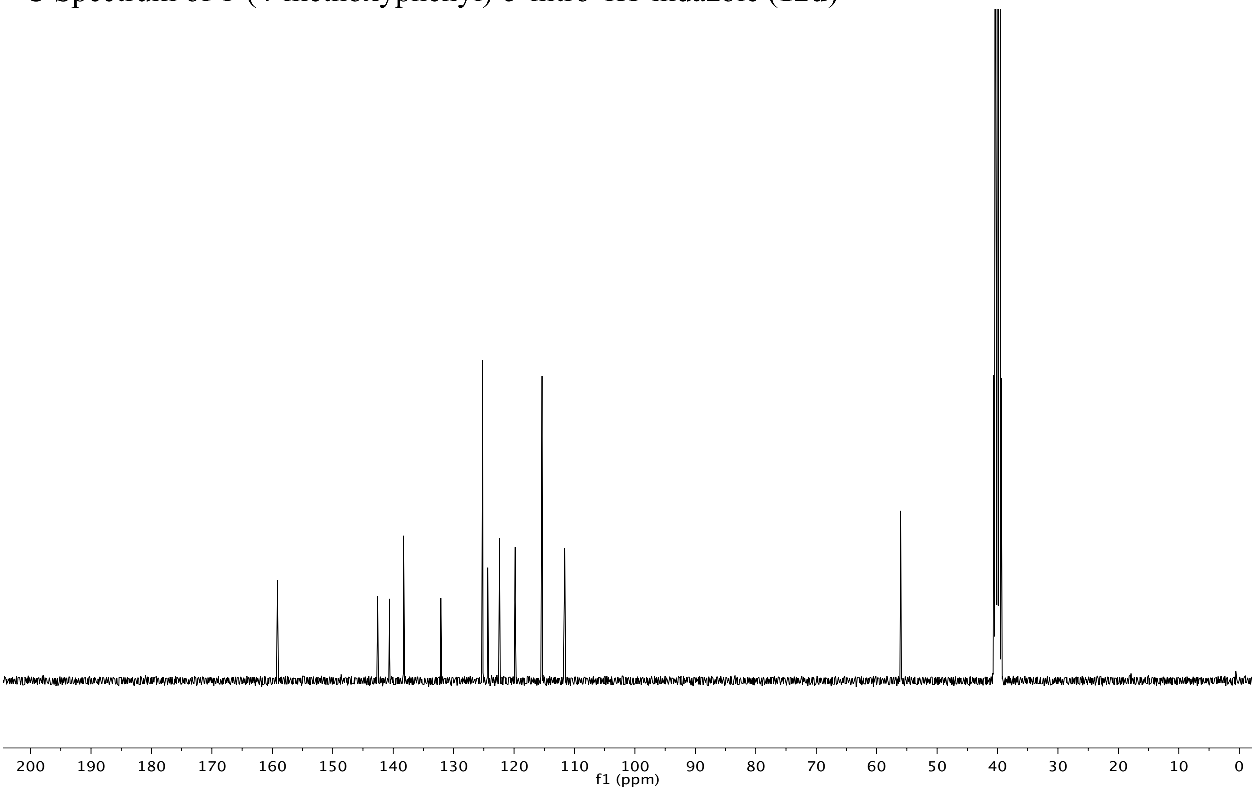

[1]H Spectrum of 1-(4-bromophenyl)-5-nitro-1*H*-indazole (**12e**)

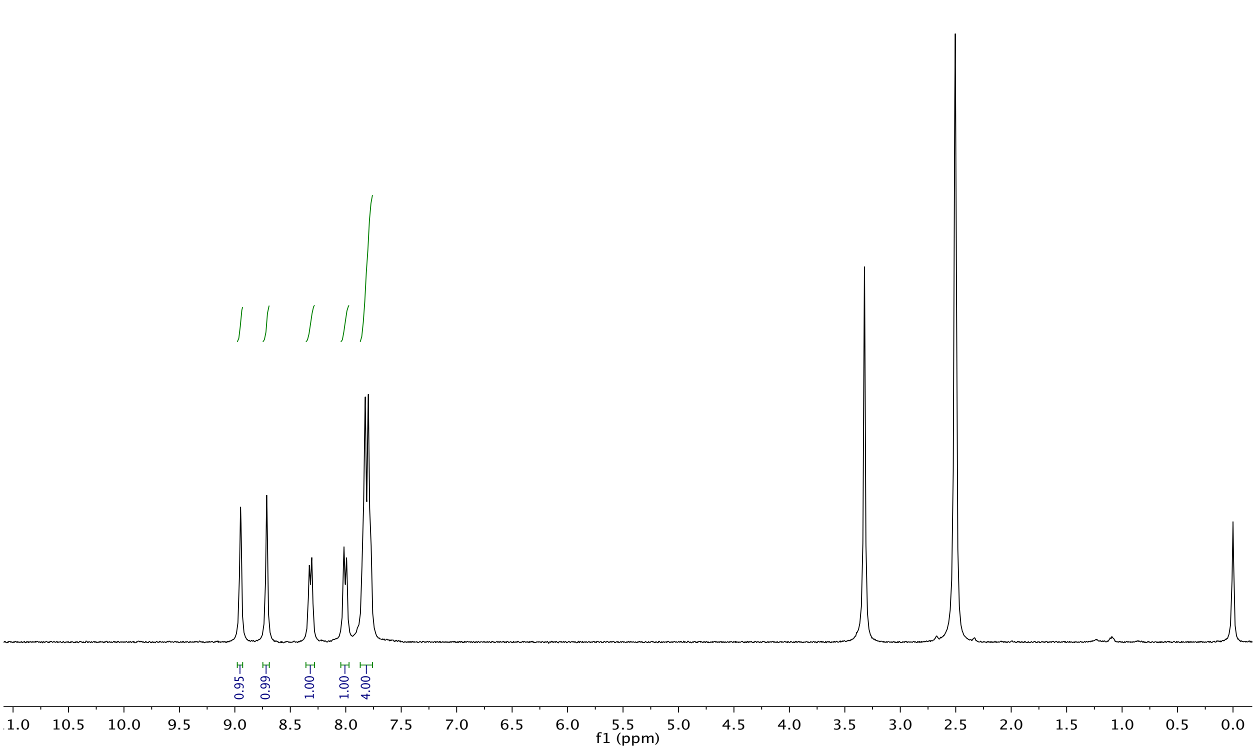

[13]C Spectrum of 1-(4-bromophenyl)-5-nitro-1*H*-indazole (**12e**)

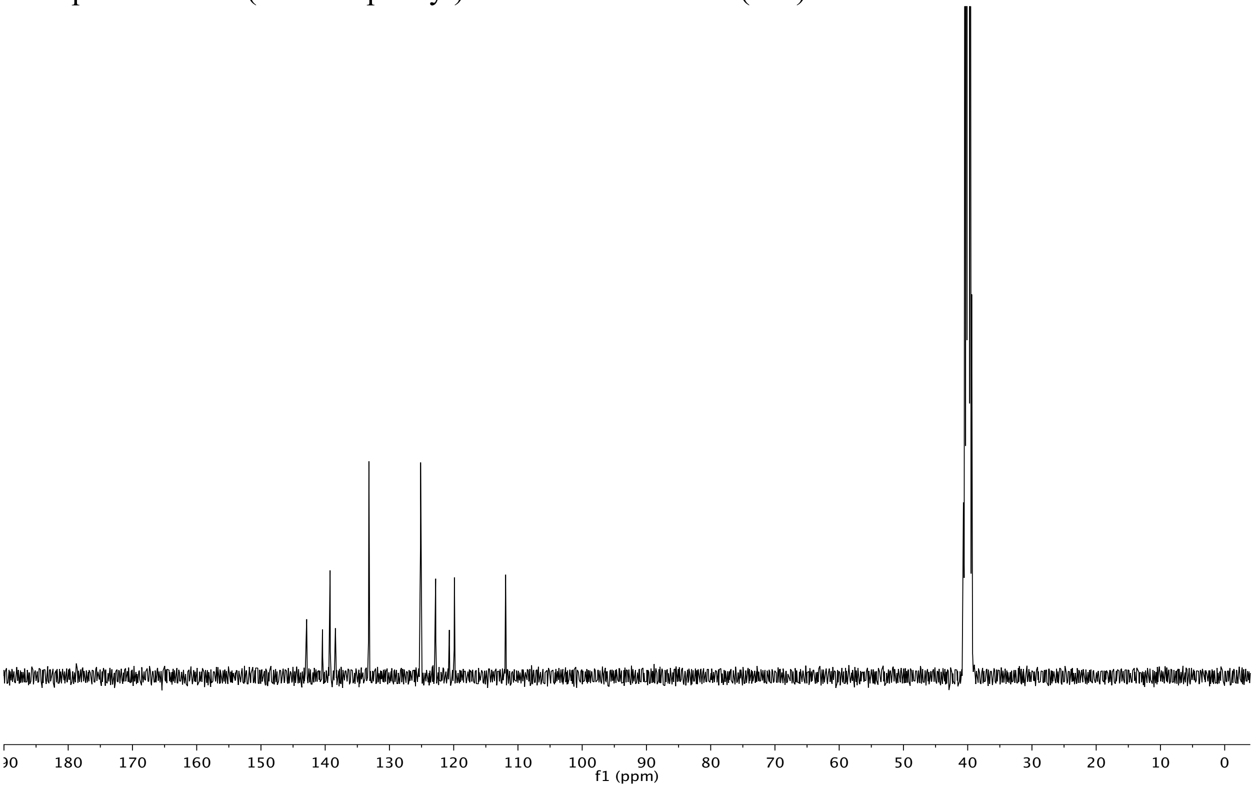

[1]H Spectrum of 1-(3-chlorophenyl)-5-nitro-1*H*-indazole (**12f**)

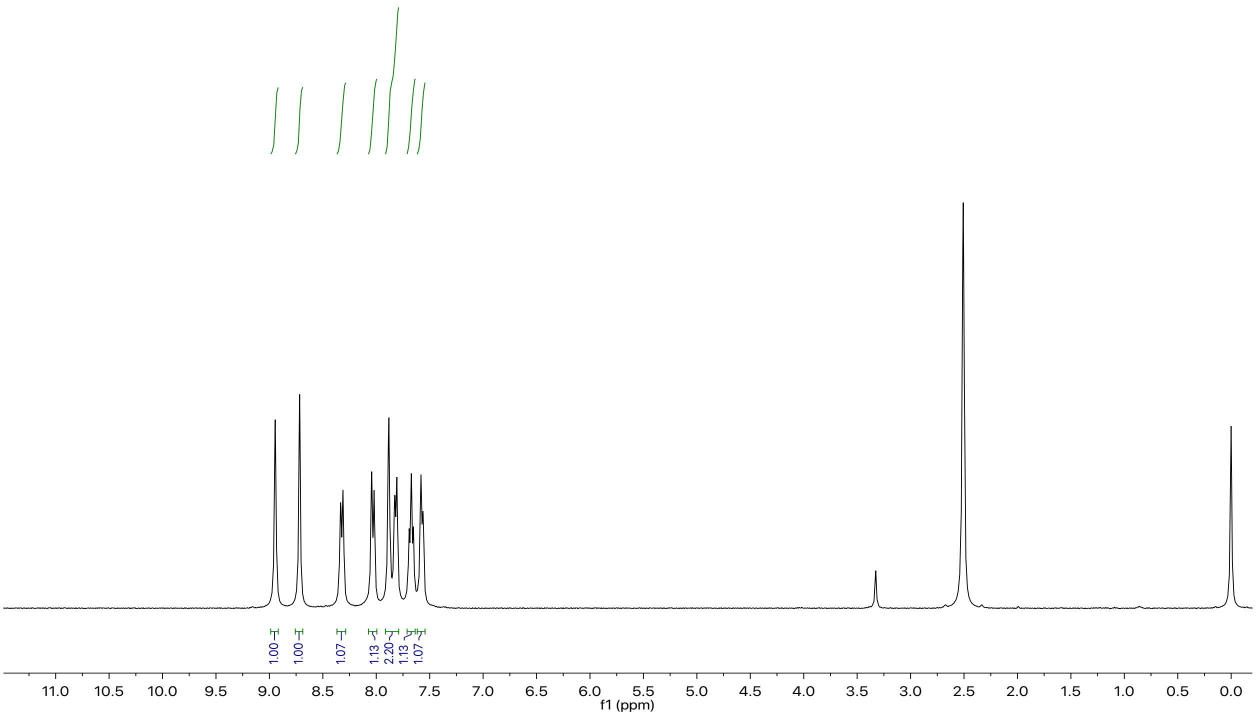

[13]C Spectrum of 1-(3-chlorophenyl)-5-nitro-1*H*-indazole (**12f**)

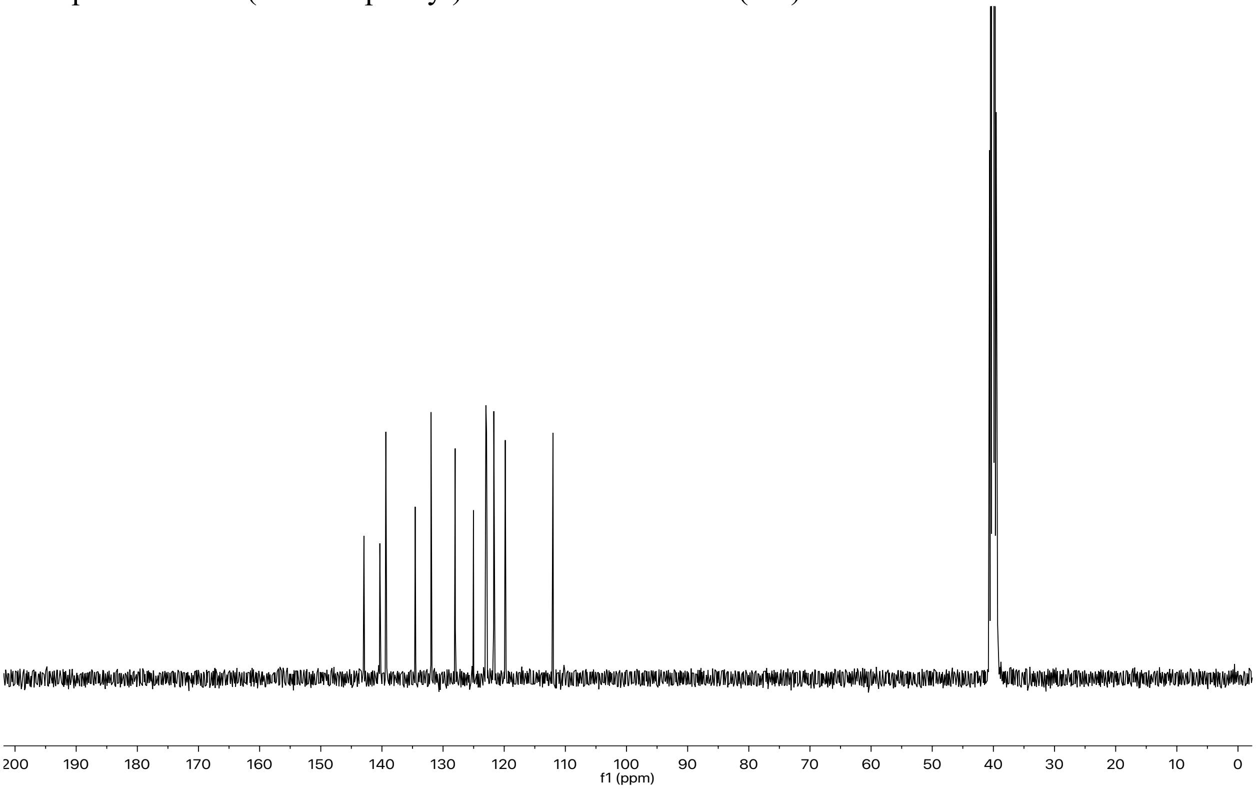

^{1}H Spectrum of 1-(4-chlorophenyl)-5-nitro-1*H*-indazole (**12g**)

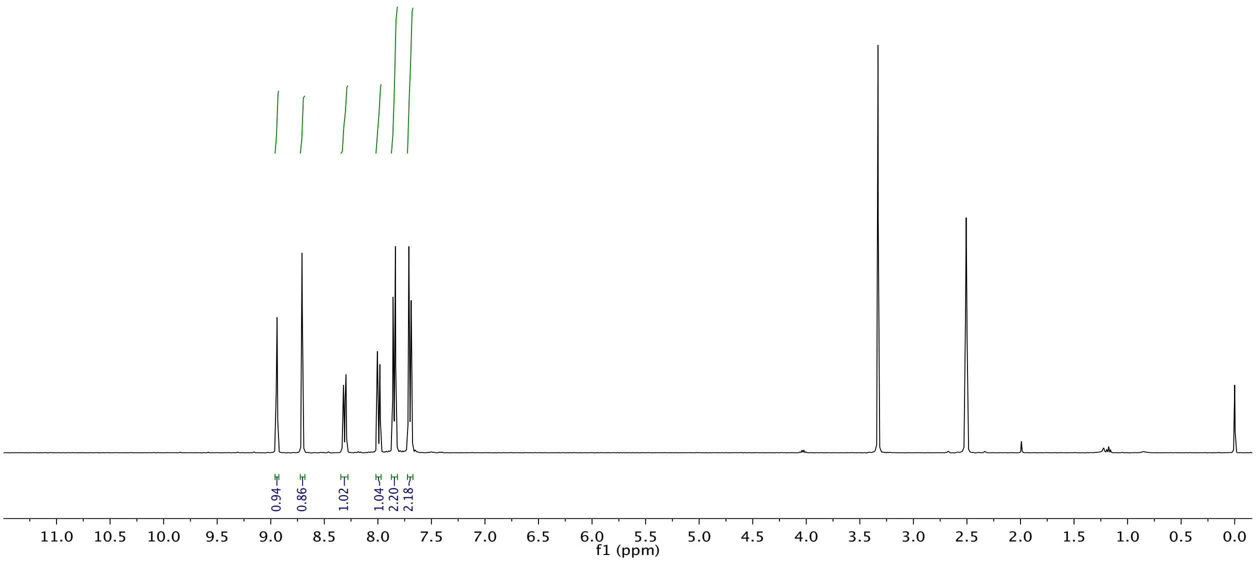

^{13}C Spectra of -(4-chlorophenyl)-5-nitro-1*H*-indazole (**12g**)

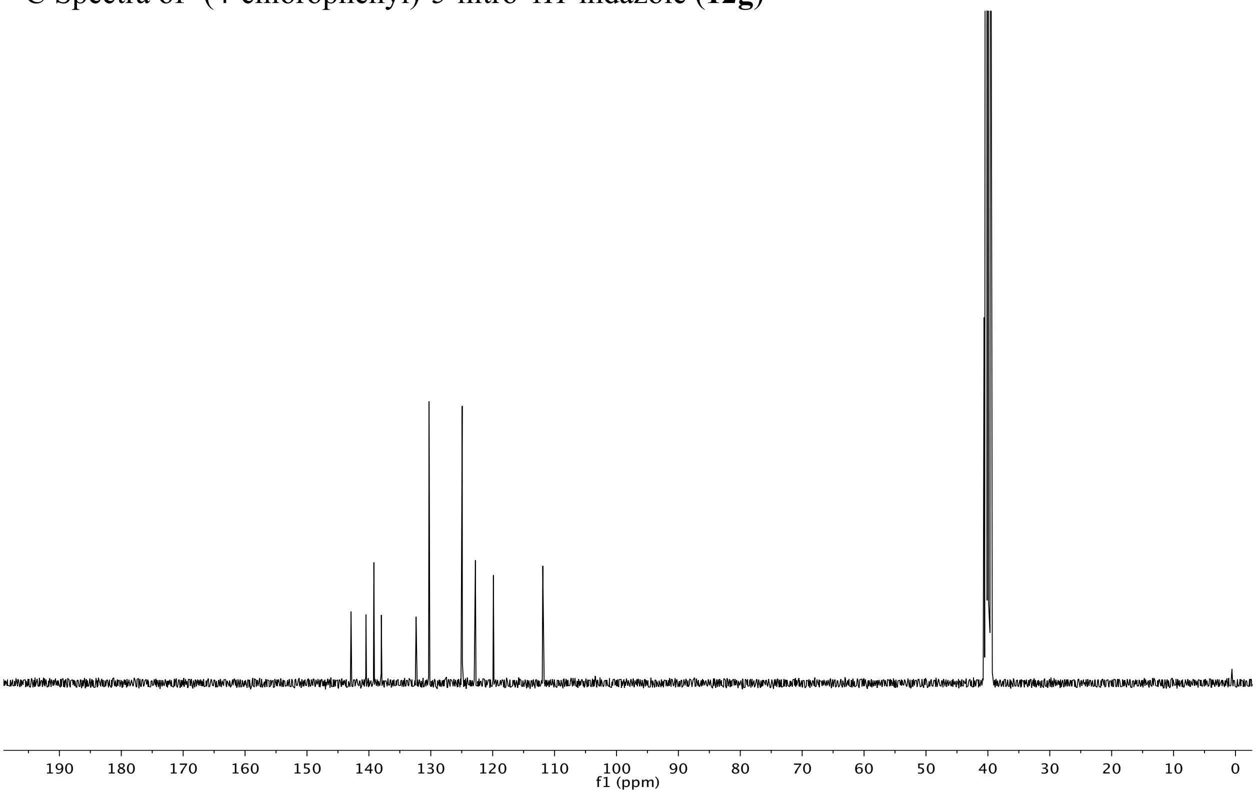

^{1}H Spectrum of 1-(2,4-dichlorophenyl)-5-nitro-1*H*-indazole (**12h**)

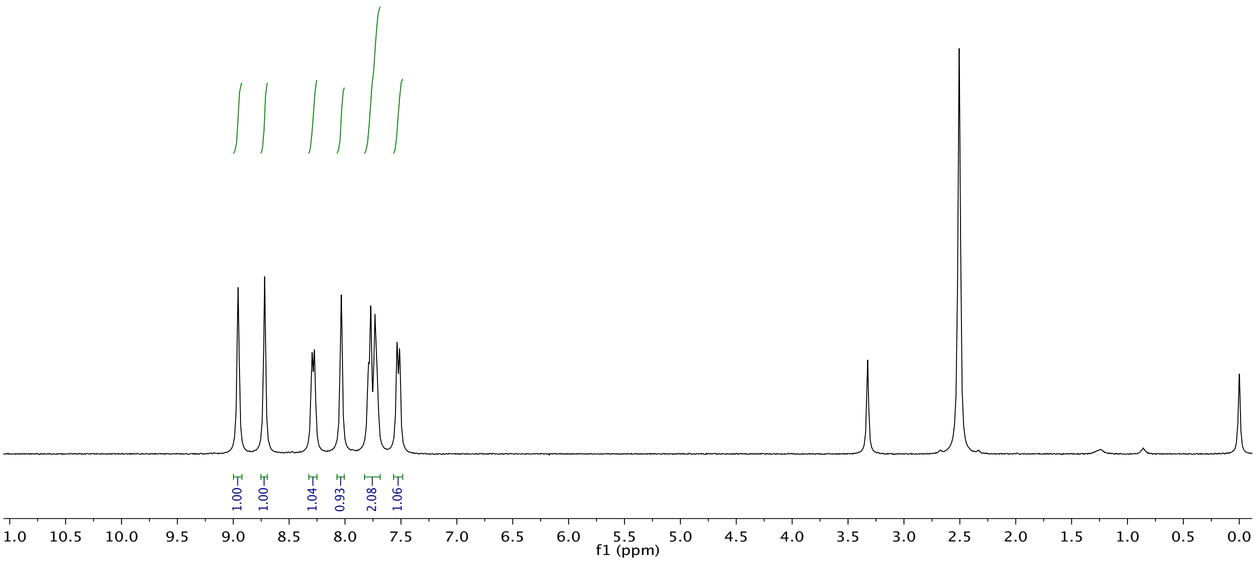

^{13}C Spectrum of 1-(2,4-dichlorophenyl)-5-nitro-1*H*-indazole (**12h**)

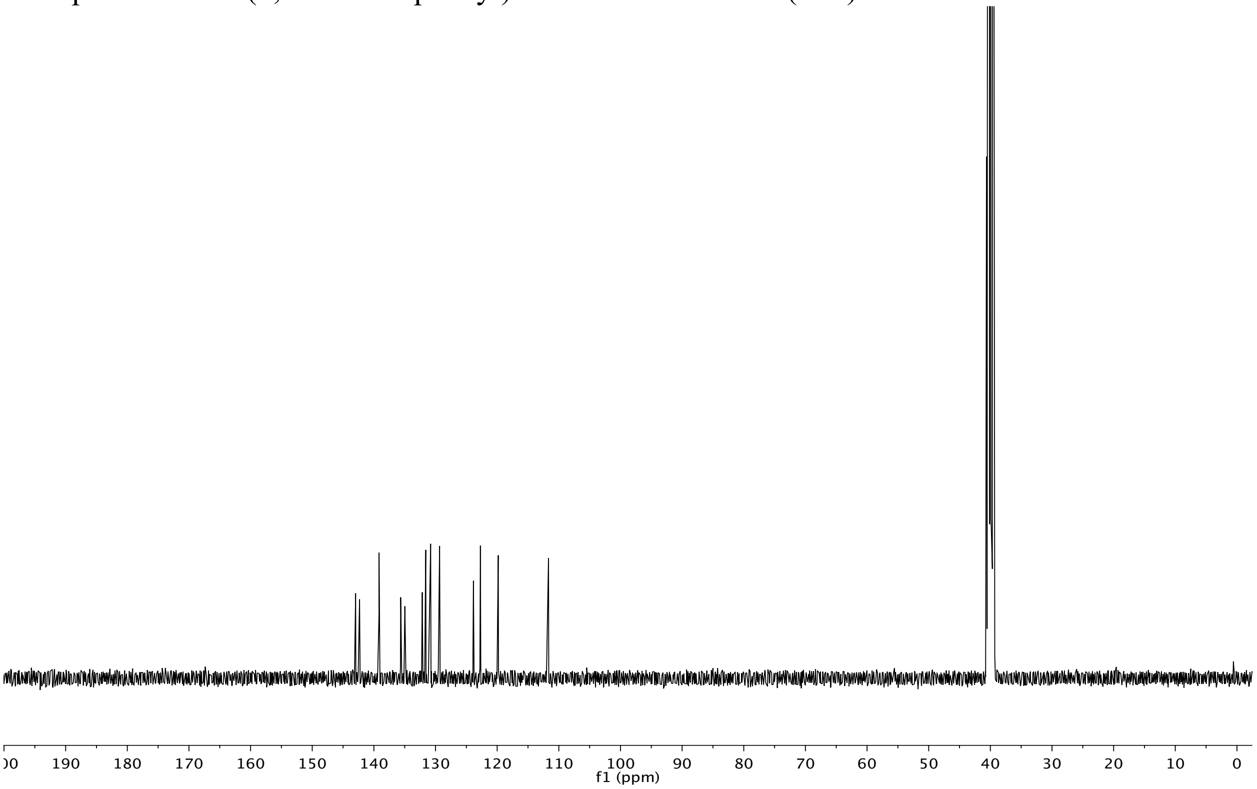

^{1}H Spectrum of 1-(3-(trifluoromethyl)phenyl)-5-nitro-1*H*-indazole (**12i**)

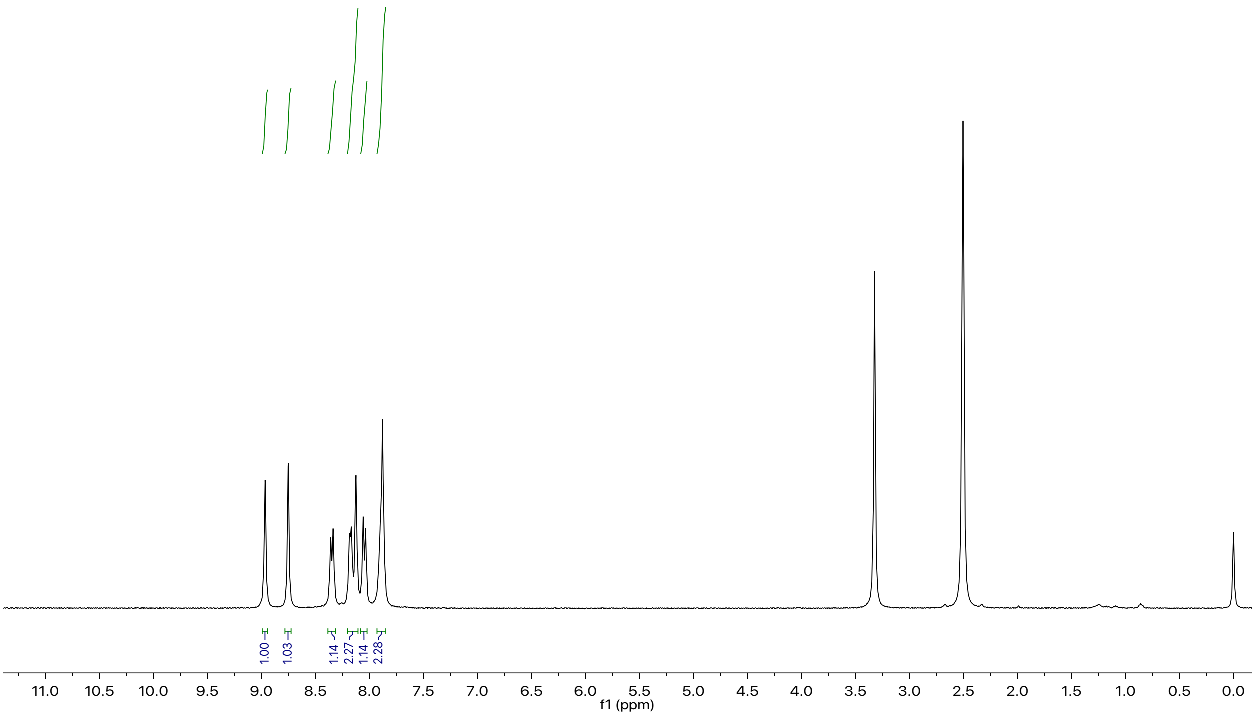

^{13}C Spectrum of 1-(3-(trifluoromethyl)phenyl)-5-nitro-1*H*-indazole (**12i**)

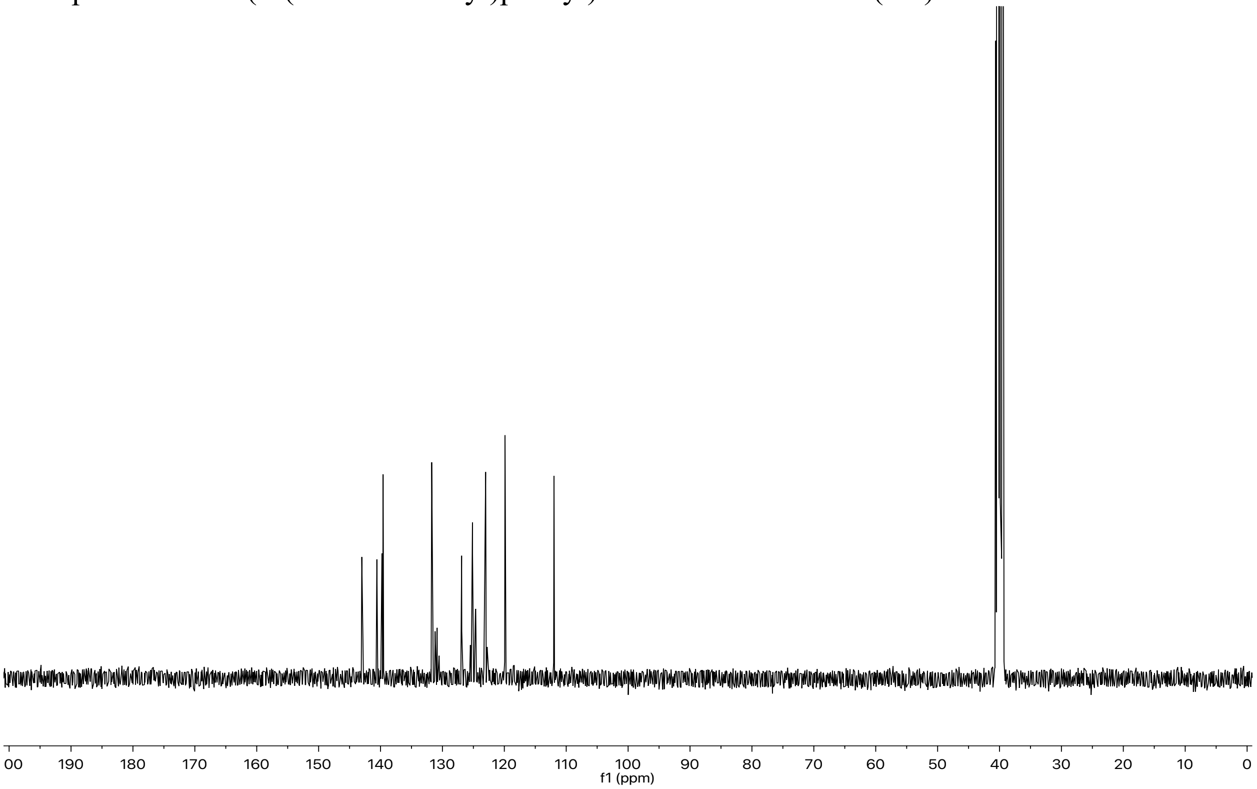

^{1}H Spectrum of 1-(4-(trifluoromethyl)phenyl)-5-nitro-1*H*-indazole (**12j**)

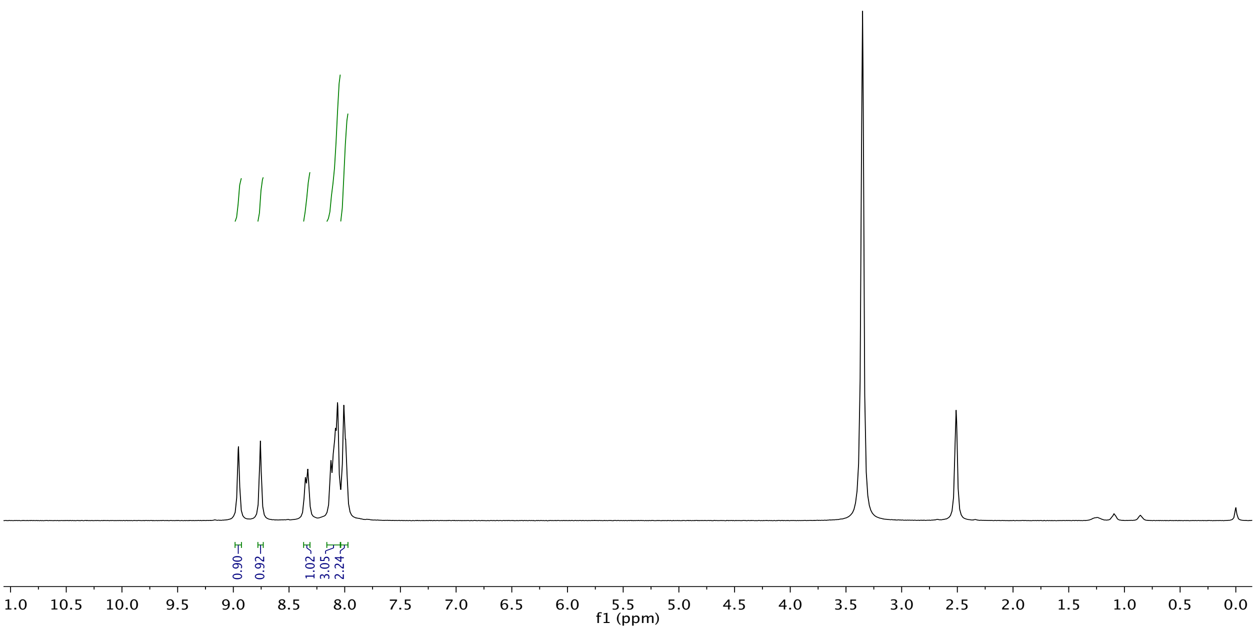

^{13}C Spectrum of 1-(4-(trifluoromethyl)phenyl)-5-nitro-1*H*-indazole (**12j**)

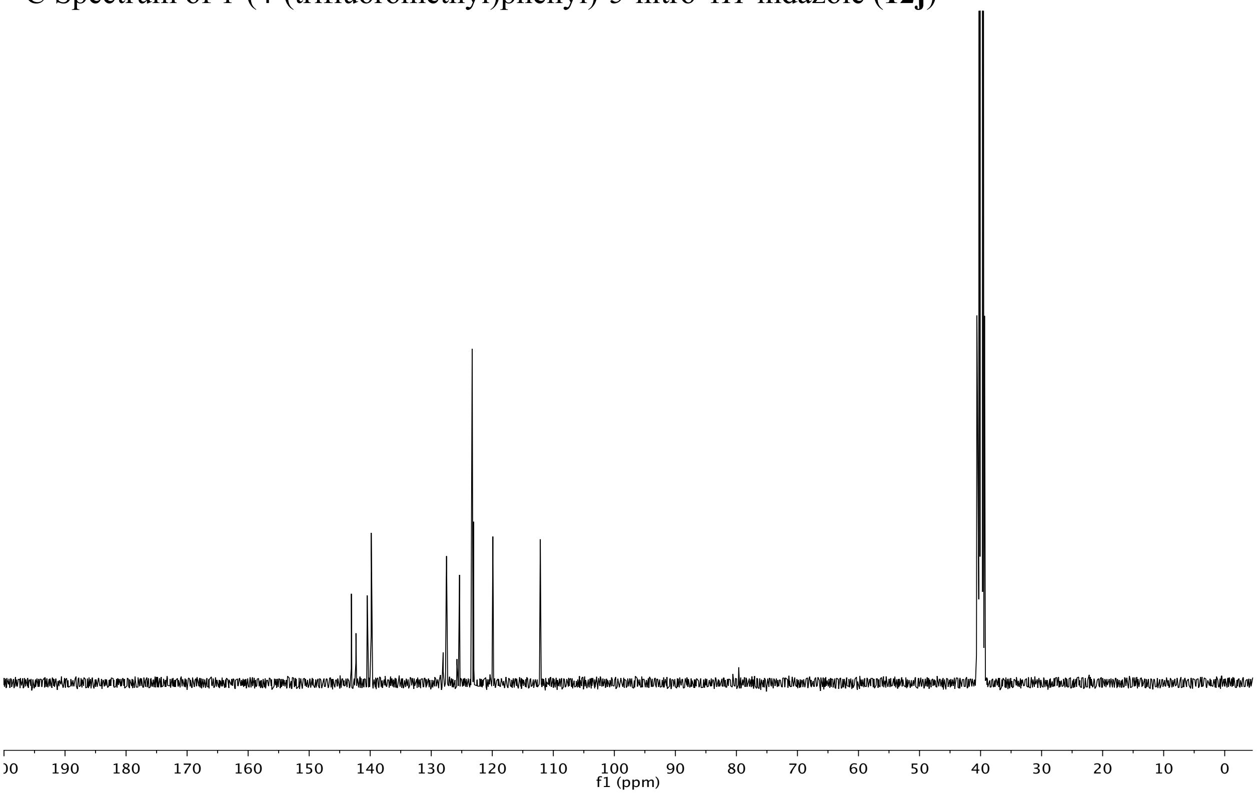

^{1}H Spectrum of 1-(4-cyanophenyl)-5-nitro-1*H*-indazole (**12k**)

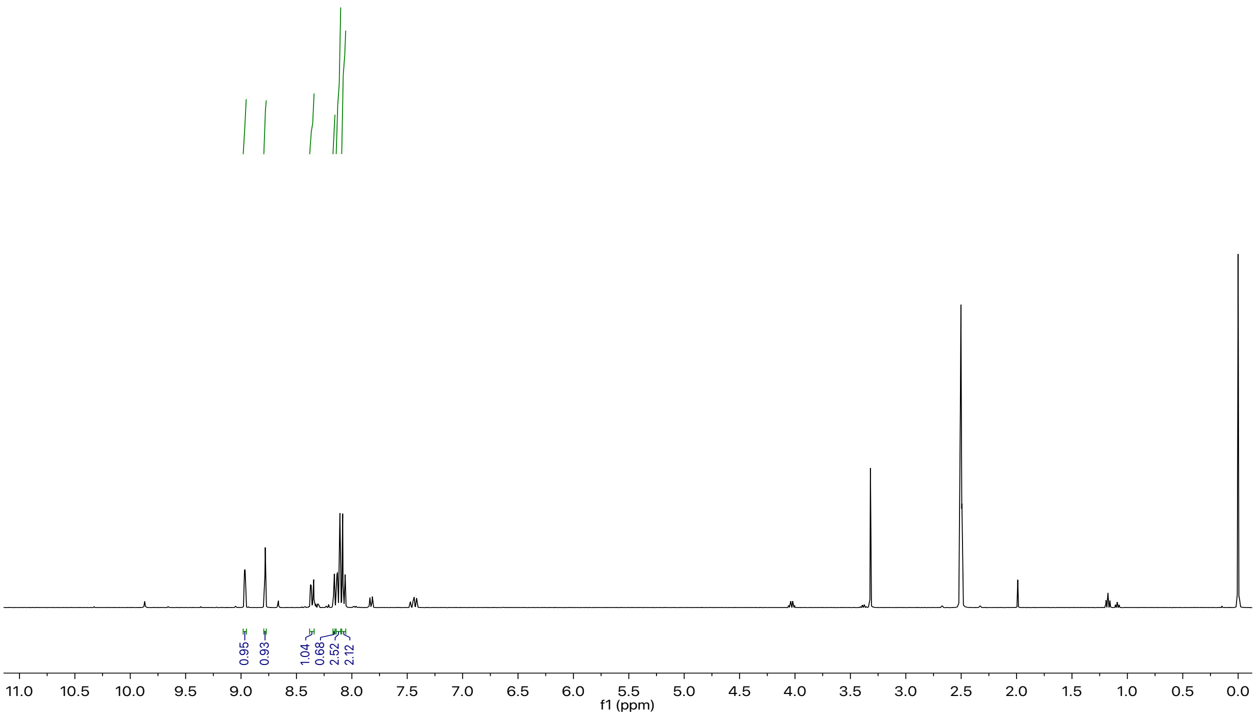

^{13}C Spectrum of 1-(4-cyanophenyl)-5-nitro-1*H*-indazole (**12k**)

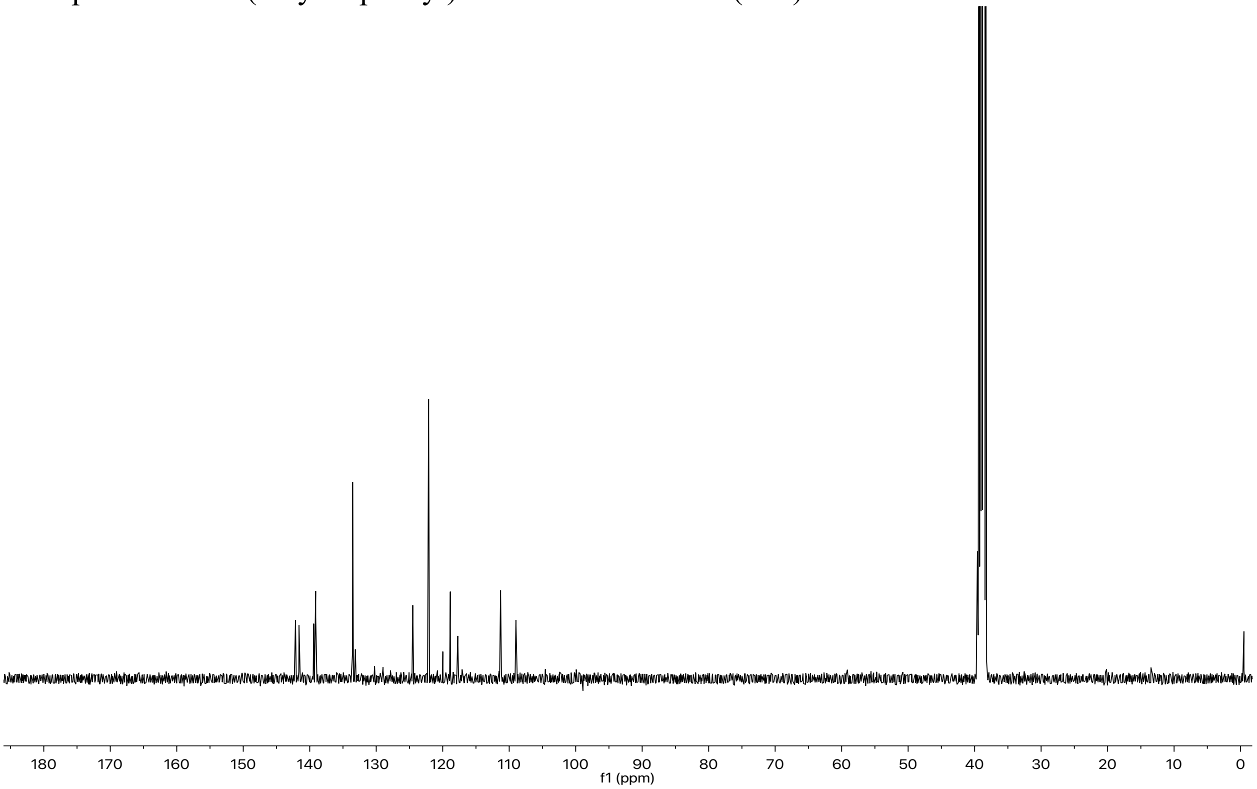

[1]H Spectrum of 4-(5-nitro-1*H*-indazol-1-yl)benzenesulfonamide (**12l**)

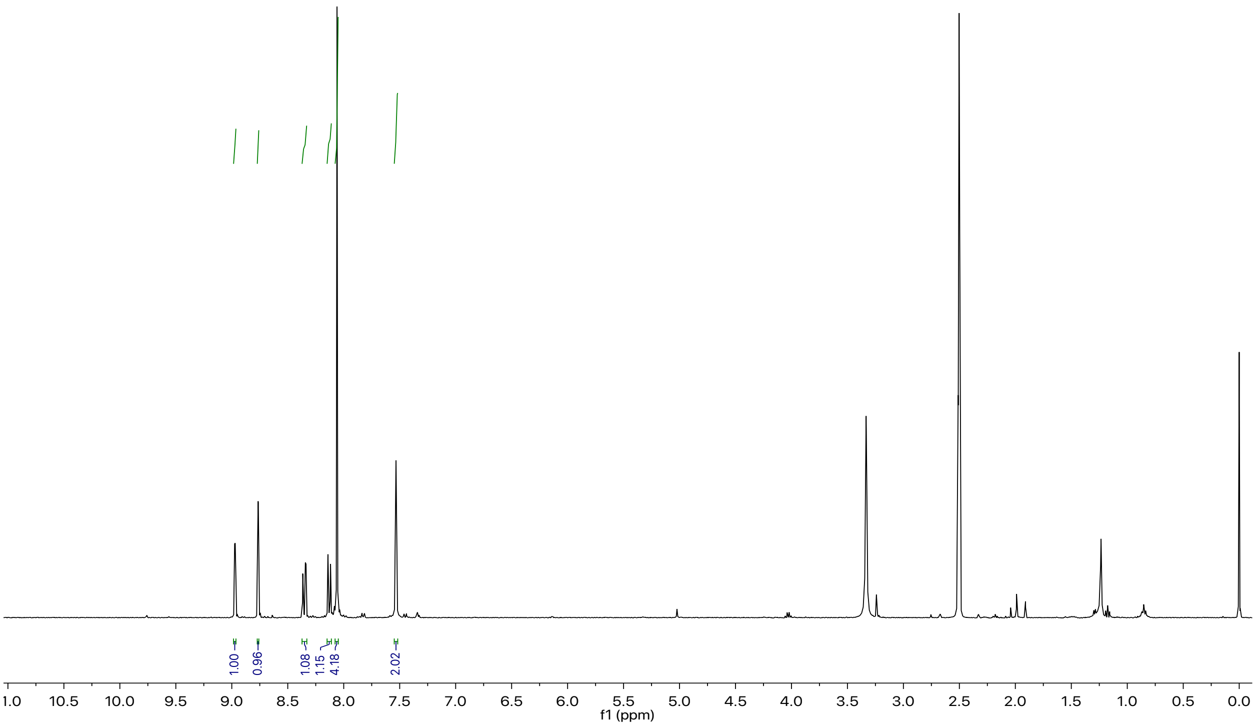

[13]C Spectrum of 4-(5-nitro-1*H*-indazol-1-yl)benzenesulfonamide (**12l**)

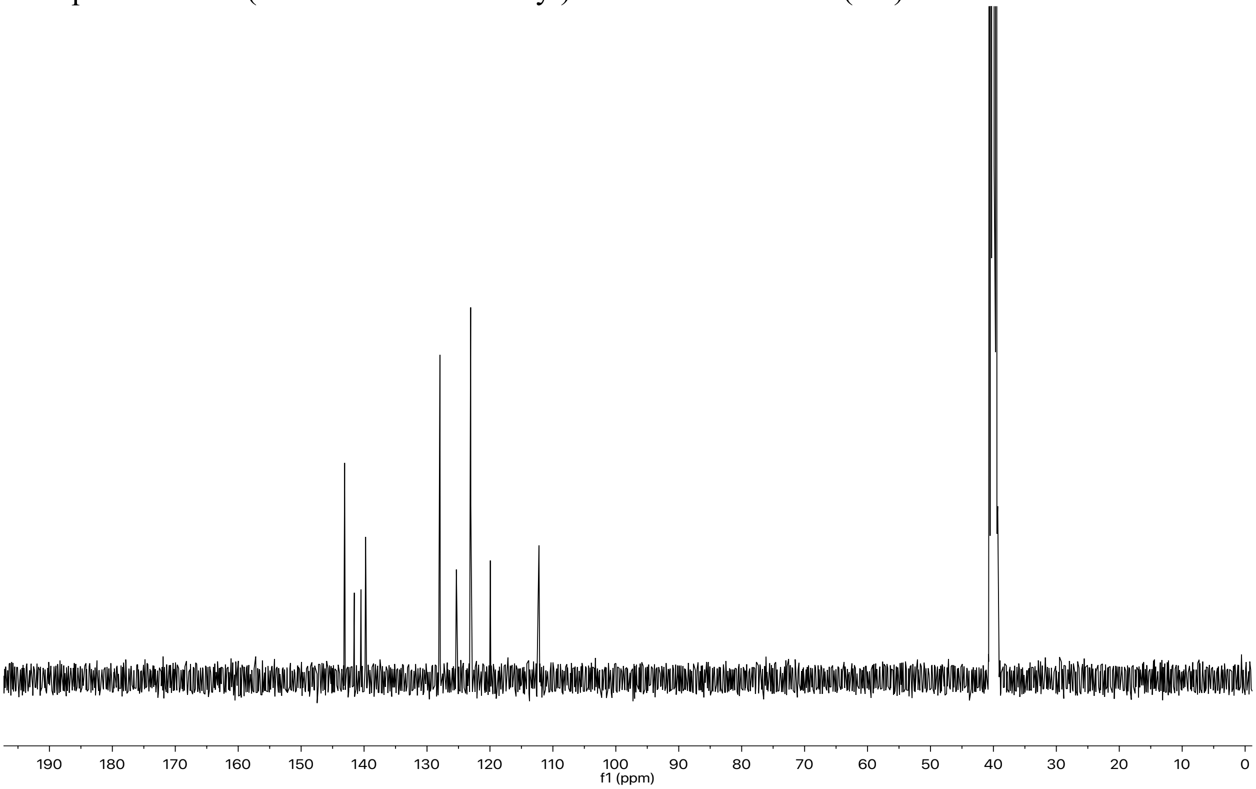

^{1}H Spectrum of 4-(5-nitro-1*H*-indazol-1-yl)benzoic acid (**12m**)

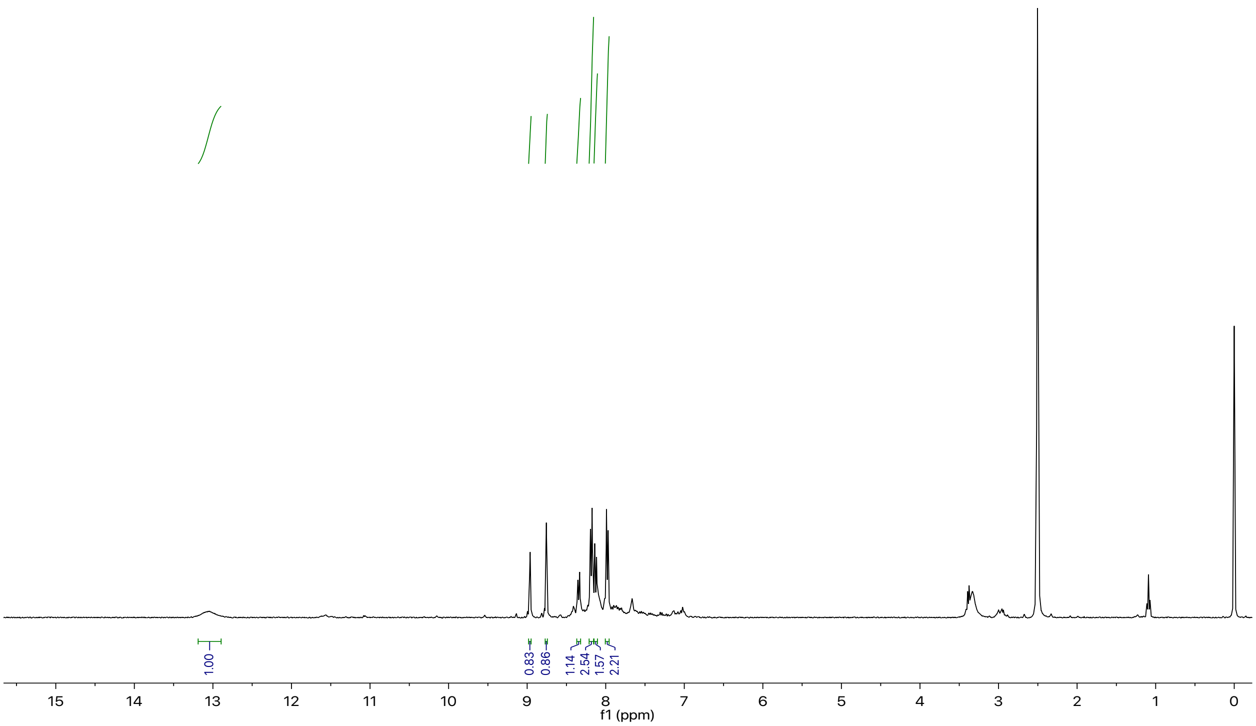

^{13}C Spectrum of 4-(5-nitro-1*H*-indazol-1-yl)benzoic acid (**12m**)

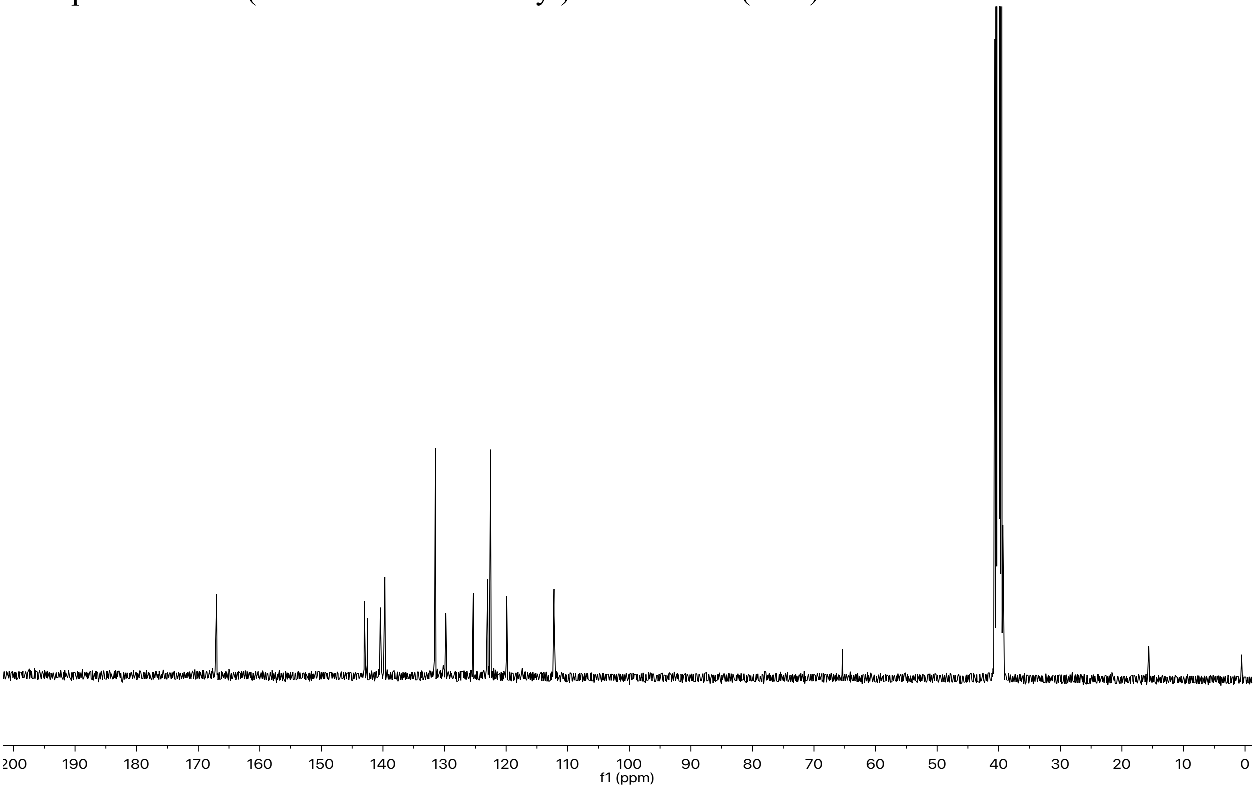

^{1}H Spectrum of 3-methyl-1-phenyl-1H-indazole (**16a**)

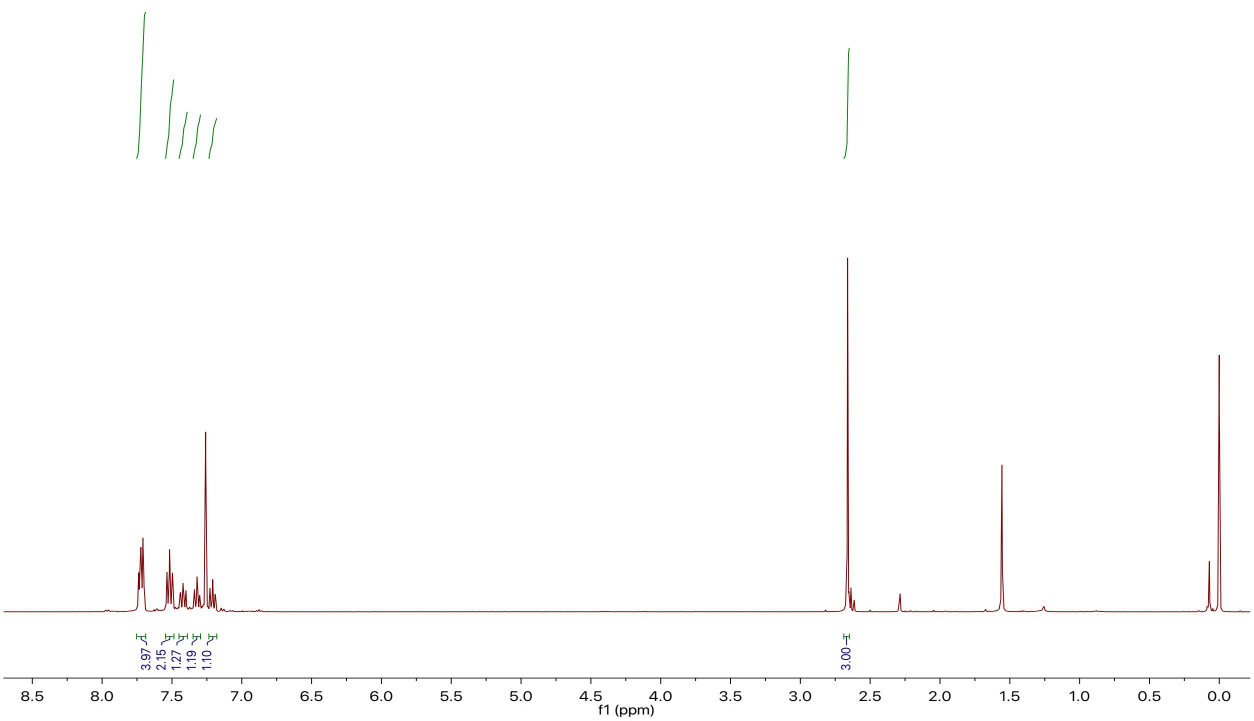

^{13}C Spectrum of 3-methyl-1-phenyl-1H-indazole (**16a**)

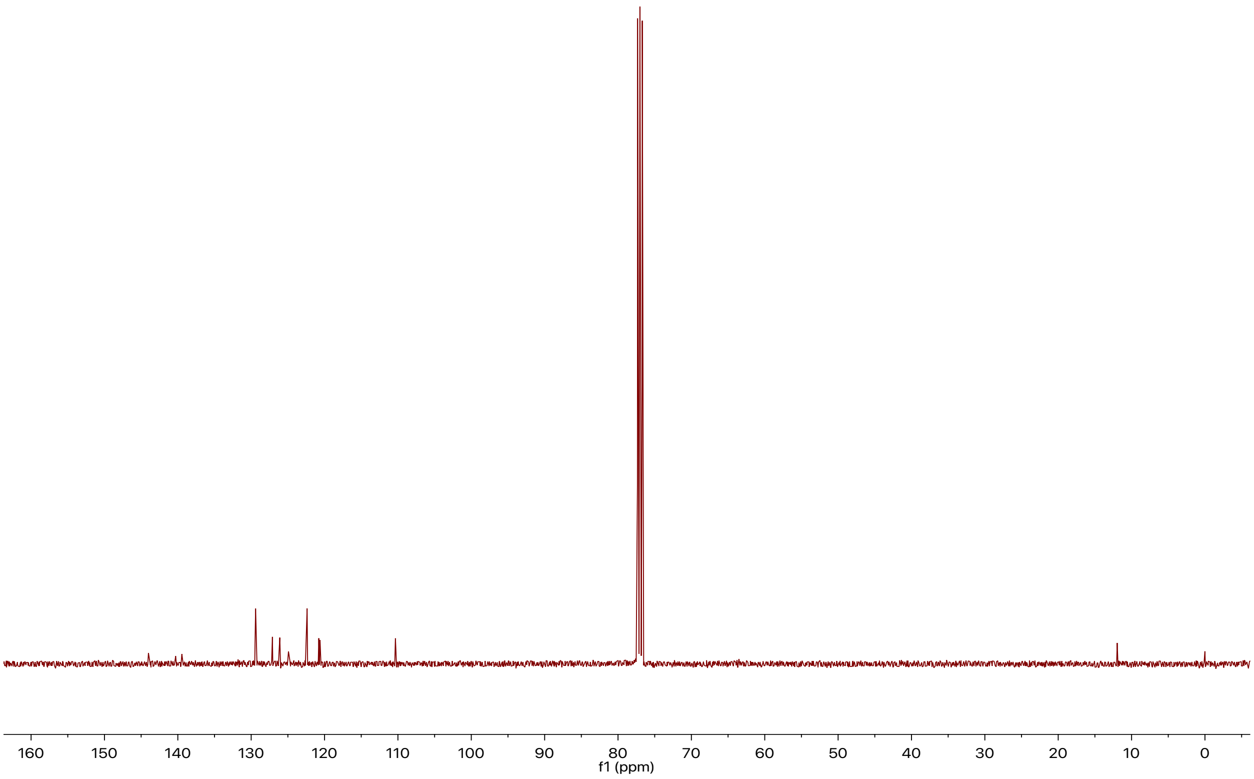

[1]H Spectrum of 1-(4-methoxyphenyl)-3-methyl-1*H*-indazole (**16d**)

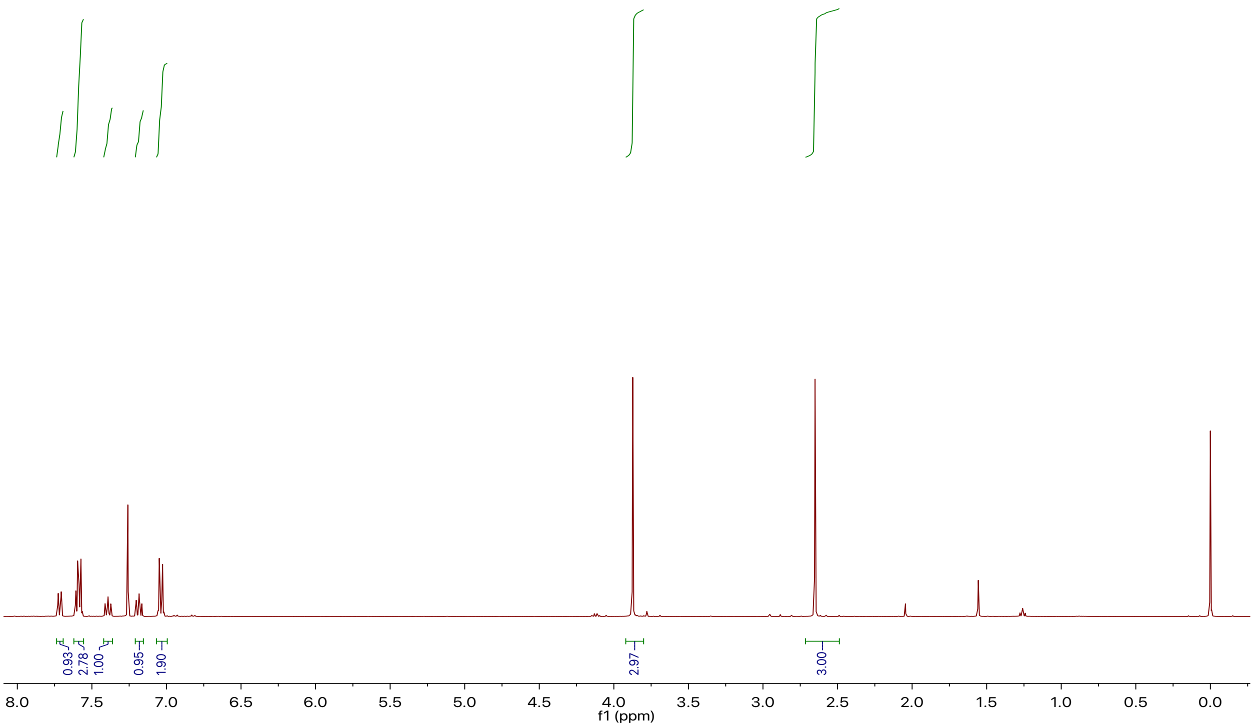

[13]C Spectrum of 1-(4-methoxyphenyl)-3-methyl-1*H*-indazole (**16d**)

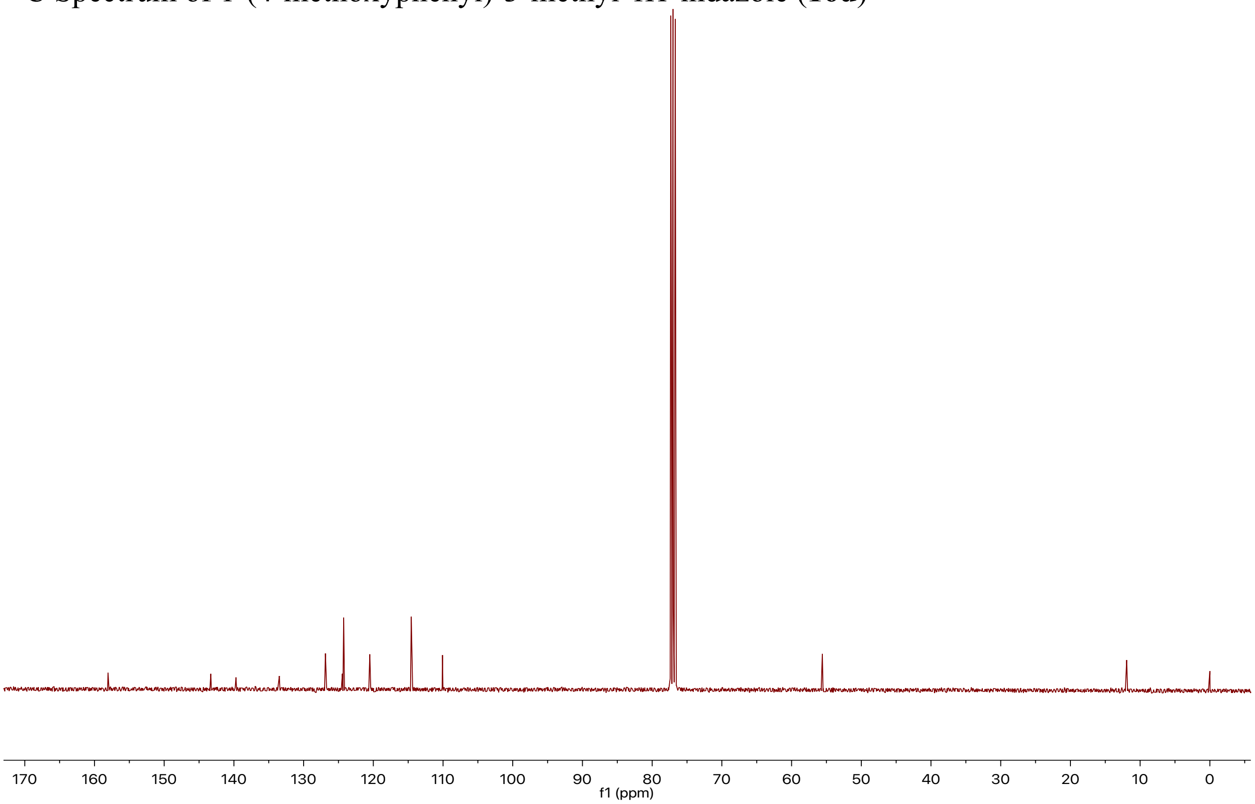

^{1}H Spectrum of 4-(3-methyl-1*H*-indazol-1-yl)benzonitrile (**16k**)

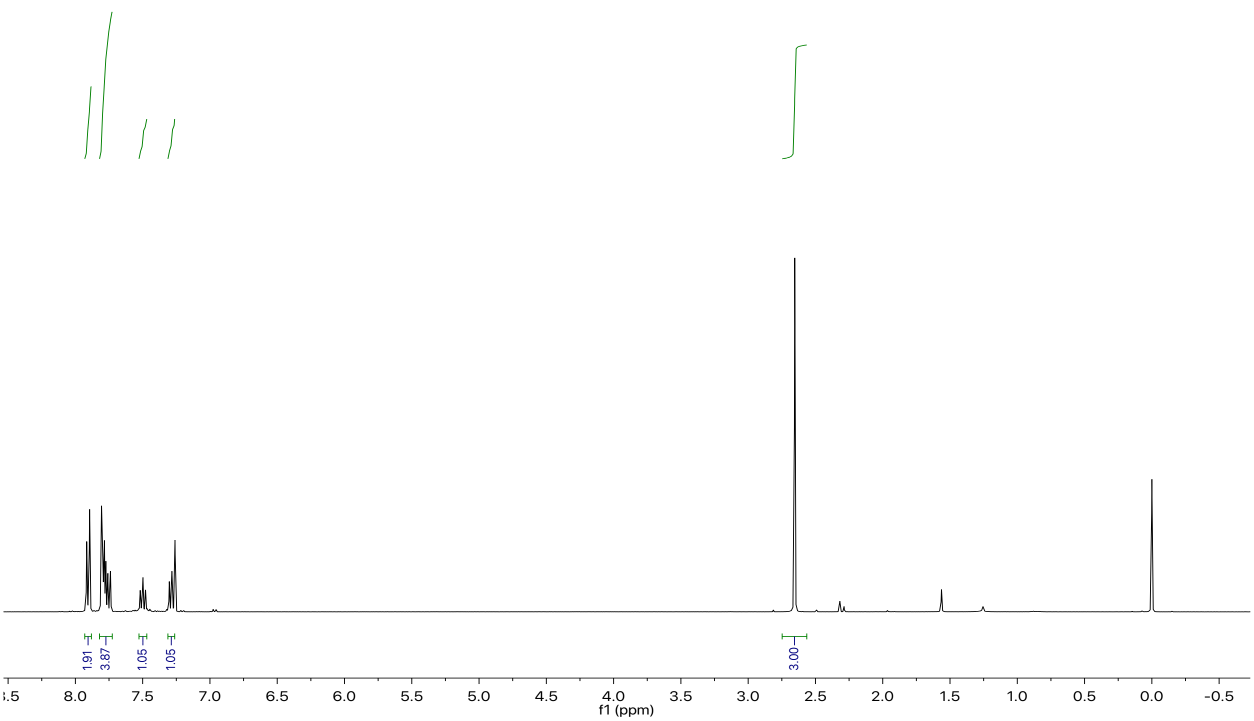

^{13}C Spectrum of 4-(3-methyl-1*H*-indazol-1-yl)benzonitrile (**16k**)

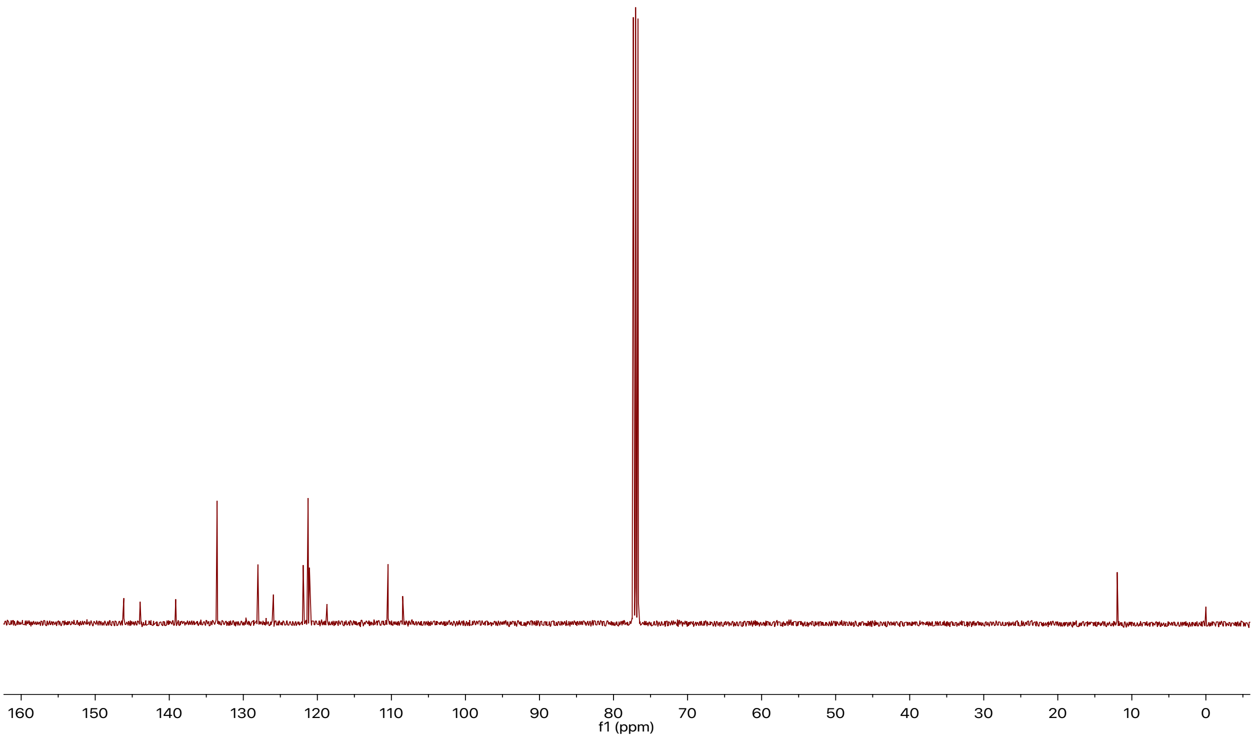

^{1}H Spectrum of 1-phenyl-1*H*-indazole (**17a**)

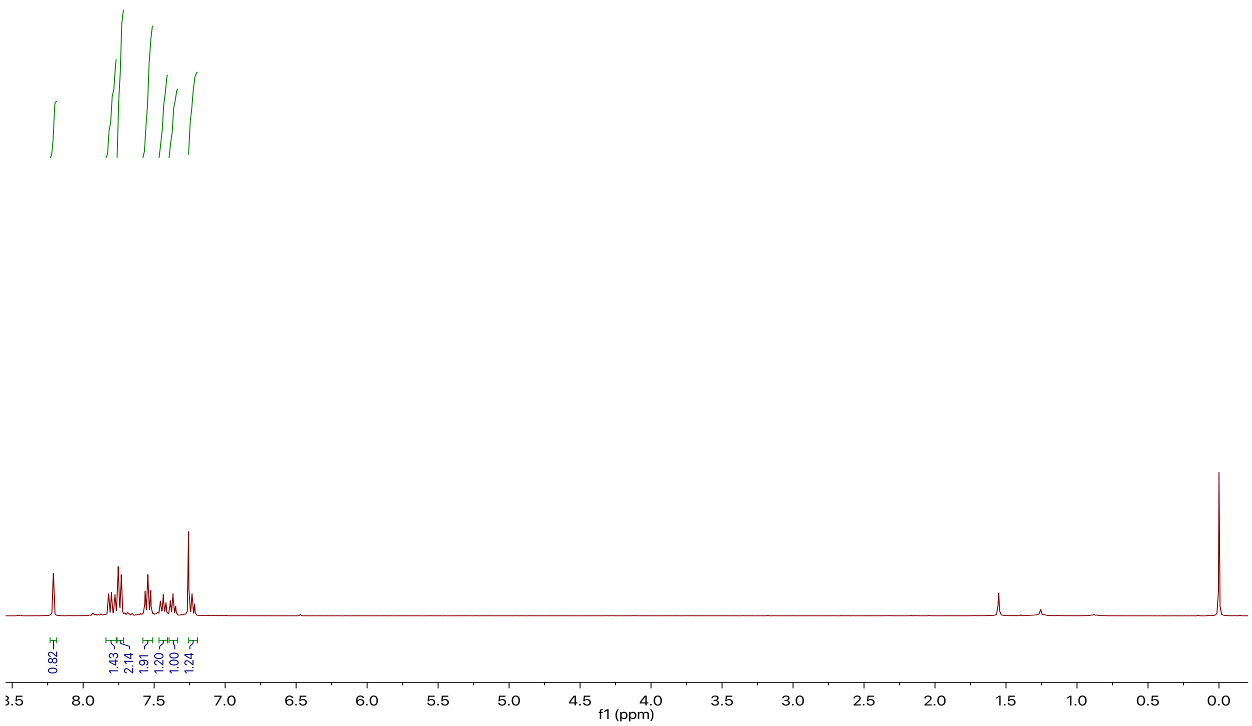

^{13}C Spectrum of 1-phenyl-1*H*-indazole (**17a**)

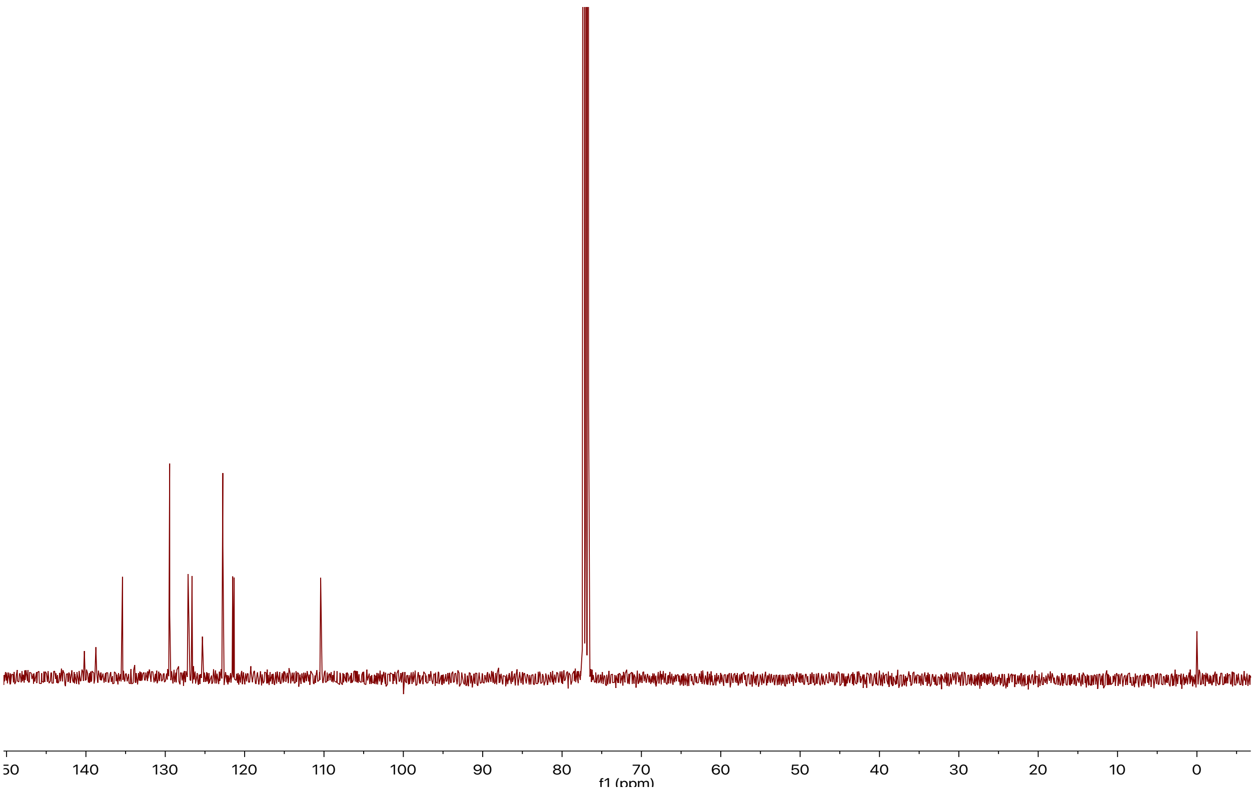

[1]H Spectrum of 1-(4-methoxyphenyl)-1*H*-indazole (**17d**)

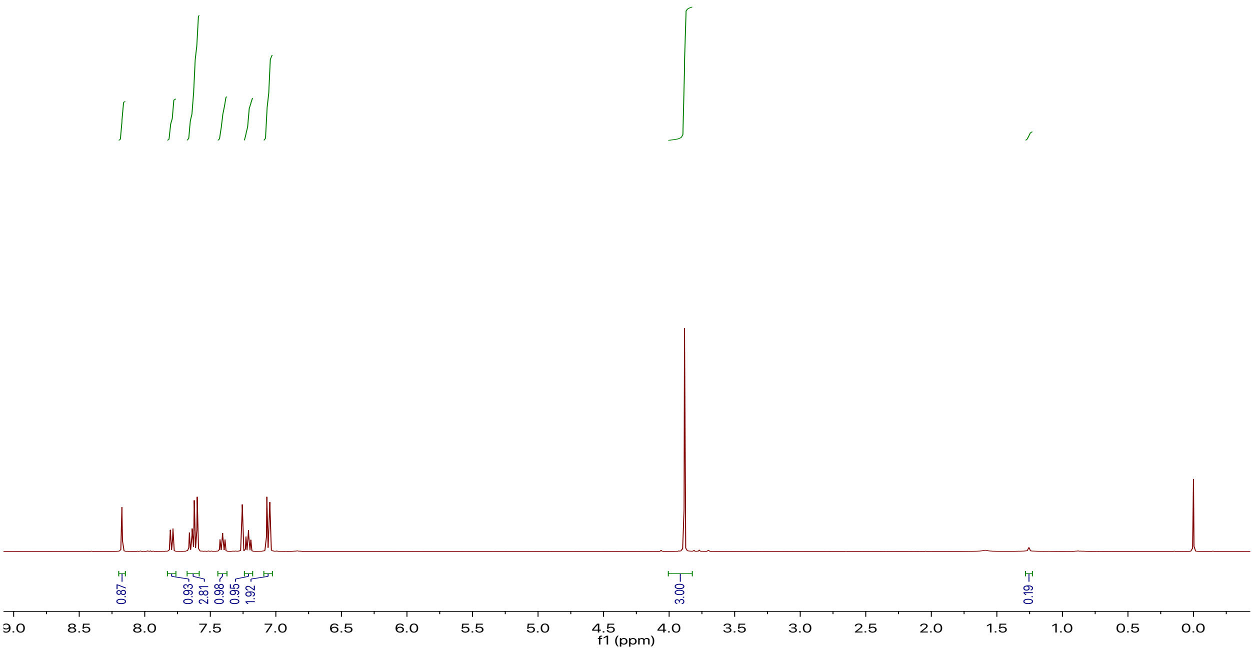

[13]C Spectra of 1-(4-methoxyphenyl)-1*H*-indazole (**17d**)

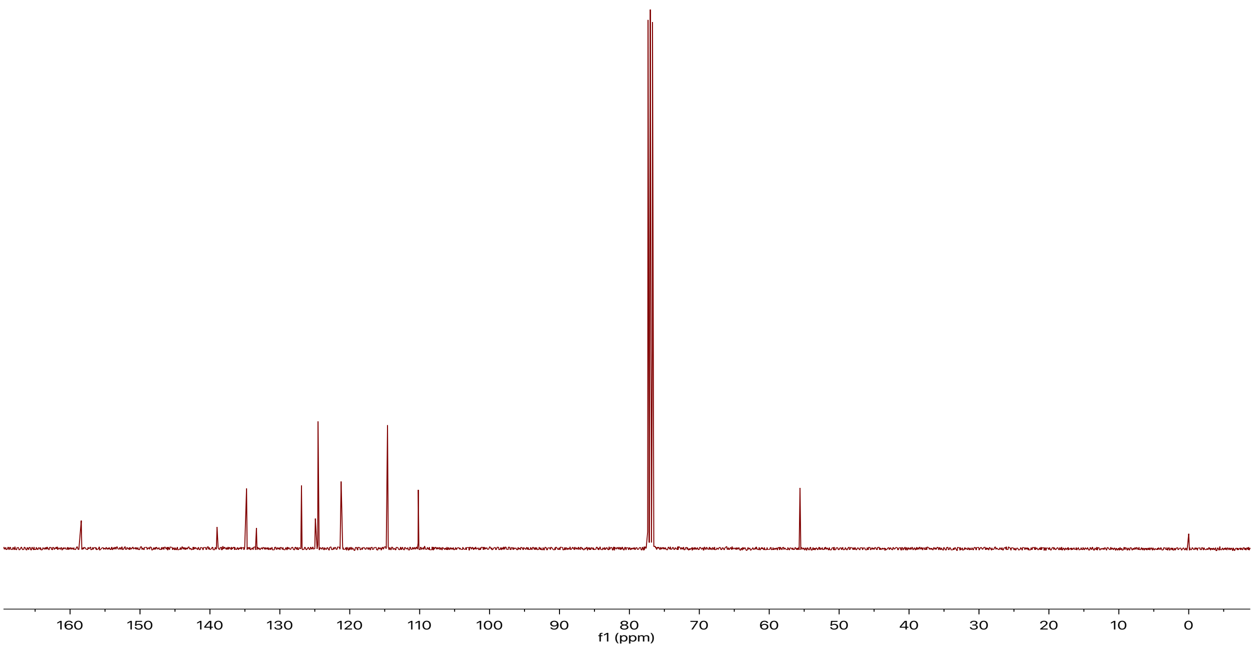

[1]H Spectrum of 4-(1*H*-indazol-1-yl)benzonitrile (**17k**)

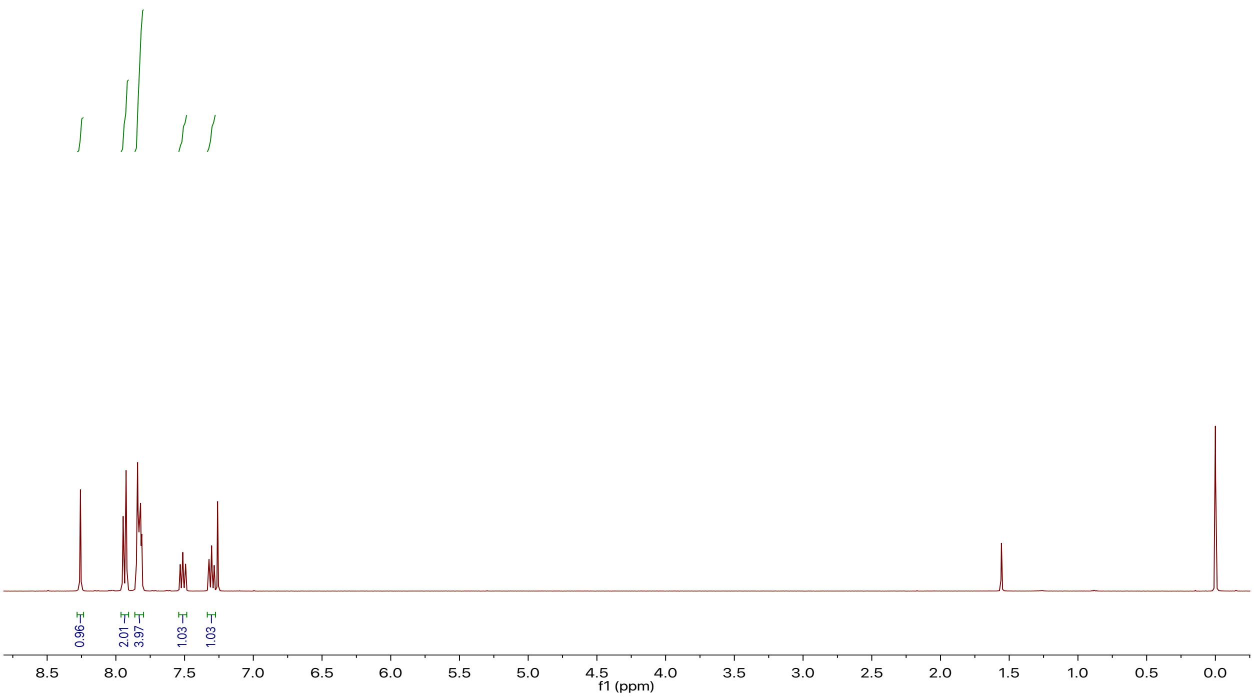

[13]C Spectrum of 4-(1*H*-indazol-1-yl)benzonitrile (**17k**)

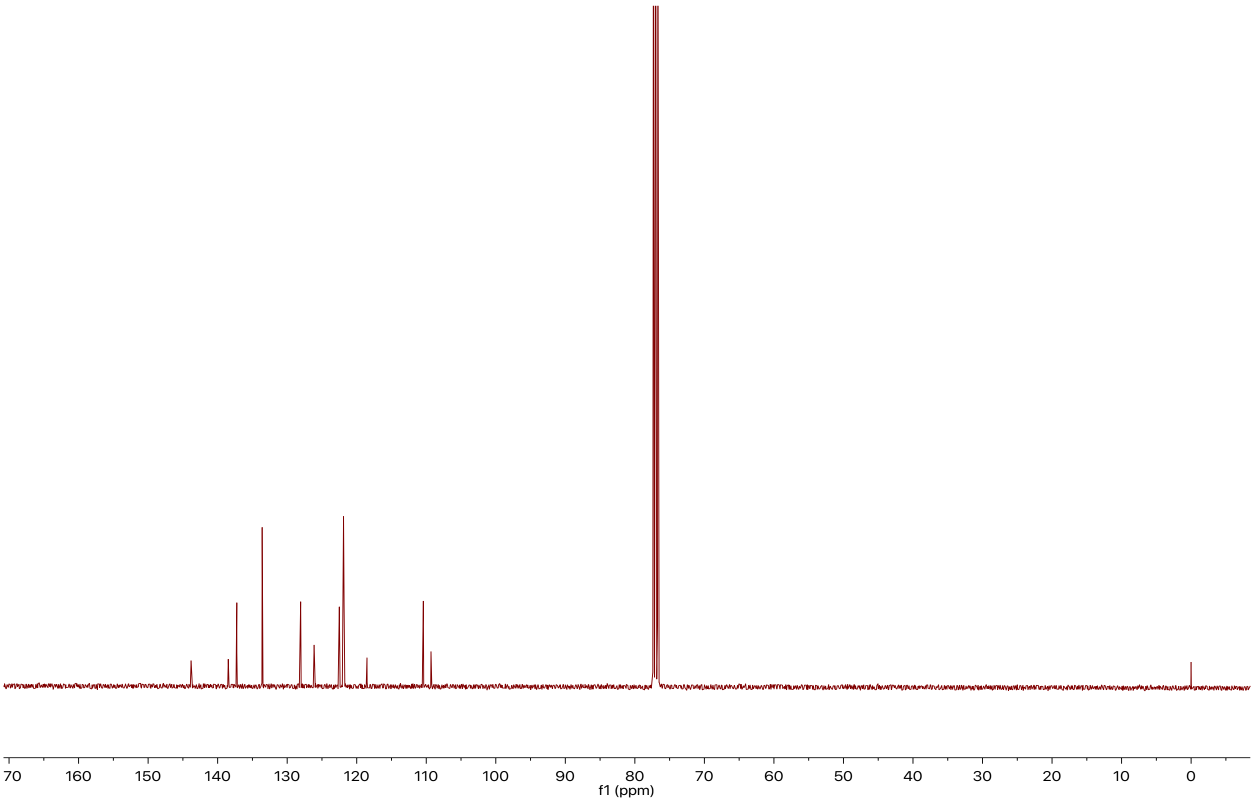

[1]H Spectrum of 1-phenyl-1*H*-pyrazolo[3,4-*b*]pyridine (**18a**)

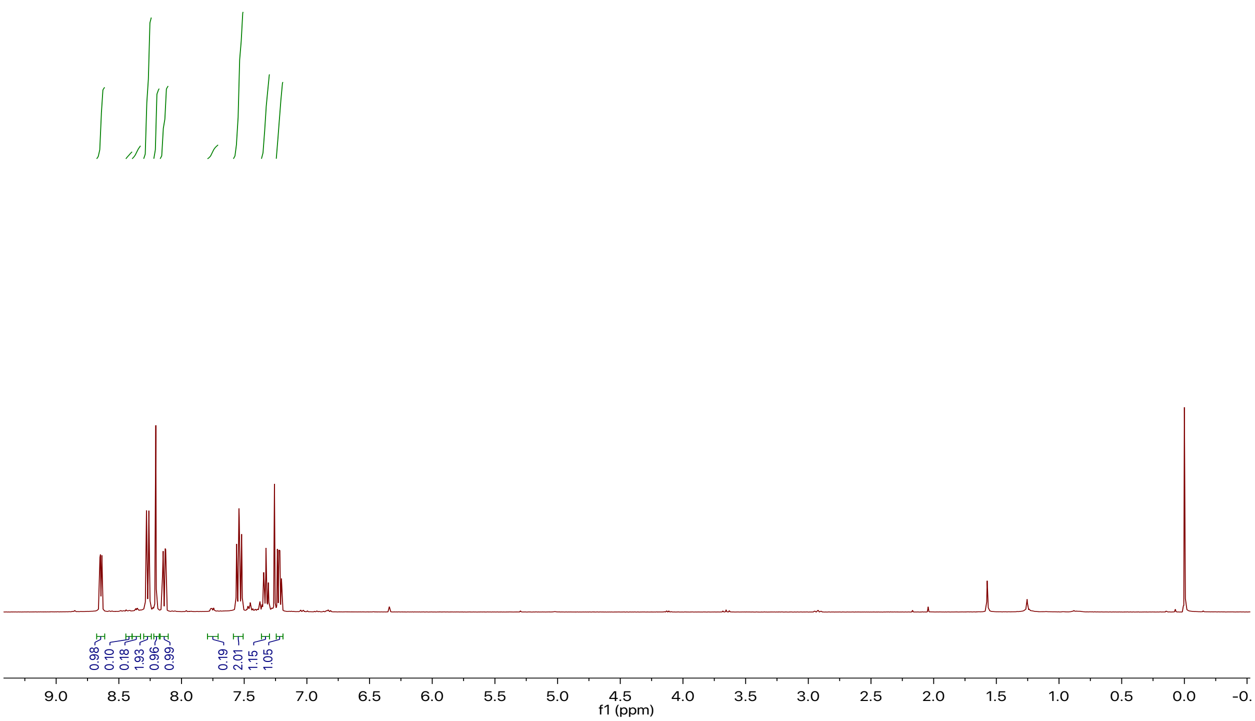

[13]C Spectrum of 1-phenyl-1*H*-pyrazolo[3,4-*b*]pyridine (**18a**)

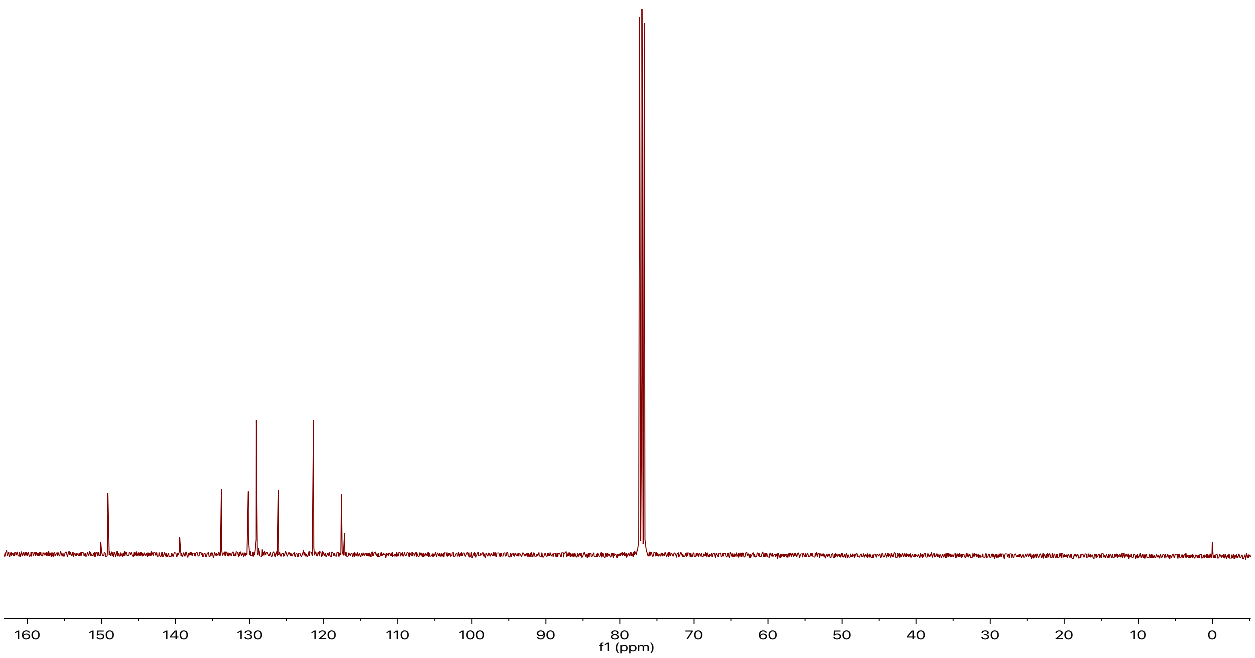

[1]H Spectrum of 1-(4-methoxyphenyl)-1*H*-pyrazolo[3,4-*b*]pyridine (**18d**)

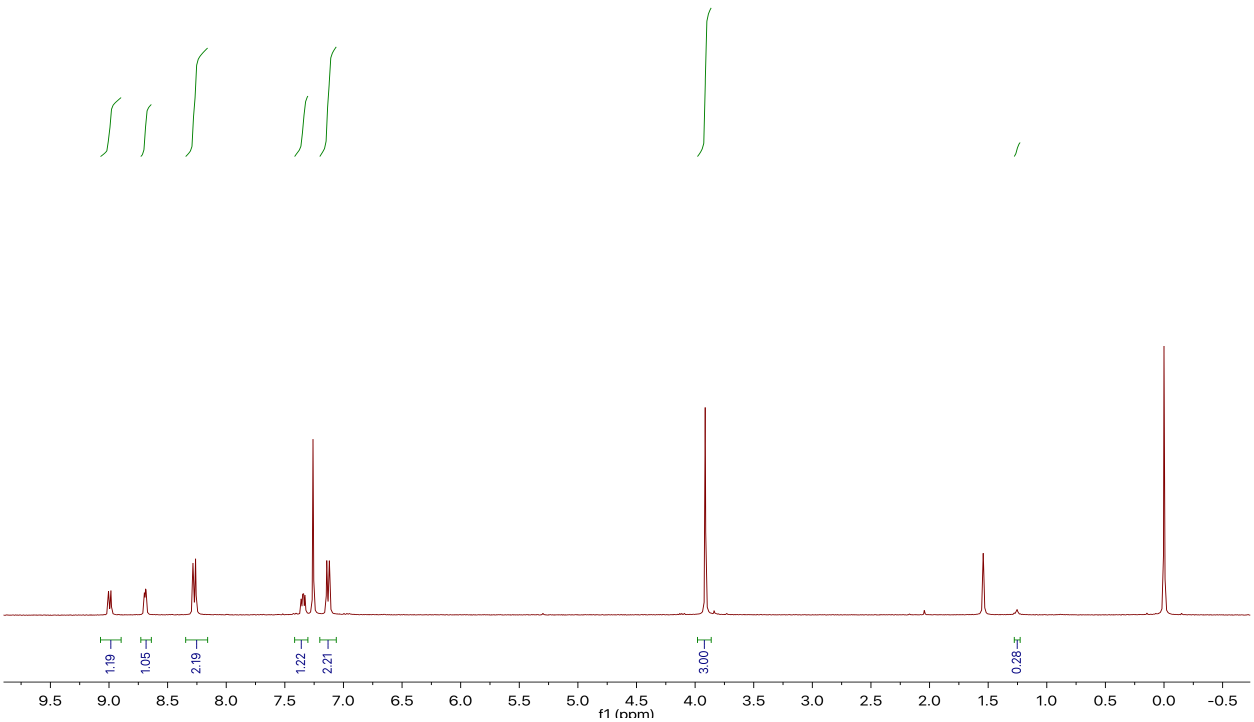

[13]C Spectrum of 1-(4-methoxyphenyl)-1*H*-pyrazolo[3,4-*b*]pyridine (**18d**)

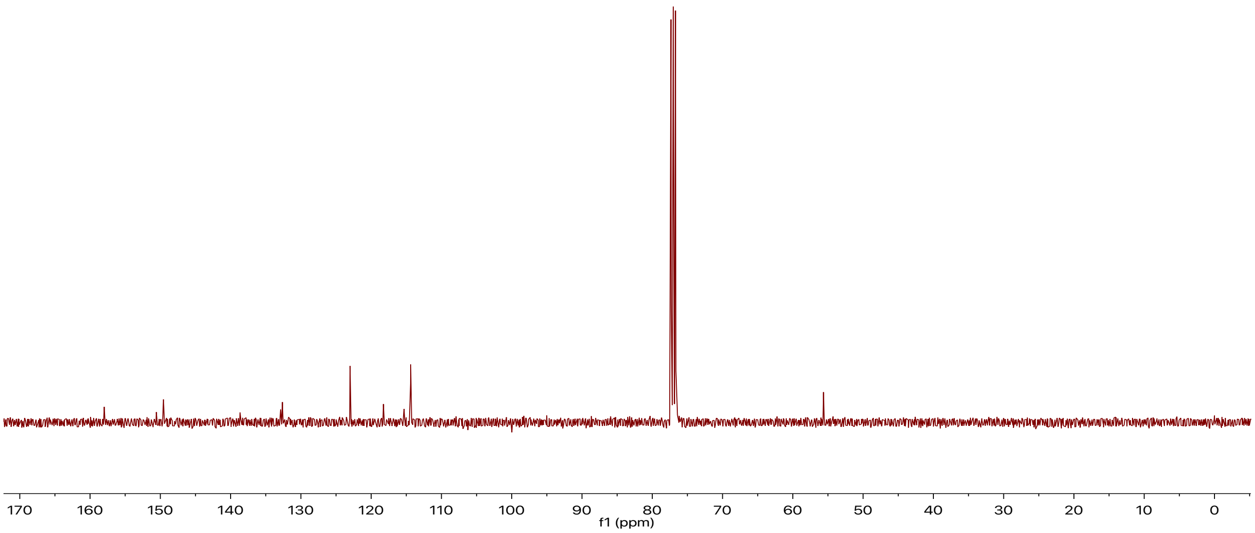

^{1}H Spectrum of 4-(1*H*-pyrazolo[3,4-*b*]pyridin-1-yl)benzonitrile (**18k**)

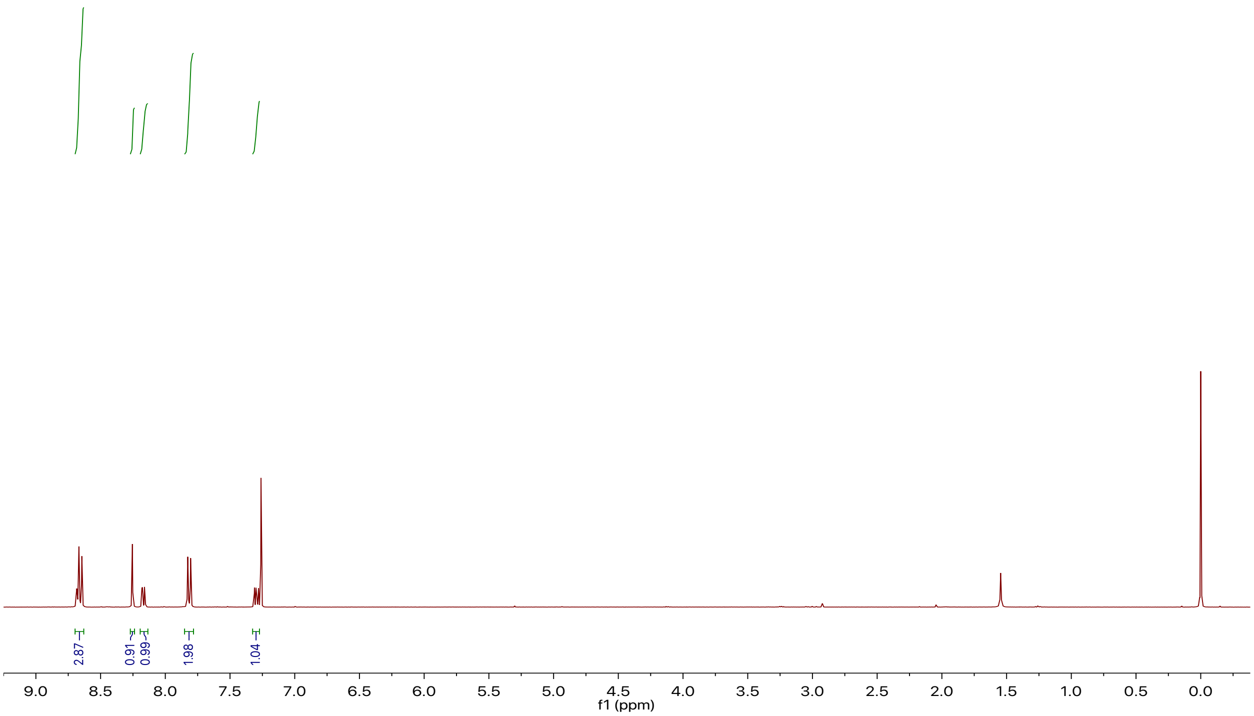

^{13}C Spectrum of 4-(1*H*-pyrazolo[3,4-*b*]pyridin-1-yl)benzonitrile (**18k**)

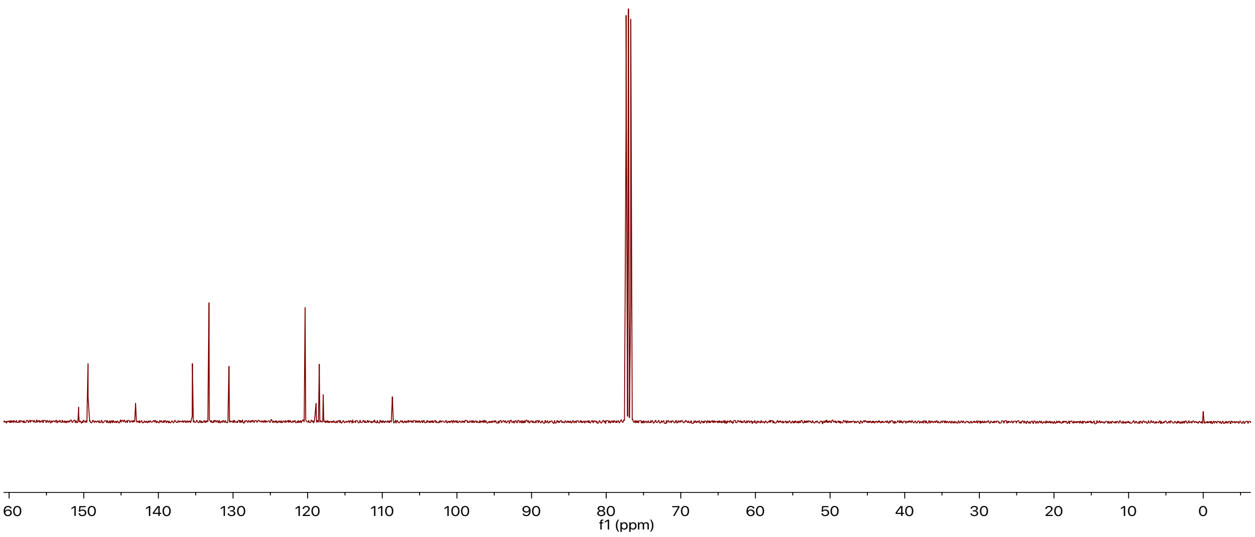

Supplement: Supplementary file 1 [file molecules-23-00674-s001.pdf]
